# Spartalizumab or placebo in combination with dabrafenib and trametinib in patients with *BRAF* V600-mutant melanoma: exploratory biomarker analyses from a randomized phase 3 trial (COMBI-i)

**DOI:** 10.1136/jitc-2021-004226

**Published:** 2022-06-21

**Authors:** Hussein A Tawbi, Caroline Robert, Jan C Brase, Daniel Gusenleitner, Eduard Gasal, James Garrett, Alexander Savchenko, Güllü Görgün, Keith T Flaherty, Antoni Ribas, Reinhard Dummer, Dirk Schadendorf, Georgina V Long, Paul D Nathan, Paolo A Ascierto

**Affiliations:** 1 The University of Texas MD Anderson Cancer Center, Houston, Texas, USA; 2 Gustave Roussy, Villejuif, and Paris-Saclay University, Orsay, France; 3 Novartis Pharma AG, Basel, Switzerland; 4 Novartis Institutes for BioMedical Research, Inc, Cambridge, Massachusetts, USA; 5 Novartis Pharmaceuticals Corporation, East Hanover, New Jersey, USA; 6 Novartis Pharmaceuticals Corporation, Cambridge, Massachusetts, USA; 7 Dana-Farber Cancer Institute/Harvard Medical School and Massachusetts General Hospital, Boston, Massachusetts, USA; 8 Jonsson Comprehensive Cancer Center, University of California, Los Angeles, Los Angeles, California, USA; 9 University Hospital Zürich Skin Cancer Center, Zürich, Switzerland; 10 University Hospital Essen, Essen, and German Cancer Consortium, Heidelberg, Germany; 11 Melanoma Institute Australia, The University of Sydney, and Royal North Shore and Mater Hospitals, Sydney, New South Wales, Australia; 12 Mount Vernon Cancer Centre, Northwood, UK; 13 Istituto Nazionale Tumori, IRCCS, Fondazione “G. Pascale", Naples, Italy

**Keywords:** Melanoma, Tumor Biomarkers, Drug Therapy, Combination

## Abstract

**Background:**

The randomized phase 3 COMBI-i trial did not meet its primary endpoint of improved progression-free survival (PFS) with spartalizumab plus dabrafenib and trametinib (sparta-DabTram) vs placebo plus dabrafenib and trametinib (placebo-DabTram) in the overall population of patients with unresectable/metastatic *BRAF* V600-mutant melanoma. This prespecified exploratory biomarker analysis was performed to identify subgroups that may derive greater treatment benefit from sparta-DabTram.

**Methods:**

In COMBI-i (ClinicalTrials.gov, NCT02967692), 532 patients received spartalizumab 400 mg intravenously every 4 weeks plus dabrafenib 150 mg orally two times daily and trametinib 2 mg orally one time daily or placebo-DabTram. Baseline/on-treatment pharmacodynamic markers were assessed via flow cytometry-based immunophenotyping and plasma cytokine profiling. Baseline programmed death ligand 1 (PD-L1) status and T-cell phenotype were assessed via immunohistochemistry; *BRAF* V600 mutation type, tumor mutational burden (TMB), and circulating tumor DNA (ctDNA) via DNA sequencing; gene expression signatures via RNA sequencing; and CD4^+^/CD8^+^ T-cell ratio via immunophenotyping.

**Results:**

Extensive biomarker analyses were possible in approximately 64% to 90% of the intention-to-treat population, depending on sample availability and assay. Subgroups based on PD-L1 status/TMB or T-cell inflammation did not show significant differences in PFS benefit with sparta-DabTram vs placebo-DabTram, although T-cell inflammation was prognostic across treatment arms. Subgroups defined by *BRAF* V600K mutation (HR 0.45 (95% CI 0.21 to 0.99)), detectable ctDNA shedding (HR 0.75 (95% CI 0.58 to 0.96)), or CD4^+^/CD8^+^ ratio above median (HR 0.58 (95% CI 0.40 to 0.84)) derived greater PFS benefit with sparta-DabTram vs placebo-DabTram. In a multivariate analysis, ctDNA emerged as strongly prognostic (p=0.007), while its predictive trend did not reach significance; in contrast, CD4^+^/CD8^+^ ratio was strongly predictive (interaction p=0.0131).

**Conclusions:**

These results support the feasibility of large-scale comprehensive biomarker analyses in the context of a global phase 3 study. T-cell inflammation was prognostic but not predictive of sparta-DabTram benefit, as patients with high T-cell inflammation already benefit from targeted therapy alone. Baseline ctDNA shedding also emerged as a strong independent prognostic variable, with predictive trends consistent with established measures of disease burden such as lactate dehydrogenase levels. CD4^+^/CD8^+^ T-cell ratio was significantly predictive of PFS benefit with sparta-DabTram but requires further validation as a biomarker in melanoma. Taken together with previous observations, further study of checkpoint inhibitor plus targeted therapy combination in patients with higher disease burden may be warranted.

**Trial registration number:**

NCT02967692.

**WHAT IS ALREADY KNOWN ON THIS TOPIC**

- First-line combination of immune checkpoint inhibitors and BRAF plus MEK inhibitor targeted therapies may not be an optimal approach for all patients with *BRAF* V600-mutant metastatic melanoma. Biomarkers to predict those patients most likely to benefit could help to inform treatment selection.

**WHAT THIS STUDY ADDS**

- This report describes preplanned exploratory biomarker analyses from the phase 3 COMBI-i trial of spartalizumab plus dabrafenib and trametinib vs placebo plus dabrafenib and trametinib, demonstrating the feasibility of such comprehensive analyses in the context of a phase 3 trial and identifying several potentially predictive and prognostic features.

**HOW THIS STUDY MIGHT AFFECT RESEARCH, PRACTICE AND/OR POLICY**

- These results provide a rationale for further investigation of CD4^+^/CD8^+^ T-cell ratio as a biomarker in melanoma and suggest that future studies of checkpoint inhibitor plus targeted therapy combination with patient selection based on tumor burden may be warranted.

## Background

Immune checkpoint inhibitors and BRAF plus MEK-targeted therapies have significantly improved long-term clinical outcomes in patients with unresectable or metastatic melanoma.^1–5^ Moreover, evidence suggests that within 2 weeks of initiation of targeted therapy, the tumor microenvironment is primed toward a proinflammatory state that could enhance antitumor responses driven by checkpoint blockade.^6–8^ Checkpoint inhibitor plus targeted therapy combinations vs targeted therapy alone have since been investigated in randomized phase 2 (KEYNOTE-022, NCT02130466)^9^ and phase 3 studies (IMspire150, NCT02908672; COMBI-i, NCT02967692)^10 11^ in patients with *BRAF* V600 mutation-positive unresectable or metastatic melanoma. Only IMspire150 met its primary endpoint, and modest improvements in progression-free survival (PFS) occurred at the cost of increased toxicity; overall survival (OS) data from phase 3 studies are not yet mature.^9–11^

Exploratory findings from COMBI-i parts 1 and 2 demonstrated on-treatment biomarker modulations, including increased expression of T-cell-inflamed signatures (TIS) and decreased mitogen-activated protein kinase (MAPK) pathway activity, in samples from patients treated with combination of the anti-programmed death receptor 1 (PD-1) monoclonal antibody spartalizumab, the BRAF inhibitor dabrafenib, and the MEK inhibitor trametinib (sparta-DabTram). The objective response rate with sparta-DabTram was 78% (28 of 36 patients), with 16 patients (44%) achieving a complete response.^12^ However, in COMBI-i part 3, the primary endpoint of improved investigator-assessed PFS vs placebo plus dabrafenib and trametinib (placebo-DabTram) was not met.^11^ Although the control arm performed better than expected based on historical dabrafenib plus trametinib data,^3 11^ a complete understanding of why the results from parts 1 and 2 did not translate to the larger randomized portion of the trial remains elusive. Through a comprehensive exploratory analysis of biospecimens from patients in the double-blind, randomized, placebo-controlled part 3 of COMBI-i, we sought to identify biomarkers that might better define patient populations more likely to derive therapeutic benefit from sparta-DabTram.

## Methods

### Study design

The global, phase 3 COMBI-i study (NCT02967692) includes a safety run-in (part 1), biomarker cohort (part 2), and randomized, double-blind, placebo-controlled part 3. Enrollment in part 3 occurred from September 13, 2017, to July 4, 2018, at 179 centers in 29 countries, with a total of 532 patients randomized 1:1 to receive the recommended phase 3 regimen of intravenous spartalizumab 400 mg or placebo every 4 weeks in combination with the approved doses of oral dabrafenib 150 mg two times daily and oral trametinib 2 mg one time daily.^12^ A random permuted block scheme and interactive response technology facilitated assignment of patient numbers to randomization numbers by investigators or study site staff. Patients, investigators, and study site staff remained blind to treatment identity from randomization until the primary analysis database lock.^11^

### Participants

Patients aged ≥18 years with histologically confirmed unresectable or metastatic (stage IIIC/IV per the American Joint Committee on Cancer’s *Cancer Staging Manual*, 7th edition) *BRAF* V600-mutant cutaneous melanoma were enrolled in COMBI-i part 3. Enrollment was based on *BRAF* status per local testing, with subsequent central confirmation. Additional eligibility criteria included no clinically active brain metastases, Eastern Cooperative Oncology Group performance status (ECOG PS) ≤2, and no prior systemic anticancer treatment for unresectable or metastatic melanoma. ECOG PS (0 vs 1 vs 2) and lactate dehydrogenase (LDH) levels (<1 × upper limit of normal (ULN) vs ≥1 to <2 × ULN vs ≥2 × ULN) were stratification factors.^11^

### Outcomes

The primary endpoint was investigator-assessed PFS per Response Evaluation Criteria in Solid Tumors version 1.1, defined as the time from randomization to first documented disease progression or death due to any cause. OS was a key secondary endpoint, defined as the time from randomization to death due to any cause.^11^ Efficacy by baseline programmed death ligand 1 (PD-L1) status was a secondary endpoint, and efficacy by tumor mutational burden (TMB) alone or in combination with PD-L1 status was a key exploratory endpoint. All other biomarker analyses were exploratory endpoints.

Biomarker analyses were conducted using tumor tissue and blood samples obtained from consenting patients. Collection of newly acquired (preferred) or archival (obtained at or since diagnosis, preferably within 3 months prior to study treatment) baseline tumor tissue samples during screening was mandatory. Additional on-treatment tumor sample collection (at 2–3 weeks, 8–12 weeks, or disease progression) was per investigator discretion. Only core, excisional, or incisional biopsies from tissue other than central nervous system or bone were acceptable. For tissue samples, the correlative analyses reported here focus on the mandatory baseline collection time point only, due to availability from most patients. Availability of results from baseline tissue samples depended on testing priority (central *BRAF* testing was performed before any other analysis) and sample size and quality. Collection of blood samples for circulating biomarker analyses (eg, tumor DNA, cytokine profiling, and flow cytometry) was mandatory at baseline, 4 weeks, 8 weeks, and disease progression.

### Immunohistochemistry

PD-L1 expression <1% or ≥1% was assessed using the immunohistochemistry (IHC) 28–8 pharmDx assay (Dako; Carpinteria, CA) on an Autostainer Link 48 (Agilent Technologies; Santa Clarita, CA) as implemented and validated at HistoGeneX (now CellCarta; Antwerp, Belgium) to follow US Food and Drug Administration-approved guidelines in the Premarket Approval Order Statement. The percentage of viable tumor cells expressing PD-L1 was scored in accordance with the *PD-L1 IHC 28–8 pharmDX Melanoma Interpretation Manual* (Dako). Discernible membrane staining of any intensity was included; cytoplasmic staining, immune cells, and necrotic cells were excluded. Negative and positive controls were reviewed to determine any interfering variables.

To assess levels of CD8^+^ immune cells within melanoma tumor nests and stromal compartments, a specific dualplex IHC assay, composed of an anti-CD8 rabbit monoclonal primary antibody (SP57, Ventana: Roche Diagnostics; Basel, Switzerland) and a Melanoma Triple Cocktail (HMB45, A103, and T311 antibodies), was performed on the Benchmark XT platform (Ventana) and quantified, including via infiltration analysis, using HALO software version 2.3 (Indica Labs; Albuquerque, NM). Evaluation of antigen-presenting cells (APCs) within defined tumor compartments was performed using multiplex fluorescence IHC by automated quantitative analysis at Navigate BioPharma, a Novartis subsidiary (Carlsbad, CA).

### RNA sequencing

Ribosomal RNA (rRNA) was depleted from extracted total RNA using RNase H (Sigma-Aldrich; St. Louis, MO). The rRNA-depleted sample was fragmented, converted to complementary DNA, and used to construct a next-generation sequencing library via the TruSeq RNA Library Prep Kit v2 (Illumina; San Diego, CA). The analysis included 1329 gene sets from MSigDB C2 Canonical Pathways V.6.2^13 14^ plus in-house and published gene sets.^12^ Pathway and gene set expression were derived using the geometric mean expression of all genes in each set. Pathways were ranked in unbiased analyses using two-sided Wilcoxon rank sum tests.

### NanoString testing and TIS

After isolation, ≤200 ng of RNA was combined with capture and reporter probes from the PanCancer IO 360 panel (NanoString Technologies; Seattle, WA) at 65°C overnight. Following hybridization, target-probe complexes were purified, conjugated to streptavidin-coated cartridges, and enumerated using the nCounter Analysis System (NanoString). TIS scores were calculated as previously described.^15 16^

### DNA sequencing and TMB

Samples were submitted to Foundation Medicine, Inc (Cambridge, MA), for next-generation sequencing with the FoundationOne CDx assay. TMB was determined by counting all synonymous and nonsynonymous variants present at ≥5% allele frequency and filtering out potential germline variants. Known and possible driver mutations were filtered out to exclude bias. The resulting mutation number was divided by the coding region corresponding to the number of total variants counted, or 793 kilobases, and reported as mutations per megabase (mut/Mb).

### Immunophenotyping by flow cytometric analysis

Peripheral blood mononuclear cells were isolated by Ficoll density gradient centrifugation and live-frozen in dimethyl sulfoxide (10%)/fetal bovine serum freezing buffer. Immunophenotyping was performed on baseline and week 4 paired samples using fluorochrome-conjugated monoclonal antibodies for cell-surface proteins and analyzed at Navigate BioPharma, a Novartis subsidiary.

### Cytokine profiling

Profiling of human cytokines was performed using a Meso Scale Diagnostics kit (Rockville, MD) and a multiplex sandwich electrochemiluminescence immunoassay (BioAgilytix; Durham, NC) validated at a clinical research organization selected by the study funder.

### Statistical analysis

The data cut-off for these analyses was July 1, 2020 (median follow-up, 27.2 months (IQR 25.4–29.0 months)). Contributions of biomarkers and covariates to PFS and OS were estimated using Cox proportional hazards models, univariate or multivariable as appropriate. Between-group comparisons were assessed by Wald or Wilcoxon rank sum tests with descriptive p values unadjusted for multiple comparisons. Biomarkers of potential predictive value were further assessed in multivariate analyses to determine statistical significance of the treatment interaction, and evidence for biomarker effects after adjusting for other factors was assessed using likelihood ratio tests with Cox models.

All biomarker analyses were performed using R 3.6.1 and Bioconductor 3.9. Kaplan-Meier curves and Cox proportional hazards models for biomarker cohorts were generated using the R survival (3.1-7) and survminer (0.4.6) packages. Population comparisons were evaluated using the R Hmisc (4.3.0) package.

Further methodological details are provided in the online supplemental materials.

10.1136/jitc-2021-004226.supp2 Supplementary data

## Results

A total of 532 patients were randomized to receive sparta-DabTram (n=267) or placebo-DabTram (n=265) (online supplemental figure S1); baseline characteristics were well balanced between treatment arms (online supplemental table S1).^11^ Because biomarker results were not available from all patients at all time points, we summarize in table 1 the availability for each analysis. Most patients were represented, with biomarker results available from approximately 64%–90% (339 to 481 of 532 patients). Key clinical and demographic variables were comparable between most biomarker cohorts and the respective subsets with no biomarker results available, although some cohorts included fewer samples from patients with poor prognostic features; for example, the subset lacking flow cytometry data at baseline was enriched for higher tumor burden characteristics, such as sum of lesion diameters and disease stage (online supplemental table S2).

10.1136/jitc-2021-004226.supp1 Supplementary data

**Table 1:** Summary of available biomarker results

	PD-L1	DNA-Seq (FoundationOne CDx)	NanoString (TIS)	Digital IHC (CD8)	Multiplex IHC	RNA-Seq	Flow Cytometry	Cytokine Profiling (n=21)	Targeted DNA-Seq (ctDNA)
Sample number, n	477	443 (TMB, 414)	433	419	339	395	Baseline, 409; week 4, 405	Baseline, 511; week 4, 479	Baseline, 481; week 8, 440
% of intention-to-treat population (N=532)	90	83 (TMB, 78)	81	79	64	74	Baseline, 77; week 4, 76	Baseline, 96; week 4, 90	Baseline, 90; week 8, 83

All numbers refer to baseline samples, unless otherwise indicated.

CD, cluster of differentiation; ctDNA, circulating tumor DNA; DNA-seq, DNA sequencing; IHC, immunohistochemistry; PD-L1, programmed death ligand 1; RNA-seq, RNA sequencing; TIS, T-cell-inflamed signature; TMB, tumor mutational burden.

As previously reported, COMBI-i did not meet its primary endpoint of improved investigator-assessed PFS with sparta-DabTram vs placebo-DabTram in the intention-to-treat population of patients with *BRAF* V600-mutant metastatic melanoma (HR 0.82 (95% CI 0.66 to 1.03); one-sided p*=*0.042).^11^ Preplanned subgroup analyses included in that report demonstrated that there were no significant differences in sparta-DabTram benefit regardless of PD-L1 status or TMB, although there was a trend toward greater benefit in patients with high TMB (≥10 mut/Mb).^11^ As a prespecified key exploratory endpoint, we further evaluated outcomes in subgroups based on combined PD-L1 status and TMB. Consistent with previous observations, patients with tumors characterized by low TMB did not derive PFS benefit from sparta-DabTram (PD-L1 negative (<1%)/TMB low: HR 1.11 (95% CI 0.71 to 1.75); PD-L1 positive/TMB low: HR 0.86 (95% CI 0.53 to 1.41)). In patients with tumors characterized by high TMB, sparta-DabTram was associated with numerically longer PFS independent of PD-L1 status, although these benefits vs placebo-DabTram were not significant (PD-L1 negative/TMB high: HR 0.71 (95% CI 0.38 to 1.32); PD-L1 positive/TMB high: HR 0.73 (95% CI 0.44 to 1.23)) (online supplemental figure S2A). An interim analysis suggested that sparta-DabTram was also associated with improved OS in the PD-L1-negative/TMB-high subgroup (HR 0.33 (95% CI 0.13 to 0.79)) (online supplemental figure S2B) but not in other PD-L1/TMB-defined subgroups. Analysis of the tumor microenvironment revealed lower baseline TIS levels,^15^ per NanoString TIS score, in PD-L1-negative tumors regardless of TMB, while multiplex fluorescence IHC demonstrated fewer APCs in PD-L1-negative/TMB-high tumors compared with all others (online supplemental figure S3).

Higher TMB and older age were associated with *BRAF* V600K-mutant (n=53) vs *BRAF* V600E-mutant (n=402) disease per central assessment (online supplemental figure S4). The V600K subgroup derived greater PFS benefit from sparta-DabTram than did the V600E subgroup (V600K: HR 0.45 (95% CI 0.21 to 0.99); V600E: HR 0.87 (95% CI 0.67 to 1.13)) (online supplemental figure S5A). OS benefit associated with sparta-DabTram was also greater in the V600K subgroup (V600K: HR 0.46 (95% CI 0.17 to 1.26); V600E: HR 0.84 (95% CI 0.60 to 1.18)) (online supplemental figure S5B), although the 95% CIs for both subgroups crossed 1.00 in this interim analysis. Gene expression signatures in the V600E and V600K subgroups were compared via RNA sequencing (online supplemental table S3). The SPRY-mediated negative feedback loop of the MAPK signaling pathway was the top pathway downregulated in the V600K subgroup compared with the V600E subgroup, suggesting comparatively decreased MAPK pathway activity (online supplemental figure S6). Given the association between *BRAF* V600K, older age, and high TMB as well as the previously observed trend toward greater PFS benefit with sparta-DabTram in patients with high TMB, a multivariate analysis was performed to evaluate the relative contribution of these variables to the treatment effect. Given age and TMB, *BRAF* V600K did not add additional predictive information (interaction p=0.7677).

**Figure 1:**
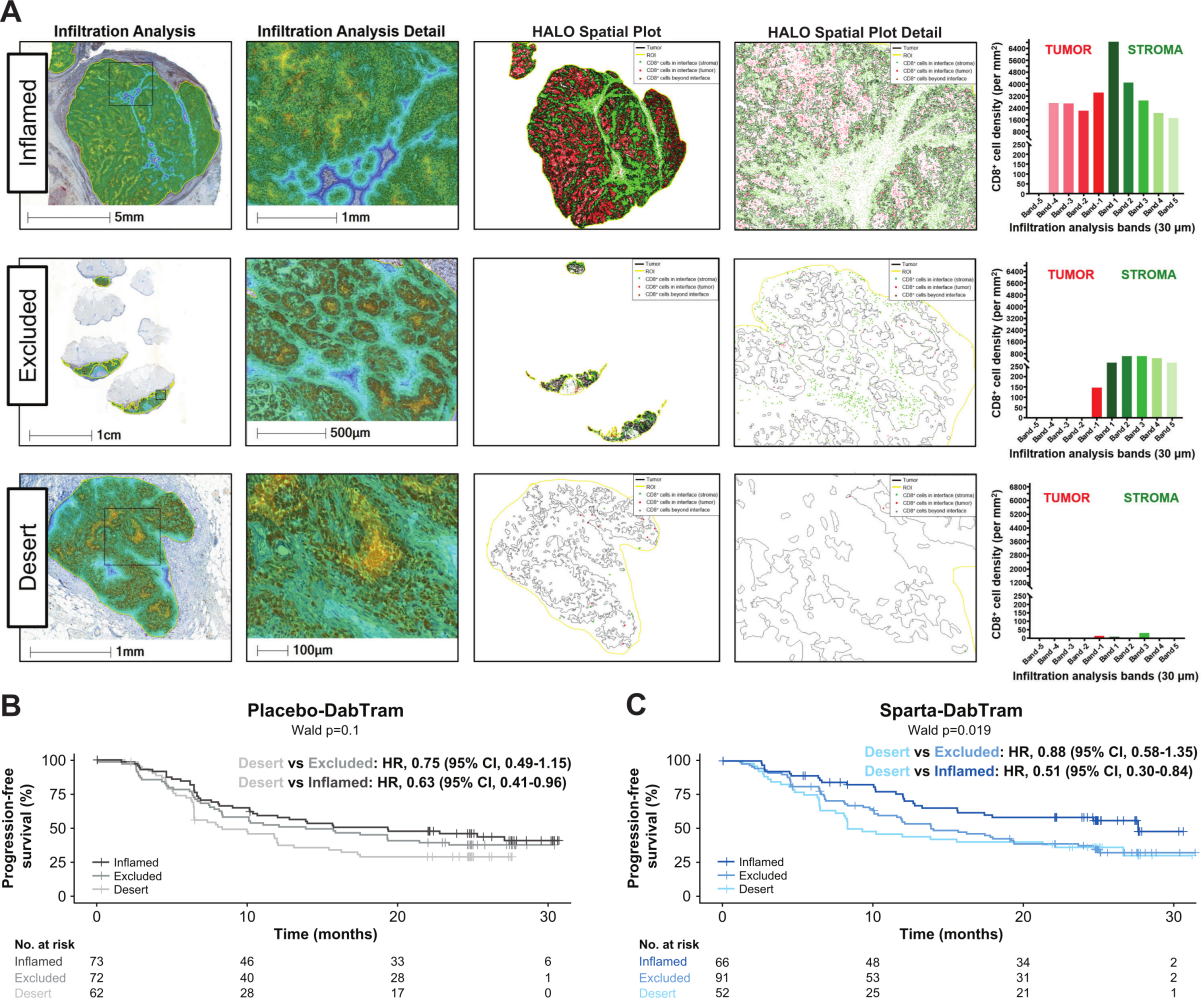
Characterization (A) and prognostic impact (B and C) of T-cell phenotypes. Representative samples of the inflamed, excluded, and desert T-cell phenotypes derived from digital pathology immunohistochemistry of tumor-infiltrating and stroma-infiltrating lymphocytes are shown in (A). Based on average CD8 density values, the top third of tumors were defined as inflamed; the bottom third of stroma and other samples were defined as desert; and samples between these thresholds were defined as excluded. Shown in (B and C) are Kaplan-Meier estimates of progression-free survival based on these phenotypes in the placebo-DabTram (B; inflamed, n=73; excluded, n=72; desert, n=62) and sparta-DabTram (C; inflamed, n=66; excluded, n=91; desert, n=52) treatment arms. CD, cluster of differentiation; placebo-DabTram, placebo plus dabrafenib and trametinib; sparta-DabTram, spartalizumab plus dabrafenib and trametinib.

In an unbiased analysis, a total of 2311 gene signatures and pathways were evaluated for prognostic value. Of the top 100 in each treatment arm, 49 were overlapping (online supplemental table S4; online supplemental figure S7), including the well-established TIS.^15^ Patients with lower TIS expression experienced relatively poor clinical outcomes in both treatment arms compared with patients with higher TIS levels (online supplemental figure S8). Given the prognostic role of the TIS, T-cell phenotypes were further characterized by digital pathology IHC. Infiltration analyses revealed that ‘inflamed’ tumor samples had substantial and homogenous CD8^+^ tumor-infiltrating lymphocyte distribution across melanoma tumor nests when assessed in multiple bands within 30–150 µm from the tumor margin (figure 1A). Patients with ‘inflamed’ or ‘excluded’ phenotypes within tumor and stromal compartments were likely to experience more favorable outcomes regardless of treatment arm (figure 1B, C). Sparta-DabTram conferred a greater benefit among patients with the ‘inflamed’ phenotype, but this was not significant (PFS HR 0.71 (95% CI 0.43 to 1.15); OS HR 0.67 (95% CI 0.35 to 1.28)) (online supplemental figure S8).

**Figure 2:**
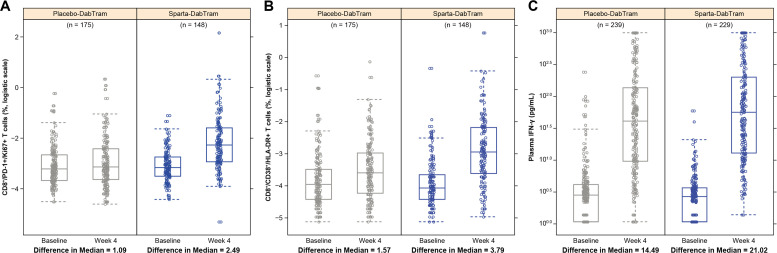
Immunophenotyping of peripheral blood mononuclear cells using markers for T-cell activation and proliferation (N=323) and cytokine profiling (N=468) of plasma samples taken at baseline and after 4 weeks of treatment. Shown are proliferating CD8^+^/PD-1^+^ T cells (A), activated cytotoxic CD8^+^ T cells (B), and plasma IFN-γ (C). CD, cluster of differentiation; HLA, human leukocyte antigen; IFN, interferon; PD-1, programmed death receptor 1; placebo-DabTram, placebo plus dabrafenib and trametinib; sparta-DabTram, spartalizumab plus dabrafenib and trametinib.

Immunophenotyping of peripheral blood mononuclear cells using preselected pharmacodynamic markers and cytokine profiling were performed at baseline and after 4 weeks of treatment to assess T-cell activation, proliferation, and cytotoxicity. Increased proliferating CD8^+^/PD-1^+^ T cells were observed from baseline to week 4 in patient samples from the sparta-DabTram arm (change in median, 2.49) compared with patient samples from the placebo-DabTram arm (change in median, 1.09) (figure 2A). CD8^+^ T cells with effector/cytotoxic phenotypes (CD38^+^/HLA-DR^+^) also increased from baseline to week 4 with sparta-DabTram (change in median, 3.79) vs placebo-DabTram (change in median, 1.57) (figure 2B). Total T-cell counts (CD3^+^) were within a normal range, with increased CD8^+^ and CD4^+^ proliferation and activation in the sparta-DabTram arm (online supplemental figure S9). In both arms, treatment resulted in enhanced cytotoxic effector T-cell modulation, reflected by increased plasma levels of interferon (IFN)-γ (figure 2C) and other cytokines (online supplemental figure S10); this modulation was more robust in the sparta-DabTram arm. These results suggest that spartalizumab induces immune effects that are similar to those reported with approved checkpoint inhibitors.^17^

**Figure 3:**
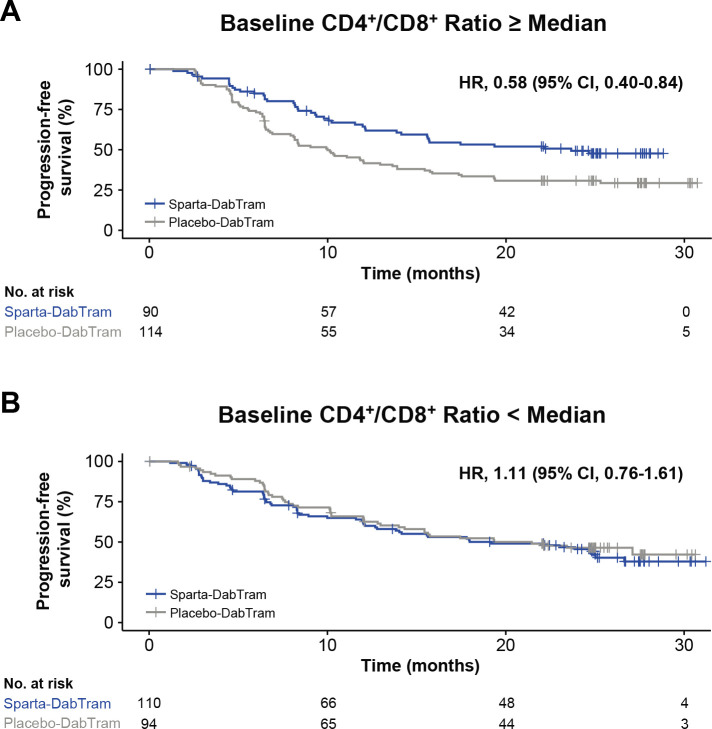
Progression-free survival based on baseline peripheral CD4^+^/CD8^+^ T cell ratios. Shown are Kaplan-Meier estimates of progression-free survival in patients randomized to either the sparta-DabTram or placebo-DabTram arm with peripheral blood mononuclear cell samples reflecting baseline CD4^+^/CD8^+^ T-cell ratios at or above the median (N=204) (A) or below the median (N=204) (B) value of 2.9 at baseline. CD, cluster of differentiation; placebo-DabTram, placebo plus dabrafenib and trametinib; sparta-DabTram, spartalizumab plus dabrafenib and trametinib.

Baseline systemic T-cell-mediated immune activity was also assessed via immunophenotyping through determination of peripheral blood helper/cytotoxic (CD4^+^/CD8^+^) T-cell ratios. A higher baseline CD4^+^/CD8^+^ ratio was associated with shorter PFS in the placebo-DabTram arm (online supplemental figure S11). However, no such association was observed with sparta-DabTram. Analysis of CD4^+^/CD8^+^ ratios between treatment arms suggested that the addition of spartalizumab to dabrafenib and trametinib may prolong PFS in patients with a baseline CD4^+^/CD8^+^ ratio at or above the median value (HR 0.58 (95% CI 0.40 to 0.84)) (figure 3).

**Figure 4:**
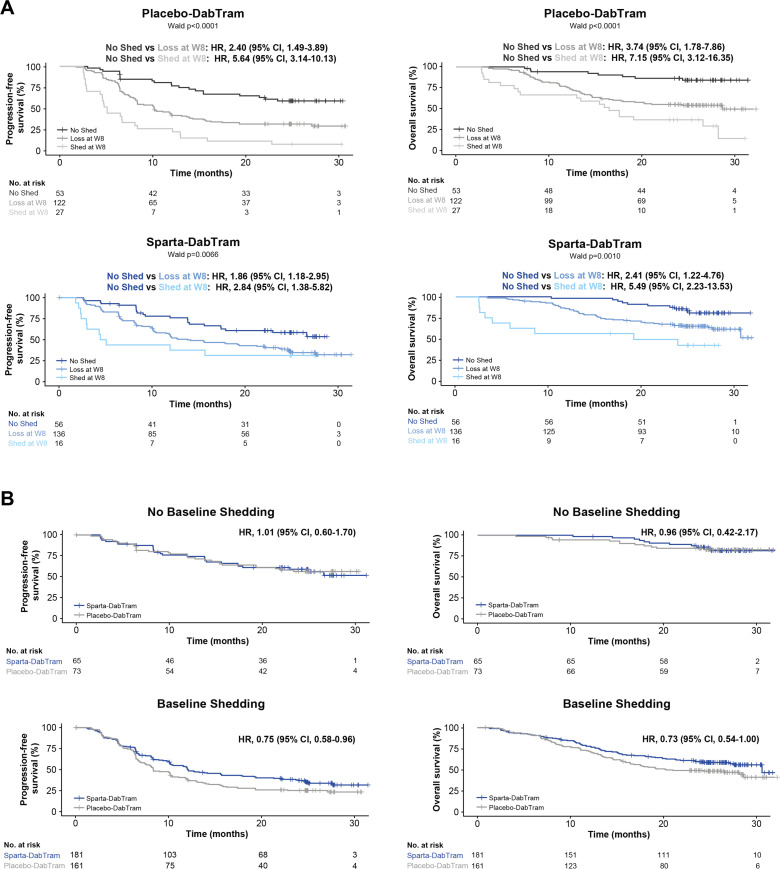
Predictive and prognostic value of baseline and on-treatment ctDNA shedding. (A) Kaplan-Meier estimates of progression-free survival (left) and overall survival (right) based on baseline and on-treatment ctDNA shedding in either the placebo-DabTram (top) or sparta-DabTram (bottom) arm. ‘No Shed’ indicates no ctDNA shedding observed at baseline or week 8 (placebo-DabTram, n=53; sparta-DabTram, n=56), ‘Loss at W8’ indicates shedding observed at baseline but not at week 8 (placebo-DabTram, n=122; sparta-DabTram, n=136), and ‘Shed at W8’ indicates shedding observed at both baseline and week 8 (placebo-DabTram, n=27; sparta-DabTram, n=16). (B) Kaplan-Meier estimates of progression-free survival (left) and overall survival (right) based on treatment with placebo-DabTram or sparta-DabTram in patients without (top; N=138) or with (bottom; N=342) baseline ctDNA shedding. ctDNA, circulating tumor DNA; placebo-DabTram, placebo plus dabrafenib and trametinib; sparta-DabTram, spartalizumab plus dabrafenib and trametinib.

Circulating tumor DNA (ctDNA) was also isolated from baseline and on-treatment blood samples. Measures reflective of higher disease burden correlated with baseline ctDNA levels (online supplemental figure S12), as did best overall response; patients who achieved a complete response had lower baseline ctDNA levels than patients with a partial response or stable disease (online supplemental figure S13). Both baseline and week 8 ctDNA shedding had prognostic value across treatment arms (figure 4A), an association that persisted after adjusting for measures of disease burden (LDH level and disease stage; table 2). Patients with no detectable ctDNA at baseline did not derive treatment benefit from sparta-DabTram (figure 4B), whereas those with detectable ctDNA showed improved outcomes (PFS HR 0.75 (95% CI 0.58 to 0.96); OS HR 0.73 (95% CI 0.54 to 1.00)). Given the predictive trends observed with both liquid biopsy-derived biomarkers assessed (ctDNA and CD4^+^/CD8^+^ ratio), potential predictive and prognostic value were further evaluated in a multivariate analysis together with the study stratification factors (LDH level and disease stage), both key clinical variables that have previously been implicated as prognostic. The prognostic value of both ctDNA and CD4^+^/CD8^+^ ratio emerged as significant in this analysis, while only CD4^+^/CD8^+^ ratio was significantly predictive (table 2).

**Table 2:** Multivariate analysis of liquid biomarkers

Category	P Value	Interaction P Value
LDH (given all else)	0.1408	–
LDH*treatment (given all else)	–	0.9334
ctDNA (given all else)	0.007	–
ctDNA*treatment (given all else)	–	0.3105
CD4^+^/CD8^+^ ratio (given all else)	0.043	–
CD4^+^/CD8^+^ ratio*treatment (given all else)	–	0.0131

Category	Coefficient	Exponential (coefficient)	Standard Error (coefficient)	Z-Statistic	Probability > │Z│
Treatment	1.0817	2.9497	0.7279	1.4859	0.1373
Stage IV M1a	–0.0846	0.9189	0.5747	–0.1472	0.883
Stage IV M1b	0.5803	1.7865	0.5513	1.0526	0.2925
Stage IV M1c (normal LDH)	0.8828	2.4176	0.4899	1.802	0.0715
Stage IV M1c (elevated LDH)	1.2818	3.6032	0.5253	2.4403	0.0147
Baseline CD4^+^/CD8^+^ ratio above median	0.3695	1.447	0.1921	1.9236	0.0544
Baseline ctDNA detectable	0.6626	1.9399	0.2352	2.8172	0.0048
Baseline CD4^+^/CD8^+^ ratio above median*treatment	–0.6999	0.4966	0.2848	–2.4577	0.014

Disease stage was also included in the model, as it was a stratification factor (along with LDH level). Hypotheses were tested using likelihood ratio tests. P<0.05 indicates significant prognostic value; interaction p<0.05 indicates significant predictive value (top). From the model including all factors of interest, coefficients identified as having evidence of non-null status are shown (bottom).

CD, cluster of differentiation; ctDNA, circulating tumor DNA; LDH, lactate dehydrogenase.

## Discussion

Investigation of checkpoint inhibitor plus targeted therapy combinations in *BRAF*-mutant melanoma was motivated by the observation that targeted therapy may lead to tumor microenvironmental changes favorable for immunotherapy.^7 8^ However, the results of three key clinical trials of such combinations have been reported at the time of this writing, showing only modest benefits vs targeted therapy alone.^9–11^ While these data collectively do not support routine use of first-line checkpoint inhibitor plus targeted therapy combinations, biomarkers that identify patient subgroups more likely to benefit remain an intriguing possibility. The results from COMBI-i reported here represent, to our knowledge, the largest prospectively collected biomarker data set from patients with metastatic melanoma and highlight several characteristics that may inform treatment selection, pending replication in prospective studies.

Based on Kaplan-Meier analyses and HRs for PFS, we identified three biomarkers with potential predictive value in the context of adding a checkpoint inhibitor to targeted therapy: detectable baseline ctDNA shedding, baseline CD4^+^/CD8^+^ T-cell ratio above median, and *BRAF* V600K mutation. With respect to ctDNA shedding, our Kaplan-Meier analyses suggest that it provides both prognostic and predictive information in this patient population. Although the predictive effect did not reach the level of significance in a subsequent multivariate analysis, ctDNA remained a strong and independent prognostic variable even when adjusting for key clinical variables previously identified as prognostic^18^ (which were also the study stratification factors: disease stage and LDH level). This positive prognostic value of low baseline and/or decreased on-treatment ctDNA levels aligns with observations in recent studies of either checkpoint inhibitors or targeted therapy alone.^19–21^

The trend toward greater survival benefit with sparta-DabTram vs placebo-DabTram in the subpopulation with detectable baseline ctDNA shedding complements the previously reported greater PFS benefit in patients with measures of clinically higher tumor burden, such as greater number of metastatic sites or sum of lesion diameters.^11^ In that analysis, there was also a predictive trend based on baseline LDH level (HR (LDH levels normal), 0.88; HR (LDH levels ≥1 to <2 × ULN), 0.78).^11^ Notably, the predictive trend based on baseline ctDNA in the present analysis was even stronger (HR (no shedding), 1.01; HR (shedding), 0.75). Thus, a strongly prognostic biomarker like ctDNA may also be helpful in identifying patients with lower tumor burden less likely to benefit from checkpoint inhibitor plus targeted therapy combination, given that patients with no detectable baseline ctDNA shedding appear to derive limited benefit.

Baseline CD4^+^/CD8^+^ T-cell ratio above median was the only biomarker in the present analysis that emerged as significantly predictive of sparta-DabTram benefit. Preclinical and clinical studies have highlighted this ratio, reflective of systemic immune activation, as an emerging predictive and prognostic marker in many cancers.^22–24^ In melanoma, intratumoral as well as blood CD4^+^/CD8^+^ ratio has been reported to associate with response to chemoimmunotherapy or radioimmunotherapy.^25 26^ Our study suggests that CD4^+^/CD8^+^ ratio may also be a useful noninvasive indicator of checkpoint inhibitor plus targeted therapy benefit, pending further validation.

There was also a PFS benefit with sparta-DabTram in our analysis of the comparatively small subset of patients with *BRAF* V600K-mutant disease, who are typically older and have higher TMB.^27^ This finding is consistent with a previous study that demonstrated differential benefits of checkpoint inhibition and targeted therapy among patients with *BRAF* V600K-mutant vs V600E-mutant disease.^27^ Both that study and ours implicate MAPK pathway activation in these effects. However, multivariate analysis suggests that *BRAF* V600K mutation itself does not add significant predictive information given other factors; the small size of this subgroup limits further interpretation.

Several biomarkers we evaluated proved to be generally prognostic rather than predictive of sparta-DabTram benefit. It is well established that tumors with features such as low PD-L1 expression, TMB, and TIS expression do not respond as well to checkpoint inhibitors.^15 16 28 29^ The predictive value of these biomarkers for targeted therapy is less definitive, although higher levels of tumor immune markers were associated with greater treatment benefit in the phase 3 COMBI-AD (dabrafenib plus trametinib) trial.^30^ In our analyses, higher levels of intratumoral T cells (assessed via TIS or IHC) were positively prognostic regardless of treatment arm. Thus, as patients with T-cell-inflamed tumors already benefit from targeted therapy alone, addition of spartalizumab may not provide additional benefit, potentially contributing to the limited treatment benefit observed with sparta-DabTram in the overall patient population.

Similarly, no subgroup defined by PD-L1/TMB status, alone or in combination, derived a significant PFS benefit from sparta-DabTram vs placebo-DabTram, although the trend favoring patients with high TMB, particularly the PD-L1-negative/TMB-high subgroup, was consistent with observations previously reported for other checkpoint inhibitor plus targeted therapy combinations.^18^ An OS benefit with sparta-DabTram was observed only in the PD-L1-negative/TMB-high subgroup, suggesting that in all other subgroups, targeted therapy followed by immunotherapy—a sequence received by most patients in the placebo-DabTram arm^11^—may be as effective as up-front combination. High TMB is associated with a higher mutation frequency and thus a greater likelihood of acquired resistance to targeted therapy; on the other hand, targeted therapy leads to cell death and antigen presentation, so high TMB could also drive immunogenic potential, although our analysis found that baseline TIS and APC levels were lowest in the PD-L1-negative/TMB-high subgroup. Notably, in COMBI-AD, adjuvant dabrafenib plus trametinib was of limited benefit in a subgroup defined by IFN-γ-low/TMB-high disease.^30^ As this subgroup is comparable to the PD-L1-negative/TMB-high subgroup in the present analysis, our findings that up-front rather than second-line use of a checkpoint inhibitor had an OS benefit only in these patients are consistent with those results; factors such as small subgroups, lack of a checkpoint inhibitor comparator arm, and the interim nature of the OS analysis preclude definitive conclusions on this point but may serve as a foundation for future investigation.

Overall, our results highlight the ability of biomarker analyses to define patient populations that may be more likely to benefit from a given treatment and demonstrate the feasibility of such comprehensive analyses in a global phase 3 study. Greater treatment benefit with sparta-DabTram was observed primarily in patients with high tumor burden, characterized by elevated LDH levels, detectable baseline ctDNA shedding, or clinical measures. CD4^+^/CD8^+^ T-cell ratio also appeared to be strongly predictive of sparta-DabTram benefit, although further validation of this biomarker in melanoma is required, while features such as baseline ctDNA shedding and T-cell inflammation were prognostic. Thus, future prospective randomized studies of checkpoint inhibitor plus targeted therapy combination with patient selection based on tumor burden may warrant further consideration.

## Data Availability

Data are available on reasonable request. Novartis is committed to sharing, with qualified external researchers, access to patient-level data and supporting clinical documents from eligible studies. Requests are reviewed and approved by an independent review panel on the basis of scientific merit. All data provided are anonymized to respect the privacy of patients who have participated in the trial in line with applicable laws and regulations. This trial data availability is according to the criteria and process described on ClinicalStudyDataRequest.com.
