# Supplementary material for: Spartalizumab or placebo in combination with dabrafenib and trametinib in patients with BRAF V600-mutant melanoma: exploratory biomarker analyses from a randomized phase 3 trial (COMBI-i)
Source: J Immunother Cancer. 2022 Jun 21;10(6):e004226. doi: 10.1136/jitc-2021-004226 (PMC9214378; doi:10.1136/jitc-2021-004226)
Supplement: Supplementary data [file jitc-2021-004226supp002.pdf]

NOVARTIS

Clinical Development

PDR001, dabrafenib, trametinib

Oncology Clinical Trial Protocol CPDR001F2301

A randomized, double-blind, placebo-controlled, phase III study comparing the combination of PDR001, dabrafenib and trametinib versus placebo, dabrafenib and trametinib in previously untreated patients with unresectable or metastatic *BRAF* V600 mutant melanoma

Document type	Amended Protocol (Clean)
EUDRACT number	2016-002794-35
Version number	05 (Amendment 5)
Development phase	III
Document status	Final
Release date	08-Mar-2019

Property of Novartis
Confidential
May not be used, divulged, published or otherwise disclosed
without the consent of Novartis

Template version 22-Jul-2016

Table of contents

Table of contents 2
List of figures 7
List of tables 7
List of abbreviations 11
Glossary of terms 15
Protocol summary 17
Amendment 5 (08-Mar-2019) 21
Amendment 4 (14-Aug-2018) 24
Amendment 3 (14-Jul-2017) 28
Amendment 2 (07-Mar-2017) 30
Amendment 1 (27-Jan-2017) 37
1 Background 39
1.1 Overview of disease pathogenesis, epidemiology and current treatment 39
1.2 Introduction to investigational treatment(s) and other study treatment(s) 41
1.2.1 Overview of PDR001 41
1.2.2 Overview of dabrafenib and trametinib 43
2 Rationale 48
2.1 Study rationale and purpose 48
2.2 Rationale for the study design 51
2.3 Rationale for dose and regimen selection 52
2.4 Rationale for choice of combination drugs 53
2.5 Rationale for choice of comparator drugs 53
2.6 Risks and benefits 54
3 Objectives and endpoints 55
4 Study design 60
4.1 Description of study design 60
4.1.1 Screening phase 63
4.1.2 Treatment phase 63
4.1.3 End of treatment 64
4.1.4 Safety follow-up 64
4.1.5 Efficacy follow-up 64
4.2 Timing of interim analyses and design adaptations 64
4.3 Definition of end of study 64
4.4 Early study termination 65
5 Population 65

5.1 Subject population ... 65
5.2 Inclusion criteria ... 65
5.2.1 Specific inclusion criteria for safety run-in (part 1) ... 65
5.2.2 Specific inclusion criteria for biomarker cohort (part 2) ... 66
5.2.3 Specific inclusion criteria for double-blind, randomized, placebo-controlled part (part 3) ... 66
5.2.4 Inclusion criteria for all subjects ... 66
5.3 Exclusion criteria ... 67
5.3.1 Specific exclusion criteria for safety run-in (part 1) ... 67
5.3.2 Specific exclusion criteria for the biomarker cohort (part 2) and the double-blind, randomized, placebo-controlled part (part 3) ... 67
5.3.3 Exclusion criteria for all subjects ... 67
6 Treatment ... 70
6.1 Study treatment ... 70
6.1.1 Dosing regimen ... 71
6.1.2 Ancillary treatments ... 73
6.1.3 Rescue medication ... 73
6.1.4 Guideline for continuation of treatment ... 73
6.1.5 Treatment duration ... 74
6.2 Determination of recommended regimen for the randomized part of the study ... 74
6.2.1 Starting dose rationale ... 74
6.2.2 Provisional dose levels ... 75
6.2.3 Guidelines for determination of recommended regimen for part 3 ... 75
6.2.4 Definition of dose limiting toxicities in the safety-run in part (part 1) ... 76
6.3 Dose modifications and reductions ... 77
6.3.1 General dose modification instructions for PDR001 or placebo ... 80
6.3.2 General dose modification instructions for dabrafenib and trametinib: ... 81
6.3.3 Dose modification and management guideline for adverse events common to PDR001, dabrafenib and trametinib ... 81
6.3.4 Mandatory dose modifications and recommended clinical management guidelines for infusion reactions ... 100
6.3.5 Dose modification and management guideline for adverse events suspected to be related to dabrafenib and/or trametinib ... 102
6.3.6 Follow-up for toxicities ... 110
6.4 Concomitant medications ... 112
6.4.1 Permitted concomitant therapy ... 112

6.4.2 Permitted concomitant therapy requiring caution and/or action 113
6.4.3 Prohibited concomitant therapy 114
6.4.4 Use of bisphosphonates (or other concomitant agents) 114
6.5 Subject numbering, treatment assignment or randomization 115
6.5.1 Subject numbering 115
6.5.2 Treatment assignment or randomization 115
6.5.3 Treatment blinding 116
6.6 Study drug preparation and dispensation 116
6.6.1 Study treatment packaging and labeling 117
6.6.2 Drug supply and storage 118
6.6.3 Study drug compliance and accountability 118
6.6.4 Disposal and destruction 119
7 Visit schedule and assessments 119
7.1 Study flow and visit schedule 119
7.1.1 Molecular pre-screening 165
7.1.2 Screening 165
7.1.3 Treatment period 167
7.1.4 Discontinuation of study treatment 168
7.1.5 Withdrawal of study consent 170
7.1.6 Follow up for safety evaluations 170
7.1.7 Follow up for efficacy evaluations 171
7.1.8 Survival follow-up 171
7.1.9 Lost to follow-up 171
7.2 Assessment types 171
7.2.1 Efficacy assessments 171
7.2.2 Safety and tolerability assessments 176
7.2.3 Pharmacokinetics 182
7.2.4 Biomarkers 189
7.2.5 Patient reported outcomes 199
8 Safety monitoring and reporting 200
8.1 Adverse events 200
8.1.1 Definitions and reporting 200
8.1.2 Laboratory test abnormalities 202
8.1.3 Adverse events of special interest 202
8.2 Serious adverse events 204
8.2.1 Definitions 204

8.2.2 Reporting 204
8.3 Emergency unblinding of treatment assignment 205
8.4 Pregnancies 206
8.5 Warnings and precautions 206
8.6 Data Monitoring Committee 207
8.7 Steering Committee 207
9 Data collection and management 208
9.1 Data confidentiality 208
9.2 Site monitoring 208
9.3 Data collection 209
9.4 Database management and quality control 209
10 Statistical methods and data analysis 210
10.1 Analysis sets 211
10.1.1 Full Analysis Set 211
10.1.2 Safety set 211
10.1.3 Per-Protocol set 211
10.1.4 Dose-determining analysis set 212
10.1.5 Pharmacokinetic analysis set 212
10.1.6 Other analysis sets 213
10.2 Subject demographics/other baseline characteristics 213
10.3 Treatments (study treatment, concomitant therapies, compliance) 213
10.4 Primary objective 214
10.4.1 Variables 214
10.4.2 Statistical hypothesis, model, and method of analysis 215
10.4.3 Handling of missing values/censoring/discontinuations 217
10.4.4 Supportive and sensitivity analyses 218
10.5 Secondary objectives 219
10.5.1 Key secondary objectives 219
10.5.2 Other secondary efficacy objectives 220
10.5.3 Safety objective(s) 222
10.5.4 Pharmacokinetics 224
10.5.5 Immunogenicity 225
10.5.6 Biomarkers 225
10.5.7 Resource utilization 227
10.5.8 Patient-reported outcomes 227
10.6 Exploratory PK and immunogenicity objectives 228

10.7 Interim analysis....228
10.7.1 Progression-Free Survival....228
10.7.2 Overall Survival (OS)....229
10.8 Sample size calculation....230
10.9 Power for analysis of key secondary variables....231
11 Ethical considerations and administrative procedures....232
11.1 Regulatory and ethical compliance....232
11.2 Responsibilities of the investigator and IRB/IEC/REB....232
11.3 Informed consent procedures....233
11.4 Discontinuation of the study....233
11.5 Publication of study protocol and results....233
11.6 Study documentation, record keeping and retention of documents....234
11.7 Confidentiality of study documents and subject records....235
11.8 Audits and inspections....235
11.9 Financial disclosures....235
12 Protocol adherence....235
12.1 Amendments to the protocol....236
13 References (available upon request)....237
14 Appendices....243
14.1 Appendix 1: RECIST 1.1....243
14.1.1 Introduction....245
14.1.2 Efficacy assessments....245
14.1.3 Efficacy definitions....254
14.1.4 Data handling and programming rules....263
14.1.5 References (available upon request)....267
14.2 Appendix 2: The Bayesian logistic regression model, prior, and dosing regimen recommendations in hypothetical scenarios....268
14.2.1 Introduction....269
14.2.2 Statistical model....269
14.2.3 Prior specifications....270
14.2.4 Hypothetical scenarios....280
14.2.5 References....283
14.3 Appendix 3: Safety follow up flow chart....284
14.4 AJCC Melanoma Staging System (Edition 8)....285

List of figures

Figure 4-1 Part 1: Safety run-in overview of study design 61
Figure 4-2 Part 2: Biomarker cohort overview of study design 62
Figure 4-3 Part 3: Double blind, randomized, placebo-controlled part overview of study design 62

List of tables

Table 3-1 Objectives and related endpoints 56
Table 6-1 Dose and treatment schedule 71
Table 6-2 Part 1 (safety run-in part): Provisional dose levels 71
Table 6-3 Part 2 (biomarker cohort): Dose and treatment schedule 71
Table 6-4 Part 3 (randomized part): Dose and treatment schedule 72
Table 6-5 Provisional dose levels 75
Table 6-6 Criteria for defining dose-limiting toxicities 76
Table 6-7 Dose modification steps for PDR001/placebo, dabrafenib and trametinib 79
Table 6-8 Reference of AEs and toxicity management guidelines 80
Table 6-9 Mandatory dose modifications and recommended clinical management guidelines for diarrhea/colitis 82
Table 6-10 Mandatory dose modifications and recommended clinical management guidelines for abnormal liver enzyme test 84
Table 6-11 Mandatory dose modifications and recommended clinical management guidelines for rash 85
Table 6-12 Mandatory dose modifications and recommended clinical management guidelines for renal function alterations 87
Table 6-13 Mandatory dose modifications and recommended clinical management guidelines for pneumonitis 89
Table 6-14 Mandatory dose modifications and recommended clinical management guidelines for encephalitis 90
Table 6-15 Mandatory dose modifications and recommended clinical management guidelines for endocrine events 92
Table 6-16 Mandatory dose modifications and recommended clinical management guidelines for hand foot skin reaction (palmar-plantar erythrodysesthesia) 93
Table 6-17 Mandatory dose modifications and recommended clinical management guidelines for neutropenia and thrombocytopenia 94
Table 6-18 Mandatory dose modifications and recommended clinical management guidelines for adverse events including abnormal laboratory related AEs 97

Table 6-19	Mandatory dose modifications and recommended clinical management guidelines for other AEs of special interest	99
Table 6-20	Mandatory dose modifications and recommended clinical management guidelines for infusion reaction and cytokine release syndromes	101
Table 6-21	General guidelines for dose modification for adverse events suspected to be related to dabrafenib and/or trametinib treatment	103
Table 6-22	Mandatory dose modification and recommended clinical management for changes in LVEF suspected to be related to dabrafenib and/or trametinib treatment	104
Table 6-23	Mandatory dose modification and recommended clinical management for hypertension suspected to be related to dabrafenib and/or trametinib treatment	105
Table 6-24	Mandatory dose modification and recommended clinical management for pyrexia syndrome suspected to be related to dabrafenib and/or trametinib treatment	107
Table 6-25	Mandatory dose modification and recommended clinical management for visual changes and/or ophthalmic examination findings suspected to be related to dabrafenib and/or trametinib treatment	109
Table 6-26	Mandatory dose modification and recommended clinical management for retinal pigment epithelial detachments (RPED) suspected to be related to trametinib treatment	109
Table 6-27	Packaging and labeling	118
Table 7-1	Visit evaluation schedule: Safety run-In part (Part 1)	120
Table 7-2	Visit evaluation schedule: Biomarker cohort (Part 2)	132
Table 7-3	Visit evaluation schedule: Double-blind, randomized, placebo-controlled part (Part 3)	144
Table 7-4	Visit evaluation schedule: Crossover (only if statistically significant interim or final OS results are demonstrated)	156
Table 7-5	Imaging assessment collection plan for Parts 1, 2, 3 and Crossover	173
Table 7-6	ECOG performance status scale	177
Table 7-7	Clinical laboratory parameters collection plan for all 3 parts	178
Table 7-8	ECG collection plan for safety run-in (part 1), biomarker cohort (part 2), and randomized part (part 3)	181
Table 7-9	ECG collection plan for crossover	181
Table 7-10	Parts 1 & 2 Safety run-in and biomarker cohorts: Blood (serum) collection schedule for PDR001 PK and IG	183
Table 7-11	Part 3 Double-blind, randomized, placebo-controlled part: Blood (serum) collection schedule for PDR001/placebo PK and IG	184

Table 7-12	Crossover subjects only: Blood (serum) collection schedule for PDR001 PK and IG	185
Table 7-13	Part 1 & 2 Safety run-in and biomarker cohorts: Pharmacokinetic blood (plasma) collection schedule for trametinib and dabrafenib	186
Table 7-14	Part 3 Double-blind, randomized, placebo-controlled part: Pharmacokinetic blood (plasma) collection schedule for trametinib and dabrafenib	187
Table 7-15	Crossover subjects only: Pharmacokinetic blood (plasma) collection schedule for trametinib and dabrafenib	188
Table 7-16	Biomarker sample collection plan for Safety run-in (part 1) and double-blind, randomized, placebo-controlled parts (part 3)	189
Table 7-17	Biomarker sample collection plan for Biomarker cohort (part 2)	193
Table 10-1	Estimated timelines for interim and final PFS analyses	229
Table 14-1	Response criteria for target lesions	250
Table 14-2	Response criteria for non-target lesions	252
Table 14-3	Overall lesion response at each assessment	253
Table 14-4	Options for event dates used in PFS, TTP, duration of response	261
Table 14-5	Prior distributions for the parameters of the MAP model used to derive the prior for the single agent dabrafenib model parameters	271
Table 14-6	Historical data from dabrafenib single agent clinical study	272
Table 14-7	Prior distributions for the parameters of the MAP model used to derive the prior for the single agent trametinib model parameters	272
Table 14-8	Historical data from trametinib single agent clinical study	273
Table 14-9	Prior distributions for the parameters of the MAP model used to derive the prior for the single agent PDR001 model parameters for model 1 (dosing regimen 1)	273
Table 14-10	Prior distributions for the parameters of the MAP model used to derive the prior for the single agent PDR001 model parameters for model 2 (dosing regimen -1a)	274
Table 14-11	Prior distributions for the parameters of the MAP model used to derive the prior for the single agent PDR001 model parameters for model 3 (dosing regimen -1b)	274
Table 14-12	Historical data from PDR001 Q4W single agent clinical study	275
Table 14-13	Historical data for combination of dabrafenib and trametinib	277
Table 14-14	Prior distribution for model parameters: model 1 (dosing regimen 1)	277
Table 14-15	Summary of prior distribution of DLT rates (with dabrafenib fixed at 150 mg BID and trametinib fixed at 2 mg QD) for model 1 (dosing regimen 1)	278

Supplemental material
BMJ Publishing Group Limited (BMJ) disclaims all liability and responsibility arising from any reliance placed on this supplemental material which has been supplied by the author(s)
J Immunother Cancer

Table 14-16 Prior distribution for model parameters: model 2 (dosing regimen -1a)....278
Table 14-17 Summary of prior distribution of DLT rates (with dabrafenib fixed at 150 mg BID and trametinib fixed at 2 mg QD) for model 2 (dosing regimen -1a) 278
Table 14-18 Prior distribution for model parameters: model 3 (dosing regimen -1b) 279
Table 14-19 Summary of prior distribution of DLT rates (with dabrafenib fixed at 150 mg BID and trametinib fixed at 2 mg QD) for model 3 (dosing regimen -1b) 279
Table 14-20 Hypothetical scenarios (with dabrafenib fixed at 150 mg BID and trametinib fixed at 2 mg QD) for all 3 dosing regimens, given 0-6 DLT's are observed in the first 6 patients receiving dosing regimen 16-6 280
Table 14-21 Hypothetical scenarios (with dabrafenib fixed at 150 mg BID and trametinib fixed at 2 mg QD) for cohorts -1a and -1b, given 3 DLT's are observed in first 6 patients receiving dosing regimen 1 281
Table 14-22 Definition of primary tumor (T)....285
Table 14-23 Definition of Regional Lymph Node (N)....286
Table 14-24 Definition of Distant Metastasis (M) 287
Table 14-25 Pathological (pTNM) 288

List of abbreviations

ADL	Activities of Daily Living
ADME	Absorption, Distribution, Metabolism and Excretion
ADA	Anti-Drug Antibodies
AE	Adverse Event
AESI	Adverse Events of Special Interest
AJCC	American Joint Committee on Cancer
ALT	Alanine Aminotransferase
ANC	Absolute Neutrophil Count
ASCO	American Society of Clinical Oncology
AST	Aspartate Aminotransferase
ATC	Anatomical Therapeutic Chemical
AV	Atrioventricular
AUC	Area Under the Concentration-time Curve
BID	*bis in diem*/twice a day
BLRM	Bayesian Logistic Regression Model
BLQ	Below Limit of Quantification
BP	Blood Pressure
BSA	Body Surface Area
BUN	Blood Urea Nitrogen
CD4	Cluster of Differentiation 4
CD8	Cluster of Differentiation 8
CDP	Clinical Development Plan
CI	Confidence interval
CT	Computed Tomography
ctDNA	Circulating Tumor DNA
ctRNA	Circulating Tumor RNA
Cmax	Maximum Concentration
CMO&PS	Chief Medical Office and Patient Safety
CNS	Central Nervous System
CRF	Case Report/Record Form
CRO	Contract Research Organization
CRP	C-Reactive Protein
CRS	Case Retrieval Strategy
CSR	Clinical Study Report
CSP	Clinical Study Protocol
CTCAE	Common Terminology Criteria for Adverse Events
CTLA-4	Cytotoxic T Lymphocyte Antigen 4
CV	Coefficient of Variation
CYPs	Cytochromes P450
D + T	Dabrafenib and trametinib
D5W	Dextrose 5% in Water
DAR	Dosage Administration Record
DBP	Diastolic Blood Pressure
DCR	Disease Control Rate
DDI	Drug-Drug Interaction
DILI	Drug Induced Liver Injury

DDS	Dose-Determining Set
DLT	Dose Limiting Toxicity
DLRT	Dose Level Review Team
DMC	Data Monitoring Committee
DOR	Duration of Response
DTIC	Dacarbazine
DVT	Deep Vein Thrombosis
EC	Ethics Committee
ECG	Electrocardiogram
ECHO	Echocardiogram
ECOG-PS	Eastern Cooperative Oncology Group Performance Status
eCRF	Electronic Case Report Form
EEA	European Economic Area
EMA	European Medicines Agency
EORTC	European Organization for Research and Treatment of Cancer
EOT	End of Treatment
ESA	Erythropoiesis Stimulating Agent
eSAE	Electronic Serious Adverse Event
EQ-5D	Quality of life questionnaire consisting of five dimensions (mobility, self-care, usual activities, pain/discomfort, anxiety/depression) offered by EuroQoL group
EWOC	Escalation With Overdose Control
FACT-M	Functional Assessment of Cancer Therapy–Melanoma
FAS	Full Analysis Set
FDG-PET	FluoroDeoxyGlucose Positron Emission Tomography
FFPE	Formalin Fixed Paraffin Embedded
G-CSF	Granulocyte Colony-Stimulating Factor
GDPR	General Data Protection Regulation
GI	Gastrointestinal
GLP	Good Laboratory Practice
GM-CSF	Granulocyte-Macrophage Colony-Stimulating Factor
HFSR	Hand Foot Skin Reactions
HGB	Hemoglobin
HLA class 2	Human Leukocyte Antigen class 2
HNSCC	Head and Neck Squamous Cell Carcinoma
HNSTD	Highest Non-Severely Toxic Dose
HR	Hazard Ratio
HRQoL	Health Related Quality of Life
IB	Investigator’s Brochure
ICF	Informed Consent Form
ICH	International Conference on Harmonization
IEC	Independent Ethics Committee
IG	Immunogenicity
IN	Investigator Notification
INR	International Normalized Ratio
IL-2	Interleukin-2
IL-6	Interleukin-6
IL-8	Interleukin-8

ILD	Interstitial Lung Disease
irAE	Immune related Adverse Events
IRB	Institutional Review Board
irRECIST	Immune related RECIST
IRT	Interactive Response Technology
IUD	Intrauterine Device
IUS	Intrauterine System
i.v.	intravenous(ly)
IWRS	Interactive Web Response System
LAG525	Lymphocyte Activation Gene525
LDH	Lactate Dehydrogenase
LFT	Liver Function Test
LLN	Lower Limit of Normal
LVEF	Left Ventricular Ejection Fraction
mAB(s)	Monoclonal Antibody(ies)
MAP	Master Analysis Plan
MAPK	Mitogen-Activated Protein Kinase
M-CSF	Macrophage Colony Stimulating Factor
MedDRA	Medical Dictionary for Regulatory Activities
MEK	Mitogen activated Extracellular Signal-regulated Kinase
mmHg	Millimeter Mercury
MTD	Maximum Tolerated Dose
MUGA	Multigated Acquisition
N/A	Not Applicable
NONMEM	Nonlinear Mixed Effects Modeling
NCI	National Cancer Institute
NSCLC	Non-Small Cell Lung Cancer
ORR	Overall Response Rate
OS	Overall Survival
PAS	Pharmacokinetic Analysis Set
PBMC	Peripheral Blood Mononuclear Cells
PE	Pulmonary Embolism
PET	Positron Emission Tomography
p.o.	*per os*/by mouth/orally
PD	Progressive Disease
PD-1	Programmed Cell Death-1
PD-L1	Programmed Cell Death-Ligand 1
pERK	Phosphorylated Extracellular Signal-regulated Kinase
PFS	Progression-Free Survival
P-gp	P-glycoprotein
PHI	Protected Health Information
PK	Pharmacokinetic
PLT	Platelet
PPES	Palmar Plantar Erythrodysesthesia Syndrome
PPS	Per Protocol Set
PR	Partial Response
PRO	Patient Reported Outcome

QD	*quaque die*/once a day
QLQ-C	Quality of Life Questionnaire Core
QoL	Quality of Life
QxW	Every x weeks
RAF	v-raf murine leukemia viral oncogene homolog serine/threonine protein kinase
RBC	Red Blood Cell
RCC	Renal Cell Carcinoma
REB	Research Ethics Board
RECIST	Response Evaluation Criteria in Solid Tumors
RMST	Restricted Mean Survival Time
RoW	Rest of World
RP2D	Recommended Phase 2 Dose
RP3R	Recommended Phase 3 Regimen
RPED	Retinal Pigment Epithelial Detachment
RSI	Reference Safety Information
R Value	ALT/ALP in multiples of ULN
RVO	Retinal Vein Occlusion
SAE	Serious Adverse Event
SAP	Statistical Analysis Plan
SBP	Systolic Blood Pressure
SEB	Staphylococcal Enterotoxin B
SC	Steering Committee
SD	Stable Disease
SLN	Sentinel Lymph Node
SOP	Standard Operating Procedure
SSD	Study Specific Document
SRS	Stereotactic RadioSurgery
SUSARs	Suspected Unexpected Serious Adverse Reactions
T1/2	Half-life
TBIL	Total Bilirubin
TCR	T Cell Receptor
TdP	Torsades de Pointe
TIL	Tumor Infiltrating Lymphocyte
tmax	Time to reach Cmax
TMB	Tumor Mutation Burden
Tregs	Regulatory T Cells
T-VEC	Talimogene laherparepvec
ULN	Upper Limit of Normal
Vd	Volume of Distribution
WBC	White Blood Cell
WT	Wild Type

Glossary of terms

Assessment	A procedure used to generate data required by the study
Biologic Samples	A biological specimen including, for example, blood (plasma, serum), saliva, tissue, urine, stool, etc. taken from a study subject
Cohort	A group of newly enrolled patients treated at a specific dose and regimen (i.e. treatment group) at the same time
Cycles	Number and timing or recommended repetitions of therapy are usually expressed as number of days (e.g., q28 days)
Dose level	The dose of drug given to the patient (total daily or weekly etc.)
Enrollment	Point/time of patient entry into the study; the point at which informed consent must be obtained (i.e. prior to starting any of the procedures described in the protocol)
Investigational drug	The study treatment whose properties are being tested in the study; this definition is consistent with US CFR 21 Section 312.3 and is synonymous with “investigational new drug.”
Investigational treatment	Drug whose properties are being tested in the study as well as their associated placebo and active treatment controls (when applicable). This also includes approved drugs used outside of their indication/approved dosage, or that are tested in a fixed combination. Investigational treatment generally does not include other study treatments administered as concomitant background therapy required or allowed by the protocol when used within approved indication/dosage
Medication number	A unique identifier on the label of each study treatment package which is linked to one of the treatment groups of a study
Subject Number	A unique identifying number assigned to each patient/subject/healthy volunteer who enrolls in the study
Period	A subdivision of the study timeline; divides stages into smaller functional segments such as screening, baseline, titration, washout, etc.
Personal data	Subject information collected by the Investigator that is transferred to Novartis for the purpose of the clinical trial. This data includes patient identifier information, study information and biological samples.
Randomization number	A unique treatment identification code assigned to each randomized patient, corresponding to a specific treatment arm assignment
Stage related to study timeline	A major subdivision of the study timeline; begins and ends with major study milestones such as enrollment, randomization, completion of treatment, etc.
Stage in cancer	The extent of a cancer in the body. Staging is usually based on the size of the tumor, whether lymph nodes contain cancer, and whether the cancer has spread from the original site to other parts of the body
Stop study participation	Point/time at which the patient came in for a final evaluation visit or when study treatment was discontinued whichever is later
Study treatment	Includes any drug or combination of drugs in any study arm administered to the patient (subject) as part of the required study procedures, including placebo and active drug run-ins.<br>In specific examples, it is important to judge investigational treatment component relationship relative to a study treatment combination; study treatment in this case refers to the investigational and non-investigational treatments in combination.
Study treatment interruption	Includes any delay or withholding of study treatment for any reason as well as an interruption during an infusion of study treatment for any reason
Study treatment discontinuation	Point/time when patient permanently stops taking study treatment for any reason
Treatment group	A treatment group defines the dose and regimen or the combination, and may consist of 1 or more cohorts. Cohorts are not expanded, new cohorts are enrolled.
Variable	Identifier used in the data analysis; derived directly or indirectly from data collected using specified assessments at specified time points

Withdrawal of study consent	Withdrawal of consent from the study occurs only when a subject does not want to participate in the study any longer, and does not allow any further collection of personal data

Protocol summary

**Title**	A randomized, double-blind, placebo-controlled, phase III study comparing the combination of PDR001, dabrafenib and trametinib versus placebo, dabrafenib and trametinib in previously untreated patients with unresectable or metastatic *BRAF* V600 mutant melanoma
**Brief title**	Study of safety and efficacy of PDR001 in combination with dabrafenib and trametinib in patients with advanced melanoma
**Sponsor and Clinical Phase**	Novartis, Phase III
**Investigation type**	Drug
**Study type**	Interventional
**Purpose and rationale**	• The purpose of this study is to evaluate safety and efficacy of the combination of an anti-PD-1 antibody (PDR001), a *BRAF* inhibitor (dabrafenib) and a MEK inhibitor (trametinib) in patients with *BRAF* V600 mutant, unresectable and metastatic melanoma.<br>• A safety run-in part will be conducted to determine the recommended phase 3 regimen (RP3R) of PDR001 in combination with dabrafenib and trametinib. The randomized, placebo-controlled part of the study will compare the efficacy and safety of this triplet combination to placebo in combination with dabrafenib and trametinib in previously untreated patients with unresectable or metastatic *BRAF* V600 mutant melanoma.<br>• A separate biomarker cohort will be run to explore changes in the immune microenvironment and biomarker modulations upon treatment with the combination of dabrafenib, trametinib and PDR001.
**Primary Objectives**	**Part 1: Safety run-in**<br>• To determine the recommended regimen of PDR001 in combination with dabrafenib and trametinib for the randomized part (part 3)<br>**Part 2: Biomarker cohort**<br>• To evaluate changes in PD-L1 levels and CD8+ cells upon treatment with PDR001 in combination with dabrafenib and trametinib<br>**Part 3: Double-blind, randomized, placebo-controlled part**<br>• To compare the anti-tumor activity of PDR001 in combination with dabrafenib and trametinib versus placebo plus dabrafenib and trametinib as measured by PFS per investigator's assessment according to Response Evaluation Criteria in Solid Tumors (RECIST) 1.1.
**Key Secondary Objectives**	**Part 3: Double-blind, randomized, placebo-controlled part**<br>• To compare overall survival of PDR001 in combination with dabrafenib and trametinib versus placebo plus dabrafenib and trametinib
**Other Secondary Objectives**	**Parts 1 & 2: Safety run-in and biomarker cohort**<br>• To determine safety and tolerability of PDR001 in combination with dabrafenib and trametinib<br>• To evaluate preliminary anti-tumor activity of PDR001 in combination with dabrafenib and trametinib<br>• To characterize pharmacokinetics (PK) of PDR001, dabrafenib and trametinib when administered in combination<br>• To evaluate the prevalence and incidence of immunogenicity<br>**Part 3: Double-blind, randomized, placebo-controlled part**<br>• To compare the anti-tumor activity of PDR001 in combination with dabrafenib and trametinib versus placebo plus dabrafenib and trametinib, as measured by ORR, DCR, DOR per investigator's assessment according to RECIST 1.1<br>• To evaluate safety and tolerability of PDR001 in combination with dabrafenib and trametinib versus placebo plus dabrafenib and trametinib<br>• To evaluate patient reported outcomes of PDR001 in combination with dabrafenib and trametinib versus placebo plus dabrafenib and trametinib

	• To characterize pharmacokinetics (PK) of PDR001, dabrafenib and trametinib when administered in combination To evaluate the prevalence and incidence of immunogenicity<br>• To characterize the potential for PD-L1 expression to identify subjects with an enhanced response to PDR001 in combination with dabrafenib and trametinib versus placebo plus dabrafenib and trametinib
**Study design**	• This study has been designed as a Phase III trial.<br>• This study begins with an open-label safety run-in part and a biomarker cohort, followed by a double-blind, randomized, placebo-controlled Phase III part.
**Population**	**Part 1: Safety run-in**<br>Adult subjects with unresectable or metastatic melanoma with *BRAF* V600 mutation and ECOG performance status ≤ 1<br>**Part 2: Biomarker cohort**<br>Adult subjects with unresectable or metastatic melanoma with *BRAF* V600 mutation and ECOG performance status ≤ 2<br>**Part 3: Double-blind, randomized, placebo-controlled part**<br>Adult subjects with unresectable or metastatic melanoma with *BRAF* V600 mutation and ECOG performance status ≤ 2
**Key Inclusion criteria**	**Part 1: Safety run-in**<br>• Histologically confirmed, unresectable or metastatic melanoma with *BRAF* V600 mutation<br>• Aspartate transaminase (AST) < 2.5× ULN and Alanine transaminase (ALT) < 2.5× ULN<br>• Measurable disease according to RECIST 1.1<br>• ECOG performance status ≤ 1<br>**Part 2: Biomarker cohort**<br>• Histologically confirmed, unresectable or metastatic melanoma with *BRAF* V600 mutation<br>• At least two cutaneous or subcutaneous lesions or nodal lesions for tumor sample collection<br>• Measurable disease according to RECIST 1.1<br>• ECOG performance status ≤ 2<br>**Part 3: Double-blind, randomized, placebo-controlled part**<br>• Histologically confirmed, unresectable or metastatic melanoma with *BRAF* V600 mutation<br>• ECOG performance status ≤ 2<br>• Measurable disease according to RECIST 1.1

**Key Exclusion criteria**	**Part 1: Safety run-in**<br>• Any history of CNS metastases<br>• Subjects with uveal or mucosal melanoma<br>• Prior systemic anticancer therapy for unresectable or metastatic melanoma<br>• Neoadjuvant and/or adjuvant therapy for melanoma completed less than 6 months prior to enrollment<br>• Radiotherapy within 4 weeks prior to the first dose of study treatment<br>• Active autoimmune disease, and/or history of autoimmune disease(s) that required treatment<br>**Parts 2 & 3 : Biomarker cohort & double-blind, randomized, placebo-controlled part**<br>• Subjects with uveal or mucosal melanoma<br>• Prior systemic anticancer therapy for unresectable or metastatic melanoma<br>• Neoadjuvant and/or adjuvant therapy for melanoma completed less than 6 months prior to enrollment<br>• Radiotherapy within 4 weeks prior to the first dose of study treatment<br>• Clinically active cerebral melanoma metastasis.<br>• Active autoimmune disease, and/or history of autoimmune disease(s) that required treatment
**Investigational and reference therapy**	• PDR001 in combination with dabrafenib and trametinib or placebo in combination with dabrafenib and trametinib
**Efficacy assessments**	• Tumor assessment by investigator's assessment per RECIST 1.1, measured at Cycle 4 Day 1 (± 7 days), then every 8 weeks, until Cycle 22 Day 1 (± 7 days) when frequency will switch to every 12 weeks
**Safety assessments**	• Physical examination<br>• ECOG PS<br>• Weight and vital signs<br>• Ophthalmic examination<br>• 12-lead ECGs<br>• ECHO/MUGA<br>• Laboratory assessments, including hematology, chemistry, thyroid function, coagulation, urinalysis, and cytokine analysis<br>• Monthly pregnancy testing for women of child-bearing potential<br>• Adverse events (AEs), the severity, the relationship to study treatment and the seriousness
**Other assessments**	• PK parameters such as but not limited to Ctrough and $Ctrough_{ss}$ for PDR001, dabrafenib and trametinib.<br>• Exploratory biomarker assessment with tissue samples, germline DNA, circulating tumor DNA (ctDNA), circulating tumor RNA (ctRNA), and peripheral blood mononuclear cells (PBMC).<br>• Patient reported outcomes (PRO) assessment by the European Organization for Research and Treatment of Cancer quality of life (EORTC QLQ-C30), EuroQoL (EQ-5D) questionnaire, and Melanoma Subscale of the FACT-M

**Data analysis**	**Part 1: Safety run-in**<br>The primary variable is the incidence of dose-limiting toxicities (DLTs) during the first 8 weeks (56 days) of PDR001 in combination with dabrafenib and trametinib. DLTs will be listed, and their incidence summarized by primary system organ class and preferred term and worst grade (CTCAE version 4.03). Listings and summaries will be based on the dose-determining analysis set.<br>The dose determination part of this study will be guided by a Bayesian analysis of DLT data for the first 8 weeks (56 days) that subjects receive the combination of PDR001, dabrafenib, and trametinib. The Bayesian analysis to assess the triple combination will be based on a separate 10-parameter model for each dose regimen that comprises single-agent toxicity parts and interaction parts to describe both two-way and three-way drug safety interactions. Details of the model are given in Section 10.4.2 and Appendix 2.<br>Dosing regimen decisions are guided by the escalation with overdose control principle. A dosing regimen may only be used for newly enrolled subjects if the risk of excessive toxicity at that dosing regimen is less than 25%. The starting dosing regimen is 400 mg i.v. Q4W PDR001, 150 mg BID dabrafenib, and 2 mg QD trametinib. For this dosing regimen, the prior risk of excessive toxicity is 12%, which satisfies the EWOC criterion. A full assessment of the prior risk to patients is given in Appendix 2.<br>**Part 2: Biomarker cohort**<br>Descriptive statistics of the primary endpoint, PD-L1 levels and CD8+ cells and changes from baseline, will be summarized by visit.<br>**Part 3: Double-blind, randomized, placebo-controlled part**<br>The primary endpoint of Progression-Free Survival (PFS), based on local (investigator) assessment of RECIST 1.1 criteria, is defined as the time from the date of randomization to the date of the first documented progression or death due to any cause.<br>The key secondary endpoint of Overall Survival (OS) is defined as the time from the date of randomization to the date of death due to any cause.<br>The following statistical hypotheses will be tested to address the primary efficacy objective for PFS:<br>$H_{01} : \theta_1 \geq 1$ vs. $H_{A1} : \theta_1 < 1$<br>where θ_1 is the PFS hazard ratio (HR) (PDR001 combined with dabrafenib and trametinib versus dabrafenib and trametinib). The primary efficacy analysis to test these hypotheses and compare the two treatment groups will consist of a stratified log-rank test at an overall one-sided 2.5% level of significance. The stratification will be based on the randomization stratification factors, i.e., (LDH level: < 1 x ULN vs ≥ 1 to < 2 x ULN vs ≥ 2 x ULN; ECOG PS: 0 vs 1 vs 2). Analyses will be based on the FAS population according to the randomized treatment group and strata assigned at randomization. The PFS distribution will be estimated using the Kaplan-Meier method, and Kaplan-Meier curves, quartiles and associated 95% confidence intervals (CI) will be presented for each treatment group. The hazard ratio for PFS will be calculated, along with its 95% confidence interval, from a stratified Cox model using the same stratification factors as for the log-rank test.<br>The PFS analysis will be performed as a part of a two-look group sequential design using a Gamma alpha spending function to control the type 1 error probability with Gamma parameter = -9.7 (Hwang et al, 1990).<br>A similar hypothesis and model will be used to address the key secondary efficacy objective for OS. The OS analysis will be performed as part of a three-look group sequential design. At the time of the interim and final PFS analysis, interim OS analyses for a possible early significance claim for efficacy will be performed. The type I error probability for the interim and final OS analyses will be controlled by using a Lan-DeMets (O'Brien-Fleming) alpha spending function. A hierarchical testing strategy will be used to control the overall type I error rate, where OS will only be formally tested and interpreted if the primary analysis of PFS is statistically significant.<br>Refer to Section 10.4.3 and Section 10.4.4 for details on censoring, as well as supportive and sensitivity analyses that will be performed for the primary and key secondary efficacy variables.
**Key words**	PDR001, dabrafenib, trametinib, melanoma, immunotherapy, combination treatment

Amendment 5 (08-Mar-2019)

In Part 1 (safety run-in), all 9 patients completed the 8 weeks dose limiting toxicity period and in Part 2 (biomarker cohort), 27 patients were enrolled and have received at least one dose of PDR001, dabrafenib, and trametinib. Among 532 patients randomized in Part 3 (randomized, placebo-controlled part), 531 patients have received at least 1 dose of PDR001/placebo, dabrafenib, and trametinib, and 1 patient died before receiving any study treatment. The last randomized patient's first visit occured on 8 June 2018 and the last randomized patient's first treatment on 11 July 2018.

Amendment rationale

The primary objective of this protocol amendment is to adjust the timing of the final PFS analysis and to include an interim anaysis for PFS based on revised assumptions on the delayed treatment effect. The original trial design of COMBI-i (CPDR001F2301) assumed a delayed treatment effect of 1.5 months for PFS and of 3 months for OS. However, a delayed treatment effect of 5 months for PFS was observed in the KEYNOTE-022 trial in a similar patient population and treatment combination as the COMBI-i (CPDR001F2301) trial, as recently presented at the European Society of Medical Oncology 2018 conference (Ascierto et al., 2018a). KEYNOTE-022 is a Phase II randomized study with pembrolizumab or placebo in combination with dabrafenib and trametinib in patients with untreated BRAF V600 mutant melanoma. Results from KEYNOTE-022 Part 3 (Ascierto et al., 2018b) suggest a 5 months delayed treatment effect for PFS in the investigational arm compared to the control arm. This delayed treatment effect of pembrolizumab is suggestive of unique immune therapy response kinetics, which manifest as a slow change in tumor response over time (Wolchok et al., 2009). Such response kinetics have already been observed with other checkpoint inhibitors in unresectable or metastatic melanoma and other tumor types (Ascierto et al. (2018b); Hoos et al. (2010); Wolchok et al. (2017)). Available data suggest that the delayed treatment effect can be seen from 1.5-6 months for PFS and 3-9 months for OS, based on the tumor type and combination used (Ascierto et al. (2018b); Hoos et al. (2010); Wolchok et al. (2017).

Should a delayed treatment effect of this magnitude be found in the COMBI-i (CPDR001F2301) study it will result in a substantial loss of statistical power for the PFS analysis (refer to Section 10.8 for statistical analysis and assumptions); therefore the study design is revised to address this eventuality. In order to achieve a statistical power of 80% based on a conservative assumption of a 5 months delayed treatment effect and followed by an effect of the same magnitude as assumed in the original protocol (i.e. HR=0.60), the number of PFS events for the final PFS analysis is increased from 246 to approximately 352 PFS events. Furthermore, an interim PFS analysis is being introduced at approximately 260 PFS events for the reasons described below.

Since the KEYNOTE-022 study is a small Phase II study with a total of 120 patients, there is still some uncertainty over the exact duration of the delayed effect. The introduction of an interim PFS analysis provides an opportunity to detect a compelling clinical benefit at an earlier timepoint if the delayed effect is in fact less than 5 months and/or there is a very high treatment effect following the no-effect period. The timing and significance boundary of this interim PFS analysis has been chosen so that the efficacy threshold is met only when the magnitude of the PFS treatment effect is sufficient and clinically relevant (i.e. using a stringent significance level

at interim PFS analysis with a small penalty for the final PFS analysis). As mentioned above, the final PFS analysis, performed at approximately 352 PFS events, will provide an 80% cumulative power to detect a statistically significant treatment effect if the delayed treatment effect is indeed 5 months long as observed in KEYNOTE-022 and the subsequent effect is of the same magnitude as currently assumed (i.e. HR=0.60).

Since the total of 352 PFS events targeted for the final PFS analysis represent a large percentage of the patients randomized in the study (66.2%) the rate of events is likely to decrease with longer follow-up and formation of a plateau in the PFS Kaplan-Meier curve might occur in the combination arm with PDR001 based on response patterns seen in studies with other checkpoint inhibitors (Robert et al., 2015; Schachter et al., 2017), hence achieving the target number of events for the final PFS analysis might be challenging to obtain within a reasonable period of follow-up. Therefore, the actual data cutoff date for the final PFS analysis will occur when the target number of approximately 352 PFS events has been observed or approximately 24 months after the last patient has been randomized, whichever occurs first. Furthermore, the 24 months cut off date allows for sufficient mature 24 months follow up for the landmark PFS as well as OS Kaplan-Meier estimates.

Additionally, the OS analysis is also revised based on an assumed 5 months delayed treatment effect (changed from 3 months delayed effect for the original study design). Although there is now provision to additionally test for OS at the interim PFS analysis with a proper control of the type I error rate it is acknowledged that the power of this first interim OS analysis will be limited.

The Data Monitoring Committee (DMC) will be responsible for reviewing the efficacy results from the interim PFS analysis, which will be performed by an independent external statistician. If the efficacy threshold has been crossed at the time of the interim PFS analysis, the DMC may recommend to consider making the unblinded study results available to Novartis. However, the individual patient blind should still be maintained for investigators and patients at least until the final OS analysis (or a significant interim OS result).

This protocol amendment will also incorporate the following changes:

- Clarification of the PRO administration and review requirements
- Clarification on the timing of the required imaging assessments during efficacy follow-up
- Clarification for patients to resume treatment in case of asymptomatic amylase and/or lipase elevation grade 4 once AE is resolved to baseline or grade ≤ 1 within 4 weeks. Asymptomatic elevation of amylase and lipase are observed in approximately 14 % of patients treated with checkpoint inhibitors. Discontinuing treatment based on the mere elevation of lipase and amylase in the absence of pancreatitis carries the risk of unnecessarily terminating treatment prematurely for patients (Brahmer et al., 2018; Puzanov et al., 2017).

Changes to the protocol

- Protocol summary and Sections 2.2, 4.1.2, 4.2, 4.3, 7.1.3, and 10: Revised number of PFS events for the final PFS analysis, included an interim PFS analysis, and modified OS analyses.

- Section 6.3, Table 6-8: Added asymptomatic amylase and/or lipase elevation to the list of AEs
- Section 6.3.3, table 6-18: Modified for cases with asymptomatic grade 4 elevation of amylase and/or lipase.
- Section 6.3.3.12, Table 6-19: Moved footnote text 'for asymptomatic amylase and/or lipase elevations refer to Table 6-18' into the table heading
- Section 7.1, Tables 7-1 through 7-5: Clarified that imaging assessments during efficacy follow-up should be performed every 12 weeks starting from the last assessment.
- Section 7.1, Tables 7-3: Corrected error in visit evaluation schedule for Part 3 only, where the urinalysis assessment (microscopy and macroscopic) was categorized incorrectly.
- Section 7.1, Tables 7-3 and 7-4, and Section 7.2.5: Changed the requirement for the investigator to review the PRO for responses which may indicate potential AEs or Serious Adverse Events (SAEs) before the clinical examination to a recommendation. This change is made to allow sites more flexibility given the cumbersome and impractical nature of these requirements.
- Section 7.2.1: Included central review of imaging data using RECIST 1.1 and tumor response criteria based on guidelines for immunotherapy at the time of the added interim PFS analysis and clarified that this is not needed at the time of the final OS analysis.
- Section 8.3: Clarified that emergency unblinding decisions must only be undertaken by the investigator. The actual unblinding procedure in the Interactive Web Response System (IWRS) can be performed either by the investigator or a designee.
- Sections 8.6 and 10.7.1: Added the review of efficacy results by the DMC.

IRBs/IECs

A copy of this amended protocol will be sent to the Institutional Review Board (IRBs)/Independent Ethics Committee (IECs) and Health Authorities.

The changes described in this amended protocol require IRB/IEC approval prior to implementation.

The changes herein affect the Informed Consent. Sites are required to update and submit for approval a revised Informed Consent that takes into account the changes described in this protocol amendment.

Amendment 4 (14-Aug-2018)

In Part 1 (safety run-in part) and Part 2 (biomarker cohort) of the study, 36 patients have been enrolled and received at least 1 dose of PDR001, dabrafenib, and trametinib. In Part 3 (randomized part), 532 patients have received at least 1 dose of PDR001/placebo, dabrafenib, and trametinib. The last patient's first visit (i.e., date Main Study Informed Consent Form (ICF) signed) was completed on 08 June 2018.

Amendment rationale

The primary objective of this protocol amendment is to align the contraception requirements during and after study treatment based on the dabrafenib Investigator's Brochure (IB) Edition 10 and trametinib IB Edition 9. Further, the safety follow-up periods have been aligned with the contraception requirements after study treatment has been discontinued.

Further, this protocol amendment will incorporate the following:

- Revised individual subject unblinding requirements to limit the impact of unblinding on the scientific validity of the study results. Except for medical emergencies, for regulatory reporting purposes, or if it is critical to determine subsequent therapy after disease progression, documented approval by the Novartis study physician is required prior to unblinding a subject's treatment assignment.
- Corrected language that if a subject is unblinded, he/she must be discontinued from the study treatment and must be followed for efficacy and survival, if applicable.
- Added a new exploratory objective and a corresponding endpoint to characterize the potential for tumor mutation burden (TMB) alone and in combination with PD-L1, or additional markers to identify subjects with an enhanced response to PDR001 in combination with dabrafenib and trametinib versus placebo plus dabrafenib and trametinib.
- Updated definition of personal data and withdrawal of consent to reflect the European Economic Area (EEA) General Data Protection Regulation (GDPR) requirements.
- Other minor corrections are also applied throughout the protocol.

Changes to the protocol (in order of appearance in the protocol)

Changes to specific sections of the protocol are shown in the track changes using strike through red font for deletions and red underline for insertions.

Major changes

- Changed the 150 day contraception follow up period from "150-days after stopping treatment with PDR001" to "150-days after stopping treatment with PDR001/placebo, 30-days after dabrafenib, or 120-days after trametinib, whichever is longer" in exclusion criterion 28 and 29 (Section 5.3). No new safety concerns have been observed in the program and/or trial. However, to align the contraception requirements during and after dabrafenib and trametinib based on dabrafenib Investigator's Brochure edition 10 and trametinib Investigator's Brochure edition 9 the protocol has been updated.
- Added a new exploratory objective "To characterize the potential for tumor mutation burden (TMB) alone and in combination with PD-L1, or additional markers to identify subjects with an enhanced response to PDR001 in combination with dabrafenib and

trametinib versus placebo plus dabrafenib and trametinib", and an endpoint "PFS by investigator's assessment according to RECIST 1.1 and OS in subgroups defined by centrally assessed TMB status (determined by Next-Generation Sequencing) alone and in combination with PD-L1, or additional markers" in Section 3. Corresponding changes in Section 7.2.4 were made to align with udpates in Section 3.

- Part 3 Other Secondary Endpoint #6 was clarified and aligned to be consistent with Exploratory Endpoint #5 by adding "in subgroups defined" after OS: "PFS by investigator's assessment according to RECIST 1.1 and OS in subgroups defined by centrally assessed PD-L1 status" in Section 3.
- Changed the start of the 150 day safety follow up period from "the last dose of PDR001" to "the last dose of PDR001/placebo" throughout the document in Sections 6.3.6, 7.1.6, 8.1.1, 8.2.2 and 10.5.3 for the randomized, placebo-controlled part (Part 3) of the study.
- Added requirements for unblinding in Section 6.5.3 and 8.3 to limit the impact of unblinding on the scientific validity of the study results. "Individual patient unblinding will be prohibited, except for medical emergencies, for regulatory reporting purposes, or if it is critical to determine subsequent therapy after disease progression. Except in these cases, documented approval by the Novartis study physician is required prior to unblinding a subject's treatment assignment. In case of unblinding, all data is required to be captured in the eCRF prior to unblinding. Data after unblinding will continue to be collected as per protocol."
- Removed language "The study will be unblinded either at interim or final analysis if statistically significant overall survival results are demonstrated." in Section 6.5.3 for clarity.
- Added "In case where individual drug components of the study treatment are discontinued at different times, subjects must be followed for safety evaluation up to 150 days following the last dose of PDR001/placebo or up to 30 days after the last dose of dabrafenib or 120 days after the last dose of trametinib, whichever is longer." in Section 7.1.6 to update and clarify safety follow up periods for each drug components of the study treatment to align with contraception requirements. Subsequently, revised the AE monitoring and Serious Adverse Event (SAE) reporting period in Sections 8.1.1 and 8.2.2, respectively, to align with Section 7.1.6.
- Added language that requires subjects to be followed for skin related toxicity every month for 6 months from the last dose of dabrafenib in Section 7.1.6 to align with Section 6.3.5.3.
- Added language in Section 8.3 to ensure continued efficacy and survival follow up for unblinded patients: "If a subject is unblinded, he/she must be discontinued from the study treatment and must be followed for efficacy and survival, as applicable per Section 7.1.7 and 7.1.8."

Minor changes

- Removed "taking medicinal products known to prolong the QT interval" from exclusion criterion 23 (Section 5.3) as dedicated QT studies in humans or analysis of phase I study ECG data showed no clinically relevant effect of dabrafenib, metabolites of dabrafenib, trametinib or PDR001 to alter the QTc interval.

- Removed “dietary” supplements from the list of products that are not permitted for use during dabrafenib and trametinib dosing in Section 6.1.1.5. Based on the available data, there is low risk of interaction between dietary supplements and dabrafenib or trametinib PK.
- Corrected an error in Table 6-10 in Section 6.3.3 Dose modification guidance for Grade 3 or 4 AST or ALT increase for the 1st and 2nd occurrence “Interrupt dabrafenib and trametinib until recovery to ≤ Grade ~~2~~1 or baseline.”
- Modified section titles and table titles in Sections 6.3.3.10 and 6.3.3.11 to indicate that Table 6-17 provides dose modification guidelines for neutropenia and thrombocytopenia and Table 6-18 for AEs including laboratory abnormality related AEs. Corresponding changes were made in Tables 6-8, 6-17 and 6-18.
- Added a footnote for pancreatitis under Table 6-19 in Section 6.3.3.12 that “For asymptomatic amylase and/or lipase elevation, please refer to Table 6-18” to clarify dose modification guideline for asymptomatic amylase and/or lipase elevation.
- Added that limited-field palliative radiotherapy to non-target lesion(s) may be allowed “after documented discussion with Novartis study physician” as concomitant therapy and in case of palliative radiotherapy, the subject should interrupt dabrafenib and trametinib for stereotactic and fractionated radiotherapy (i.e., ≥ 3 days) and stereotactic radiosurgery (i.e., ≥1 day) before and after radiotherapy in Section 6.4.1 per publication indicating that BRAF inhibitors increase the risk of dermatologic toxicity when given concurrently with radiation treatment (Anker CJ et al, 2016).
- Added “(recurrent) pyrexia” to the conditions for which systemic steroid therapy and other immunosuppressive drugs are allowed in Section 6.4.3.1.
- Corrected the discrepancies regarding the permitted dose of steroids during study treatment from < 10 mg/day to ≤ 10 mg/day in Section 6.4.3.
- Information regarding follow-up for new cutaneous malignancies was added to Visit Evaluation Schedule Tables 7-1 to 7-4 to be consistent with the required safety follow-up specified in protocol Section 6.3.5.3. Subsequently, added short physical examination to be performed every month for 6 months from the last dose of dabrafenib or until initiation of another antineoplastic therapy in Tables 7-1 to 7- 4 to align with Section 6.3.5.3.
- Added language to clarify that short physical examination at 30 day safety follow up period is based on the last dose of PDR001/placebo in Tables 7-1 to 7-4 in Section 7.1.
- Updated definition of Personal Data and withdrawal of consent in Glossary of terms and Section 7.1.5 to reflect the European Economic Area (EEA) General Data Protection Regulation (GDPR) requirements.
- Added a footnote to clarify the PDR001/placebo PK and IG sample collection at 30 day and 150 day safety follow up visits in Tables 7-10 to 7-12: “If PDR001/placebo is permanently discontinued prior to dabrafenib/trametinib, 30 day and 150 day safety follow up visits should be based on the last dose of PDR001/placebo” to ensure post treatment PK & IG samples are collected based on the last dose of PDR001/placebo.
- Clarified that CNS and bone are not only excluded organs for biopsy, but also excluded for biomarker analyses even if archival samples are available (Section 7.2.4).

- Corrected the time point of blood collection for germline in DNA Section 7.2.4.2.1 from "any time during screening prior to study treatment" to "anytime" and corresponding changes were made in Tables 7-1 to 7-3 and Tables 7-16 to 7-17.
- New references cited under Amendment 04 have been added to Section 13.

IRBs/IECs

A copy of this amended protocol will be sent to the Institutional Review Board (IRBs)/Independent Ethics Committee (IECs) and Health Authorities.

The changes described in this amended protocol require IRB/IEC approval prior to implementation.

The changes herein affect the Informed Consent. Sites are required to update and submit for approval a revised Informed Consent that takes into account the changes described in this protocol amendment.

Amendment 3 (14-Jul-2017)

The first patient's first visit (i.e., date Main Study ICF signed) was completed on 17 February 2017. Subsequently, the first treatment of a patient occurred on 07 March 2017. As of 17 July 2017, 12 patients have received at least 1 dose of PDR001, dabrafenib, and trametinib in Part 1 and Part 2 of the study.

Amendment rationale

The primary objective of this protocol amendment is to change pyrexia management guidelines based on the safety profile observed in the safety run-in (part 1) and feedback received from investigators upon review of safety data.

At the same time, changes to improve data collection for radiotherapy events, central review using tumor response criteria based on guidelines for immunotherapy, ophthalmologic examination assessment frequency, and patient reported outcomes are implemented. In addition, the *BRAF* V600 testing method was clarified and language was added to reflect the AJCC edition 8 melanoma staging system. Further, the frequency the data monitoring committee will review safety data was revised from 6 to 3-6 months to ensure appropriate safety monitoring. Other minor corrections are also applied throughout the protocol.

Changes to the protocol

Changes to specific sections of the protocol are shown in the track changes version of the protocol using strike through red font for deletions and red underline for insertions.

- 'Absence of irPD' in Section 6.1.5.1 was removed, given local assessment based on response criteria for immunotherapy is not required in this study. Subsequently, the text was adjusted in Sections 2.2, 4.1, 7.2.4.1; Figure 4-3; Tables 7-1, 7-2, 7-3, 7-4, 7-16, 7-17 to reflect this change.
- To allow further central analysis using tumor response criteria based on guidelines for immunotherapy by the blinded independent radiology committee, at least two additional tumor assessments that are at least 4 weeks apart should be performed no less than 4 weeks after the criteria for PD are first met. Additionally, clarification was added to require an additional tumor assessment to be performed to confirm response (CR or PR) to be consistent with Section 7.2.1.2. (Sections 4.1.5, 6.1.5.1, 7.1.7, 7.2.1.2 and Tables 7-1, 7-2, 7-3, 7-4).
- Clarified definition of end of study (Section 4.3) in the case that statistically significant interim (at the time of the primary PFS analysis) or final OS results are demonstrated.
- Changed dose modification guidelines for pyrexia syndrome to reflect that dabrafenib and trametinib must be interrupted promptly at the very first symptom of pyrexia or its associated prodrome and should be restarted at the same dose if symptom free at least 24 hours (Section 6.3.5.4).
- Clarified dose modification guidelines for abnormal liver enzyme test to reflect that PDR001/placebo must be permanently discontinued if abnormal liver enzyme functions are considered to be related to study treatment by the investigator (Table 6-10).
- Changed the reporting of concurrent radiotherapies during study treatment from 'Surgical and Medical Procedures' to 'Concomitant Antineoplastic Therapy – Radiotherapy'

electronic Case Report Form (eCRF) in order to capture all required data fields for analysis (Sections 6.4.1, 6.4.4).

- Removed statement alluding to “If the dose of prednisone or equivalent cannot be reduced to less than 10 mg/day before the administration of next dose of study treatment then PDR001/placebo must be discontinued” (Section 6.4.3.1) as this language is not applicable to this study.
- Decreased the ophthalmologic examination assessment frequency (Tables 7-1, 7-2, 7-3, 7-4) in order to align with clinical practice and label. In addition, to reflect clinical practice, clarified that dilation of pupils is only required if clinically indicated (e.g., changes in visual acuity), and optical coherence tomography is recommended at scheduled visits, and mandated if retinal abnormalities are suspected (Section 7.2.2.5).
- Added more specific imaging-related instructions to visit evaluation schedules (Tables 7-1, 7-2, 7-3, 7-4) in alignment with Section 7.1.4, Table 7-5, and Section 7.2.1.2 for clarity. Further, updated Section 4.1.2 to be consistent with Section 7.1.4.
- Revised patient reported outcome schedule of assessments to align with imaging assessments (Tables 7-1, 7-2, 7-3, 7-4).
- Added that needle aspiration, biopsy or any other form of collection (e.g., paracentesis, punctuation) performed for any reason must be collected to capture all data required data fields for analysis (Tables 7-1, 7-2, 7-3, 7-4).
- Added clarification for the timing of when PRO assessments should be completed to align with Section 7.2.5 (Tables 7-3, 7-4).
- Clarified that a validated tissue-based molecular test is required to determine the *BRAF* V600 mutation status. Immunohistochemistry is not an accepted method and liquid biopsy based *BRAF* V600 results cannot be used to enroll patients. Further, clarified that if the local lab uses a non-FDA approved BRAF V600 assay, this information must be provided to the Sponsor or representative for review. (Section 7.1.2).
- Added language in Section 7.1.2.3 to allow data collection of newly published AJCC edition 8 melanoma staging (published Q4/2016). In addition, the AJCC edition 8 staging parameters were added as an appendix (Section 14.4).
- Added that tumor and PRO assessments performed as part of End of Treatment (EOT) in part 3 will not be required to be repeated if performed within 30 days prior to the first dose of study treatment in crossover part (Section 7.1.3; Tables 7-4, 7-5).
- Revised central review of imaging data from irRECIST criteria to tumor response criteria based on guidelines for immunotherapy (e.g., Seymour 2017) (Sections 7.2.1, 10.4.1, 10.4.4, 10.5.2).
- Revised data transfer requirements for blinded independent radiology committee review (Section 7.2.1).
- Removed language to be consistent with mandatory brain imaging requirement at screening (Section 7.2.1.1).
- Clarified in footnote that PK and immunogenicity samples for PDR001 will not be collected at Cycle 1 Day 8 for Part 2 (Table 7-10).
- Added language to allow request for additional archival tumor tissue in case the sample provided is insufficient for the proposed biomarker analyses (Section 7.2.4.1).

- Clarified that all blood samples collected on the study for biomarker analysis may be interchangeably used for analyzing ctDNA, ctRNA, cytokines and other circulating markers of interest to address scientific questions related to PDR001, melanoma, treatment response or clinical outcome (Section 7.2.4.2).
- Revised the frequency the data monitoring committee (DMC) will review safety data from approximately 6 to 3-6 months in order to ensure appropriate safety monitoring (Section 8.6).
- Added that PD-L1 level cut-off value of 5% will also be part of an efficacy subgroup analysis (in addition to the analysis using a 1% and 10% cut-off) (Section 10.5.2).
- Corrected evaluability criteria for biomarker cohort patients in Part 2 (Section 10.8).

IRBs/IECs

A copy of this amended protocol will be sent to the Institutional Review Board (IRBs)/Independent Ethics Committee (IECs) and Health Authorities.

The changes described in this amended protocol require IRB/IEC approval prior to implementation.

The changes herein affect the Informed Consent. Sites are required to update and submit for approval a revised Informed Consent that takes into account the changes described in this protocol amendment.

Summary of previous amendments

Amendment 2 (07-Mar-2017)

As of 07-Mar-2017, 3 patients have signed Main ICF, and 1 patient has received study treatment in the study. The original protocol has been initiated at 2 study centers globally.

Amendment rationale

Amendment 2 (substantial amendment) is required to implement specific feedback received from the U.S. Food and Drug Administration (FDA) and the German Paul-Ehrlich Institute (PEI) and the French health authority ANSM (agence nationale de sécurité du medicament) upon review of the protocol. In addition, clarifications and corrections are made throughout the protocol next to editorial change to improve flow and consistency.

Changes to the protocol

Changes to specific sections of the protocol are shown in the track changes version of the protocol using strike through red font for deletions and red underline for insertions.

Major changes

- Updated criteria for dose-limiting toxicities (DLTs) for part 1 (Table 6-6):
    - Added ‘Grade 4 anemia’, ‘Grade 4 hypertension of any duration’ and ‘≥ Grade 3 infusion related reaction’. Changes mandated by FDA.

    - Subsequently, 'Severe infusion reactions' were deleted from 'Events which will NOT be considered as DLT', and in addition, the following language was added in Section 6.1.1.5: 'For part 1, on days of coadministration with PDR001, dabrafenib and trametinib should be taken approximately 90 minutes after completion of PDR001 administration.'
  - Added '≥ Grade 3 AEs that are known to occur with dabrafenib, trametinib and/or PDR001, but cannot be controlled using the recommended product-specific management guidelines or lead to <50% of planned exposure to study medications'. Change mandated by FDA.
  - Added window for correction of ≥ Grade 3 electrolyte abnormalities to specify the exception from the definition of DLT: 'For laboratory values ≥ Grade 3, the maximum allowable time limit for correction of electrolyte abnormalities to ≤ Grade 1 is 72 hr'. Change mandated by FDA.
  - Changed threshold for subjects with normal baseline AST and ALT values: 'AST or ALT > 8.0 × ULN'. Change mandated by FDA.
    - Subsequently, 'Grade 4 AST or ALT elevation' was removed.
  - Changed definition for thrombocytopenia to be independent of duration and transfusion requirement: 'Grade 3 thrombocytopenia with clinically significant bleeding regardless of duration or requirement of platelet transfusion'. Change mandated by FDA.
  - Deleted 'or test results (e.g., ECG, ECHO/MUGA)' from 'Events which will NOT be considered as DLT for the purpose of this protocol' as these are covered under cardiac events.
  - Added 'and 2.0 x ULN' for subjects with abnormal baseline AST or ALT or abnormal baseline bilirubin value to align with the drug-induced liver injury (DILI) definition in Section 6.3.6.2.
- Revised exclusion criterion #17 (Section 5.3) to include a time window: 'Active infection requiring systemic antibiotic therapy within 2 weeks prior to start of study treatment'. Change mandated by German Paul-Ehrlich Institute.
- Added requirement for a local HIV testing at screening for subjects in Germany to exclusion criterion #18: 'Known history of testing positive for Human Immunodeficiency Virus (HIV) infection. For Germany only: testing positive for HIV during screening using a local test.' Change mandated by German Paul-Ehrlich Institute. (Sections 5.3, 7.1.2.3, 7.2.2.6, 7.2.2.6.9).
- Updated the primary objective in Table 3-1 for part 2 to specifically mention the main 2 biomarkers of interest. Under 'Objective': 'To evaluate changes in PD-L1 levels and CD8+ cells upon treatment with PDR001 in combination with dabrafenib and trametinib', and under 'Endpoint': 'Descriptive statistics of PD-L1 levels and CD8+ cells and changes from baseline by visit' were added. All other biomarkers will be considered as exploratory objectives. (Table 3-1; Sections 10.4, 10.5).
- Added the same secondary objectives and endpoints specified for part 1 for part 2 in Sections 3 (Table 3-1) and 10.5.2.

- Subsequently, language was updated to mention that supportive analysis using central review of RECIST and irRECIST will also be performed for part 2 (Section 10.5).
- Corrected frequency of ECG assessments for part 1, 2 and 3 to account for results from prospective cardiac repolarization studies with dabrafenib and trametinib (BRF113773, MEK114655), prescribing information and preliminary/expected results for the anti-PD-1 (PDR001): ECGs are now required (1) at screening, (2) Cycle 2 Day 1 at Cmax (1 hour after PDR001 administration), (3) on-treatment as clinically indicated and (4) EOT. Tables 7-1, 7-2, 7-3, 7-4; Section 7.2.2.7.1; Tables 7-8, 7-9 have been updated accordingly. In addition language for triplicate collection and central review has been added.

Minor Changes

- Revised biomarker sample collection plan for part 2 and part 3 to accommodate the different dose levels studied (Section 2.2; Figure 4-2; Tables 7-2, 7-3, 7-16, 7-17; Section 7.2.4.1)
- Added a paragraph to clarify the rationale for testing part 1 cohorts DL-1a and DL-1b in parallel instead of sequentially (Section 2.3). Change suggested by FDA.
- Added rationale for combination dose to Section 2.3 based on clinical, PK, and preliminary data from other studies with dabrafenib and trametinib in combination with other anti-PD-1 or anti-PD-L1 antibodies. Change suggested by German Paul-Ehrlich Institute.
- Clarified that part 1 subjects in the DL-1b cohort, who do not tolerate and discontinue dabrafenib and/or trametinib during the first 4 weeks will be replaced since they would not have received sufficient exposure to the triplet combination therapy (Section 4.1).
- Corrected exclusion criterion 13: ‘within 28 days or 5 half-lives, whichever is longer” to “within 28 days or 5 half-lives, whichever is shorter” (Sections 5.3.3, 6.4, 7.1.2.3), in order to cap the enrollment window at 28 days, considering the broad therapeutic index of dabrafenib and expected positive risk benefit profile in patients with unresectable and metastatic melanoma.
- Corrected ‘lyophilisate in vial for iv infusion’ to ‘powder for solution for infusion’ in Sections 6.1 (Tables 6-1, 6-3, 6-4) and 6.6.1 to harmonize with a standard pharmaceutical term. Change requested by Swedish Medical Products Agency.
- Added instructions (Sections 6.1.1.4., 6.6) to counsel subjects to notify study personnel for suspected infusion reactions: ‘Subjects should be provided instructions to notify study personnel if symptoms of infusion reaction occur after any PDR001 infusion.’ Change mandated by German Paul-Ehrlich Institute.
- Specified dose interruptions for PDR001/placebo include delaying or withholding the treatment for any reason as well as an interruption of treatment during an infusion (Section 6.3.1). Further, removed the criteria that ‘if a subject requires a dose interruption of > 12 weeks from the time the immune-related AE reaches a grade that leads to PDR001/placebo interruption, then the subject must be discontinued from the study’, in order to allow for dabrafenib and/or trametinib to be continued if tolerated.
- Revised general dose modification instructions (Section 6.3.2) and mandatory dose modification and management guideline for pyrexia (Section 6.3.5.4; Table 6-24) suspected to be related to dabrafenib and/or trametinib treatment to withhold trametinib in

addition to dabrafenib when the fever is higher than 40°C or104°F or the fever is complicated by rigors, hypotension, dehydration, or renal failure. Change mandated by FDA. Added ‘Monitor renal function for complicated pyrexia’ in the recommended management guideline for the 1st, 2nd and subsequent occurrences of pyrexia. Removed ‘trametinib may be continued’ in dose modification requirements for the subsequent occurrences of pyrexia.

- Added mandatory dose modification and management guideline for serious skin reactions (Section 6.3.3.4) to include Stevens-Johnson syndrome and toxic epidermal necrolysis. Change mandated by FDA.
- Revised mandatory dose modification and management for visual changes (Section 6.3.5.5; Table 6-25) suspected to be related to dabrafenib and/or trametinib treatment to permanently discontinue dabrafenib for ≥ Grade 2 uveitis (including iritis and iridocyclitis) of > 6 weeks duration. Change mandated by FDA.
- Added mandatory dose modification and management guideline for hemorrhage (Section 6.3.5.7) and thromboembolic events (Section 6.3.5.8) suspected to be related to dabrafenib and/or trametinib treatment. Change mandated by FDA.
- Clarified and better aligned Section 6.4.3.2 with the SPC. Although not contraindicated, certain medications should be used with caution due to potential drug-drug interactions. In addition a cross-reference to Section 6.4.2.2 was added. Change requested by French health authority (AMNS).
- After Cycle 1, changed the window for all assessments from ± 3 days to ± 7 days, to allow more time for adverse event management before skipping of PDR001 dose if required. Subsequently, removed the ± 7 day window of assessment for radiographic assessments and patient reported outcome and during holidays to avoid redundancy (Section 7.1).
- Revised schedule of assessments for ECHO and biomarker sample collection plans (Tables 7-1, 7-2, 7-3, 7-4).
- Specified that the BRAF V600 mutation will be assessed using a validated test and added Biomerieux THxID™-BRAF as an example of FDA approved assay (Section 7.1.2). Change mandated by FDA.
- Implemented mandatory brain imaging (MRI/CT) as part of the screening assessment, and removed ‘as clinically indicated’ (Sections 7.2.1, 7.2.1.1). Change mandated by German Paul-Ehrlich Institute.
- Added an additional optional tumor biopsy collection at Cycle 1 Day 15 for part 3 to expand biomarker analyses with samples collected in part 3 (Tables 7-3; Table 7-16); Section 7.2.4.1)
- Updated adverse events of special interest (AESI) to align with current prescribing information and AEs of other immune checkpoint inhibitors. Removed ‘Deep vein thrombosis/pulmonary embolism’ as AESI for dabrafenib but added to AESI for trametinib. (Section 8.1.3). Change mandated by FDA.
- Clarified ‘Neutropenia (in combination with trametinib)’ is considered AESI for dabrafenib and trametinib. Updated AESI for PDR001 based on most recent safety data: endocrinopathies, colitis, and rash; added infusion reaction and other immune disorders as AESI. (Section 8.1.3)

- Added clarification to allow Novartis drug safety to be unblinded to study treatment per Novartis SOP if a SAE is confirmed as SUSAR (Section 8.2.2). Change mandated by FDA.
- Added language to convene DMC quickly if warranted: 'The DMC will be informed and convened quickly for DMC evaluation and input in the event of unexpected results that raise concerns' (Section 8.6). Change mandated by FDA.
- Specified that PD-L1 expression levels will be determined by immunohistochemistry (Section 10.5.2). Change mandated by FDA.
- Added additional criteria to the dose-determining set (DDS) (Section 10.1.4) to clarify that subjects who do not meet the minimum exposure criterion due to an AE will be counted as having a DLT. Subjects who do not meet the minimum exposure criterion for reasons other than AE's (e.g., rapid disease progression or non-compliance) will not be included in the DDS.
- Added that PD-L1 level cut-off value of 10% will also be part of an efficacy subgroup analysis (in addition to the analysis using a 1% cut-off) (Section 10.5.2).

Other protocol changes:

- Protocol summary: Updated to reflect the major changes throughout the document
- To be consistent with Section 4.1, revised language from 'six to or 18' to 'at least 6 subjects, and up to approximately 18' subjects will be enrolled in part 1 (Section 2.2).
- Aligned rationale for the dose of dabrafenib and trametinib with the label (Section 2.3).
- Added 'rash' as an example of potential irAE of immune checkpoint inhibitors (Section 2.6).
- Added language 'it is possible for additional dosing regimens to be used based on emerging data from part 1, and recruitment may be suspended at any time' in Section 4.1 to align with Sections 2.2 and 4.1.2.
- Added that every effort will be made 'in alignment with local regulations' to continue provision of investigational treatment outside the study to subjects who are deriving clinical benefit (Section 4.3).
- Revised inclusion criterion 13 to remove 'albumin infusion' for consistency (Section 5.2.4).
- Moved exclusion criterion 30 to the notes of exclusion criteria 28 for flow and clarity (Section 5.3).
- The start day of PDR001 for part 1 DL-1b was revised from 'from Day 29' to 'starting at C2D1 (Day 29)' in Table 6-2 to align with Table 6-5.
- Clarified that the study treatment duration presented in Section 6.1.5 applies to all 3 parts of the study.
- Added Q8W regimen for PDR001/placebo as the potential starting dose level to Table 6-7 (Section 6.3).
- Updated the 'Reference of AEs and toxicity management guidelines' and added serious skin reaction, hemorrhage, and thromboembolic events to the guideline to Table 6-8 (Section 6.3) to be consistent with the changes mandated by the FDA.

- Updated the list of AEs that require dose modification for only dabrafenib to specify 'uncomplicated' pyrexia (Section 6.3.2). Change mandated by FDA.
- Added 'mandatory' to the section titles of all dose modifications and recommended clinical management guidelines for AEs (Section 6.3).
- Applied consistent titles to accommodate for casual relationship ('suspected to be related to dabrafenib and trametinib') for mandatory dose modifications for dabrafenib and trametinib (Section 6.3.5).
- Revised permitted concomitant therapies to include RANK-ligand inhibitors (e.g., denosumab) for bone targeted therapies. Clarified that any radiotherapy needs to be listed in the Surgical and Medical Procedures eCRF page. Further, the name of the eCRF page to record concomitant therapy was corrected to be consistent with CRF database. (Section 6.4.1).
- Added cautionary language when co-administration of CYP2C or CYP3A4 substrates with narrow therapeutic index is required, while taking dabrafenib (Section 6.4.2.2).
- As steroids may be used for treatment of pyrexia, added 'pyrexia' to one of the conditions that require tapering of systemic corticosteroids if used and dose should be at non-immunosuppressive doses (< 10 mg/day of prednisone or equivalent) before the next administration of study treatment (Section 6.4.3.1).
- To maintain the scientific integrity of the study, specific guidance was provided to maintain blinding for part 3 (Section 6.5.3):
  - Added exceptions to who may be unblinded to randomization data until the time of unblinding: 'unblinded CRAs, independent biostatistician and programmer who will perform DMC analysis'.
  - Added that the sleeve used to cover the infusion bag will be 'non-translucent' in addition to opaque, to ensure any potential visible differences in treatments will be concealed.
  - Added language to clarify that unblinding will be permitted if information is critical to determine the optimal subsequent treatment for the subject. Further, added language that in case of unblinding for post progression determination to subsequent treatment, all data up to the point of the unblinding is required to be captured in the eCRF. The study will be unblinded either at interim or final analysis if statistically significant overall survival results are demonstrated.
  - Added language to specify that the unblinded pharmacist must not administer the drug to the subject nor have any contact with the study participants.
- Specified that the CRA (part 1 and part 2) and unblinded CRA (part 3) are responsible for drug accountability (Section 6.6.3.2).
- Updated instructions for disposal and destruction of study drug to follow 'local regulations and institutional guidelines' (Section 6.6.4).
- Removed ECHO assessment and ophthalmic examination on C1D1 from Tables 7-1, 7-2, 7-3, 7-4 to avoid repeating assessments performed at Screening.
- Added 'physical examination', in addition to laboratory assessments, as one of the screening evaluation that will not require to be repeated prior to dosing if performed within 7 days prior to the first dose of treatment (Section 7.1.2).

- Added 'For the retrospective central confirmation all subjects will be required to provide a tumor tissue sample at screening prior to study treatment, as either a tumor block or a minimum of 25 FFPE slides (see Section 7.2.4)' in Section 7.1.2 for clarification.
- Added 'BRAF mutation status by local assessment' eCRF to the eCRFs to be completed for screen failures (Section 7.1.2.2).
- Added 'skin exam' as part of the physical examination to be performed at screening (Section 7.1.2.3), to a short physical exam and to a periodic exam (every 8 weeks) (Section 7.2.2.1) to closely examine for new cutaneous malignancies and skin toxicities, which are AESIs of dabrafenib and trametinib.
- Added that physical examinations, laboratory, ECG, and ECHO assessments performed as part of the EOT in part 3 will not require to be repeated if performed within 7 days prior to the first dose of study treatment in crossover (Section 7.1.3).
- Clarified that central review will be performed for both RECIST and irRECIST in Section 7.2.1 to be consistent with Section 10.4.
- Added an instruction that new cutaneous lesions need to be color photographed including a metric ruler to measure the size of the lesion (Section 7.2.1.2).
- Added an instruction for physical examination that 'if indicated based on medical history and/or symptoms, rectal, external genitalia, breast, and pelvic exams will be performed' (Section 7.2.2.1).
- Added chloride to chemistry assessment to Table 7-7 to obtain additional information for potential electrolyte changes (Section 7.2.2.6).
- Corrected an error in hepatitis monitoring period from 'until 30-day safety follow up' to 'until 150 day safety follow up' (Section 7.2.2.6.7).
- Updated a footnote in blood collection schedule for PDR001 PK and IG table (Table 7-10) to clarify that PK and IG samples will not be collected during Cycle 1 for the DL-1b dosing regimen during part 1 or if DL-1b is the selected dosing regimen for part 2.
- Deleted a footnote in blood collection schedule for PDR001/(placebo) PK and IG table (Tables 7-10, 7-11, 7-12) regarding the dose reference ID for the end of treatment, 30 day safety and 150 day safety follow up visits since dose reference IDs are not applicable to these samples.
- Added different sets of dose reference IDs for dabrafenib and trametinib in PK collection tables (Tables 7-13, 7-14, 7-15) to collect dosing date and time for the last dose taken before PK collection. Inserted a footnote to ensure collection of the date and time for dosing before and after PK sample collection (Tables 7-13, 7-14, 7-15).
- Increased blood volume (10 mL to 12 mL) to be collected for PBMC for part 3 in Table 7-16 and Section 7.2.4.2.4 to accommodate two flow cytometry assays for biomarker analysis in part 3.
- Drug Safety and Epidemiology (DS&E) department at Novartis is now Chief Medical Office and Patient Safety (CMO&PS). This has been changed consistently throughout the documents (Sections 8.2.2, 8.4).
- Corrected the definition of Full Analysis Set (FAS) and Safety Set in Section 10.1.1 to clarify that subjects in non-randomized part 1 and part 2 will be analyzed according to the study treatment they were assigned to (as opposed to treatment actually received).

Supplemental material BMJ Publishing Group Limited (BMJ) disclaims all liability and responsibility arising from any reliance placed on this supplemental material which has been supplied by the author(s) J Immunother Cancer

- Inserted the following definitions of immunogenicity prevalence and incidence sets under 'Other analysis sets (Section 10.1.6)'. 'The immunogenicity prevalence set includes all subjects in the full analysis set with a determinant baseline IG sample **or** at least one determinant post-baseline IG sample and the immunogenicity incidence set includes all subjects in the Immunogenicity prevalence set with a determinant baseline IG sample **and** at least one determinant post-baseline IG sample.'
- Revised the definition of observation period (Section 10.5.3.1). The end of 'on-treatment period' was changed to '30 days after date of last actual administration of any study medication' and the beginning of the 'post treatment' was changed to 'at day 31 after last dose of study medication'. Inserted a paragraph stating that summaries for deaths, all AEs, and all SAEs collected up to 150 days after last administration of PDR001 will be reported.
- Revised wording regarding part 2 biomarker cohort sample size (Section 10.8) by changing 'up to 20 evaluable subjects' to 'approximately 20 evaluable subjects'.
- Corrected errors in Table 14-6 (Section 14.2.3.1) and changed the dose level of dabrafenib from 35 mg BID to 350 mg BID, number of evaluable patients from 4 to 6, and number of DLTs from 0 to 1.

As part of this amendment, minor editorial changes, to improve flow and consistency, and correction of spelling errors or typographical errors, have been made throughout the protocol.

IRBs/IECs

A copy of this amended protocol will be sent to the Institutional Review Board (IRBs)/Independent Ethics Committee (IECs) and Health Authorities.

The changes described in this amended protocol require IRB/IEC approval prior to implementation.

The changes herein affect the Informed Consent. Sites are required to update and submit for approval a revised Informed Consent that takes into account the changes described in this protocol amendment.

Amendment 1 (27-Jan-2017)

As of 24-Jan-2017, no patients have received study treatment in the trial and the original protocol has not been initiated at any study center globally.

Amendment rationale

The primary purpose of this protocol amendment is to clarify that double barrier contraception methods are not classified as highly effective methods, per Clinical Trial Facilitation Group (CTFG) guidelines.

Changes to the protocol

Changes to specific sections of the protocol are shown in the track changes version of the protocol using strike through red font for deletions and red underline for insertions.

The major change being made to the protocol due to this amendment is incorporated in the following section:

- Section 5.3.3 (exclusion criterion #28): Removed double barrier contraception from the list of highly effective methods of contraception, and added a note to clarify this exclusion criterion.

IRBs/IECs

A copy of this amended protocol will be sent to the Institutional Review Board (IRBs)/Independent Ethics Committee (IECs) and Health Authorities.

The changes described in this amended protocol are considered non-substantial and do not require IRB/IEC approval prior to implementation.

The changes herein affect the Informed Consent. Sites are required to update and send a revised Informed Consent that takes into account the changes described in this protocol amendment.

1 Background

1.1 Overview of disease pathogenesis, epidemiology and current treatment

Melanoma is the most aggressive form of all skin cancers. Worldwide, it is expected that over 232,000 people will be diagnosed with cutaneous melanoma each year and more than 55,000 people are expected to die of this disease annually (Ferlay 2015). In adults, cutaneous melanoma is the fifth most common cancer in men and the seventh most common cancer in women in the United States (USA), with approximately 76,400 new cases and 10,100 deaths expected in 2016 (SEER 2016). In Europe, the annual incidence of melanoma is somewhat lower than in the USA, but is the seventh most common cancer among women (Ferlay 2013, Siegel 2014). In Europe as a whole, approximately 100,300 cases were newly diagnosed and 22,000 patients died from this disease in 2012 (EUCAN 2012). The incidence of melanoma is increasing rapidly worldwide. This increase is the most rapid of any cancer with the exception of lung cancer in women (Jemal 2006).

Usually melanoma is diagnosed at an early stage in which surgical excision is curative in most cases. Patients who are at high-risk for developing metastatic disease may benefit from adjuvant therapy. The management of patients with unresectable or metastatic melanoma is more difficult, although recent advances have led to important improvements of clinical outcomes for this population. Many studies also showed elevated LDH was a negative prognostic factor for response and other efficacy outcomes (Sirott 1993, Eton 1998, Balch 2001). The key strategic approaches for these patients include immunotherapy with immune checkpoint inhibitors and/or targeted therapy that inhibits the mitogen-activated protein kinase (MAPK) pathway for *BRAF* V600 mutant melanoma (NCCN Guidelines® Melanoma V3 2016).

Immunotherapy

Under physiological conditions, immune checkpoints are crucial for the maintenance of self-tolerance and autoimmunity. However, the expression of immune checkpoint proteins can be dysregulated by cancer cells which is an important immune resistance mechanism (Pardoll 2012). Immune checkpoints play a key role as negative regulators that restrain an effective anti-tumor response against melanoma (Pardoll 2012).

Cytotoxic T lymphocyte antigen 4 (CTLA-4) was the first checkpoint to be clinically targeted with ipilimumab. The inhibitory CTLA-4 receptor is expressed exclusively on T cells where it primarily regulates the amplitude of T cell activation in the early stages (Pardoll 2012). Ipilimumab (Yervoy®) is a monoclonal antibody to CTLA-4 and was the first drug to demonstrate a survival benefit in melanoma. The pivotal phase III study of ipilimumab showed an overall survival (OS) improvement in subjects with previously treated metastatic melanoma with HLA-A2*0201 genotype, as compared with an investigational peptide vaccine gp100 (Hodi 2010). In a second phase III trial in treatment naive patients the combination of ipilimumab plus dacarbazine improved OS versus placebo plus dacarbazine (Robert 2011). Importantly, extended follow-up showed that ipilimumab resulted in long-term survival in approximately 20% of patients (Maio 2015). In 2011, ipilimumab was approved by the U.S. Food and Drug Administration (FDA) for the treatment of unresectable or metastatic melanoma (FDA 2011).

Programmed cell death receptor 1 (PD-1) is another inhibitory immune checkpoint receptor expressed by T cells. Its primary ligand 1 (PD-L1) is frequently expressed in the tumor microenvironment, including cancer cells and tumor-infiltrating macrophages. PD-1 has a second ligand (PD-L2), which is preferentially expressed by antigen-presenting cells (Pardoll 2012). Pembrolizumab (Keytruda®) and nivolumab (Opdivo®) are monoclonal antibodies that directly block the interaction between PD-1 and its ligands, PD-L1 and PD-L2. Pembrolizumab received accelerated approval by the FDA based on response rate and durability of response for the treatment of patients with disease progression following ipilimumab, and if *BRAF* V600 mutation positive, a *BRAF* V600 inhibitor (FDA 2014). The approval was based on the results of an open-label, randomized cohort within a large, multi-stage, multiple cohort dose-finding and activity-estimating study (KEYNOTE-001). In the sub-study, patients with unresectable or metastatic melanoma which had prior treatment with ipilimumab or *BRAF* or MEK inhibitor if *BRAF* V600 positive were randomly assigned to pembrolizumab 2 mg/kg Q3W or 10 mg/kg Q3W. Anti-tumor activity was similar between the two doses with respect to both overall response rate (ORR) and duration of response (DOR) (Robert 2014). The KEYNOTE 002 study of pembrolizumab versus investigator-choice chemotherapy confirmed the benefits of pembrolizumab in ipilimumab-refractory melanoma patients (Ribas 2015). Importantly, pembrolizumab demonstrated statistically significant improvements in OS and progression free survival (PFS) for patients randomized to pembrolizumab as compared to ipilimumab in a phase III study (KEYNOTE-006) in previously treated and untreated unresectable or metastatic melanoma (Robert 2015). Two phase III studies have demonstrated efficacy for nivolumab: In CheckMate 037, nivolumab demonstrated significantly improved ORR, PFS and OS versus chemotherapy in ipilimumab-refractory patients (Weber 2015). In the second phase III study (CheckMate 066), nivolumab showed an improved ORR and PFS versus dacarbazine, among previously untreated patients without a *BRAF* V600 mutation (Robert 2015). No head to head trial has been done comparing pembrolizumab with nivolumab in melanoma.

Despite important clinical benefits, checkpoint inhibition is associated with a unique spectrum of side effects termed immune-related adverse events (irAEs). IrAEs include dermatologic, gastrointestinal, hepatic, endocrine, and other less common inflammatory events. IrAEs are more common with the anti-CTLA-4 antibody ipilimumab than with the anti-PD-1 agents pembrolizumab (Robert 2015) or nivolumab (Postow 2015). In general, treatment of moderate or severe irAEs requires interruption of the checkpoint inhibitor and the use of corticosteroid immunosuppression (NCCN Guidelines for Patients® Melanoma 2016).

The combination of CTLA-4 blockade with ipilimumab and PD-1 blockade with nivolumab has resulted in longer PFS and higher ORR than either agent alone at the expense of significantly increased toxicity (Larkin 2015, Postow 2015). Overall survival results from the phase III study (CheckMate 067) are pending.

Targeted therapy (see also Section 1.2.2)

MAPK pathways are evolutionarily conserved kinase modules that link extracellular signals to the machinery that controls fundamental cellular processes such as growth, proliferation, differentiation, migration and apoptosis (Dhillon 2007). This signaling pathway is an attractive therapeutic target because it is aberrantly activated in many human cancers. It can be activated by mutations in *BRAF* kinase, which have been found in almost 50% of metastatic melanomas.

In 2011 the selective small molecule *BRAF* V600-inhibitor vemurafenib received FDA approval as the first molecular-targeted agent for unresectable or metastatic melanoma harboring *BRAF* V600E mutations. In the pivotal phase III study (BRIM-3) treatment with vemurafenib resulted in a significant improvement of PFS and OS compared to chemotherapy alone (Chapman 2011). In 2013, another *BRAF* V600 inhibitor, dabrafenib, was approved by the FDA based on significant improvement of clinical benefit compared to DTIC chemotherapy in the pivotal phase III study BREAK-3 (Hauschild 2012). Trametinib, a MEK inhibitor, successfully demonstrated clinical benefit in the same patient population in the pivotal phase III study (METRIC) and was also approved by the FDA at the same time (Flaherty 2012).

While monotherapy with either dabrafenib or trametinib represented a significant advance in the treatment of *BRAF* V600 mutated, unresectable or metastatic melanoma, responses were not observed in all patients; underscoring the fact that intrinsic mechanisms of resistance were in place and approximately half of the patients treated with a *BRAF* V600 or MEK inhibitor as monotherapy progressed within 5 to 7 months of starting treatment. Therefore, the combination of a selective and potent *BRAF* V600 and a MEK-inhibitor is favored to address specific molecular mechanisms of intrinsic and acquired resistance to a *BRAF* V600 inhibitor monotherapy (Nissan 2011). Data from a the phase I/II study, BRF113220 established that the approved doses of dabrafenib and trametinib were safe when administered in combination, and additionally demonstrated a statistically significant and clinically meaningful improvement in several key efficacy endpoints when compared directly to dabrafenib monotherapy. These efficacy measures were also superior when indirectly compared to trametinib monotherapy. The superiority of the efficacy of the combination over *BRAF* V600 inhibitor monotherapy was subsequently confirmed across two randomized phase III studies where dabrafenib in combination with trametinib consistently demonstrated clinical benefit when compared to dabrafenib (COMBI-d) or vemurafenib monotherapy (COMBI-v). Statistically significant and clinically meaningful improvements were observed for OS, PFS, ORR, DOR and quality of life (QoL) with manageable adverse events profile (Long 2014, Robert 2014, Schadendorf 2015). Another combination of *BRAF* V600 and MEK inhibitor, vemurafenib and cobimetinib, also successfully demonstrated clinical benefit in *BRAF* V600-mutated metastatic melanoma as well as superior efficacy when compared with *BRAF* V600 inhibitor monotherapy with vemurafenib (Larkin 2014).

At present, the combination of immune checkpoint inhibitors or the combination of targeted therapy with or in sequence with immunotherapy are being investigated in different settings to further improve the clinical outcome for patients with unresectable or metastatic melanoma.

1.2 Introduction to investigational treatment(s) and other study treatment(s)

1.2.1 Overview of PDR001

PDR001 is a high-affinity, ligand-blocking, humanized IgG4 antibody directed against PD-1 that blocks the binding of PD-L1 and PD-L2. PD-1 is a critical immune checkpoint receptor that is expressed on CD4 and CD8 positive T cells upon activation (Freeman 2008). Engagement of PD-1 by its ligands, PD-L1 and PD-L2, transduces a signal that inhibits T-cell proliferation, cytokine production, and cytolytic function (Riley 2009). Monoclonal antibody

(mAb) inhibitors of immunological checkpoints, including PD-1 and PD-L1, have demonstrated significant antitumor activity in patients with various solid tumors. For further details please refer to the latest PDR0001 Investigator's Brochure.

1.2.1.1 Non-clinical experience of PDR001

PDR001 binds specifically and with high affinity to human PD-1 and enhances interleukin-2 (IL-2) production in *in vitro* lymphocyte stimulation assays. It does not cross react with rodent PD-1; therefore, toxicology studies were performed only in cynomolgus monkeys where there was acceptable cross reactivity with monkey PD-1. Repeat administration of PDR001 to monkeys was tolerated at all doses tested up to 100 mg/kg/week for 5 weeks in the GLP toxicology single-agent study. No drug-related in-life, mortality, organ weight changes, or macroscopic findings were noted. There were no PDR001-related effects seen in any of the safety pharmacology endpoints assessed (cardiovascular, neurobehavioral, and respiratory). Macrophage infiltrates into the splenic white pulp were observed in animals given 100 mg/kg/week and mononuclear cell infiltrates, often associated with fibrosis, around the injection site blood vessel (saphenous vein) in a few animals given ≥25 mg/kg/week. These PDR001-related microscopic changes were fully reversible after an eight week recovery. Additionally, mostly low grade mononuclear infiltrates in the vascular and perivascular space in several tissues of main and recovery treated animals and in recovery controls were observed but with a slightly higher incidence in treated animals. No evidence of parenchymal damage was associated with the vascular/perivascular changes in any of the organs examined and the changes were not associated with any frank tissue injury. Dose-proportional exposure to PDR001 in each dose group was confirmed. Anti-drug antibodies (ADA) to PDR001 were observed in some PDR001 treated cynomolgus monkeys. A trend of reduced drug exposure was observed in these ADA-positive animals. Based on the toxicology studies with PDR001 as a single-agent, the Highest Non-Severely Toxic Dose (HNSTD) dose is 100 mg/kg. For further details, please refer to the latest PDR001 Investigator's Brochure.

1.2.1.2 Clinical experience with PDR001

There are two ongoing studies with PDR001; [CPDR001X2101] and [CLAG525X2101C]. Sixty-four patients have received PDR001 either as a single-agent or in combination with LAG525 (an anti-LAG3 antibody).

In the first-in-human [CPDR001X2101] study, a total of 58 patients (as of cut-off date of 27-Mar-2016) were exposed to PDR001 single-agent at 1 to 10 mg/kg on an every 2 weeks (Q2W) schedule or at 3 and 5 mg/kg every 4 weeks (Q4W) schedule. The patient population treated in this study included patients with a diverse range of advanced solid tumors, where the most frequent tumor types were renal (6 patients) and liposarcoma (3 patients). The median duration of exposure was 14 weeks (range 2–46) in a patient population that had been heavily pretreated (59% of the patients were previously exposed to ≥ 3 lines). The preliminary pharmacokinetic (PK) parameters from the study demonstrate approximately dose-proportional increases in exposure (C1D1 AUC0-336h) from 1 to 10 mg/kg with the half-life of PDR001 of 20 days for both schedules. Accumulation of approximately 2.1-3.4 –fold was observed with Q2W dosing and 1.6-2.2 –fold with Q4W dosing. No Dose Limiting Toxicities (DLTs) were reported. Population PK analysis indicated that the changes in exposure due to patient weight are minimal

across the anticipated weight range of 30 to 150 kg for the patient population. Therefore, a flat dosing scheme was selected. The recommended phase 2 dose (RP2D) therefore was established at 400 mg Q4W or 300 mg Q3 weeks. Adverse events (AEs), all grades, regardless of relationship to study drug, were reported in 58 patients (100%) overall, with the most frequent AEs (in ≥20% of patients) being nausea (40%), anemia (33%), fatigue (36%), diarrhea (29%), dyspnea (29%), vomiting (24%), abdominal pain (22%), decreased appetite (22%) and constipation (21%) which are consistent with the AEs reported in studies with other PD-1 inhibitors and with the AEs commonly reported for patients with advanced solid malignancies (Opdivo (Nivolumab) USPI 2015, Keytruda (pembrolizumab) USPI 2015). The AE profile was similar across the different dose groups. The most frequent Grade 3/4 AEs (in ≥10% of patients) regardless of causality was anemia (15.5%). The most frequent AEs (≥10% of patients) suspected to study drug were fatigue (20.7%), diarrhea (15.5%), nausea (10.3%), and pruritus (10.3%). Grade 3/4 AEs related to study drug were rare, occurring in only 2 (3.4%) patients; one case of autoimmune colitis and another of hypophosphataemia. There were 4 deaths while on-treatment but none related to study drug.

As of cut-off date of 27-Mar-2016, serious adverse events (SAEs), all grades, regardless of relationship to study drug, were reported in 23 (39.7%) patients in study CPDR001X2101. Most of the SAEs were Grade 3 or 4 (22 out of 23). One SAE (Grade 3 autoimmune colitis) was suspected to be related to study treatment. This patient was treated with PDR001 at 10 mg/kg Q2W and the patient presented with sudden onset of diarrhea after four doses of treatment. Diagnosis of autoimmune colitis was confirmed by colonoscopy with biopsy. Treatment with PDR001 was interrupted and the patient's diarrhea responded immediately to treatment with corticosteroids.

In terms of preliminary activity, the disease control rate across the wide range of tumor types was 41%, with one partial response in a patient with a metastatic, atypical pulmonary carcinoid tumor (Naing 2016). Although follow-up data are premature, preliminary data from the ongoing phase II portion of the study already showed confirmed responses per RECIST 1.1 in two of 16 evaluable patients with unresectable or metastatic melanoma (cut-off date: 26 August 2016). As of 24 October 2016, a total of five investigator notifications due to serious, unexpected, possibly related adverse events have been issued during phase I and the ongoing phase II.

1.2.2 Overview of dabrafenib and trametinib

Dabrafenib (Tafinlar®) is an orally bioavailable, potent and selective RAF kinase inhibitor of human wild-type (WT) BRAF and CRAF enzymes as well as the mutant forms of the BRAF enzyme, *BRAF* V600E, *BRAF* V600K, and *BRAF* V600D. The mechanism of action of dabrafenib is consistent with competitive inhibition of ATP binding.

Dabrafenib was first approved by the FDA in 2013 as a single-agent oral treatment for unresectable or metastatic melanoma in adult patients with the *BRAF* V600E mutation. Dabrafenib is currently also approved in the EU, Switzerland, Canada, Australia and multiple other countries for the treatment of adult patients with unresectable or metastatic melanoma with a *BRAF* V600 mutation. Prior to initiation of dabrafenib, patients must have confirmation of tumor *BRAF* V600 mutation. These approvals include the following limitation of use: dabrafenib is not indicated for the treatment of wild-type BRAF melanoma. The recommended

dose of dabrafenib is 150 mg (two 75 mg capsules) twice daily (BID) (corresponding to a total daily dose of 300 mg).

Trametinib (Mekinist®) is a reversible and highly selective allosteric inhibitor of MEK1 and MEK2. MEK proteins are critical components of the MAPK pathway which is commonly hyperactivated in tumor cells. Oncogenic mutations in both BRAF and RAS signal through MEK1 or MEK2. Trametinib was first approved by the FDA on 2013 as a single-agent oral treatment for unresectable or metastatic melanoma in adult patients with *BRAF* V600 mutations. Trametinib is currently also approved in the EU, Canada, and Australia and multiple other countries for the treatment of adult patients with unresectable or metastatic melanoma. The recommended dose of trametinib is 2 mg once daily (QD).

Trametinib in combination with dabrafenib was first approved by the FDA in 2014 to treat unresectable or metastatic melanoma in adult patients with *BRAF* V600 mutations. The combination therapy is currently also approved in the EU, Australia, Chile, Canada, and multiple other countries.

1.2.2.1 Nonclinical experience with dabrafenib and trametinib combination

In vitro and *in vivo* preclinical data indicated increased anti-tumor activity by the combination of a *BRAF* V600-inhibitor dabrafenib and a MEK inhibitor trametinib. The combination of dabrafenib and trametinib has demonstrated enhanced anti-proliferative activity against a panel of *BRAF*-mutant cell lines *in vitro*, suggesting a synergistic effect of dabrafenib and trametinib in addressing primary resistance to each single agent. In addition, the combination was also effective in inhibiting the growth of dabrafenib- resistant BRAF-mutant melanoma cell clones indicating the potential ability of the combination therapy to overcome acquired resistance. This cell-line data are comparable to recently published *in vitro* results of other experimental BRAF- and MEK-inhibitor combinations (Corcoran 2010, Emery 2009). Moreover, the combination of dabrafenib and trametinib demonstrated improved activity in mouse xenograft models of BRAF mutant melanoma compared to either single agent. Furthermore, in skin toxicity studies performed in rats, the addition of trametinib to dabrafenib prevented the development of proliferative skin lesions observed following treatment with dabrafenib alone. These results suggest that the addition of a MEK inhibitor to a BRAF inhibitor may suppress the proliferative signals in normal skin cells which can lead to the development of hyperproliferative skin lesions including keratoacanthomas and cutaneous squamous-cell carcinomas frequently observed in clinical trials involving BRAF inhibitors (Flaherty 2010, Chapman 2011, Robert 2011). Similar results have been published with another combination of *BRAF* V600 and MEK inhibitors (Carnahan 2010).

1.2.2.2 Clinical experience with dabrafenib and trametinib combination

Dabrafenib and trametinib are approved as monotherapy or in combination for the treatment of adult patients with unresectable or metastatic melanoma with a *BRAF* V600 mutation in the US, EU and over 20 other countries including Switzerland, Canada and Australia. For more details, please refer to the most current dabrafenib Investigator's Brochure and the approved product information.

1.2.2.2.1 Human pharmacokinetics

Dabrafenib is absorbed orally with median time to achieve tmax of 2 hours post-dose. Mean absolute bioavailability of oral dabrafenib is 95% (90% CI: 81 to 110%). Dabrafenib exposure (Cmax and AUC) increased in a dose proportional manner between 12 and 300 mg following single-dose administration, but the increase was less than dose proportional after repeat twice daily dosing. There was a decrease in exposure observed with repeat dosing, likely due to induction of its own metabolism. Mean accumulation AUC Day 18/Day 1 ratios was 0.73. Dabrafenib binds to human plasma protein and is 99.7% bound. The metabolism of dabrafenib is primarily mediated by cytochromes P450 (CYP) 2C8 and CYP3A4 to form hydroxy-dabrafenib, which is further oxidized via CYP3A4 to form carboxy-dabrafenib. Carboxy-dabrafenib can be decarboxylated via a non-enzymatic process to form desmethyl-dabrafenib. Mean metabolite to parent AUC ratios following repeat dose administration were 0.9, 11 and 0.7 for hydroxy-, carboxy-, and desmethyl-dabrafenib, respectively. Based on exposure, relative potency, and pharmacokinetic properties, both hydroxy- and desmethyl-dabrafenib are likely to contribute to the clinical activity of dabrafenib; while the activity of carboxy-dabrafenib is not likely to be significant. Dabrafenib terminal half-life is 8 hours. Faecal excretion is the major route of elimination after oral dosing, accounting for 71% of a radioactive dose while urinary excretion accounted for 23% of radioactivity.

Trametinib is absorbed orally with median time to achieve peak concentrations (tmax) of 1.5 hours post-dose. The mean absolute bioavailability of a single 2 mg tablet dose is 72%. The increase in exposure (Cmax and AUC) was dose proportional following repeat dosing. Trametinib accumulates with repeat daily dosing with a mean accumulation ratio of 6.0 following a 2 mg once daily dose. Mean terminal half-life is 127 hours (5.3 days) after single oral dose administration. Steady-state was achieved by Day 15. *In vitro* and *in vivo* studies demonstrated that trametinib is metabolized predominantly via deacetylation alone or in combination with mono-oxygenation. The deacetylated metabolite was further metabolized by glucuronidation. The deacetylation is mediated by the carboxyl-esterases 1b, 1c and 2, with possible contributions by other hydrolytic enzymes. Drug-related material was excreted predominantly in the feces (≥ 81% of recovered radioactivity) and to a small extent in urine (≤ 19%). Less than 0.1% of the excreted dose was recovered as parent in urine.

Repeated co-administration of dabrafenib 150 mg twice daily and trametinib 2 mg once daily resulted in a 16% increase in dabrafenib Cmax and a 23% increase in dabrafenib AUC. A small decrease in trametinib bioavailability, corresponding to a decrease in AUC of 12%, was estimated when dabrafenib is administered in combination with trametinib using a population pharmacokinetic analysis. These changes in dabrafenib or trametinib Cmax and AUC are considered not clinically relevant.

1.2.2.2.2 Drug-drug interactions

Dabrafenib induces CYP3A4- and CYP2C9-mediated metabolism and may induce other enzymes and transporters. Co-administration of dabrafenib and medicinal products which are affected by the induction of CYP3A4 or CYP2C9 such as hormonal contraceptives, warfarin or dexamethasone may result in decreased concentrations and loss of efficacy. If co-administration of these medications is necessary, monitor subjects for loss of efficacy or consider substitutions of these medicinal products.

Medicinal products that are strong inhibitors or inducers of CYP2C8 or CYP3A4 are likely to increase or decrease, respectively, dabrafenib concentrations. Alternative agents should be considered during administration with dabrafenib when possible. Use caution if strong inhibitors (e.g., ketoconazole, nefazodone, clarithromycin, ritonavir, gemfibrozil) or inducers (e.g., rifampin, phenytoin, carbamazepine, phenobarbital, St. John's wort) of CYP2C8 or CYP3A4 are coadministered with dabrafenib.

Trametinib is an inducer of CYP3A4 *in vitro*. However, trametinib's efficacious dose of 2 mg once daily results in a low systemic maximal concentration (22.2 ng/mL or 0.036 μM), relative to its *in vitro* inhibition potency of CYP enzymes and transporters, rendering the risk of an inhibitory effect of trametinib on the PK of co-administered CYP or transporter substrates low.

Trametinib is eliminated primarily via deacetylation and possibly other hydrolases. There is little evidence from clinical studies for drug interactions mediated by carboxylesterases. Trametinib is also a substrate of P-gp and BSEP. However, due to its high passive permeability, these active transport processes are likely of limited relevance. Therefore, a clinically relevant effect of a co-administered P-gp or BSEP inhibitor on the PK of trametinib is unlikely.

No clinically relevant drug-drug interaction (DDI) was observed after repeat dosing dabrafenib 150 mg twice daily and trametinib 2 mg once daily.

1.2.2.2.3 Clinical safety

Safety data pooled from the dabrafenib and trametinib combination arms of 2 completed phase III studies in subjects (N=559) with melanoma [MEK115306 and MEK116513] based on a cut-off date of 13 March 2015 are provided below. Data are in comparison to the dabrafenib monotherapy arm (N=211) of Study MEK115306 and the vemurafenib monotherapy arm (N=349) of Study MEK116513. The safety population consists of all subjects who received at least 1 dose of study treatment.

The median duration of follow-up, defined as the time from study start to last contact or death, was 19 months for the combination therapy group, 15 months for the vemurafenib group, and 16 months for the dabrafenib group.

Skin adverse effects were less frequent in the combination therapy group, especially the events linked to a paradoxical activation of the MAPK pathway, including both benign and malignant skin tumors. This finding is in accordance with preclinical models showing that the addition of MEK inhibitors may down regulate the BRAF inhibitor induced paradoxical activation of the MAPK pathway. Thus, secondary resistance and paradoxical activation of the MAPK pathway that occur with BRAF inhibitor monotherapy, which translate into rapid tumor relapses and emergence of skin cancers, respectively, were both improved by the combination therapy.

The incidences of subjects with any on-therapy AE, study treatment-related AE, SAEs, and study treatment-related SAE were similar between the treatment arms. The incidence of AEs leading to study treatment discontinuation, dose reductions, or dose interruptions was similar in the combination therapy group and the vemurafenib arm, and higher than in the dabrafenib monotherapy arm. The rate of fatal SAEs was higher in the combination therapy group compared to the two monotherapy groups. However, no subjects in the combination therapy group had fatal SAEs considered related to study treatment by the investigator.

Common adverse events

The most commonly occurring AEs (≥20%) in subjects treated with dabrafenib alone were hyperkeratosis, headache, pyrexia, arthralgia, papilloma, alopecia, and palmar-plantar erythrodysesthesia syndrome (PPES).

The most commonly occurring AEs (>20%) in subjects treated with trametinib alone were rash, diarrhea, and lymphedema.

The most commonly occurring AEs (>20%) in subjects treated with dabrafenib in combination with trametinib) were pyrexia, nausea, rash diarrhea, chills, headache, vomiting, hypertension, arthralgia, peripheral edema and cough and the most common Grade 3 or 4 AEs (≥5%) occurring in patients treated with the combination were hypertension and pyrexia.

The most common AEs leading to discontinuation (>1% of subjects) were pyrexia and ejection fraction decreased in the combination therapy group.

Adverse events leading to dose reduction or interruption of study treatment

Pyrexia, PPES, chills, fatigue, and headache were most frequent AEs leading to dose reduction of dabrafenib. The most common AEs leading to dose reduction (≥3% of subjects) were pyrexia and ejection fraction decreased in the combination therapy group. Of the most common events, dose reductions due to pyrexia were more common in the combination therapy group compared with each monotherapy arm.

Of the most common AEs leading to dose interruption, dose interruptions due to pyrexia were more common in the combination therapy group compared with either monotherapy arm.

The incidences of pyrexia and neutropenia were higher (≥10% difference) in the combination therapy group compared with either monotherapy arm. In addition, the incidences of diarrhea, hepatic events, hypertension and edema were higher (≥10% difference) in the combination therapy group compared with the dabrafenib monotherapy arm only, but similar to vemurafenib, and the incidence of bleeding events was higher in the combination therapy group compared with the vemurafenib monotherapy arm.

1.2.2.2.4 Clinical efficacy

Both dabrafenib and trametinib have proven anti-tumor activity as monotherapy in *BRAF* V600 mutant metastatic melanoma (BREAK 3 and METRIC trials respectively).

Combining a *BRAF* V600 inhibitor with a MEK inhibitor addresses the limitations of single agent *BRAF* V600 inhibitors and results in a significant delay in the emergence of resistance, with a longer median PFS than with dabrafenib alone as well as a decreased incidence of *BRAF* V600 inhibitor induced skin tumors (Long 2014, Long 2015). The efficacy of dabrafenib and trametinib combination therapy for *BRAF* V600 mutation positive metastatic melanoma has been demonstrated in two pivotal randomized phase III studies (COMBI-d and COMBI-v) with patients harboring a *BRAF* V600 mutation.

Study MEK115306 [COMBI-d] was a two-arm, double-blind, randomized, phase III study comparing dabrafenib and trametinib combination therapy to dabrafenib monotherapy. Patients with histologically confirmed cutaneous melanoma that is either unresectable (Stage IIIC) or metastatic (Stage IV), and *BRAF* V600E/K mutation positive were enrolled. The primary

endpoint was investigator-assessed PFS for patients receiving the combination therapy compared with those receiving dabrafenib monotherapy. In this study, the median PFS was 11 months in the dabrafenib and trametinib combination arm versus 8.8 months in the dabrafenib arm (p<0.001). The overall response rate was 69% in the dabrafenib and trametinib combination arm versus 53% in dabrafenib arm (p=0.001). Median OS was 25.1 months in the dabrafenib and trametinib combination arm versus 18.7 months in the dabrafenib only arm (p=0.011). Dabrafenib and trametinib combination therapy provided better preservation of health related quality of life (HRQoL) and pain improvements versus dabrafenib monotherapy (Robert 2015).

Study MEK116513 [COMBI-v] was a two-arm, open-label, randomized, phase III study comparing dabrafenib and trametinib combination therapy to vemurafenib monotherapy. Patients with histologically confirmed cutaneous melanoma that is either unresectable (Stage IIIC) or metastatic (Stage IV), and *BRAF* V600E/K mutation positive were enrolled. The primary endpoint was OS for patients receiving the combination therapy compared with those receiving vemurafenib only. Results from this study showed that the combination of dabrafenib and trametinib significantly (p<0.001) improved OS compared to vemurafenib monotherapy, and median OS was 25.6 months in the dabrafenib and trametinib combination arm versus 18 months in the vemurafenib only arm. A statistically significant reduction of 34% in the risk of death among patients receiving combination therapy was observed in the study. The median PFS was 12.6 months in the dabrafenib and trametinib combination arm versus 7.3 months in the vemurafenib arm (p<0.001). The ORR was 64% in the dabrafenib and trametinib combination arm versus 51% in vemurafenib arm (p<0.001). In addition, an analysis of the patients' HRQoL showed statistically significant and clinically meaningful improvements among those receiving the combination of dabrafenib and trametinib, compared to those receiving vemurafenib monotherapy (Long 2014, Robert 2015).

In these two randomized phase III studies, dabrafenib in combination with trametinib consistently demonstrated clinical benefit in *BRAF* V600 mutant metastatic melanoma when compared to *BRAF* V600 inhibitor monotherapy (dabrafenib or vemurafenib), that was established by statistically significant and clinically meaningful improvements in OS, PFS, ORR, DOR and quality of life without increased toxicity. In both studies, patients with elevated LDH at baseline consistently had a poorer clinical outcome in terms of PFS and OS compared to patients with normal LDH.

2 Rationale

2.1 Study rationale and purpose

Both immunotherapy with checkpoint inhibitors and targeted therapy are important in the management of *BRAF* V600-mutated unresectable or metastatic melanoma. Immunotherapy has gained much interest due to the promise of long-term disease control. To date, the main limitation of single agent immune checkpoint inhibitor therapy is that only a subset of patients respond, likely due to evasion of immunosurveillance and suppression of effector function in the tumor microenvironment. Strategies to further improve these response rates are needed (Wargo 2014). Combination immunotherapy with ipilimumab and nivolumab has resulted in longer PFS and higher ORR than either agent alone, however at the expense of significantly increased toxicity (Larkin 2015, Postow 2015). While rapid and deep responses have been

observed with the combination of *BRAF* V600 and MEK inhibitor therapies, most patients progress within a year and there are only a few complete responses. Thus, strategies to improve the durability of these responses are needed (Wargo 2014).

This randomized, double-blind, placebo-controlled, phase III study will evaluate the efficacy and safety of the combination of the anti-PD1 antibody PDR001 in combination with the *BRAF* V600 inhibitor dabrafenib and the MEK inhibitor trametinib in previously untreated *BRAF* V600 mutated melanoma. Based on preclinical and preliminary clinical data, it is expected that dabrafenib in combination with trametinib alter the immune microenvironment in a way that could be beneficial for combination with PD-1 inhibitors.

Preclinical data

Early data for the potential synergy of targeted therapy with immune checkpoint inhibitors in melanoma was based on observations that oncogenic *BRAF* can lead to immune escape in melanoma (Sumimoto 2006) and that blocking *BRAF* activity leads to increased expression of melanoma differentiation antigens *in vitro* (Kono 2006) through release of transcriptional repression and subsequent expression of MITF targets including MART-1, gp100, TRP-1 and TRP-2 (Boni 2010).

The effect of MAPK pathway inhibition on T cell activation and signaling was also studied *in vitro*. While *BRAF* V600 inhibitors did not show any adverse effects on T cell function and may even augment their function through paradoxical signaling through RAS-GTP (Callahan 2014), MEK inhibitors showed dose-dependent inhibition of T cell function *in vitro* (Boni 2010, Vella 2014). The *in vivo* anti-tumor effect of trametinib was explored in a murine immunocompetent BALB/C syngeneic CT26 tumor model. In this study, trametinib monotherapy increased $CD4^+$ tumor infiltrating lymphocytes (TILs) and did not negatively affect the prevalence of $CD8^+$ TILs. Importantly the combination of trametinib with murine anti-PD-1 resulted in an effective anti-tumor response in the KRAS-mutant CT26 colorectal tumor syngeneic mouse model (Liu 2015). Another group showed that the addition of trametinib significantly improved the anti-tumor effect of anti-PD-1 in an immunocompetent mouse model of *BRAF* V600 mutant melanoma through improved effector T cell homing and preserved effector function, increased tumor antigen and MHC expression and cytokine release in the tumor microenvironment (Hu-Lieskovan 2015).

Clinical data

Additional supporting evidence for the potential synergy of *BRAF* V600 targeted therapy and checkpoint inhibitors comes from immune biomarker analysis of tumor samples from 16 patients with metastatic melanoma treated with vemurafenib or dabrafenib plus trametinib. In these analysis *BRAF* V600 inhibition (1) was associated with increased melanoma antigen expression (MART, TYRP-1, TYRP-2, gp100), (2) significantly increased $CD8^+$ TILs, (3) decreased expression of immunosuppressive cytokines in the tumor microenvironment, and (4) increased markers of T cell toxicity but also increased T cell exhaustion marker (TIM-3, PD-1). Another important observation was that the antigen expression and $CD8^+$ TILs were decreased at time of disease progression but could be restored through addition of dabrafenib and trametinib combination therapy (Frederick 2013, Wilmott 2012).

A phase I, open-label, multi-center, dose-finding study (NCT01767454) in subjects with unresectable or metastatic melanoma and *BRAF* V600E/K mutations was undertaken to characterized the safety of dabrafenib and ipilimumab with and without trametinib. The study consisted of two treatment arms: a doublet combination treatment arm of dabrafenib and ipilimumab, and a triple combination treatment arm of dabrafenib, trametinib and ipilimumab, with the potential for expansion cohorts for each treatment arm. In the doublet combination treatment arm, the starting dose of dabrafenib was the recommended monotherapy dose of 150 mg twice daily (BID). Ipilimumab was administered at the approved dose of 3 mg/kg every 3 weeks for a total of 4 doses in this study. Cohorts in the doublet arm followed a 3+3 enrollment. The study was closed due to DLTs in 2 subjects in the triplet combination treatment arm, as both subjects developed colitis with colonic perforation that raised the possibility of added toxicity with the triplet combination over ipilimumab as a single agent. The majority of AEs reported in both the doublet and triplet combination treatment arms were consistent with the safety profile of one or more of the study treatments. More than 65% of the subjects in both combination treatment arms had Grade 3 or Grade 4 AEs. No fatal events were reported.

In study KEYNOTE-022 (NCT02130466), dabrafenib and trametinib are currently being evaluated in combination with pembrolizumab in a phase I/II study in *BRAF* V600 mutant melanoma (Ribas 2016). Based on the phase I results, the recommended regimen for phase II is pembrolizumab 2 mg/kg Q3W plus dabrafenib 150 mg BID with trametinib 2 mg QD. The double-blind, placebo-controlled, randomized phase II part is ongoing. Fifteen patients were treated with the triplet combination and 14 patients were evaluable for DLT from the first two parts. Three of the 14 patients had DLTs and all were laboratory abnormalities. One patient had Grade 4 neutropenia that lasted about 2 weeks and resolved with drug interruption and subsequently continued study treatment, one patient had Grade 4 ALT elevation that resolved in about 2 weeks and study treatments were discontinued, and one patient had elevated Grade 3 ALT, AST and GGT elevation which were resolving and the patient was restarted on pembrolizumab alone. The adverse event profile was representative of the individual study agents. The recommended regimen was the labeled doses of each of the individual study medications. Preliminary efficacy data were promising with 5 patients achieving a confirmed response, 9 patients with an unconfirmed response and 13 of the 14 patients having a reduction in tumor size. The randomized portion of the study is ongoing, and as of 30 September 2016, 89 of the proposed 120 patients have been accrued. The interim analysis will be conducted when the first 80 enrolled patients have had at least a 6-month follow-up (Long 2016).

Additionally, the combination of dabrafenib and trametinib with the anti-PD-L1 antibody durvalumab in *BRAF* V600 mutant melanoma was tested (NCT02027961). Two doses of durvalumab were tested. Initially 6 patients received the labeled dose of dabrafenib and trametinib and durvalumab (3 mg/kg Q2W) and 20 patients received 10 mg/kg of durvalumab Q2W. Both doses were tolerated and were manageable and the AE profile was representative of the individual AE profile of each study medication. Combining data from both doses, the ORR was 69.2% (95% CI: 48.2-85.7), median DOR was 67.1 weeks, and 55.6% of responders had an ongoing response at the time of the data cut-off. (Ribas 2015).

2.2 Rationale for the study design

This phase III study will evaluate the safety and efficacy of the investigational anti-PD-1 antibody, PDR001 in combination with the approved doses of dabrafenib and trametinib in previously untreated patients with *BRAF* V600 mutant unresectable or metastatic melanoma (AJCC edition 7: stage IIIC/IV) in three parts:

Part 1: Safety run-in

At least 6 subjects, and up to approximately 18 subjects will be enrolled in part 1 of the study in order to establish the recommended phase 3 regimen (RP3R) for the randomized, double-blind, placebo-controlled part (part 3).

An adaptive Bayesian logistic regression model (BLRM) will be used to identify the RP3R. The BLRM will be guided by the Escalation with Overdose Control (EWOC) principle. Subjects with any history of CNS metastases will not be eligible for part 1. The starting dose (dose level 1) will be 400 mg PDR001 Q4W in combination with the approved doses for dabrafenib (150 mg BID) and trametinib (2 mg QD).

Part 2: Biomarker cohort

The potential for dabrafenib and trametinib to alter the immune microenvironment in a way that could be beneficial in terms of response to immunotherapy agents has been reported in preclinical models and patients treated with MAPK inhibitors (Schilling 2014, Liu 2015, Kakavand 2015). The tissue and blood samples from the biomarker cohort will be used to characterize baseline immune markers and to explore potential modulations of immune markers by the combination treatment. The biomarker cohort will enroll approximately 20 eligible subjects with previously untreated unresectable or metastatic *BRAF* V600 mutant melanoma. Subjects will receive PDR001 400 mg Q4W in combination with dabrafenib 150 mg BID and trametinib 2 mg QD. It is possible for additional dosing regimens to be used based on emerging data from part 1 and recruitment may be suspended at any time. Tumor tissue will be collected (1) at baseline, (2) after 2-3 weeks (Cycle 1 Day 15 + 1 week window), (3) 8-12 weeks after initiation of PDR001 therapy and (4) at disease progression per RECIST 1.1 and/or at a subsequent disease progression per response criteria for immunotherapy if subject continues treatment beyond progression. In addition to these mandatory tissue sample collections, optional biopsies may be collected during the course of this biomarker study depending on investigator assessment (e.g., in case the biopsy needs to be collected from a responding/shrinking lesion prior to cycle 3). Blood samples will be also collected at several time points (mandatory collection; as specified in Table 7-17).

Part 3: Randomized, double-blind, placebo-controlled part

The randomized, double-blind, placebo-controlled part of the study will open once the RP3R for the combination of PDR001 with dabrafenib and trametinib has been identified in part 1. Part 3 will enroll approximately 500 subjects with previously untreated unresectable or metastatic *BRAF* V600 mutant melanoma. Subjects will be randomized 1:1 to receive either PDR001 in combination with dabrafenib and trametinib or placebo in combination with dabrafenib and trametinib. A primary endpoint of PFS (based on investigator-assessed RECIST 1.1) and key secondary endpoint of OS will be assessed in this part of the study and subjects will be followed up until death. Other secondary efficacy endpoints include ORR, disease

control rate (DCR), and DOR based on RECIST 1.1. Other secondary endpoints include safety, tolerability and patient reported outcomes, as well as pharmacokinetics (PK) that will be evaluated to characterize the PK of PDR001, dabrafenib, and trametinib when administered in combination.

The number of PFS events required for the final PFS analysis (352) is sufficient to achieve 80% cumulative power to detect a significant difference between treatment groups based on the conservative assumption of a 5 months delayed treatment effect as observed in the KEYNOTE-022 study (Ascierto et al., 2018b) and the subsequent effect is of the same magnitude as currently assumed (HR=0.60). However, since the KEYNOTE-022 study showing a 5 months delayed effect was of relatively small sample size, there is still some uncertainty over the exact duration of the delayed effect. Therefore, the design for this study incorporates an interim PFS analysis, which will allow a good chance to detect a clinical relevant benefit at an earlier timepoint if the delayed effect is in fact less than 5 months duration. The timing and significance boundary of this interim PFS analysis is chosen so that the efficacy threshold is only met when the PFS treatment effect is sufficient and clinically relevant (i.e. using a stringent significance level with very little penalty for the final PFS analysis). The final PFS analysis will serve as a safeguard in case the delayed effect is 5 months in duration as observed in KEYNOTE-022 (Section 10.7, Section 10.8 and Section 10.9).

2.3 Rationale for dose and regimen selection

Rationale for PDR001 dose (See also Section 1.2.1.2)

The dose of PDR001 selected for dose level 1 (DL1) in the safety run-in part (part 1) of this study is 400 mg Q4W, administered as an intravenous infusion. In study [CPDR001X2101], PDR001 single-agent was administered as an intravenous infusion over 30 minutes at doses ranging from 1 to 10 mg/kg on an every 2 weeks (Q2W) schedule or at 3 and 5 mg/kg every 4 weeks (Q4W) schedules. Approximately dose proportional increase in exposure (C1D1 AUC0-336) was observed with doses from 1 to 10 mg/kg and no DLTs were observed. Accumulation of approximately 2.1-3.4-fold was observed with Q2W dosing and 1.6-2.2-fold with Q4W dosing. Population PK analysis indicated that the changes in exposure due to patient weight differences are minimal across the anticipated weight range of 30 to 150 kg for the patient population. Therefore, a flat dosing scheme was selected. Two recommended phase II dosing regimens have been established: 300 mg Q3W and 400 mg Q4W flat dosing schedules. A flat dose of 400 mg Q4W or 300 mg Q3W is expected to achieve a mean steady-state C_{trough} value higher than the ex vivo EC50 for antigen-stimulated IL-2 production, a translational biomarker for PD-1 blockade (Patnaik 2015). Based on the safety profile observed in study [CPDR001X2101] (Section 1.2) and the expected Ctrough values, 400 mg Q4W is expected to be a safe and efficacious dose. Subjects in the safety run-in part (part 1) will receive fixed dose combination of dabrafenib (150 mg BID) and trametinib (2 mg QD) and PDR001 400 mg every 4 weeks. If this initial dose level (DL1) is not tolerated, the next dose level (DL-1a and DL-1b) will be tested in parallel (see Section 4.1.2). The cohorts DL-1a and DL-1b use different and independent strategies to improve the tolerability of the triplet combination. DL-1a aims to reduce drug exposure for PDR001 by increasing the interval for the PDR001 from Q4W to Q8W. DL-1b allows a run-in period of 28 days for the combination treatment with dabrafenib and trametinib with subsequent addition of PDR001. This allows optimal AE management for

dabrafenib and trametinib combination prior to the administration of PDR001. The DLT observation period for DL-1b is 8 weeks from the initiation of the triplet treatment (Cycle 2 Day 1). As the strategies are independent and considered similar with regards to benefit/risk, they are tested in parallel.

Rationale for dabrafenib dose

The dabrafenib dose is 150 mg BID (corresponding to a total daily dose of 300 mg) which is the approved dose for patients with unresectable or metastatic melanoma with *BRAF* V600E or V600K mutations.

Rationale for trametinib dose

Trametinib will be administered as 2 mg QD, which is the approved dose for patients with unresectable or metastatic melanoma with *BRAF* V600E or V600K mutations.

Refer to Section 10.4.2 for details on how historical data were used to derive priors for the BLRM single agent (PDR001, dabrafenib, and trametinib) and interaction parameters, as well as for confirmation that the posterior probability of excessive toxicity for all 3 dosing regimens satisfy EWOC criteria (probability < 25%).

Rationale for combination dose

The approved doses of dabrafenib 150 mg twice daily (BID) and trametinib 2 mg once-daily (QD) will be used in all parts of the study as data from study BRF113220 showed decreased efficacy when lower doses of trametinib were used. Study BRF113220 was an open label study designed to assess the safety, pharmacokinetic and clinical activity of combination therapy with dabrafenib plus trametinib. Part C was a randomized phase 2 study in which subjects were randomly assigned in a 1:1:1 ratio to either receive 150 mg of dabrafenib BID plus trametinib QD, at a dose of either 1 mg or 2 mg, or dabrafenib 150 mg BID as monotherapy. The response rate and PFS of the dabrafenib 150 mg BID and trametinib 2 mg QD combination was superior to the other groups (Flaherty 2012, Long 2012).

Lower doses of PDR001 would result in a reduction of the required Ctrough level, thus 400 mg Q4W should be the starting dose in combination with the approved doses of dabrafenib and trametinib. From simulations based on the population PK model, the dose of 400 mg Q4W is predicted to achieve mean steady-state Ctrough concentrations of approximately 31 μg/mL (90% CI: 22-42 μg/mL). Finally preliminary data from studies combining dabrafenib and trametinib with other anti-PD-1 or anti-PD-L1 antibodies have reported acceptable DLT rates in combination with the approved doses of dabrafenib and trametinib.

2.4 Rationale for choice of combination drugs

Please refer to Section 2.1 for the rationale for choice of combination drugs.

2.5 Rationale for choice of comparator drugs

The combination therapy of dabrafenib and trametinib is approved for the treatment of metastatic *BRAF* V600 mutated melanoma and is an accepted treatment standard for this patient population globally (NCCN Guidelines for Patients® Melanoma V1.2017, Cutaneous

Melanoma: ESMO Clinical Practice Guidelines 2015, AWMF S3-Leitlinie Melanoma 2016) (Refer to Section 1.2.2).

2.6 Risks and benefits

Both dabrafenib and trametinib have proven anti-tumor activity as monotherapy in *BRAF* V600 mutant metastatic melanoma (BREAK-3 and METRIC trials respectively). Combining a *BRAF* V600 inhibitor with a MEK inhibitor addresses the limitations of single agent *BRAF* V600 inhibitors and results in a significant delay in the emergence of resistance, with a longer median PFS than with dabrafenib alone as well as a decreased incidence of *BRAF* V600 inhibitor induced skin tumors (Long 2014, Long 2015). The efficacy of dabrafenib and trametinib combination therapy for *BRAF* V600 mutation positive unresectable or metastatic melanoma has been demonstrated in two large randomized, pivotal phase III studies (COMBI-d and COMBI-v) and is an accepted treatment option globally for the population studied (NCCN Guidelines for Patients® Melanoma V1.2017, Cutaneous Melanoma: ESMO Clinical Practice Guidelines 2015, AWMF S3-Leitlinie Melanoma 2016). As of June 2016, an estimated 9,600 subjects, primarily with *BRAF* V600 mutated cancer, have received dabrafenib monotherapy or dabrafenib in combination with trametinib across the clinical development program.

PDR001 is an investigational humanized IgG4 monoclonal antibody specifically to anti-PD-1. This class of compound has demonstrated significant improvement in efficacy combined with tolerable and manageable safety profile, supporting recent regulatory approvals in melanoma, non-small cell lung cancer, head and neck squamous cell carcinoma (HNSCC), bladder cancer, renal cancer and classical Hodgkin's lymphoma (Opdivo® USPI 2015, Keytruda® USPI 2015).

Immune checkpoint inhibitors including anti-PD-1/PD-L1 may be associated with the occurrence of irAE. In general, irAE can potentially involve every organ system but gastrointestinal (colitis), dermatologic (rash), hepatic (hepatitis), pulmonary (pneumonitis), renal (nephritis) and endocrine toxicities (hypothyroidism, hyperthyroidism, diabetes, hypophysitis and hypopituitarism) are the most frequent and rarely CNS (encephalitis). These side effects are generally manageable but fatal events have been reported in some cases with checkpoint inhibitor compounds (Eggermont 2015, Friedman 2016, Hofmann 2016).

Based on the *in vitro* and *in vivo* preclinical, translational and preliminary clinical data it is expected that the addition of an anti-PD-1 antibody (like PDR001) to dabrafenib and trametinib may further relieve immunosuppression and allow a more robust anti-tumor response. Importantly, the combination of the approved anti-PD-1 antibody pembrolizumab with dabrafenib and trametinib showed a manageable toxicity profile in patients with *BRAF* V600-mutant melanoma and a phase II study is ongoing (KEYNOTE-022). Drug-drug interactions (DDI) with dabrafenib and trametinib are not anticipated as PDR001 is a monoclonal antibody, and is not metabolized by CYP450 enzymes, or transported by P-gp or related ABC membrane transporters. The clinical relevance of cytokines impacting the levels of P-gp and CYP450 with administration of PDR001 is unknown but clinically significant effects are considered unlikely. In the case of nivolumab, another mAb targeting PD-1 with the same mechanism of action, the Clinical Pharmacology and Biopharmaceutics Review (Opdivo® USPI 2015) states a lack of CYP interaction via cytokine modulation up to a dose of 10 mg/kg in renal cell carcinoma (RCC) patients. In addition, *in vitro* and *in vivo* data suggests that CYP3A4 contributes maximally 50% to the elimination of dabrafenib and is not involved to a relevant level in the elimination of

trametinib. In summary, the risk of DDI between dabrafenib, trametinib and PDR001 cannot be totally excluded, but it is anticipated to be low.

This study incorporates routine safety monitoring and regularly scheduled safety assessments to identify and report any potential safety issues. All subjects must have safety evaluations for 150 days after the last dose of study drug. Comprehensive dose modification, stopping rules and toxicity management plan for immune related events or drug related adverse events are provided in Section 6.3. Immune checkpoint inhibition is associated with a unique spectrum of side effects termed irAEs. Appropriate eligibility criteria and specific DLT definitions (Section 6.2.4), as well as specific dose modification and stopping rules along with adverse event management guidelines (Section 6.3.1), are included in this protocol. Recommended guidelines for prophylactic or supportive treatment for expected toxicities, including management of study-drug induced adverse events i.e., skin toxicity and diarrhea are provided in Section 6.3.1.

Based on the clinical experience with PDR001, which has been consistent with other monoclonal antibodies targeting PD-1, overlapping toxicities with trametinib and dabrafenib may include skin rash, diarrhea/ colitis, hepatic abnormalities, renal toxicity and pneumonitis. For these and other potential treatment-related adverse events of special interest, specific management and dose modification guidelines as well as stopping criteria are in place to safeguard patients (Section 6.3). In addition to following these guidelines, the risk to patients in this trial is further minimized by compliance with the eligibility criteria, study procedures and close clinical monitoring. Emerging safety data of this study will be monitored by an independent data monitoring committee (DMC).

In summary, the data from phase I-III studies on combination of dabrafenib and trametinib and phase I studies on monotherapy of PDR001 indicate that the majority of adverse events in patients receiving dabrafenib and trametinib combination or PDR001 as single agent have been manageable. Based on the available preclinical and clinical data, especially, the experience from phase I/II studies of dabrafenib and trametinib in combination with other anti PD-1/L1 mAbs, Novartis considers the potential risks of PDR001, dabrafenib and trametinib combination therapy are justified by the anticipated benefits that may be afforded to patients.

In addition, prior to enrollment, this protocol will undergo appropriate review by local and regional governance bodies including ethic committees and drug regulatory bodies.

3 Objectives and endpoints

Objectives and related endpoints are described in Table 3-1 below.

Table 3-1 Objectives and related endpoints

Objective	Endpoint	Analysis
**Primary**		Section 10.4.
**Safety run-in (part 1)**		
To determine the recommended regimen of PDR001 in combination with dabrafenib and trametinib for the randomized part (part 3)	• Incidence of DLTs during the first 8 weeks of treatment for each dose level associated with administration of PDR001 in combination of dabrafenib and trametinib.	
**Biomarker cohort (part 2)**		
To evaluate changes in PD-L1 levels and CD8+ cells upon treatment with PDR001 in combination with dabrafenib and trametinib	• Descriptive statistics of PD-L1 levels and CD8+ cells and changes from baseline by visit	
**Double-blind, randomized, placebo-controlled (part 3)**		
To compare the anti-tumor activity of PDR001 in combination with dabrafenib and trametinib versus placebo plus dabrafenib and trametinib as measured by PFS per investigator's assessment according to RECIST 1.1	• Investigator assessed PFS (according to RECIST 1.1)	
**Key Secondary**		Section 10.5.1.
**Double-blind, randomized, placebo-controlled (part 3)**		
To compare overall survival of PDR001 in combination with dabrafenib and trametinib versus placebo plus dabrafenib and trametinib	• Overall survival	
**Other Secondary**		Section 10.5.1.
**Safety run-in (part 1) and Biomarker cohort (part 2)**		
1) To determine safety and tolerability of PDR001 in combination with dabrafenib and trametinib	• Safety: Incidence and severity of AEs and SAEs, including changes in laboratory values, ECOG PS, vital signs, liver and cardiac parameters.<br>• Tolerability: Dose interruptions, reductions, and dose intensity	

Objective	Endpoint	Analysis
2) To evaluate preliminary anti-tumor activity of PDR001 in combination with dabrafenib and trametinib	• PFS, OS, ORR, DOR, DCR by investigator's assessment according to RECIST 1.1	
3) To characterize pharmacokinetics (PK) of PDR001, dabrafenib and trametinib when administered in combination	• PK parameters such as, but not limited to, Ctrough and $Ctrough_{ss}$, for PDR001, dabrafenib and trametinib.	
4) To evaluate the prevalence and incidence of immunogenicity	• ADA prevalence at baseline and ADA incidence on-treatment	
**Double-blind, randomized, placebo-controlled part (part 3)**		
1) To compare the anti-tumor activity of PDR001 in combination with dabrafenib and trametinib versus placebo plus dabrafenib and trametinib as measured by ORR, DCR, DOR per investigator's assessment according to RECIST 1.1	• ORR, DOR and DCR by investigator's assessment according to RECIST 1.1	
2) To evaluate safety and tolerability of PDR001 in combination with dabrafenib and trametinib versus placebo plus dabrafenib and trametinib	• Safety: Incidence and severity of AEs and SAEs, including changes in laboratory values, ECOG PS, vital signs, liver assessments and cardiac assessments.<br>• Tolerability: Dose interruptions, reductions, and dose intensity	
3) To evaluate patient reported outcomes of PDR001 in combination with dabrafenib and trametinib versus placebo plus dabrafenib and trametinib	• Change from baseline in EORTC QLQ-C30, EQ-5D, and FACT-M melanoma subscale<br>• Time to 10 point definitive deterioration in overall quality of life score from EORTC QLQ-30.	
4) To characterize PK of PDR001, dabrafenib and trametinib when administered in combination	• PK parameters such as but not limited to Ctrough and $Ctrough_{ss}$ for PDR001, dabrafenib and trametinib.	
5) To evaluate the prevalence and incidence of immunogenicity	• ADA prevalence at baseline and ADA incidence on-treatment	
6) To characterize the potential for PD-L1 expression to identify subjects with an enhanced response to PDR001 in combination with dabrafenib and trametinib versus placebo plus dabrafenib and trametinib	• PFS by investigator's assessment according to RECIST 1.1 and OS in subgroups defined by centrally assessed PD-L1 status.	

Objective	Endpoint	Analysis
**Exploratory**		Section 10.6.
1) To explore the association of immune biomarkers in the tumor microenvironment and blood derived markers with clinical response of PDR001 in combination with dabrafenib and trametinib	• Correlation between immune biomarkers in tissue samples (e.g., CD8+ cells, PD-L1 levels)/blood samples (e.g., ctDNA, cytokines) and clinical endpoints including PFS (using investigator's assessment) and OS.	
2) To explore changes in markers of the immune microenvironment in the tumor between baseline and post baseline samples and investigate the potential relationship with clinical response and mechanism of resistance to PDR001.	• Change from baseline of immune markers (RNA/protein or presence/absence and localization of specific immune cells) in pre- vs post dose tumor biopsies and association of marker changes with clinical endpoints including PFS (using investigator's assessment) and OS.	
3) To explore changes in blood derived markers upon treatment and investigate their potential relationship with clinical response and mechanism of resistance to PDR001.	• Change from baseline of blood derived markers (e.g., ctDNA: mutations in BRAF and other genes, plasma RNA levels of selected markers, cytokines) over the course of therapy and association of marker changes with clinical endpoints including PFS (using investigator's assessment) and OS.	
4) To explore the association of T-cell phenotype and clinical response of PDR001 in combination with dabrafenib and trametinib	• TCR sequencing and association of phenotype with clinical endpoints including PFS (using investigator's assessment) and OS.	
5) To characterize the potential for tumor mutation burden (TMB) alone and in combination with PD-L1, or additional markers to identify subjects with an enhanced response to PDR001 in combination with dabrafenib and trametinib versus placebo plus dabrafenib and trametinib	• PFS by investigator's assessment according to RECIST 1.1 and OS in subgroups defined by centrally assessed TMB status (determined by Next-Generation Sequencing) alone and in combination with PD-L1, or additional markers.	
6) To assess the relationship between PDR001 exposure and pharmacodynamics, safety, and efficacy	• PK parameters vs. pharmacodynamics, AEs, efficacy.	
7) To assess the impact of immunogenicity on efficacy, safety and pharmacokinetics of PDR001	• ADA vs. occurrence of AEs, efficacy endpoints, PK parameters	

Objective	Endpoint	Analysis
8) To explore baseline levels and changes from baseline in serum LDH and clinical response	• Correlation between baseline LDH level, as well as changes in LDH level, with clinical endpoints including PFS (based on RECIST 1.1 using investigator's assessment), OS, and circulating biomarkers.	

4 Study design

4.1 Description of study design

This study has been designed as a phase III, multi-center study consisting of 3 parts.

- Part 1: Safety run-in part (Figure 4-1)
- Part 2: Biomarker cohort (Figure 4-2)
- Part 3: Double-blind, randomized, placebo-controlled part (Figure 4-3)

Part 1: Safety run-in (Figure 4-1)

The safety run-in part will determine the recommended regimen of PDR001 in combination with dabrafenib and trametinib in previously untreated patients with *BRAF* V600 mutant unresectable or metastatic melanoma (AJCC Cancer Staging edition 7: Stage IIIC/IV).

The safety run-in part will consist of cohorts of approximately 6 newly enrolled subjects who will be treated at the specified dose regimen of PDR001 with fixed doses of dabrafenib 150 mg BID and trametinib 2 mg QD guided by the Bayesian Logistic Regression Model (BLRM) with EWOC criteria to determine the RP3R for part 3. Data from each cohort, including safety and PK data, will be reviewed by a Dose Level Review Team (DLRT) consisting of the Novartis team including at least one clinician, safety representative, and biostatistician, at least one investigator participating in the study who has enrolled subjects into the Safety run-in part of the study before the study can proceed to another dose level. The decision of the PDR001 regimen will be based on the BLRM model with EWOC criteria as well as clinical judgement of the DLRT. If dose DL1 is tolerated, then this dosing regimen will be the RP3R. If DL1 is not tolerated, then fixed dose combinations will be explored in two parallel cohorts (DL-1a and DL-1b), enrolling six additional subjects each provided that both dose levels are recommended based on results from BLRM with EWOC criteria. For the DL-1b cohort, subjects who do not tolerate and discontinue dabrafenib and/or trametinib during the first 4 weeks will be replaced.

The primary objective of the safety run-in part is to determine the RP3R. At least 6 subjects, and up to approximately 18, will be enrolled in this part of the study. The starting dose (DL1) for PDR001 in part 1 is 400 mg Q4W in combination with the approved doses for dabrafenib and trametinib. DLTs from 2 complete cycles of treatment (28 days per cycle; a total of 56 days) will be assessed. Provisional dose levels and guidelines for safety run-in part are detailed in Section 6.2.

Part 2: Biomarker cohort (Figure 4-2)

The open-label, biomarker cohort will enroll approximately 20 patients with previously untreated *BRAF* V600 mutant unresectable or metastatic melanoma (AJCC Cancer Staging edition 7: Stage IIIC/IV). Part 2 will open when the fourth subject in dose level 1 (DL1) of part 1 completes approximately 4 weeks of study treatment and less than 3 DLTs have been observed. It is possible for additional dosing regimens to be used based on emerging data from part 1, and recruitment may be suspended at any time.

Tumor tissue will be collected (1) at baseline, (2) after 2-3 weeks (C1D15 + 1 week window), (3) 8-12 weeks after initiation of PDR001 therapy and (4) at disease progression per RECIST

1.1 and/or at a subsequent disease progression per response criteria for immunotherapy if subject continues treatment beyond progression. In addition to these mandatory tissue sample collections, optional biopsies may be collected during the course of this biomarker study depending on investigator assessment (e.g., in case the biopsy needs to be collected from a responding/shrinking lesion prior to cycle 3). Blood samples will also be collected at several time points (mandatory collection; as specified in Table 7-17).

The samples will be used to characterize the kinetics of immune biomarkers and potential immune resistance mechanisms.

Part 3: Double-blind, randomized, placebo-controlled part (Figure 4-3)

Once the RP3R for the combination of PDR001 with dabrafenib and trametinib has been determined in part 1, the randomized, double-blind, placebo-controlled part 3 will open. Part 3 will enroll approximately 500 previously untreated patients with *BRAF* V600 mutant unresectable or metastatic melanoma (AJCC Cancer Staging edition 7: Stage IIIC/IV) and will be randomized in a 1:1 ratio to one of the following treatment arms:

- Arm 1: PDR001 in combination with dabrafenib and trametinib
- Arm 2: placebo in combination with dabrafenib and trametinib

The randomization will be stratified by LDH level (< 1 x upper limit of normal [ULN] vs ≥ 1 to < 2 × ULN vs ≥ 2 × ULN) in addition to ECOG performance status (0 vs 1 vs 2).

Figure 4-1 Part 1: Safety run-in overview of study design

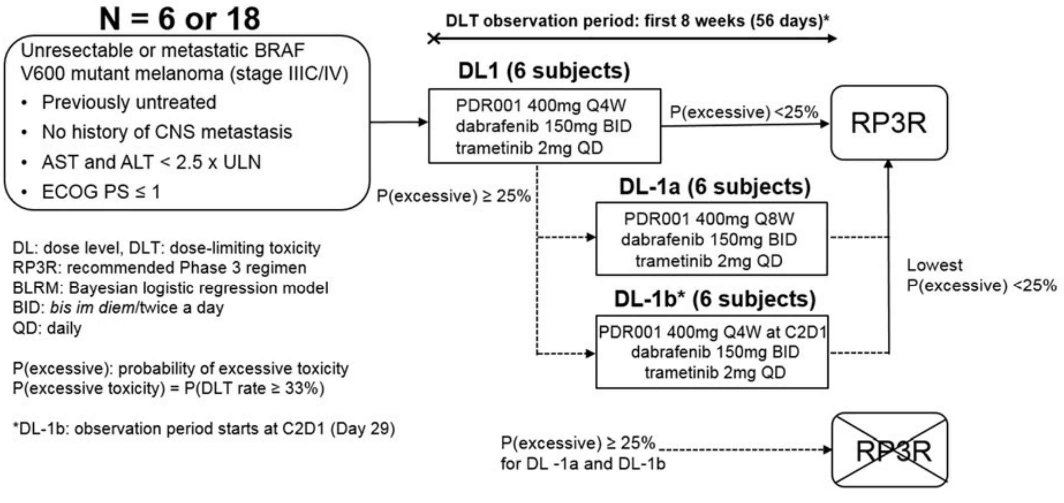

If the first 2 subjects in a cohort experience a DLT, further enrollment to that cohort will stop, the BLRM will be updated with this new information and re-evaluation of the available safety, pharmacokinetic, and pharmacodynamic data will occur.

Figure 4-2 Part 2: Biomarker cohort overview of study design

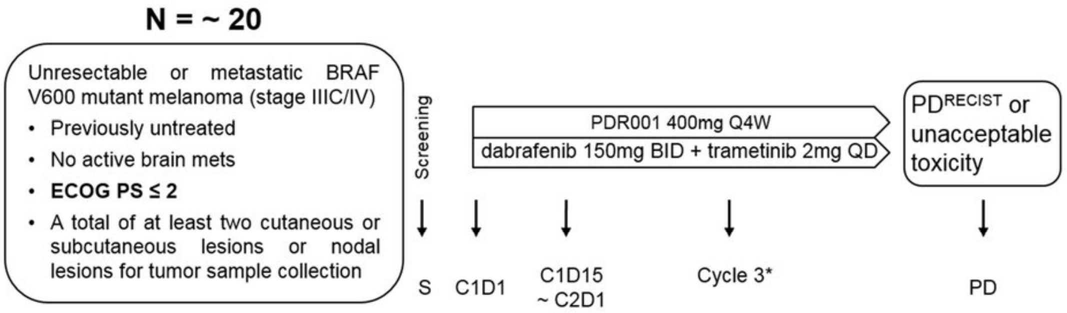

	S	C1D1	C1D15 ~ C2D1	Cycle 3*	PD
Tumor biopsy (FFPE) Central BRAFV600 mutation confirmation, assessment of IO biomarkers	X				
Blood/Plasma for biomarker analyses	X	X	X	X	X
Tumor biopsy (FFPE) for biomarker analyses			X	X	X
Optional Tumor biopsy on treatment	Anytime – depending on investigator assessment				

*Cycle 4 if DL-1b is determined to be the regimen

Figure 4-3 Part 3: Double blind, randomized, placebo-controlled part overview of study design

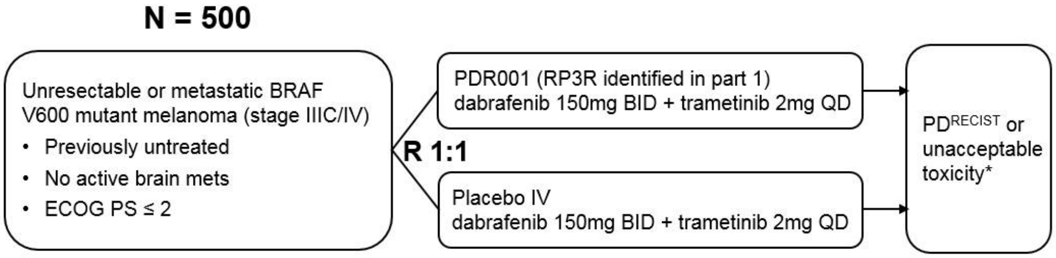

Randomization Stratification
- ECOG PS (0 vs 1 vs 2)
- LDH (< 1 x ULN vs ≥ 1 to < 2 x ULN vs ≥ 2 x ULN)

Primary endpoints: PFS^{RECIST}
Key Secondary: OS
Other Secondary: ORR, DOR, DCR, Safety, PROs, PK

*Treatment beyond PD^{Recist} is permitted if all of the following criteria are met: (1) informed consent for treatment beyond disease progression is provided by the subject, (2) the treatment will not delay an imminent intervention to prevent serious complications, (3) tolerance of study treatment, and (4) stable performance status

4.1.1 Screening phase

The screening phase begins once written informed consent is provided and ends after 28 days or when subject receives the first dose (part 1 and 2) or is randomized (part 3), whichever comes first.

Subjects must sign an informed consent form (ICF) prior to any study specific screening evaluations, within 28 days prior to the first dose of study treatment. Following completion of screening procedures and verifying subject eligibility based on assessments, the subject will be enrolled via the Interactive Response Technology (IRT) system. Laboratory results from the central laboratory will be used to determine subject's eligibility to the study. However, the site does not need to wait for the results of centrally-analyzed laboratory assessments when an immediate clinical decision needs to be made and in those cases locally unscheduled testing may be performed and used for eligibility assessments. Refer to Section 7.1 for additional details.

4.1.2 Treatment phase

The treatment phase begins when the first dose of study treatment is administered to a subject and ends at disease progression by RECIST 1.1, unacceptable toxicity, start of a new anti-neoplastic therapy, pregnancy, withdrawal of consent, lost to follow-up, physician's decision, lost to follow-up, death, or study is terminated by the sponsor.

Subjects may continue study treatment beyond disease progression by RECIST 1.1 until disease progression as per response criteria for immunotherapy (if criteria stipulated in Section 6.1.5.1 are fulfilled), unacceptable toxicity, start of new anti-neoplastic therapy, withdrawal of consent, physician's decision, lost to follow-up, death, or study is terminated by the sponsor. In case of continuation of study treatment beyond disease progression by RECIST 1.1, the subject will continue tumor assessments as outlined in Section 7.

Part 1: Safety run-in

In the first cohort, 6 subjects will be treated with a fixed dose combination (dose level) of dabrafenib 150 mg BID, trametinib 2 mg QD, and PDR001 400 mg Q4W (see Table 6-1). If dose DL1 is tolerated, then this dosing regimen will be the RP3R. If DL1 is not tolerated, then fixed dose combinations will be explored in two parallel cohorts (DL-1a and DL-1b), enrolling six additional subjects each. For the DL-1b cohort, subjects who do not tolerate and discontinue dabrafenib and/or trametinib during the first 4 weeks will be replaced.

For each cohort, decisions regarding the dosing regimen of PDR001 will be based upon the Investigators and Novartis study personnel after review of the available safety data, as well as the recommendations from the BLRM incorporating the EWOC principle.

Part 2: Biomarker cohort

Subjects in the biomarker cohort will receive PDR001 400 mg Q4W in combination with dabrafenib 150 mg BID and trametinib 2 mg QD. Based on emerging data from part 1, it is possible for additional dosing regimens to be used in this part of the study, and recruitment may be suspended at any time. The proposed biomarker sampling timing is optimized to address the modulation of the immune microenvironment and other biomarker endpoints over time with treatment.

Part 3: Double-blind, randomized, placebo-controlled part

Subjects will be randomly assigned (1:1) to receive either the RP3R identified in part 1 or placebo, dabrafenib 150 mg BID and trametinib 2 mg QD. Randomization will be stratified according to LDH level (< 1 x ULN vs. ≥1 to < 2 × ULN vs. ≥ 2 × ULN) and ECOG PS (0 vs. 1 vs. 2).

In the case that statistically significant interim or final OS results are demonstrated, the Sponsor may allow crossover only for subjects who were randomized to receive dabrafenib, trametinib, and placebo and meet the following criteria: (1) absence of progressive disease according RECIST 1.1, (2) adequate tolerance to study treatment, and (3) stable performance status.

4.1.3 End of treatment

Subjects should be scheduled for an End of Treatment (EOT) visit within 7 days after discontinuation of study treatment due to any reasons (Section 7.1).

4.1.4 Safety follow-up

All subjects will be followed for safety evaluations as outlined in detail in Section 7 and Section 3.

4.1.5 Efficacy follow-up

All subjects who discontinue study treatment without disease progression by RECIST 1.1 will continue tumor assessments as outlined in Section 7 until documented disease progression by RECIST 1.1, withdrawal of consent, lost to follow-up or death irrespective of start of new anti-neoplastic therapy.

All subjects who continue treatment beyond disease progression per RECIST 1.1 (see Section 6.1.5.1) will continue tumor assessments as outlined in Section 7 until confirmed progression per response criteria for immunotherapy, unacceptable toxicity, start of new anti-neoplastic therapy, withdrawal of consent, physician's decision, lost to follow-up, death or study is terminated by sponsor.

Refer to Section 7.2.1.2 for details on additional tumor assessments to perform in order to confirm response or allow for central confirmation.

4.2 Timing of interim analyses and design adaptations

An interim PFS analysis with possible early significance claim for efficacy will be conducted once approximately 260 PFS events have been observed.

Interim analyses of OS with early significance claim for efficacy will be conducted once approximately 260 PFS events (for the interim PFS analysis) have been observed and at the time of the final PFS analysis. Refer to Section 4.3, Section 10.4.2 and Section 10.7 for more details.

4.3 Definition of end of study

In the randomized part of this study, after the final analysis of PFS (based on investigator reported RECIST 1.1), the study will remain open provided the PFS demonstrates treatment

benefit. Patients still being followed on the study after the final PFS analysis time point will continue as per the schedule of assessments. If the primary PFS endpoint is met at the interim PFS analysis by showing a significant PFS benefit, the same guidelines will apply.

The final (inferential) OS analysis will be performed approximately when 245 deaths are observed (or earlier if specifically requested by health authorities) or when statistical significance is reached at the interim OS analysis, (see Section 10) and the final analysis of study data is conducted. All available data from all patients up to this cut-off date will be analyzed.

In the case that statistically significant interim or final OS results are demonstrated, the Sponsor may allow crossover for subjects randomized to the control/placebo arm who meet the following criteria: (1) absence of progressive disease according to RECIST 1.1, (2) adequate tolerance to study treatment, and (3) stable performance status. The study will be closed when all subjects have either died, withdrawn consent, are lost to follow-up, or have had at least 5 years of follow-up (measured from the date of randomization).

At the end of the study, every effort will be made in alignment with local regulations to continue provision of investigational treatment outside this study through an alternative setting to subjects who in the opinion of the investigator are still deriving clinical benefit.

If neither the interim nor the final PFS analysis demonstrate treatment benefit, the follow-up for OS will end.

4.4 Early study termination

The study can be terminated at any time for any reason by Novartis. Should this be necessary, the subject should be seen as soon as possible for the EOT visit and the same assessments should be performed as described in Section 7 for a discontinued or withdrawn subject. The investigator may be informed of additional procedures to be followed in order to ensure that adequate consideration is given to the protection of the subject's interests. The investigator will be responsible for informing IRBs and/or ECs of the early termination of the trial.

5 Population

5.1 Subject population

The investigator or designee must ensure that only subjects who meet all the following inclusion and none of the exclusion criteria are offered treatment in the study.

5.2 Inclusion criteria

Subjects eligible for inclusion in this study have to meet **all** of the following criteria:

5.2.1 Specific inclusion criteria for safety run-in (part 1)

1. ECOG performance status ≤ 1
2. Aspartate transaminase (AST) < 2.5× ULN and Alanine transaminase (ALT) < 2.5× ULN

5.2.2 Specific inclusion criteria for biomarker cohort (part 2)

3. ECOG performance status ≤ 2
4. A total of at least two cutaneous or subcutaneous lesions or nodal lesions for tumor sample collection. Lesions situated in a previously irradiated area, or an area subject to other loco-regional therapy (e.g., intralesional injections) cannot be considered for sample collection.

5.2.3 Specific inclusion criteria for double-blind, randomized, placebo-controlled part (part 3)

5. ECOG performance status ≤ 2

5.2.4 Inclusion criteria for all subjects

6. ≥ 18 years of at the time of informed consent
7. Written informed consent must be obtained prior to any screening procedures
8. Histologically confirmed, unresectable or metastatic melanoma (stage IIIC or IV)
9. *BRAF* V600 mutation positive melanoma as assessed locally, or if local *BRAF* testing is unavailable, at a Novartis designated central reference laboratory (Section 7.1.2)
10. Measurable disease per RECIST 1.1.
11. Treatment-related toxicities (except alopecia) must ≤ Grade 1 at the time of randomization according to CTCAE version 4.03 (NCI 2009)
12. An adequate amount of tumor tissue (archived tumor tissue, or new biopsy if archived tissue is not available) must be available at the time of enrollment for central validation of *BRAF* V600 mutation and biomarker assessments (see Section 7.1.2 and Section 7.2.4 for testing details and requirements)
13. Subject has adequate bone marrow and organ function as defined by the following laboratory values without continuous supportive treatment (such as blood transfusion, coagulation factors and/or platelet infusion, or red/white blood cell growth factor administration) as assessed by laboratory for eligibility:
    - Hematological
        - Absolute neutrophil count ≥ 1.5 × 10^9/L
        - Platelet count ≥ 100 × 10^9/L
        - Hemoglobin ≥ 9 g/dL
    - Coagulation
        - PT/INR and PTT ≤ 1.5 x ULN. Subjects receiving anticoagulation treatment may be allowed to participate with INR established within the therapeutic range prior to randomization
    - Renal
        - Serum creatinine < 1.5 mg/dL
    - Hepatic
        - Total bilirubin ≤ 1.5 x ULN except for subjects with Gilbert's syndrome who may only be included if the total bilirubin is ≤ 3.0 × ULN or direct bilirubin ≤ 1.5 × ULN

- Aspartate transaminase (AST) < 2.5 × ULN, except for subjects with liver metastasis, who are only included if the AST is < 5 × ULN
- Alanine transaminase (ALT) < 2.5 × ULN, except for subjects with liver metastasis, who are only included if the ALT is < 5 × ULN
- Albumin ≥ 2.5 g/dL

14. Left ventricular ejection fraction (LVEF) ≥ lower limit of institutional normal (LLN) as assessed by echocardiogram (ECHO) or multigated acquisition (MUGA) scan
15. Able to swallow and retain oral medication and must not have any clinically significant gastrointestinal abnormalities that may alter absorption such as malabsorption syndrome or major resection of the stomach or bowels
16. Subjects must avoid consumption of grapefruit, Seville oranges or products containing the juice of each during the entire study and preferably 7 days before the first dose of study medications, due to potential CYP3A4 interaction with the study medications. Orange juice is allowed.

5.3 Exclusion criteria

Subjects eligible for this study must not meet any of the following criteria:

5.3.1 Specific exclusion criteria for safety run-in (part 1)

1. Any history of CNS metastases

5.3.2 Specific exclusion criteria for the biomarker cohort (part 2) and the double-blind, randomized, placebo-controlled part (part 3)

2. Clinically active cerebral melanoma metastasis. Subjects with up to three cerebral metastases are eligible, if all lesions are stable and have been definitively treated with stereotactic radiation therapy, surgery or gamma knife therapy with no evidence of disease progression prior to enrollment as assessed by two consecutive assessments ≥ 6 weeks apart and have not required steroids for at least ≥ 4 weeks prior to enrollment (physiological doses of corticosteroids are allowed).

5.3.3 Exclusion criteria for all subjects

3. Subjects with uveal or mucosal melanoma
4. Prior systemic anti-cancer treatment (e.g., checkpoint inhibitors, targeted therapy [e.g., *BRAF* and/or MEK inhibitors], chemotherapy, biologic therapy, tumor vaccine therapy, or any systemic investigational treatment) for unresectable or metastatic melanoma.
5. Prior loco-regional treatment with intralesional therapy (e.g., talimogene laherparepvec) for unresectable or metastatic melanoma in the last 6 month prior to start of study treatment.
6. Prior neoadjuvant and/or adjuvant therapy for melanoma completed less than 6 months prior to start of study treatment (e.g., targeted therapy, immunotherapy [e.g., interferon], biochemotherapy, tumor vaccine).
7. Radiation therapy ≤ 4 weeks prior to start of study treatment (palliative radiotherapy to bone lesions allowed ≤ 2 weeks prior to start of study treatment).

8. Major surgery, open biopsy, or significant traumatic injury ≤ 2 weeks prior to start of study treatment. Minor surgical procedures should be completed 7 days prior to start of study treatment.
9. Active, known or suspected autoimmune disease or a documented history of autoimmune disease, including ulcerative colitis and Crohn's disease (Subjects with vitiligo, type I diabetes mellitus, residual hypothyroidism due to autoimmune condition only requiring hormone replacement, psoriasis not requiring systemic treatment, or conditions not expected to recur in the absence of an external trigger are permitted to enroll).
10. Systemic chronic steroid therapy (≥ 10mg/day prednisone or equivalent) or any immunosuppressive therapy 7 days prior to planned date for first dose of study treatment. Topical, inhaled, nasal and ophthalmic steroids are allowed.
11. Current pneumonitis or interstitial lung disease.
12. History of organ transplant requiring use of immunosuppressive medication.
13. Taken an investigational drug ≤ 28 days or ≤ 5 half-lives (minimum 14 days) prior to start of study treatment, whichever is shorter.
14. History of severe hypersensitivity reactions to other monoclonal antibodies, which in the opinion of the investigator may pose an increased risk of serious infusion reaction.
15. Current use of a prohibited medication.
16. Malignant disease, other than that being treated in this study. Exceptions to this exclusion include the following: malignancies that were treated curatively and have not recurred within 2 years prior to study treatment; completely resected basal cell and squamous cell skin cancers and any completely resected carcinoma *in situ*.
17. Active infection requiring systemic antibiotic therapy within 2 weeks prior to start of study treatment.
18. Known history of testing positive for Human Immunodeficiency Virus (HIV) infection. For Germany only: testing positive for HIV during screening using a local test.
19. Subjects with active Hepatitis B infection (HbsAg positive) will be excluded.
    Note: Subjects with antecedent of Hepatitis B (anti-HBc positive, HbsAg and HBV-DNA negative) are eligible.
20. Subjects with positive test for hepatitis C ribonucleic acid (HCV RNA)
    Note: Subjects in whom HCV infection resolved spontaneously (positive HCV antibodies without detectable HCV-RNA) or those that achieved a sustained virological response after antiviral treatment and show absence of detectable HCV RNA ≥ 6 months (with the use of IFN-free regimes) or ≥ 12 months (with the use of IFN-based regimes) after cessation of antiviral treatment are eligible
21. Any medical condition that would, in the investigator's judgment, prevent the subject's participation in the clinical study due to safety concerns, compliance with clinical study procedures or interpretation of study results.
22. Use of any live vaccines against infectious diseases within 4 weeks of initiation of study treatment.
23. Uncorrectable electrolyte abnormalities (e.g., hypokalemia, hypomagnesemia, hypocalcemia), or long QT syndrome.

24. Known immediate or delayed hypersensitivity reaction or idiosyncrasy to drugs chemically related to the study treatments, their excipients, and/or dimethyl sulfoxide (DMSO).
25. Pregnant or nursing (lactating) women confirmed by a positive hCG laboratory test within 72 hours prior to initiating study treatment. Note: Low levels of hCG may also be considered a tumor marker, therefore if low hCG levels are detected, another blood sample at least 4 days later must be taken to assess the kinetics of the increase and transvaginal ultrasound must be performed to rule out pregnancy.
26. A history or current evidence/risk of retinal vein occlusion (RVO) or central serous retinopathy including:
    - Presence of predisposing factors to RVO or central serous retinopathy (e.g., uncontrolled glaucoma or ocular hypertension, uncontrolled hypertension, uncontrolled diabetes mellitus, or a history of hyperviscosity or hypercoagulability syndromes); or
    - Visible retinal pathology as assessed by ophthalmic examination that is considered a risk factor for RVO or central serous retinopathy such as:
    - Evidence of new optic disc cupping;
    - Evidence of new visual field defects on automated perimetry;
    - Intraocular pressure >21 mmHg as measured by tonometry.
27. Cardiac or cardiac repolarization abnormality, including any of the following:
    - History or current diagnosis of cardiac disease indicating significant risk of safety for subjects participating in the study such as uncontrolled or significant cardiac disease, including any of the following:
    - Recent (within last 6 months) myocardial infarction (MI)
    - Unstable angina (within last 6 months),
    - Uncontrolled congestive heart failure (CHF)
    - Clinically significant (symptomatic) cardiac arrhythmias (e.g., sustained ventricular tachycardia, and clinically significant second or third degree atrioventricular [AV] block without a pacemaker).
28. Women of child-bearing potential, defined as all women physiologically capable of becoming pregnant, **unless** they are using highly effective methods of contraception during dosing and for 150-days after stopping treatment with PDR001/placebo, 30-days after dabrafenib or 120-days after trametinib, whichever is longer. Highly effective contraception methods include:
    a. Total abstinence (when this is in line with the preferred and usual lifestyle of the subject). Periodic abstinence (e.g., calendar, ovulation, symptothermal, post-ovulation methods) and withdrawal are not acceptable methods of contraception.
    b. Female sterilization (have had surgical bilateral oophorectomy with or without hysterectomy), total hysterectomy, or tubal ligation at least six weeks before taking study treatment. In case of oophorectomy alone, only when the reproductive status of the woman has been confirmed by follow up hormone level assessment.

c. Male sterilization (at least 6 months prior to screening). The vasectomized male partner should be the sole partner for that subject.
d. Placement of a non-hormonal intrauterine device (IUD) or intrauterine system (IUS) with a documented failure rate of less than 1% per year.

Notes:

- Double-barrier contraception: condom and occlusive cap (diaphragm or cervical/vault caps) with a vaginal spermicidal agent (foam/gel/cream/suppository) are not considered highly effective methods of contraception.
- Hormonal-based methods (e.g., oral contraceptives) are not considered as highly effective methods of contraception due to potential drug-drug interactions with dabrafenib.
- Women are considered post-menopausal and not of child bearing potential if they have had 12 months of natural (spontaneous) amenorrhea with an appropriate clinical profile (i.e. age appropriate, history of vasomotor symptoms) or have had surgical bilateral oophorectomy (with or without hysterectomy), total hysterectomy, or tubal ligation at least six weeks ago. In the case of oophorectomy alone, only when the reproductive status of the woman has been confirmed by follow up hormone level assessment is she considered not of child bearing potential.

29. Sexually active males unless they use a condom during intercourse while on treatment and for 150 days after stopping treatment with PDR001/placebo, 30 days after dabrafenib or 120 days after trametinib, whichever is longer and should not father a child in this period. A condom is required to be used by vasectomized men as well during intercourse in order to prevent delivery of the drug via semen.

6 Treatment

6.1 Study treatment

For this study, the investigational drug is PDR001, and the study treatment is PDR001 or placebo in combination with dabrafenib 150 mg BID and trametinib 2 mg QD.

PDR001 will be supplied in vial in liquid or powder for solution for infusion pharmaceutical form. PDR001 (liquid or reconstituted powder) will be diluted in dextrose 5% in water (D5W). Due to incompatibility, 0.9% sodium chloride solution must not be used.

Placebo will be a D5W for infusion supplied by the site.

Infusion must take place in a facility with appropriate resuscitation equipment available at the bedside and a physician readily available during the period of drug administration.

Trametinib will be provided as 0.5 mg and 2 mg tablets, and dabrafenib as 50 mg and 75 mg capsules for oral administration.

All dosages prescribed and dispensed to subjects and all dose changes during the study must be recorded on the Dosage Administration Record (DAR) eCRF.

6.1.1 Dosing regimen

Table 6-1 Dose and treatment schedule

Study treatments	Pharmaceutical form and route of administration	Strength	Frequency and/or Regimen	Dose
PDR001	Powder for solution for infusion[a]	100 mg	Q4W or Q8W	400 mg
PDR001	Liquid [b] in vial for i.v. infusion	100 mg	Q4W or Q8W	400 mg
Dabrafenib	Capsules for oral use	50 mg, 75 mg	BID	150 mg BID (300 mg/day
Trametinib	Tablets for oral use	0.5 mg, 2 mg	QD	2 mg/day

a. PDR001 powder for solution for infusion formulation will be used in the safety run-in (part 1) and biomarker cohort (part 2).
b. PDR001 liquid formulation may be used for the double-blind, randomized part (part 3) once available.

6.1.1.1 Dosing regimen for Part 1: Safety run-in part

The safety run-in part of the study will determine the regimen of PDR001 in combination with dabrafenib and trametinib for the part 3 of the study. The starting dose for DL1 will be PDR001 400 mg given as an intravenous infusion every 4 weeks, dabrafenib 150 mg orally twice daily and trametinib 2 mg orally once daily.

Table 6-2 Part 1 (safety run-in part): Provisional dose levels

Dose level	PDR001 infusion	Dabrafenib capsules	Trametinib tablets
Dose level 1 (DL1) [a]	400 mg Q4W	150 mg BID (300 mg/day)	2 mg QD
Dose level 1a (DL-1a)	400 mg Q8W	150 mg BID (300 mg/day)	2 mg QD
Dose level 1b (DL-1b) [b]	400 mg Q4W (starting at C2D1)	150 mg BID (300 mg/day)	2 mg QD

a. Starting dose level. It is possible for additional and/or intermediate dose levels to be added during the course of the study based on emerging data.
b. 4 weeks of dabrafenib and trametinib only period followed by addition of 400 mg PDR001 Q4W starting at C2D1 (Day 29).

6.1.1.2 Dosing regimen for Part 2: Biomarker cohort

Table 6-3 Part 2 (biomarker cohort): Dose and treatment schedule

Study treatments	Pharmaceutical form and route of administration	Dose	Frequency and/or regimen
PDR001	Powder for solution for infusion in vial for i.v. infusion	400 mg	Q4W
Dabrafenib	Capsules for oral use	150 mg	BID (300 mg/day)
Trametinib	Tablets for oral use	2 mg	QD

It is possible for dosing regimens to be added during the course of the study based on emerging data.

6.1.1.3 Dosing regimen for Part 3: Randomized part

The double-blind, randomized, placebo-controlled part of the study will enroll approximately 500 patients in a 1:1 ratio to the following treatment arms:

- Arm 1: PDR001 in combination with dabrafenib and trametinib
- Arm 2: placebo in combination with dabrafenib and trametinib

The study treatment phase begins on C1D1 with the first administration of study treatment. Each cycle in both treatment arms is defined as 28 days.

Table 6-4 Part 3 (randomized part): Dose and treatment schedule

Study treatments	Pharmaceutical form and route of administration	Dose	Frequency and/or regimen
PDR001	Powder for solution for infusion or liquid [a] in vial for i.v. infusion	400 mg	To be determined in part 1 (safety run-in)
Dabrafenib	Capsules for oral use	150 mg	BID (300 mg/day)
Trametinib	Tablets for oral use	2 mg	QD

[a.] PDR001 liquid formulation may be used for the double blind, randomized part (part 3) once available.

6.1.1.4 PDR001 or placebo administration

PDR001 or placebo will be administered via intravenous infusion over 30 minutes (up to 2 hours, if clinically indicated) once every 4 or 8 weeks as determined in part 1. Subjects should be provided instructions to notify study personnel if symptoms of infusion reaction occur after any PDR001 infusion. The dose may be interrupted up to 12 weeks. The safety assessments as outline in Section 7 should be performed.

Study visits with scheduled PK sampling should be scheduled in the morning so that a pre-dose PK blood sample can be collected. Scheduling these visits in the morning is especially important for Cycle 1 Day 1 and Cycle 3 Day 1 during which a post-dose (end-of-infusion) PK sample is to be collected.

6.1.1.5 Dabrafenib and trametinib administration

Dabrafenib and trametinib will be provided by the sponsor to the investigative site or supplied locally as commercially available and labeled accordingly to comply with legal requirements of each country.

Dabrafenib and trametinib should be taken as follows:

- Dabrafenib will be administered orally twice daily (BID) for Days 1-28 of a 28-day cycle.
- Trametinib will be administered orally once daily (QD) for Days 1-28 of a 28-day cycle.
- Subjects should be instructed to take the dabrafenib and trametinib concurrently in the morning, at approximately the same time every day. The second (evening) dose of dabrafenib (150 mg) should be administered approximately 12 (± 4) hours apart from the first dose (morning) of dabrafenib.
- For Part 1, on days of coadministration with PDR001, dabrafenib and trametinib should be taken approximately 90 minutes after completion of PDR001 administration.
- For Parts 2 and 3, on days of coadministration with PDR001, dabrafenib and trametinib should be taken prior to the start of infusion with PDR001.
- Dabrafenib and trametinib should be taken with approximately 120-240 mL of water under fasting conditions, at least 1 hour before or 2 hours after a meal.

- Subjects should be instructed to swallow whole capsules of dabrafenib and not chew or open them.
- If a subject vomits after taking study drug, the subject should be instructed not to retake the dose and wait for the next scheduled dose. The occurrence and frequency of any vomiting during a treatment cycle must be noted in the AE section of the eCRF.
- If a subject misses a dose, he/she should be instructed not to double the next regularly scheduled dose. However, subject may take the missed dose immediately if the next scheduled dose is at least 6 hours later for dabrafenib and 12 hours later for trametinib. Subject may then take the next dose at the scheduled time.
- Subjects must avoid consumption of grapefruit, grapefruit hybrids, pomelos, star-fruit, Seville oranges or products containing the juice of each during the entire study and preferably 7 days before the first dose of study medications, due to potential CYP3A4 interaction with the study medications.
  - Orange juice is allowed.
  - No herbal supplements are permitted.
  - Multivitamins are permitted.

6.1.2 Ancillary treatments

PDR001

Subjects should not receive pre-medication to prevent infusion reaction before the first infusion of study treatment. If a subject experiences an infusion reaction, he/she may receive pre-medication on subsequent dosing days. The pre-medication should be chosen per institutional standard of care, at the discretion of the investigator.

Acute allergic reactions should be treated as needed per institutional standard of care. In the event of anaphylactic/anaphylactoid reactions, this includes any therapy necessary to restore normal cardiopulmonary status. If a subject experiences a Grade 3 anaphylactic/anaphylactoid reaction, the subject will be discontinued from the study.

Guidelines on management of infusion reactions are provided in Table 6-20.

The CTCAE v4.03 (...//ctep.cancer.gov) category of “Infusion related reaction” should be used to describe study treatment related infusion reactions, unless the investigator considers another category, such as “allergic reaction,” “anaphylaxis,” or “cytokine release syndrome” more appropriate in a specific situation.

Dabrafenib and trametinib

Not applicable.

6.1.3 Rescue medication

Not applicable.

6.1.4 Guideline for continuation of treatment

Guidelines on management of common PDR001, dabrafenib or trametinib associated toxicities and dose modification instructions are provided in Section 6.3.

6.1.5 Treatment duration

For all parts, study treatment will continue until the subject experiences one of the following: disease progression per investigator's assessment by RECIST 1.1 (and not meeting the criteria in Section 6.1.5.1), unacceptable toxicity, pregnancy, treatment is discontinued at the discretion of the investigator or the subject, start of a new anti-neoplastic therapy, withdrawal of consent, lost to follow-up, death, or study is terminated by the sponsor.

6.1.5.1 Treatment beyond disease progression per RECIST 1.1

All subjects (including placebo arm in the randomized, double blind part 3) will be permitted to continue study treatment beyond disease progression per RECIST 1.1 provided they meet all the following criteria:

1. Informed consent for treatment beyond disease progression is provided by the subject
2. The continuation of treatment beyond progression will not delay an imminent intervention to prevent serious complications of disease progression (e.g., CNS metastases)
3. Subject exhibits adequate tolerance to study treatment
4. Subject remains with stable performance status

Subjects who meet the above criteria should continue study treatment and continue all study procedures as outlined in Section 7. Additional informed consent needs to be obtained for treatment beyond progression. The reasons for the subject continuing treatment beyond progression will be documented in the eCRF.

An additional tumor assessment must be performed to confirm response (CR or PR) no less than 4 weeks after the criteria for response are first met.

In case of clinical deterioration or suspicion of disease progression, a follow-up imaging assessment should be performed promptly rather than waiting for the next scheduled assessment. Subjects can be treated beyond progression until confirmed progression per response criteria for immunotherapy, unacceptable toxicity, start of new anti-neoplastic therapy, withdrawal of consent, physician's decision, lost to follow-up, death or study is terminated by sponsor.

Subsequent to disease progression per RECIST 1.1, at least two additional tumor assessments that are at least 4 weeks apart must be performed no less than 4 weeks after criteria for PD per RECIST 1.1 are first met. Tumor assessments after start of new anti-neoplastic therapy are recommended however not required.

6.2 Determination of recommended regimen for the randomized part of the study

6.2.1 Starting dose rationale

The starting dose of PDR001 is 400 mg IV Q4W in combination with 150 mg dabrafenib BID and 2 mg trametinib QD. Please refer to Section 2.3 for more details on the dose rationale.

6.2.2 Provisional dose levels

Table 6-5 describes the dose levels that may be evaluated during this trial. For the safety run-in period, the BLRM model will consider DLT data from the first 8 weeks of triple combination for each tested dose level.

Table 6-5 Provisional dose levels

Dose level (DL)	PDR001 IV	Dabrafenib oral	Trametinib oral	DLT observation period
DL1 [a]	400mg Q4W	150mg BID (300 mg/day)	2mg QD	8 wks
DL-1a	400mg Q8W	150mg BID (300 mg/day)	2mg QD	8 wks
DL-1b [b]	400mg Q4W starting at C2D1	150mg BID (300 mg/day)	2mg QD	8 wks Starting at C2D1

[a.] Starting dose level. It is possible for additional and/or intermediate dose levels to be added during the course of the study based on emerging data.
[b.] Dabrafenib and trametinib will be administered for 4 weeks initially, followed by addition of PDR001 400 mg Q4W at C2D1 (day 29)

6.2.3 Guidelines for determination of recommended regimen for part 3

For the purposes of determining the recommended dosing regimen for PDR001 in combination with dabrafenib and trametinib to be used in part 3, each cohort will consist of approximately 6 newly enrolled subjects who will be treated at the specified dose level. The first cohort will be treated with the starting dose of PDR001 400 mg Q4W, dabrafenib 150 mg BID and trametinib 2 mg QD.

To be considered ‘evaluable’ for dose regimen decisions, subjects must have received at least one dose of PDR001, received at least 50% of planned doses of dabrafenib and trametinib, and had safety assessments for a minimum of 8 weeks (56 days), or have a DLT within the first 8 weeks (56 days) of study treatment. Dose regimen decisions will occur when the cohort of subjects has met these criteria.

Dose regimen decisions will be made by Investigators and Novartis study personnel. Decisions will be based on a synthesis of all relevant data available from all dose levels evaluated in the ongoing study including safety information, DLTs, all CTCAE v4.03 ≥ Grade 2 toxicity data during the first 8 weeks (56 days) of triple combination treatment, and pharmacokinetic data from evaluable subjects. The recommended dose for the next cohort of subjects will be guided by the BLRM with EWOC principle.

The adaptive Bayesian methodology provides an estimate of DLT rates for all dose levels of the combination of PDR001, dabrafenib, and trametinib and incorporates all DLT information at all dose levels for this estimation.

If the first 2 subjects in a cohort experience a DLT, further enrollment to that cohort will stop, the BLRM will be updated with this new information, and re-evaluation of the available safety, and pharmacokinetic data will occur. By incorporating information gained at the preceding dose levels, additional subjects may be enrolled into the current dose level only if the combination still meets the EWOC criteria. Alternatively, if recruitment to the same dose level may not resume, a new cohort of subjects may be recruited to a lower dose combination, as agreed by Investigators and Novartis personnel, and if the BLRM predicts that the risk for this lower dose

combination to exceed toxicity remains below 25% (EWOC). Re-escalation may then occur if data in subsequent cohorts supports this (EWOC criteria are satisfied) and Investigators and Novartis personnel agree.

The recommended dose regimen is identified when the following conditions are met:

1. at least 6 subjects have been treated at this dose and observed for 8 weeks (56 days)
2. this dose satisfies the EWOC criteria
3. the selected regimen is recommended either per the model or by review of all clinical data by Novartis and Investigators in a teleconference.

6.2.3.1 Implementation of dose escalation decisions

Not applicable.

6.2.3.2 Intra-patient dose escalation

Not applicable.

6.2.4 Definition of dose limiting toxicities in the safety-run in part (part 1)

A DLT is defined as (1) an adverse event or abnormal laboratory value assessed as unrelated to disease, disease progression, inter-current illness, or concomitant medications that occurs within the 8 weeks (56 days) of treatment with PDR001 in combination with dabrafenib and trametinib and (2) meets any of the predefined criteria in Table 6-6. NCI CTCAE v4.03 (http://ctep.cancer.gov) will be used for grading of DLTs and adverse events in this protocol.

Table 6-6 Criteria for defining dose-limiting toxicities

**TOXICITY**	**DLT CRITERIA** (NCI CTCAE v4.03 will be used for grading)
Hematological	• Grade 4 anemia<br>• Grade 4 neutropenia (for > 7 consecutive days) or Grade 4 febrile neutropenia<br>• Grade 4 thrombocytopenia (of any duration).<br>• Grade 3 thrombocytopenia with clinically significant bleeding regardless of duration or requirement of platelet transfusion
Hepato-biliary	• Grade 4 bilirubin elevation<br>• For subjects with normal baseline AST and ALT values:<br>◦ AST or ALT > 8.0 × ULN<br>• For subjects with normal baseline AST and ALT and normal baseline bilirubin value:<br>◦ AST or ALT > 3.0 × ULN combined with total bilirubin > 2.0 × ULN without evidence of cholestasis<br>• For subjects with abnormal baseline AST or ALT or abnormal baseline bilirubin value:<br>◦ [AST or ALT > 2 × baseline AND > 3.0 × ULN] OR [AST or ALT > 8.0 x ULN], combined with [TBIL > 2 × baseline AND > 2.0 × ULN] without evidence of cholestasis
Gastrointestinal	• Nausea and vomiting ≥ Grade 3 for > 3 days despite optimal anti-emetic therapy.<br>• ≥ Grade 3 diarrhea for > 5 days despite optimal antidiarrheal treatment (which could include steroids).
Pancreas	• Symptomatic serum amylase or lipase elevation, medical intervention required (Pancreatitis Grade 3 or higher)

TOXICITY	DLT CRITERIA (NCI CTCAE v4.03 will be used for grading)
Hypertension	• ≥ Grade 3 hypertension related to the study medication if it persists > 7 days despite optimal anti-hypertensive treatment<br>• Grade 4 hypertension of any duration
Cardiac	• ≥ Grade 3 cardiac event that is symptomatic or requires medical intervention
Pneumonitis	• Grade 2 pneumonitis if it persists > 7 days despite treatment with corticosteroids.<br>• ≥ Grade 3 pneumonitis of any duration
Immune-related toxicities (except pneumonitis)	• Grade 3 immune-related toxicities that persist > 14 days with same severity despite treatment with corticosteroids.<br>• Grade 4 immune related toxicities of any duration<br>• ≥ Grade 3 infusion related reaction
Other Adverse Events	• Other clinically significant adverse events:<br>• ≥ Grade 3 adverse events that has not been previously identified for dabrafenib/trametinib and/or PDR001.<br>• ≥ Grade 3 AEs that are known to occur with dabrafenib, trametinib and/or PDR001, but cannot be controlled using the recommended product-specific management guidelines or leads to <50% of planned exposure of study medications<br>• Other clinically significant toxicities, including a single event or multiple occurrences of the same event that lead to a dosing delay of > 12 weeks should be considered to be DLTs by the Investigators and Novartis, even if not Grade 3 or higher.
Events which will NOT be considered as DLT for the purpose of this protocol:<br>• Clinically insignificant laboratory values ≤ Grade 2. For laboratory values ≥ Grade 3, the maximum allowable time limit for correction of electrolyte abnormalities to ≤ Grade 1 is 72 hr.<br>• Cutaneous squamous cell carcinoma (CuSCC), basal cell carcinoma, and new primary melanoma which are manageable with surgical excision alone	

The investigator must notify Novartis within less than 24 hours of any unexpected CTCAE v4.03 ≥ Grade 3 AEs or laboratory abnormalities.

Dose modification during DLT observation period

If a subject experiences a DLT, during the DLT observation period, then treatment with dabrafenib, trametinib and PDR001 must be interrupted and the subject may be discontinued from the study depending on the severity of the DLT. However, following resolution of the DLT to CTCAE v4.03 Grade 1 or baseline value, the subject may continue to receive study treatment at a reduced dose level, if appropriate, at the discretion of the investigator.

6.3 Dose modifications and reductions

Adverse events of special interest (AESI) include AEs of a potential irAE that are associated with PDR001 treatment. An irAE may be experienced by subjects treated with PDR001 due to its mechanism of action and predicted based on the reported experience with other immunotherapies that have a similar mechanism of action. Investigators must be vigilant and carefully identify AEs that may be suggestive of potential irAEs as their appearance may be sub-clinical and early diagnosis is critical for its adequate management and resolution.

An irAE is typically low grade and self-limited, often occurring after multiple doses, and most frequently involving the GI tract (diarrhea/colitis), skin (rashes), liver (hepatitis), lung (pneumonitis), kidneys (nephritis) and endocrine systems (a variety of endocrinopathies) and

rarely CNS (encephalitis). Serological, immunological and histological assessments should be performed as deemed appropriate by the investigator, to verify the potential immune-related nature of the AE, and exclude a neoplastic, infectious or metabolic origin of the AE.

Severe grade or persistent lower grade irAEs typically require interrupting or permanently discontinuing treatment and administration of systemic steroids or other non-corticosteroid immunosuppressive medication when systemic steroids are not effective. Early recognition of irAEs and initiation of treatment are critical to reduce the risk of complications, since the majority of irAEs are reversible with the use of steroids and other immune suppressants.

In some cases, it may be unclear to determine if an adverse event is immune related or not, thus the following dose reduction guidelines should be followed.

The following sections address the specific instructions for mandatory dose modifications and recommended management for AEs considered suspected to be related to study treatment. For subjects who do not tolerate the protocol-specified dosing schedule, dose interruptions or modifications are mandated in order to allow subjects to continue study treatment.

No dose reductions are allowed for PDR001. Dose interruption for PDR001/placebo includes delaying or withholding the treatment for any reason as well as an interruption of treatment during an infusion. All dose interruptions and the reason for the dose interruption must be recorded on the Dosage Administration Record eCRF. Three dose reductions for dabrafenib and two dose reductions for trametinib are permitted. If more dose reductions of dabrafenib and trametinib are required, the subject must be permanently discontinued from that specific study treatment. Dose modification steps for PDR001 or placebo, dabrafenib and trametinib in Table 6-7 must be followed for the AEs presented in Section 6.3.

Table 6-8 provides a list of AEs of special interest and relevant sections where detailed dose modification and management requirements for relevant AEs and/or irAEs can be found. All AEs are to be graded according to NCI-CTCAE v4.03, unless otherwise specified. Investigators should refer to the current PDR001 Investigator’s Brochure and the dabrafenib and trametinib local prescribing information for additional information regarding the background of each drug and the management of other AEs or potential safety-related issues not specifically mentioned in this protocol.

Table 6-7 Dose modification steps for PDR001/placebo, dabrafenib and trametinib

**Dose modification for PDR001 or placebo [a]**		
	Starting dose level	Dose level - 1
PDR001 or placebo	400 mg Q4W<br>400 mg Q8W (only if DL-1a is determined to be the dose regimen)	Not applicable

**Dose reduction for dabrafenib [a, b]**				
	Starting dose level	Dose level - 1	Dose level - 2	Dose level - 3
Dabrafenib	150 mg BID<br>(300 mg/day)	100 mg BID<br>(200 mg/day)	75 mg BID<br>(150 mg/day)	50 mg BID<br>(100 mg/day)
**Dose reduction for trametinib [a, b]**				
	Starting dose level	Dose level - 1	Dose level - 2	Dose level - 3
Trametinib	2 mg QD	1.5 mg QD	1.0 mg QD	-

Q4W: once in every 4 weeks; BID: twice daily; QD: once daily
[a.] Dose modification should be based on the worst toxicity demonstrated at the last dose.
[b.] Dose reduction below 50 mg BID for dabrafenib and 1.0 mg QD for trametinib are not allowed.

Table 6-8 Reference of AEs and toxicity management guidelines

Adverse Events	PDR001	dabrafenib	trametinib	Guideline
Diarrhea/colitis	X	X	X	Section 6.3.3.1 Table 6-9.
Abnormal liver enzyme test	X	X	X	Section 6.3.3.2 Table 6-10.
Skin rash	X	X	X	Section 6.3.3.3 Table 6-11.
Serious skin reaction	X	X	X	Section 6.3.3.4
Nephritis	X	X	X	Section 6.3.3.5 Table 6-12
Pneumonitis	X	X	X	Section 6.3.3.6 Table 6-13
Encephalitis	X	X	X	Section 6.3.3.7 Table 6-14
Endocrine events	X	X	X	Section 6.3.3.8 Table 6-15
Hand foot skin reaction	X	X	X	Section 6.3.3.9 Table 6-16
Neutropenia and thrombocytopenia	X	X	X	Section 6.3.3.10 Table 6-17
Adverse events including abnormal laboratory related AEs	X	X	X	Section 6.3.3.11 Table 6-18
Asymptomatic amylase and/or lipase elevation	X	X	X	Section 6.3.3.11 Table 6-18
Others	X	X	X	Section 6.3.3.12 Table 6-19
Infusion reaction and cytokine release syndrome	X			Section 6.3.4 Table 6-20
General		X	X	Section 6.3.5 Table 6-21
LVEF		X	X	Section 6.3.5.1 Table 6-22
Hypertension		X	X	Section 6.3.5.2 Table 6-23
New malignancies		X	X	Section 6.3.5.3.
Pyrexia syndrome		X	X	Section 6.3.5.4 Table 6-24
Visual changes		X (Uveitis)	X (RVO)	Section 6.3.5.5 Table 6-25
RPED			X	Section 6.3.5.5 Table 6-26
Hyperglycemia		X		Section 6.3.5.6
Hemorrhage		X	X	Section 6.3.5.7
Thromboembolic events			X	Section 6.3.5.8

"X" indicates study treatment (s) that may need to be modified.

6.3.1 General dose modification instructions for PDR001 or placebo

No dose reductions are allowed for PDR001. Dose interruption for PDR001/placebo includes delaying or withholding the treatment for any reason as well as an interruption of treatment

during an infusion. Subjects with adverse events suspected to be related to PDR001 or placebo including those of potential immune-mediated etiology (irAE) (see definition in Section 6.3) must be permanently discontinued from PDR001 or placebo if (1) the AE doesn't recover to ≤ Grade 1 or baseline within 12 weeks and/or (2) the dose of steroids (for the management of irAE) remains > 10 mg/day prednisone or equivalent for >12 weeks.

The 12 week timeframe will begin from the time the irAE reaches a grade that leads to PDR001/placebo interruption.

Subjects who discontinue the study for a study related adverse event or an abnormal laboratory value must be followed as described in Section 7.

6.3.2 General dose modification instructions for dabrafenib and trametinib:

If treatment related toxicities occur that are specific to combination treatment of dabrafenib and trametinib, then both treatments should be simultaneously dose reduced, interrupted or discontinued with the exceptions shown below:

Exception where dose modification is necessary for **only dabrafenib**:

- Uveitis (Table 6-25)

Exception where dose modifications are necessary for **only trametinib**:

- Retinal vein occlusions (RVO) (Table 6-25) and retinal pigment epithelial detachment (RPED) (Table 6-26)
- Left ventricular ejection fraction (LVEF) reduction (Table 6-22)

If a subject's dose of dabrafenib and trametinib has been reduced per the dose modification instructions, re-escalation of the subject's dose is recommended provided the following criteria are met:

- a period of 4 weeks of treatment has passed since restarting dosing at the lower dose level and there is no recurrence of the AE
- the subject is deriving clinical benefit

For dabrafenib and/or trametinib related adverse events: if following an interruption of dabrafenib and trametinib, an AE doesn't recover to ≤ Grade 1 or baseline within 4 weeks, dabrafenib and trametinib must be discontinued.

6.3.3 Dose modification and management guideline for adverse events common to PDR001, dabrafenib and trametinib

Adverse events that have been reported with PDR001, dabrafenib or trametinib include, but are not limited to diarrhea/colitis, abnormal liver enzymes, skin rash, nephritis, pneumonitis, encephalitis, and endocrinopathies. It is possible that these and /or other adverse events may be exacerbated when PDR001, dabrafenib and trametinib are combined. Therefore, it is important to note that the management guidelines for the combination of PDR001, dabrafenib and trametinib may differ for a given AE from the management guidelines for each agent or combination treatment of dabrafenib and trametinib.

6.3.3.1 Mandatory dose modifications and recommended clinical management guidelines for diarrhea/colitis

Guidelines for dose modification and management for diarrhea and colitis considered to be related to study treatment by the investigator are provided in Table 6-9.

Table 6-9 Mandatory dose modifications and recommended clinical management guidelines for diarrhea/colitis

CTCAE v4.03	Recommended adverse event management guidelines	Mandatory dose modification requirements
Grade 1 diarrhea (increase of <4 stools per day over baseline; mild increase in ostomy output compared to baseline)<br>OR<br>Grade 1 colitis (asymptomatic; clinical or diagnostic observations only; intervention not indicated)<br>OR<br>Grade 2 diarrhea (increase of 4 - 6 stools per day over baseline; moderate increase in ostomy output compared to baseline)<br>OR<br>Grade 2 colitis (abdominal pain; mucus or blood in stool)	• Diet: stop all lactose containing products; eat small meals, BRAT diet (banana, rice, apples, toast)<br>• Hydration: 8-10 large glasses of clear liquids per day (e.g., Gatorade or broth)<br>• Loperamide: initially 4 mg, followed by 2 mg every four hours or after every unformed stool; maximum 16 mg/day. Continue until diarrhea free for 12 hours;<br>• Diarrhea > 24hr: loperamide 2 mg every two hours; maximum 16 mg/day. Consider adding oral antibiotics.<br>• Diarrhea > 48hr: loperamide 2 mg every two hours; maximum 16 mg/day. Consider other second-line therapies for diarrhea (e.g., octreotide, oral diphenoxylate, and oral antibiotics)<br>• If Grade 2 and no improvement in 5 days consider oral steroids<br>• If Grade 2 diarrhea persists >1 week consider gastroenterologist consultation and endoscopy to evaluate for colitis<br>• If Grade 2 persists for 5 days and worsening of symptoms or diffuse ulcerations and bleeding seen on endoscopy, commence oral steroids at a dose 0.5 to 1 mg/kg/day prednisone equivalents and continue until symptoms improve to Grade 1. If no improvement occurs, manage as per Grade 3. Steroids can be tapered as per Section 6.3.4.1.<br>• Lower GI endoscopy and biopsy should be considered and may assist in determining the duration of steroid taper based on the evidence of macroscopic and microscopic inflammation.	• Continue PDR001/placebo, dabrafenib and trametinib at the same dose level.<br>• If diarrhea is Grade 2, despite loperamide at 2 mg every two hours for > 48hr:<br>• Interrupt PDR001/placebo, dabrafenib and trametinib until ≤ Grade 1, and then reinstate PDR001, dabrafenib and trametinib at the same dose level.

CTCAE v4.03	Recommended adverse event management guidelines	Mandatory dose modification requirements
Grade 3 diarrhea (increase of ≥7 stools per day over baseline; incontinence; hospitalization indicated; severe increase in ostomy output compared to baseline; limiting self-care ADL)<br>OR<br>Grade 3 colitis (severe abdominal pain; change in bowel habits; medical intervention indicated; peritoneal signs)	• Clinical evaluation and hospitalization mandatory; rule out bowel perforation and intravenous hydration.<br>• Consider consultation with gastroenterologist and confirmation biopsy with lower GI endoscopy.<br>• In addition to symptomatic treatment (diet, hydration, loperamide, antibiotics if indicated); initiate immediate treatment with intravenous steroids (methylprednisolone 1mg/kg/day) followed by high dose oral steroids (1 to 2 mg/kg/day prednisone equivalents)<br>• When symptoms improve to ≤ Grade 1, steroid taper should be started and continued over no less than 4 weeks as per Section 6.3.4.1.<br>• Taper over 6 to 8 weeks in subjects with diffuse and severe ulceration and/or bleeding.<br>• If no improvement in 2-3 days: consider initiating infliximab 5 mg/kg and continue steroids. (Infliximab is contraindicated in subjects with sepsis or a perforation). Upon symptomatic relief initiate a prolonged steroid taper over 45 to 60 days.<br>• If symptoms worsen during steroid reduction, initiate a re-tapering of steroids starting at a higher dose followed by a more prolonged taper and administer infliximab.<br>• If symptoms persist despite the above treatment, a surgical consult should be obtained.	**1st occurrence**<br>• Interrupt PDR001/placebo, dabrafenib and trametinib until ≤ Grade 1, and then reinstate PDR001 treatment (after appropriate steroid tapering) and reduce dabrafenib and trametinib to the next lower dose level per Table 6-7. Re-escalation of the subject's dose is recommended if criteria in Section 6.3.2 are met.<br>• AE resolution to ≤ Grade 1 or baseline must occur within a period of 12 weeks since a grade 3 event has been identified, otherwise PDR001 must be permanently discontinued.<br>**2nd occurrence**<br>• Permanently discontinue PDR001/placebo.<br>• Interrupt dabrafenib and trametinib until ≤ Grade 1, and then reduce dabrafenib and trametinib to the next lower dose per Table 6-7. Re-escalation of the subject's dose is recommended if criteria in Section 6.3.2 are met.<br>**3rd occurrence**<br>• Permanently discontinue dabrafenib and trametinib.
Grade 4: Life-threatening consequences; urgent intervention indicated	• Same as Grade 3	• Permanently discontinue PDR001/placebo, dabrafenib and trametinib.

6.3.3.2 Mandatory dose modifications and recommended clinical management guidelines for abnormal liver enzyme test

Guidelines for dose modification and management of abnormal liver enzyme functions considered to be related to study treatment by the investigator are provided in Table 6-10.

In addition to the instructions below, subjects with normal baseline transaminases and bilirubin who experience sudden elevations even within Grade 1, should be monitored more closely.

Table 6-10 Mandatory dose modifications and recommended clinical management guidelines for abnormal liver enzyme test

Abnormal liver enzyme tests/hepatitis		
**Grade**	**Recommended adverse event management guidelines**	**Mandatory dose modification requirements**
Grade 2: AST or ALT >3× ULN to ≤ 5× ULN and/or bilirubin > 1.5× ULN to ≤ 3× ULN	• Monitor hepatic laboratory tests more frequently(every 2-3 days) until returned to baseline values	• Interrupt PDR001/placebo, dabrafenib and trametinib until ≤ Grade 1, and then reinstate PDR001/placebo, dabrafenib and trametinib at the same dose level.
Grade 3 or 4: AST or ALT > 5× ULN and/or bilirubin > 3× ULN	• Monitor hepatic laboratory tests more frequently (every 2-3 days) until return to baseline values.<br>• Consider appropriate consultation* with hepatologist and liver biopsy to establish etiology of hepatic injury, if necessary<br>• If after 2-3 days new liver assessment shows worsening of laboratory test consider to initiate treatment with high dose steroids (1 to 2 mg/kg/day prednisone or equivalents)<br>• Add prophylactic antibiotics for opportunistic infections<br>• Start steroid taper as outlined in Section 6.3.4.1 if symptoms improve to Grade ≤1<br>• If serum transaminase levels do not decrease 48 hours after initiation of systemic steroids, oral mycophenolate mofetil 500 mg every 12 hours may be given.<br>• Infliximab is not recommended due to its potential for hepatotoxicity	• Permanently discontinue PDR001/placebo only if causality is suspected to be related to PDR001/placebo **<br>• If lab results show worsening, follow the management and dose modification (instructions below)<br>**1st occurrence**<br>• Interrupt dabrafenib and trametinib until recovery to ≤ Grade 1 or baseline<br>◦ Reduce dabrafenib and trametinib to the next lower dose level per Table 6-7. Re-escalation of the subject's dose is recommended if criteria in Section 6.3.2 are met.<br>◦ If no recovery to ≤ Grade 2 within 10 weeks, permanently discontinue dabrafenib and trametinib.<br>**2nd occurrence**<br>• Interrupt dabrafenib and trametinib until ≤ Grade 1.<br>◦ Reduce dabrafenib and trametinib to the next lower dose level per Table 6-7. Re-escalation of the subject's dose is recommended if criteria in Section 6.3.2 are met.<br>**3rd occurrence**<br>• Permanently discontinue dabrafenib and trametinib

*Rule out viral hepatitis and other potential causes of liver injury

**For patients with liver metastasis who begin treatment with Grade 2 AST or ALT, if AST or ALT increase by ≥ 50% relative to baseline and last for at least 1 week then the patient should be discontinued

Note: For additional information on follow-up of potential drug induced liver injury cases, refer to Section 6.3.6.2.

6.3.3.3 Mandatory dose modifications and recommended clinical management guidelines for skin rash

Guidelines for dose modification and management of skin rash considered to be related to study treatment by the investigator are provided in Table 6-11.

Table 6-11 Mandatory dose modifications and recommended clinical management guidelines for rash

**Rash Events (NCI-CTCAE v4.03)**		
**Grade**	**Recommended adverse event management guidelines**	**Mandatory dose modification requirements**
Grade 1: Rash covering < 10% body surface area	• Initiate prophylactic and symptomatic treatment measures.<br>• Consider use of topical corticosteroids or urea containing creams in combination with oral antipruritics or moderate strength topical steroid (hydrocortisone 2.5% cream or fluticasone propionate 0.5% cream)<br>• Reassess after 2 weeks	• Continue PDR001/placebo, dabrafenib and trametinib at the same dose.
Grade 2: 10-30% of body surface area	• If tolerable, as per Grade 1<br>• If intolerable, initiate systemic steroids (0.5 to 1 mg/kg/day prednisone or equivalents)<br>• If symptoms persist or recur consider skin biopsy.	• If tolerable, continue PDR001/placebo, dabrafenib and trametinib at the same dose.<br>• If intolerable:<br>**1st occurrence**<br>• Interrupt PDR001/placebo, dabrafenib and trametinib until ≤ Grade 1, and then reinstate PDR001 treatment (after appropriate steroid tapering) and reduce dabrafenib and trametinib to the next dose level per Table 6-7. Re-escalation of the subject's dose is recommended if criteria in Section 6.3.2 are met.<br>• AE resolution to ≤ Grade 1 or baseline must occur within a period of 12 weeks since a Grade 3 event has been identified, otherwise PDR001 must be permanently discontinued.
Grade 3: More than 30% of body surface area	• Obtain a skin biopsy and dermatology consult.<br>• Initiate therapy with high dose steroids (1 to 2 mg/kg/d prednisone or equivalents)	**1st occurrence**<br>• Interrupt PDR001/placebo, dabrafenib and trametinib until ≤ Grade 1, and then reinstate PDR001 treatment (after appropriate steroid tapering), and reduce dabrafenib and trametinib to the next dose level per Table 6-7. Re-escalation of the subject's dose is recommended if criteria in Section 6.3.2 are met.<br>• AE resolution to ≤ Grade 1 or baseline must occur within a

Rash Events (NCI-CTCAE v4.03)		
**Grade**	**Recommended adverse event management guidelines**	**Mandatory dose modification requirements**
		period of 12 weeks since a grade 3 event has been identified, otherwise PDR001 must be permanently discontinued.<br>**2nd occurrence**<br>• Permanently discontinue PDR001/placebo.<br>• Interrupt dabrafenib and trametinib until ≤ Grade 1 or baseline. Once recovered, reduce dabrafenib and trametinib to the next dose level per Table 6-7. Re-escalation of the subject's dose is recommended if criteria in Section 6.3.2 are met.<br>**3rd occurrence**<br>• Interrupt dabrafenib and trametinib until ≤ Grade 1 or baseline. Once recovered, reduce dabrafenib and trametinib to the next dose level per Table 6-7. Re-escalation of the subject's dose is recommended if criteria in Section 6.3.2 are met.<br>**4th occurrence**<br>• Permanently discontinue PDR001/placebo, dabrafenib and trametinib.
Grade 4: Life-threatening	• Same as Grade 3	• Permanently discontinue PDR001/placebo, dabrafenib and trametinib.

6.3.3.4 Mandatory dose modifications and recommended clinical management of serious skin reactions

Withhold trametinib for intolerable or severe skin toxicity. Resume trametinib at reduced doses in subjects with improvement or recovery from skin toxicity within 3 weeks. In cases of Stevens-Johnson syndrome and toxic epidermal necrolysis, permanently discontinue dabrafenib, trametinib and PDR001 and institute supportive care as per institutional guidelines.

6.3.3.5 Mandatory dose modifications and recommended clinical management guidelines for nephritis

Guidelines for does modification and management of nephritis considered to be related to study treatment by the investigator are provided in Table 6-12.

Table 6-12 Mandatory dose modifications and recommended clinical management guidelines for renal function alterations

NCI-CTCAE v4.03		
**Grade**	**Recommended adverse event management guidelines**	**Mandatory dose modification requirements**
Grade 1 (Creatinine > ULN to ≤ 1.5× ULN)	• Monitor creatinine weekly<br>• If creatinine return to baseline resume routine creatinine monitoring per protocol<br>• Promote hydration and cessation of nephrotoxic drugs.	• Continue PDR001/placebo, dabrafenib and trametinib at the same dose.
Grade 2 (Creatinine > 1.5× to ≤ 3× ULN	• Monitor creatinine every 2 to 3 days<br>• Initiate 0.5 to 1 mg/kg/day prednisone equivalents<br>• If worsening or no improvement: increase to 1 to 2 mg/kg/day prednisone equivalents<br>• Consult with specialist and consider renal biopsy<br>• Promote hydration and cessation of nephrotoxic drugs.	**1st occurrence**<br>• Interrupt PDR001/placebo, dabrafenib and trametinib until ≤ Grade 1 or baseline and then reinstate PDR001 treatment and dabrafenib and trametinib at the same dose.<br>• AE resolution to ≤ Grade 1 must occur within a period of 12 weeks since Grade 2 event has been identified. Otherwise, PDR001/placebo must be permanently discontinued.<br>**2nd occurrence:**<br>• Permanently discontinue PDR001/placebo.<br>• Interrupt dabrafenib and trametinib until ≤ Grade 1 or baseline. Once recovered, reduce dabrafenib and trametinib to the next dose level per Table 6-7. Re-escalation of the subject's dose is recommended if criteria in Section 6.3.2 are met.<br>**3rd occurrence:**<br>• Interrupt dabrafenib and trametinib until ≤ Grade 1 or baseline. Once recovered, reduce dabrafenib and trametinib to the next dose level per Table 6-7. Re-escalation of the subject's dose is recommended if criteria in Section 6.3.2 are met.<br>**4th occurrence**<br>• Permanently discontinue dabrafenib and trametinib.
Grade 3 (Creatinine > 3.0 to < 6×ULN)	• Monitor creatinine every 1 to 2 days.<br>• Start 1 to 2 mg/kg/day prednisone equivalents<br>• Consult with nephrologist.<br>• Promote hydration and cessation of nephrotoxic drugs.	**1st occurrence:**<br>• Interrupt PDR001/placebo, dabrafenib and trametinib until ≤ Grade 1 or baseline and then reinstate PDR001 then reduce dabrafenib and trametinib to the next lower dose per Table 6-7. Re-escalation of the subject's

NCI-CTCAE v4.03		
**Grade**	**Recommended adverse event management guidelines**	**Mandatory dose modification requirements**
		dose is recommended if criteria in Section 6.3.2 are met.<br>• AE resolution to ≤ Grade 1 or baseline must occur within a period of 12 weeks since the Grade 3 event has been identified, otherwise PDR001/placebo must be permanently discontinued<br>**2nd occurrence:**<br>• Permanently discontinue PDR001/placebo.<br>• Interrupt dabrafenib and trametinib until ≤ Grade 1 or baseline and then reduce dabrafenib and trametinib to the next lower dose per Table 6-7. Re-escalation of the subject's dose is recommended if criteria in Section 6.3.2 are met.<br>**3rd occurrence:**<br>• Interrupt dabrafenib and trametinib until ≤ Grade 1 or baseline and then reduce dabrafenib and trametinib to the next lower dose per Table 6-7. Re-escalation of the subject's dose is recommended if criteria in Section 6.3.2 are met.<br>**4th occurrence:**<br>• Permanently discontinue dabrafenib and trametinib.
Grade 4: Creatinine > 6× ULN	• Monitor creatinine daily.<br>• Initiate steroids with 1 to 2 mg/kg/day prednisone equivalents<br>• Consult with specialist and recommend renal biopsy.<br>• Promote hydration and cessation of nephrotoxic drugs.	• Permanently discontinue PDR001/placebo, dabrafenib and trametinib.

6.3.3.6 Mandatory dose modifications and recommended clinical management guidelines for pneumonitis

Guidelines for does modification and management of pneumonitis considered to be related to study treatment by the investigator are provided in Table 6-13.

Table 6-13 Mandatory dose modifications and recommended clinical management guidelines for pneumonitis

Pneumonitis (NCI-CTCAE v4.03)		
**Grade**	**Recommended adverse event management guidelines**	**Mandatory dose modification requirements**
Grade 1: Radiographic changes only-Asymptomatic	• CT scan (high-resolution with lung windows) recommended, with serial imaging to monitor for resolution or progression- re-image at least every 3 weeks<br>• Monitor for symptoms every 2-3 days - Clinical evaluation and laboratory work-up for infection<br>• Monitoring of oxygenation via pulse oximetry recommended<br>• Consultation of pulmonologist recommended.	• Continue dabrafenib and trametinib at the same dose.<br>• Interrupt PDR001/placebo, until recovery to baseline.<br>◦ Once recovered, reinitiate PDR001/placebo at the same schedule.<br>◦ If worsens, treat as Grade 2 or 3-4.<br>◦ AE resolution to ≤ Grade 1 or baseline must occur within a period of 12 weeks since Grade 1 event has been identified, otherwise PDR001 /placebo must be permanently discontinued
Grade 2: Symptomatic-medical intervention indicated; limits instrumental ADLs	• CT scan (high-resolution with lung windows)<br>• Monitor symptoms daily, consider hospitalization<br>• Clinical evaluation and laboratory work up for infection<br>• Consult pulmonologist<br>• Pulmonary function tests - if normal at baseline, repeat every 8 weeks<br>• Bronchoscopy with biopsy and/or BAL recommended<br>• Symptomatic therapy including corticosteroids if clinically indicated (systemic corticosteroids at a dose of 1 to 2 mg/kg/day prednisone or equivalent as clinically indicated).	**1st occurrence:**<br>• Interrupt PDR001/placebo, and trametinib until recovery to ≤ Grade 1 or baseline. Dabrafenib may continue at the same dose.<br>◦ Once recovered, reinstate PDR001/placebo at same schedule and reduce trametinib to the next lower dose per Table 6-7. Re-escalation of the subject's dose is recommended if criteria in Section 6.3.2 are met.<br>◦ If no recovery to ≤ Grade 1 within 4 weeks, permanently discontinue trametinib. Dabrafenib may continue.<br>◦ AE resolution to ≤ Grade 1 or baseline must occur within a period of 12 weeks since Grade 2 event has been identified, otherwise.PDR001/placebo must be permanently discontinued.<br>◦ If worsens treat as Grade 3 or 4.<br>**2nd occurrence:**<br>• Permanently discontinue PDR001/placebo.

Pneumonitis (NCI-CTCAE v4.03)		
**Grade**	**Recommended adverse event management guidelines**	**Mandatory dose modification requirements**
Grade 3: Severe symptoms; limits self-care ADLs; oxygen indicated	• CT scan (high-resolution with lung windows)<br>• Clinical evaluation and laboratory work-up for infection<br>• Consult pulmonologist<br>• Pulmonary function tests-if < normal, repeat every 8 weeks until ≥ normal<br>• Bronchoscopy with biopsy and/or BAL if possible<br>• Treat with intravenous steroids (methylprednisolone 125 mg) as indicated. When symptoms improve to ≤ Grade 1, a high dose oral steroid (prednisone 1 to 2 mg/kg once per day or dexamethasone 4 mg every 4 hours).<br>• If IV steroids followed by high dose oral steroids does not reduce initial symptoms within 48 to 72 hours, consider non-corticosteroid immunosuppressive medication (e.g., infliximab, cyclophosphamide, IVIG or mycophenolate mofetil).	• Permanently discontinue PDR001/placebo.<br>• Interrupt trametinib until recovery to Grade ≤1 or baseline. Dabrafenib may continue.<br>◦ Once recovered, trametinib may be restarted at the next lower dose per Table 6-7. Re-escalation of the subject's dose is recommended if criteria in Section 6.3.2 are met.<br>◦ If no recovery to Grade ≤ 1 or baseline within 4 weeks, permanently discontinue trametinib. Dabrafenib may continue.
Grade 4: Life- threatening respiratory compromise	• Same as Grade 3	• Same as Grade 3

6.3.3.7 Mandatory dose modifications and recommended clinical management guidelines for encephalitis

Guidelines for does modification and management of encephalitis considered to be related to study treatment by the investigator are provided in Table 6-14.

Table 6-14 Mandatory dose modifications and recommended clinical management guidelines for encephalitis

Encephalitis		
**Grade**	**Recommended adverse event management guidelines**	**Mandatory dose modification requirements**
Grade 1: Asymptomatic or mild symptoms		• Continue PDR001/placebo, dabrafenib and trametinib at the same dose.

**Encephalitis**		
**Grade**	**Recommended adverse event management guidelines**	**Mandatory dose modification requirements**
Grade 2: New onset of moderate symptoms, limiting instrumental ADL	• Consider consulting neurology.<br>• Treat with 0.5 to 1 mg/kg/day prednisone equivalents	**1st occurrence:**<br>• Interrupt PDR001/placebo, until recovery to ≤ Grade 1 or baseline.<br>• Once recovered, restart PDR001/placebo at the same dose.<br>• AE resolution to ≤ Grade 1 or baseline must occur within a period of 12 weeks since Grade 2 event has been identified, otherwise PDR001/placebo must be permanently discontinued.<br>• If it worsens, treat as Grade 3 – 4.<br>**2nd occurrence:**<br>• Permanently discontinue PDR001/placebo.<br>• Continue dabrafenib and trametinib at the same dose.<br>**3rd occurrence:**<br>• Permanently discontinue dabrafenib and trametinib.
Grade 3 or 4: New onset of severe symptoms, limiting self-care ADL, life-threatening	• Recommend consultation with neurologist.<br>• Treat with 1 to 2 mg/kg/day prednisone equivalents.	**1st occurrence:**<br>• Permanently discontinue PDR001/placebo.<br>• Interrupt dabrafenib and trametinib until recovery to ≤ Grade 1 or baseline. Once recovered, reduce dabrafenib and trametinib at the next dose level per Table 6-7.Re-escalation of the subject's dose is recommended if criteria in Section 6.3.2 are met.<br>**2nd occurrence:**<br>• Permanently discontinue dabrafenib and trametinib.

6.3.3.8 Mandatory dose modifications and recommended clinical management guidelines for endocrine events

Guidelines for does modification and management of endocrine events considered to be related to study treatment by the investigator are provided in Table 6-15.

Table 6-15 Mandatory dose modifications and recommended clinical management guidelines for endocrine events

Endocrine events (NCI-CTCAE v4.03)		
**Grade**	**Recommended adverse event management guidelines**	**Mandatory dose modification requirements**
Asymptomatic, intervention not indicated (hyperthyroidism or hypothyroidism)	• If TSH <0.5x LLN, or TSH >2x ULN, or consistently out of range in 2 subsequent measurements, include free T4 at subsequent cycles as clinically indicated<br>• Consider endocrinologist consult<br>• If hypophysitis is considered, pituitary gland imaging should be considered (MRIs with gadolinium and selective cuts of the pituitary can show enlargement or heterogeneity and confirm the diagnosis)<br>• Repeat labs in 1 to 3 weeks/MRI in 1 month if laboratory abnormalities persist but normal lab/pituitary scan	• Continue PDR001/placebo, dabrafenib and trametinib at the same dose.
Symptomatic endocrinopathy (e.g., hypophysitis, adrenal insufficiency, hypothyroidism, hyperthyroidism)	• Consider Endocrinology consultation<br>• Rule out infection and sepsis with appropriate cultures and imaging<br>• Treat with an initial dose of methylprednisolone 1 to 2 mg/kg intravenously followed by oral prednisone 1 to 2 mg/kg per day.<br>• Replacement of appropriate hormones may be required as the steroid dose is tapered<br>• Hypophysitis with clinically significant adrenal insufficiency and hypotension, dehydration, and electrolyte abnormalities (such as hyponatremia and hyperkalemia) constitutes adrenal crisis<br>• Consider hospitalization and intravenous methylprednisolone should be initiated.	**1st occurrence:**<br>• Interrupt PDR001/placebo, until symptomatic recovery to mild or no symptoms.<br>◦ Once recovered, restart PDR001/placebo.<br>◦ AE resolution to mild or no symptoms must occur within a period of 12 weeks since symptomatic endocrinopathy event has been identified, otherwise PDR001/placebo must be permanently discontinued.<br>**2nd occurrence:**<br>• Permanently discontinue PDR001/placebo and may continue dabrafenib and trametinib at the same dose.

6.3.3.9 Mandatory dose modifications and recommended clinical management guidelines for hand-foot reaction (palmar-plantar erythrodysesthesia)

In general, management of hand-foot skin reactions include:

- Prevention/prophylaxis: promote sunscreen use and avoidance of unnecessary sun exposure, use alcohol-free emollient creams, topical steroids and antibiotics as needed.
- Pruritic lesions: cool compresses and oral antihistamines
- Fissuring lesions: Monsel’s solution, silver nitrate or zinc oxide cream
- Desquamation: thick emollients and mild soap

- Paronychia: antiseptic bath, local potent corticosteroids, antibiotics, surgery as needed
- Infected lesions: topical or systemic antibiotics
- Measures for PPES should include:
  - Lifestyle modification: avoidance of hot water, traumatic activity, constrictive footwear, or excessive friction on the skin and the use of thick cotton socks and gloves, and shoes with padded insoles
  - Symptomatic treatments: apply moisturizing creams frequently, topical keratolytics (e.g., urea 20-40% cream, salicylic acid 6%, tazarotene 0.1% cream, fluorouracil 5% cream), clobetasol propionate 0.05% ointment for erythematous areas, topical lidocaine 2%, and / or systemic pain medication such as nonsteroidal anti-inflammatory drugs, codeine, and pregabalin for pain.

Guidelines for does modification for hand foot skin reaction considered to be related to study treatment by the investigator are provided in Table 6-16.

Table 6-16 Mandatory dose modifications and recommended clinical management guidelines for hand foot skin reaction (palmar-plantar erythrodysesthesia)

**Hand foot skin reaction**		
**Grade**	**Recommended adverse event management guidelines**	**Mandatory dose modification requirements**
Grade 1: Numbness, dysesthesia, paresthesia, tingling, painless swelling, erythema or discomfort of the hands or feet which does not disrupt the subject's normal activities	• Recommend topical therapy for symptomatic relief.	• Continue PDR001/placebo, dabrafenib and trametinib at the same dose.
Grade 2: Painful erythema and swelling of the hands or feet and/or discomfort affecting the subject's normal activities	• Recommend topical therapy for symptomatic relief	• Continue treatment and if no improvement within 7 days, see below.<br>• No improvement within 7 days or 2nd or 3rd occurrence:<br>• Interrupt PDR001/placebo, dabrafenib and trametinib until toxicity resolves to ≤ Grade 1 or baseline.<br>• Reduce dabrafenib and trametinib at the next lower dose per Table 6-7. Re-escalation of the subject's dose is recommended if criteria in Section 6.3.2 are met.<br>**4th occurrence:**<br>• Discontinue PDR001/placebo, dabrafenib and trametinib.

Hand foot skin reaction		
**Grade**	**Recommended adverse event management guidelines**	**Mandatory dose modification requirements**
Grade 3: Moist desquamation, ulceration, blistering or severe pain of the hands or feet, or severe discomfort that causes the subject to be unable to work or perform activities of daily living	• Recommend topical therapy for symptomatic relief	**1st occurrence:**<br>• Interrupt PDR001/placebo, dabrafenib and trametinib until toxicity resolves to ≤ Grade 1 or baseline.<br>• Once recovered, reinstate PDR001/placebo, and reduce dabrafenib and trametinib to the next dose level per Table 6-7. Re-escalation of the subject's dose is recommended if criteria in Section 6.3.2 are met.<br>**2nd occurrence:**<br>• Interrupt PDR001/placebo, dabrafenib and trametinib until toxicity recovers to ≤ Grade 1 or baseline.<br>• Once recovered, reinstate PDR001/placebo, and reduce dabrafenib and trametinib to the next dose level per Table 6-7. Re-escalation of the subject's dose is recommended if criteria in Section 6.3.2 are met.<br>**3rd occurrence:**<br>• Discontinue PDR001/placebo, dabrafenib and trametinib.

6.3.3.10 Mandatory dose modifications and recommended clinical management guidelines for neutropenia and thrombocytopenia

Guidelines for dose modification for neutropenia and thrombocytopenia suspected to be related to study treatment by the investigator are provided in Table 6-17.

Table 6-17 Mandatory dose modifications and recommended clinical management guidelines for neutropenia and thrombocytopenia

Neutropenia and thrombocytopenia suspected AEs (NCI-CTCAE v4.03)		
**Grade**	**Recommended adverse event management guidelines**	**Mandatory dose modification requirements**
**Neutropenia**		
Grade 1, 2	NA	• Continue PDR001/placebo, dabrafenib and trametinib at the same dose.
Grade 3, 4	• Monitor blood test more frequently (every 7 days for Grade 3, and 3-5 days for Grade 4).	**1st occurrence:**<br>• Interrupt PDR001/placebo, dabrafenib and trametinib until toxicity recovers to ≤ Grade 2 or baseline.<br>• Restart PDR001/placebo at the same regimen, and reduce dabrafenib and trametinib at the next dose level. Re-escalation of the subject's dose is

Neutropenia and thrombocytopenia suspected AEs (NCI-CTCAE v4.03)		
**Grade**	**Recommended adverse event management guidelines**	**Mandatory dose modification requirements**
		recommended if criteria in Section 6.3.2 are met.<br>**2nd occurrence:**<br>• Interrupt PDR001/placebo, dabrafenib and trametinib until toxicity recovers to ≤ Grade 2 or baseline.<br>• Restart PDR001/placebo at the same regimen, and reduce dabrafenib and trametinib at the next dose level. Re-escalation of the subject's dose is recommended if criteria in Section 6.3.2 are met.<br>• AE resolution to ≤ Grade 2 or baseline must occur within a maximum period of 12 weeks since Grade 3 or 4 event has been identified, otherwise PDR001/placebo must be permanently discontinued.<br>**3rd occurrence:**<br>• Permanently discontinue PDR001/placebo and interrupt dabrafenib and trametinib until toxicity recovers to ≤ Grade 2 or baseline.<br>• Once recovered, reduce dabrafenib and trametinib to the next dose level. Re-escalation of the subject's dose is recommended if criteria in Section 6.3.2 are met.<br>**4th occurrence:**<br>• Permanently discontinue dabrafenib and trametinib
Febrile Neutropenia	• Apply Institutional guidelines	• Follow modification guide for neutropenia Grade 4 (above).
**Thrombocytopenia**		
Grade 1, 2, 3 without clinically significant bleeding	• Grade 3: monitor blood test more frequently (every 7 days)	• Continue PDR001/placebo, dabrafenib and trametinib at the same dose.
Grade 3 with clinically significant bleeding<br>Grade 4	• Grade 3: monitoring blood test more frequently (every 3-5 days)	**1st occurrence:**<br>• Interrupt PDR001/placebo, dabrafenib and trametinib until toxicity resolves to ≤ Grade 2 or baseline.<br>• Restart PDR001/placebo, dabrafenib and trametinib at same dose.<br>**2nd occurrence:**

Neutropenia and thrombocytopenia suspected AEs (NCI-CTCAE v4.03)		
**Grade**	**Recommended adverse event management guidelines**	**Mandatory dose modification requirements**
		• Interrupt PDR001/placebo, dabrafenib and trametinib until toxicity resolves to ≤ Grade 2 or baseline.<br>◦ Restart dabrafenib and trametinib at the next lower dose per Table 6-7 and restart PDR001/placebo. Re-escalation of the subject's dose is recommended if criteria in Section 6.3.2 are met.<br>◦ AE resolution to ≤ Grade 2 or baseline must occur within a maximum period of 12 weeks since Grade 3 with clinically significant bleeding or Grade 4 event has been identified, otherwise PDR001/placebo must be permanently discontinued.<br>**3rd occurrence:**<br>• Interrupt PDR001/placebo, dabrafenib and trametinib until toxicity resolves to ≤ Grade 2 or baseline.<br>◦ Permanently discontinue PDR001/placebo.<br>◦ Restart dabrafenib and trametinib at another lower dose per Table 6-7. Re-escalation of the subject's dose is recommended if criteria in Section 6.3.2 are met.<br>**4th occurrence:**<br>• Interrupt PDR001/placebo, dabrafenib and trametinib until toxicity resolves to ≤ Grade 2 or baseline.<br>◦ Permanently discontinue dabrafenib and trametinib.

6.3.3.11 Mandatory dose modifications and recommended clinical management guidelines for adverse events including abnormal laboratory related AEs

Guidelines for dose modification for adverse events including abnormal laboratory related AEs suspected to be related to study treatment by the investigator are provided in Table 6-18.

Table 6-18 Mandatory dose modifications and recommended clinical management guidelines for adverse events including abnormal laboratory related AEs

**Non-Hematologic AEs (except Grade 2 alopecia, Grade 2 fatigue) and abnormal laboratory related AEs (except neutropenia, thrombocytopenia) (NCI-CTCAE v4.03)**		
**Grade**	**Recommended adverse event management guidelines**	**Mandatory dose modification requirements**
Grade 1-2 tolerable	• Monitor closely.<br>• Provide supportive care according to institutional standards.	• Continue PDR001/placebo, dabrafenib and trametinib at the same dose.
Grade 2 intolerable or<br>Grade 3	• Monitor closely<br>• Provide supportive care according to institutional standards	**1st or 2nd occurrence:**<br>• Interrupt PDR001/placebo, dabrafenib and trametinib until toxicity recovers to ≤ Grade 1 or baseline.<br>• Restart PDR001/placebo, dabrafenib and trametinib at the same dose.<br>• AE resolution to ≤1 Grade or baseline must occur within a maximum period of 12 weeks since intolerable Grade 2 or Grade 3 event has been identified, otherwise PDR001 must be permanently discontinued.<br>**3rd occurrence:**<br>• Permanently discontinue PDR001/placebo.<br>• Restart dabrafenib and trametinib at the next lower dose per Table 6-7. Re-escalation of the subject's dose is recommended if criteria in Section 6.3.2 are met.<br>**4th occurrence:**<br>• Permanently discontinue dabrafenib and trametinib.

Non-Hematologic AEs (except Grade 2 alopecia, Grade 2 fatigue) and abnormal laboratory related AEs (except neutropenia, thrombocytopenia) (NCI-CTCAE v4.03)		
**Grade**	**Recommended adverse event management guidelines**	**Mandatory dose modification requirements**
Grade 4	• Monitor closely<br>• Provide supportive care according to institutional standards	**1st occurrence:**<br>• Interrupt PDR001/placebo and dabrafenib and trametinib.<br>• Permanently discontinue PDR001/placebo. If benefit risk assessment support treatment continuation, re-start PDR001/placebo. Dabrafenib and trametinib may be reinitiated at the next lower dose per Table 6-7. Re-escalation of the subject's dose is recommended if criteria in Section 6.3.2 are met.<br>• For asymptomatic amylase and/or lipase* elevation, if AE resolves to ≤ Grade 1 or baseline within 4 weeks, subject can resume treatment with PDR001/placebo, otherwise PDR001/placebo must be permanently discontinued.<br>**2nd occurrence:**<br>• Permanently discontinue PDR001/placebo, dabrafenib and trametinib.<br>• For asymptomatic amylase and/or lipase* elevation, if AE resolves to ≤ Grade 1 or baseline within 4 weeks, subject can resume treatment with PDR001/placebo, otherwise PDR001/placebo must be permanently discontinued. Dabrafenib and trametinib may be reinitiated at the next lower dose per Table 6-7. Re-escalation of the subject's dose is recommended if criteria in Section 6.3.2 are met.<br>• Asymptomatic amylase and/or lipase elevation in the absence of clinical evidence of pancreatitis. This includes negative radiographic evaluation as well as absence of clinical symptoms of pancreatitis.

6.3.3.12 Mandatory dose modifications and recommended clinical management guidelines for other adverse events of special interest

Guidelines for mandatory dose modifications and clinical management of "other" AEs are provide in Table 6-19. Under the category of "other" are several AEs of special interest that must be managed specifically. "Other" AEs included in this section are: autoimmune neuropathy, demyelinating polyneuropathy, Guillain-Barre, myasthenia Gravis-like syndrome, non-infectious myocarditis, non-infectious pericarditis, pancreatitis and, rapid onset of Grade 3 fatigue in the absence of disease progression.

Table 6-19 Mandatory dose modifications and recommended clinical management guidelines for other AEs of special interest

Other: Autoimmune neuropathy, demyelinating polyneuropathy, Guillane-Barré, Myasthenia Gravis- like syndrome, Non-infectious myocarditis, pericarditis, pancreatitis (for asymptomatic amylase and/or lipase elevations refer to Table 6-18), and Grade 3 Fatigue with rapid onset in absence of disease progression		
**Grade**	**Recommended adverse event management guidelines**	**Mandatory dose modification requirements**
Mild (Grade 1)	• Provide symptomatic treatment	• Continue PDR001/placebo, dabrafenib and trametinib at the same dose.
Moderate (Grade 2) or Grade 1 that does not improve with symptomatic treatment	• Provide symptomatic treatment.<br>• Systemic corticosteroids may be indicated.<br>• Recommend biopsy for confirmation of diagnosis.<br>• A specialist should be consulted.	**1st occurrence:**<br>• Interrupt PDR001/placebo and dabrafenib and trametinib until recovery to ≤ Grade 1 or baseline.<br>• Restart PDR001/placebo at the same schedule.<br>• Continue dabrafenib and trametinib at the same dose.<br>• AE resolution to ≤ Grade 1 or baseline must occur within a period of 12 weeks since event has been identified, otherwise PDR001/placebo must be permanently discontinued.<br>**2nd occurrence:**<br>• Permanently discontinue PDR001/placebo.<br>• Interrupt dabrafenib and trametinib until recovery to ≤ Grade 1 or baseline, dabrafenib and trametinib may be reduced to the next lower dose per Table 6-7. Re-escalation of the subject's dose is recommended if criteria in Section 6.3.2 are met.<br>**3rd occurrence:**<br>• Interrupt dabrafenib and trametinib until toxicity resolves to ≤ Grade 1 or baseline, then reduce dabrafenib and trametinib to the next lower dose level per Table 6-7. Re-escalation of the subject's dose is recommended if criteria in Section 6.3.2 are met.<br>**4th occurrence:**<br>• Permanently discontinue dabrafenib and trametinib.
Severe (Grade 3)	• All management for moderate irAEs<br>• Initiate systemic corticosteroids (prednisone or equivalent) at a dose of 1-2 mg/kg QD.	**1st occurrence:**<br>• Interrupt PDR001/placebo, dabrafenib and trametinib until

Other: Autoimmune neuropathy, demyelinating polyneuropathy, Guillane-Barré, Myasthenia Gravis- like syndrome, Non-infectious myocarditis, pericarditis, pancreatitis (for asymptomatic amylase and/or lipase elevations refer to Table 6-18), and Grade 3 Fatigue with rapid onset in absence of disease progression		
**Grade**	**Recommended adverse event management guidelines**	**Mandatory dose modification requirements**
		recovery to ≤ Grade 1 or baseline<br>• Restart PDR001/placebo at the same schedule.<br>• Dabrafenib and trametinib may be reduced to the next lower dose per Table 6-7. Re-escalation of the subject's dose is recommended if criteria in Section 6.3.2 are met.<br>• AE resolution to ≤ Grade 1 or baseline must occur within a maximum period of 12 weeks since a Grade 3 event has been identified, otherwise PDR001/placebo must be permanently discontinued.<br>**2nd occurrence:**<br>• Permanently discontinue PDR001/placebo and consider reducing dabrafenib and trametinib to the next lower dose per Table 6-7. Re-escalation of the subject's dose is recommended if criteria in Section 6.3.2 are met.<br>**3rd occurrence:**<br>• Permanently discontinue dabrafenib and trametinib.
Grade 4	• Refer to management of severe AEs	• Permanently discontinue PDR001.

6.3.4 Mandatory dose modifications and recommended clinical management guidelines for infusion reactions

Acute infusion reactions (which can include cytokine release syndrome, angioedema, or anaphylaxis) are different from allergic/hypersensitive reactions, although some of the manifestations are common to both AEs. Signs and symptoms usually develop during or shortly after drug infusion and generally resolve completely within 24 hours of completion of infusion.

Table 6-20 Mandatory dose modifications and recommended clinical management guidelines for infusion reaction and cytokine release syndromes

Infusion reaction and cytokine release syndrome (NCI-CTCAE v4.03)		
**Grade**	**Recommended Adverse Event management guidelines**	**Mandatory Dose Modification requirements**
Grade 1 infusion reaction or Grade 1 cytokine release syndrome: mild/mild transient reaction; infusion interruption not indicated; intervention not indicated.	Increase monitoring of vital signs as medically indicated until the patient is deemed medically stable in the opinion of the investigator.	• Continue PRD001/placebo<br>• Continue dabrafenib and trametinib at the same dose level.
Grade 2 infusion reaction or Grade 2 cytokine release syndrome: therapy or infusion interruption indicated but responds promptly to symptomatic treatment (e.g., antihistamines, NSAIDS, narcotics, IV fluids); prophylactic medications indicated for ≤ 24 hr	• Stop Infusion<br>• Additional appropriate medical therapy may include but is not limited to:<br>◦ IV fluids<br>◦ Antihistamines<br>◦ NSAIDS<br>◦ Acetaminophen<br>◦ Narcotics<br>• Increase monitoring of vital signs as medically indicated until the patient is deemed medically stable in the opinion of the investigator.<br>• If symptoms resolve within one hour of stopping drug infusion, the infusion may be restarted at 50% of the original infusion rate (e.g., from 100 mL/hr to 50 mL/hr). Otherwise dosing will be held until symptoms resolve and the patient should be re-premedicated for the next scheduled dose.<br>• Patient may be premedicated 1.5 hr (± 30 minutes) prior to infusion of PDR001 with:<br>◦ Diphenhydramine 50 mg PO (or equivalent dose of antihistamine).<br>◦ Acetaminophen 500-1000 mg PO (or equivalent dose of analgesic).	**1st occurrence:**<br>• Continue PRD001/placebo treatment with premedication<br>**2nd occurrence:**<br>• Despite premedication or prolongation of infusion, consider permanent discontinuation of PDR001/placebo.
Grade 3 infusion reaction or grade 3 cytokine release syndrome: prolonged (e.g., not rapidly responsive to symptomatic medication and/or brief interruption of infusion); recurrence of symptoms following initial improvement; hospitalization indicated for clinical sequelae (e.g., renal impairment, pulmonary infiltrates)	• Stop Infusion<br>• Additional appropriate medical therapy may include but is not limited to:<br>◦ IV fluids<br>◦ Antihistamines<br>◦ NSAIDS<br>◦ Acetaminophen<br>◦ Narcotics<br>◦ Oxygen	• Permanently discontinue PDR001/placebo

Infusion reaction and cytokine release syndrome (NCI-CTCAE v4.03)		
**Grade**	**Recommended Adverse Event management guidelines**	**Mandatory Dose Modification requirements**
Grade 4:<br>Grade 4 infusion reaction: Life-threatening consequences; urgent intervention indicated<br>OR<br>Grade 4 cytokine release syndrome: Life-threatening consequences; pressor or ventilatory support indicated	• Pressors<br>• Corticosteroids<br>• Epinephrine<br>• Increase monitoring of vital signs as medically indicated until the patient is deemed medically stable in the opinion of the investigator. Hospitalization may be indicated.	

6.3.4.1 Guidance for corticosteroids tapering for management of immune-related AEs

Reduce prednisone dose by 2.5 to 5.0 mg decrements every 3–7 days until physiologic dose (5 to 7.5 mg of prednisone per day) is reached. Consider to complete tapering over a period of at least 4 weeks. Slower tapering of corticosteroids therapy may be recommended if the adverse event is not showing improvement. Once corticosteroid tapering is achieved at a level of ≤ 10 mg of prednisone/day (or equivalent), PDR001 can be restarted as indicated in the dose modification tables.

6.3.5 Dose modification and management guideline for adverse events suspected to be related to dabrafenib and/or trametinib

Continue PRD001/placebo.

For adverse events of special interest reported **only for dabrafenib and/or trametinib**, general guidelines for dose modification in Table 6-21 should be followed for dabrafenib and trametinib.

In general, if an AE resolves to Grade 1 or baseline at the reduced dose level, and no additional toxicities are seen after 4 weeks of study treatment at the reduced dose, the dose may be increased to the previous dose level.

A dose reduction below 50 mg BID for dabrafenib or below 1 mg QD for trametinib is not allowed. If a dose reduction below 50 mg BID for dabrafenib is required, dabrafenib will be permanently discontinued but these subjects will be allowed to continue trametinib. If a dose reduction below 1.0 mg QD for trametinib is required, then trametinib will be permanently discontinued, but these subjects will be allowed to continue dabrafenib.

Table 6-21 General guidelines for dose modification for adverse events suspected to be related to dabrafenib and/or trametinib treatment

Grade	Recommended adverse event management guidelines	Mandatory dose modification requirements
Grade 1 or Grade 2 tolerable	• Monitor closely.<br>• Provide supportive care according to institutional standards.	• Continue dabrafenib and trametinib at the same dose level.
Grade 2 (intolerable) or Grade 3	• Monitor closely.<br>• Provide supportive care according to institutional standards.	• Interrupt dabrafenib and trametinib (except for cuSCC, keratoacanthoma, new primary melanoma, and basal cell carcinoma)<br>• When toxicity resolves to ≤ Grade 1 or baseline, restart dabrafenib and trametinib reduced by one dose level per Table 6-7.<br>• If the Grade 2 (intolerable) or Grade 3 toxicity recurs, interrupt dabrafenib and trametinib.<br>• When toxicity resolves to Grade 1 or baseline, restart dabrafenib and trametinib reduced by another dose level per Table 6-7. Re-escalation of the subject's dose is recommended if criteria in Section 6.3.2 are met.
Grade 4	• Monitor closely.<br>• Provide supportive care according to institutional standards.	• Interrupt dabrafenib and trametinib.<br>• Restart with dabrafenib and trametinib reduced by one dose level per Table 6-7 once toxicity resolves to ≤ Grade 1 or baseline or permanently discontinue dabrafenib and trametinib at the discretion of investigator.

6.3.5.1 Mandatory dose modification and management guideline for changes in LVEF suspected to be related to dabrafenib and/or trametinib treatment

Decreases of left-ventricular-ejection-fraction (LVEF) have been observed in subjects receiving trametinib monotherapy and in combination with dabrafenib. Therefore, ECHOs must be performed to assess cardiac ejection fraction in regular intervals as outlined in the Visit Evaluation Schedule (Table 7-1, Table 7-2, Table 7-3, and Table 7-4). Dose modification guidance and stopping criteria for LVEF decrease are provided in Table 6-22.

Table 6-22 Mandatory dose modification and recommended clinical management for changes in LVEF suspected to be related to dabrafenib and/or trametinib treatment

Left Ventricular Ejection Fraction decrease (NCI-CTCAE v4.03)		
**LVEF-drop (%) & clinical symptoms**	**Recommended adverse event management guidelines**	**Mandatory dose modification requirements**
**Asymptomatic:**<br>Absolute decrease of >10% in LVEF compared to baseline **and** ejection fraction below the institution's LLN	• Report as SAE.<br>• Closely monitoring LVEF via ECHO, repeat ECHO within 2 weeks*.<br>• If the LVEF recovers within 4 weeks (defined as LVEF ≥LLN **and** absolute decrease ≤10% compared to baseline)<br>• Repeat ECHO 2, 4, 8 and 12 weeks after re-start; continue in intervals of 12 weeks thereafter.<br>• If repeat LVEF does not recover within 4 weeks.<br>• Consult with cardiologist<br>• Repeat ECHO after 2, 4, 8, 12, and 16 weeks or until resolution	• Interrupt trametinib.<br>• If the LVEF recovers, restart trametinib reduced by one dose level per Table 6-7 and continue dabrafenib at the same dose level.<br>• More than two occurrence, permanently discontinue trametinib.<br>• Permanently discontinue trametinib if repeat LVEF does not recover within 4 weeks.
**Symptomatic:**<br>Resting LVEF 39-20%<br>or >20% absolute reduction from baseline<br>resting LVEF <20%	• Report as SAE.<br>• Consult with cardiologist.<br>• Repeat ECHO after 2, 4, 8, 12, and 16 weeks or until resolution.	• Permanently discontinue trametinib.<br>• Interrupt dabrafenib<br>• Restart dabrafenib if LVEF recovers including resolution of symptoms.

* If ECHO does not show LVEF recovery after 2 weeks, repeat ECHO 2 weeks later.

6.3.5.2 Mandatory dose modification and management guideline for hypertension suspected to be related to dabrafenib and/or trametinib treatment

Increases in blood pressure have been observed in subjects receiving trametinib. For adequate monitoring and management of hypertension, all blood pressure assessments should be performed under the following optimal conditions:

- the subject has been seated with back support, ensuring that legs are uncrossed and flat on the floor
- the subject is relaxed comfortably for at least 5 minutes
- restrictive clothing has been removed from the cuff area and the right cuff size has been selected
- the subject's arm is supported so that the middle of the cuff is at heart level
- the subject remains quiet during the measurement.

In subjects with an initial blood pressure reading within the hypertensive range, a second reading should be taken at least 1 minute later, with the 2 readings averaged to obtain a final blood pressure measurement. The averaged value should be recorded in the eCRF.

Persistent hypertension is defined as an increase of systolic blood pressure (SBP) > 140 mm Hg and/or diastolic blood pressure (DBP) > 90 mm Hg in three consecutive visits with blood pressure assessments from two readings collected as described above. Visits to monitor increased blood pressure can be scheduled independently from the per- protocol visits outlined in the Visit Evaluation Schedule (Section 7.1). Ideally, subsequent blood pressure assessments should be performed within one week.

Asymptomatic hypertension is defined as an increase of SBP >140 mm Hg and/or DBP >90 mmHg in the absence of headache, light-headedness, vertigo, tinnitus, episodes of fainting or other symptoms indicative of hypertension.

For subjects experiencing an increase in systolic and/or diastolic blood pressure that is persistent and may be associated with the study treatment, recommendations for dose modifications and management of hypertension are described below in Table 6-23.

Table 6-23 Mandatory dose modification and recommended clinical management for hypertension suspected to be related to dabrafenib and/or trametinib treatment

**Hypertension (NCI-CTCAE 4.03)**		
**Severity**	**Recommended adverse event management guidelines**	**Mandatory dose modification requirements**
(Scenario A)<br>• Asymptomatic and persistent [a] SBP of >140 and <160 mmHg, or DBP >90 and <100 mmHg, or<br>• Clinically significant increase in DBP of 20 mmHg (but still below 100 mmHg).	• Adjust current or initiate new antihypertensive medication.<br>• Titrate antihypertensive medication(s) during the next 2 weeks as indicated to achieve well-controlled [b] BP<br>• If BP is not well controlled within 2 weeks, recommended to refer to a specialist and go to scenario (B).	• Continue dabrafenib and trametinib at the same dose.
(Scenario B)<br>• Asymptomatic SBP ≥160 mmHg, or DBP ≥100 mmHg, or<br>Failure to achieve well-controlled BP within 2 weeks in Scenario A	• Adjust current or initiate new antihypertensive medication(s).<br>• Titrate antihypertensive medication(s) during the next 2 weeks as indicated to achieve well-controlled BP.	• Interrupt dabrafenib and trametinib if clinically indicated.<br>• Once BP is well controlled, restart dabrafenib and trametinib reduced by one dose level per Table 6-7.
• Symptomatic [c] hypertension or<br>• Persistent SBP ≥160 mmHg, or DBP ≥100 mmHg, despite antihypertensive medication and dose reduction of study treatment.	• Adjust current or initiate new antihypertensive medication(s)<br>• Titrate antihypertensive medication during the next 2 weeks as indicated to achieve well-controlled BP.<br>• Referral to a specialist for further evaluation and follow-up is recommended	• Interrupt dabrafenib and trametinib.<br>• Once BP is well controlled, restart dabrafenib and trametinib reduced by one dose level per Table 6-7.
• Refractory hypertension unresponsive to above interventions or hypertensive crisis.	• Continue follow-up per protocol.	• Permanently discontinue dabrafenib and trametinib.

Hypertension (NCI-CTCAE 4.03)		
**Severity**	**Recommended adverse event management guidelines**	**Mandatory dose modification requirements**

a. Hypertension detected in two separate readings during up to three consecutive visits.

b. Well-controlled blood pressure defined as SBP ≤140 mm Hg and DBP ≤90 mm Hg in two separate readings during up to three consecutive visits.

c. Symptomatic hypertension defined as hypertension aggravated by symptoms (e.g., headache, light-headedness, vertigo, tinnitus, episodes of fainting) that resolve after the blood pressure is controlled within the normal range.

6.3.5.3 Mandatory dose modification and management guideline for new malignancies suspected to be related to dabrafenib and/or trametinib treatment

New cutaneous malignancies:

Cutaneous squamous cell carcinoma (CuSCC), keratoacanthomas (KA) and new primary melanomas have been observed in subjects treated with dabrafenib and dabrafenib/trametinib combination therapy. These treatment-related lesions should be surgically removed according to institutional practices. Dose modification or interruption of study treatment is not required for cuSCC, KA, or new primary melanoma, however cuSCC and new primary melanoma should be reported as a SAE. In addition, a biopsy of the lesion should be taken, where possible, and submitted for further analyses and a summary of the results submitted to Novartis.

Subjects should be instructed to immediately inform their physician if new lesions develop. Skin examination should be performed prior to initiation of study treatment and throughout therapy as detailed in the Visit Evaluation Schedule. Monitoring should continue every month for 6 months following discontinuation of dabrafenib or until initiation of another anti-neoplastic therapy.

New non-cutaneous malignancies:

In vitro experiments have demonstrated paradoxical activation of MAP-kinase signaling in *BRAF* wild type cells with RAS mutations when exposed to *BRAF* inhibitors, which may lead to increased risk of non-cutaneous malignancies in subjects treated with dabrafenib. Cases of RAS-driven malignancies have been seen with *BRAF* inhibitors, including dabrafenib. Subjects should be monitored as clinically appropriate. Consider the benefits and risks before continuing treatment with dabrafenib in subjects with a non-cutaneous malignancy harboring a RAS mutation. No dose modification of trametinib is required when taken in combination with dabrafenib.

Following discontinuation of dabrafenib, monitoring for non-cutaneous secondary/recurrent malignancies should continue for up to 6 months or until initiation of another anti-neoplastic therapy.

New cutaneous and non-cutaneous malignancies that are reported to the Investigator should be reported as an SAE. A biopsy of the new malignancy should be taken, where possible, and submitted for further analyses including RAS mutation status. Testing of these biopsies may include analysis of genomic alterations, which include but not limited to DNA, RNA and protein analysis of these biopsy specimens, and would analyze the biological pathways known to be associated with, and relevant to, *BRAF*-mutant tumor activation.

6.3.5.4 Mandatory dose modification and recommended clinical management for pyrexia syndrome suspected to be related to dabrafenib and/or trametinib treatment

Episodes of pyrexia syndrome have been observed in subjects receiving dabrafenib monotherapy or in combination with trametinib. The pyrexia syndrome is defined as treatment-related fever (≥ 38°C) or chills/rigors/night sweats or flu-like symptoms. In a minority of cases the pyrexia was accompanied by symptoms such as severe rigors/chills, dehydration, hypotension, dizziness or weakness and required hospitalization. The incidence and severity of pyrexia syndrome are increased when dabrafenib is used in combination with trametinib compared to dabrafenib monotherapy.

Dabrafenib and trametinib must be interrupted promptly at the very first symptom of pyrexia or its associated prodrome (chills or rigors or night sweats or flu-like symptoms) and should be restarted upon improvement of symptoms at the same dose if symptom free at least 24 hours.

Subjects should be instructed on the importance of immediately reporting febrile episodes. In the event of a fever, the subject should be instructed to take non-steroidal anti-pyretics as appropriate to control fever. The use of oral corticosteroids should be considered in those instances in which anti-pyretics are insufficient. In subjects experiencing pyrexia associated with rigors, severe chills, dehydration or hypotension, serum creatinine and other evidence of renal function should be monitored carefully during and following severe events of pyrexia.

Pyrexia accompanied by hypotension, dehydration requiring intravenous fluids, renal insufficiency and/or severe (≥ Grade 3) rigors/chills in the absence of an obvious infectious cause should be reported as a SAE.

Guidelines for dose modification and management for pyrexia syndrome considered to be related to dabrafenib are provided in Table 6-24.

Table 6-24 Mandatory dose modification and recommended clinical management for pyrexia syndrome suspected to be related to dabrafenib and/or trametinib treatment

**Pyrexia**		
**Occurrence**	**Recommended adverse event management guidelines**	**Mandatory dose modification requirements**
1st occurrence and subsequent occurences	• Educate patient about pyrexia syndrome and to immediately interrupt dabrafenib and trametinib at the very first symptom of pyrexia or its associated prodrome (chills or rigors or night sweats or flu-like symptoms).<br>• Clinical evaluation for infection and hypersensitivity [a]<br>• Laboratory work-up [a]<br>• Administer anti-pyretic treatment with non-steroidal anti-inflamatory drugs (NSAID) and/or paracetamol [b]<br>• Recommend oral corticosteroids	• Dabrafenib and trametinib must be interrupted promptly at the very first symptom of pyrexia or its associated prodrome (chills, rigors, night sweats or flu-like symptoms) and should be restarted upon improvement of symptoms at the same dose if symptom free at least 24 hours.<br>• If pyrexia cannot be managed with interruption, dose reduction per Table 6-7 must be considered if clinically indicated. For re-escalation, refer to Section 6.3.2.<br>• Note: proactive intermittent dosing is not allowed.

Pyrexia		
**Occurrence**	**Recommended adverse event management guidelines**	**Mandatory dose modification requirements**
	(i.e., prednisone 10 mg) for at least 5 days or as clinically indicated.<br>• Optimize oral corticosteroid dose as clinically indicated for recalcitrant pyrexia.<br>• Oral hydration should be encouraged in subjects without evidence of dehydration. Intravenous hydration is recommended in subjects experiencing pyrexia complicated by dehydration/hypotension.	

[a.] For subjects experiencing pyrexia, a clinical evaluation and laboratory work-up is mandatory for each event; thorough clinical examination for signs and symptoms of infection or hypersensitivity is required; laboratory work-up should include full-blood-count, electrolytes, creatinine, blood urea nitrogen (BUN), C-reactive protein (CRP), liver-function tests, blood culture, and urine culture.

[b.] Anti-pyretic treatment should be started immediately at the first occurrence and prophylactic anti-pyretic treatment is recommended. Anti-pyretic treatment may include acetaminophen, ibuprofen, or suitable anti-pyretic medication according to institutional standards. Prophylactic anti-pyretic treatment is recommended to be discontinued after three days in the absence of pyrexia.

6.3.5.5 Mandatory dose modification and management guideline for visual changes suspected to be related to dabrafenib and/or trametinib treatment

Episodes of visual changes have been observed in subjects receiving trametinib, dabrafenib or the combination of dabrafenib/trametinib. An ophthalmologist should be consulted if changes in vision develop. However, if the visual changes are clearly unrelated to study treatment (e.g., allergic conjunctivitis), then monitor closely as it may be reasonable to defer ophthalmic examination.

Treatment with dabrafenib has been associated with the development of uveitis, including iritis. Monitor subjects for visual signs and symptoms (such as, change in vision, photophobia and eye pain) during therapy. Permanently discontinue dabrafenib for persistent ≥ Grade 2 uveitis (including iritis and iridocyclitis) of > 6 weeks duration. No dose modification of trametinib is required when taken in combination with dabrafenib.

In subjects treated with trametinib, special attention should be given to retinal findings (e.g., RPED) or retinovascular abnormalities (i.e., branch or central RVO). Treatment emergent cases of RVO and RPED should be reported as SAEs.

Guidelines for dose modification and management for visual changes and/or ophthalmic examination findings considered to be related to dabrafenib and/or trametinib are provided in Table 6-25 and Table 6-26.

Table 6-25 Mandatory dose modification and recommended clinical management for visual changes and/or ophthalmic examination findings suspected to be related to dabrafenib and/or trametinib treatment

Visual changes (Eye disorders – Other, CTCAE Version 4.03)		
**Grade**	**Recommended adverse event management guidelines**	**Mandatory dose modification requirements**
Grade 1 [a]	• Consult ophthalmologist within 7 days of onset	• If dilated fundus examination cannot be performed within 7 days of onset, interrupt trametinib until RPED and RVO can be excluded by retina specialist/ophthalmologist. Continue dabrafenib.<br>• If RPED and RVO excluded, continue (or restart) trametinib at the same dose level.<br>• **If RPED suspected or diagnosed:** see RPED dose modification in Table 6-26; **report as SAE if diagnosed**.<br>• **If RVO diagnosed:** Permanently discontinue trametinib and report as SAE.
Grade 2 and Grade 3	• Consult ophthalmologist immediately	• Interrupt trametinib. Dabrafenib may be continued at the same dose [b].If RPED and RVO excluded, restart trametinib at the same dose level.<br>• **If RPED diagnosed**, see RPED dose modification in Table 6-26; **report as SAE**.<br>• **If RVO diagnosed: Permanently discontinue trametinib and report as SAE.**
Grade 4	• Consult ophthalmologist immediately	• Interrupt trametinib. Dabrafenib may be continued at the same dose [b].If RPED and RVO excluded, should consider restarting trametinib at same or reduced dose after discussion with study medical monitor.<br>• If RVO or RPED diagnosed, permanently discontinue trametinib and report as SAE.

[a.] If visual changes are clearly unrelated to study treatment (e.g., allergic conjunctivitis), monitor closely but ophthalmic examination is not required.

[b.] Permanently discontinue dabrafenib for ≥ Grade 2 uveitis (including iritis and iridocyclitis) of > 6 weeks duration.

Table 6-26 Mandatory dose modification and recommended clinical management for retinal pigment epithelial detachments (RPED) suspected to be related to trametinib treatment

Retinal pigment epithelial detachments (RPED)		
**Grade**	**Recommended adverse event management guidelines**	**Mandatory dose modification requirements**
• Grade 1 (Asymptomatic; clinical or diagnostic observations only)	• If RPED worsens follow instructions below.	• Continue treatment with retinal evaluation monthly until resolution.
• Grade 2-3 RPED (Symptomatic with mild to moderate decrease in visual acuity).	• Retinal evaluation monthly.	• Interrupt trametinib.<br>• If improved to ≤ Grade 1, restart trametinib at a lower dose per Table 6-7.

6.3.5.6 Monitoring guideline for hyperglycemia suspected to be related to dabrafenib treatment

Hyperglycemia requiring an increase in the dose of, or initiation of insulin or oral therapy can occur with dabrafenib. Monitor serum glucose levels as clinically appropriate during treatment with dabrafenib in subjects with pre-existing diabetes or hyperglycemia.

Advise subjects to report symptoms of severe hyperglycemia such as excessive thirst or any increase in the volume or frequency of urination.

6.3.5.7 Mandatory dose modification and management guideline for hemorrhage

Hemorrhage, including major hemorrhage defined as symptomatic bleeding in a critical area or organ, can occur when dabrafenib is administered with trametinib. Permanently discontinue dabrafenib and trametinib for all Grade 4 hemorrhagic events and for any persistent Grade 3 hemorrhagic events. Withhold dabrafenib and trametinib for Grade 3 hemorrhagic events; if improved, resume at the next lower dose level.

6.3.5.8 Mandatory dose modification and management guideline thromboembolic events

Advise patients to immediately seek medical care if they develop symptoms of deep vein thrombosis (DVT) or pulmonary embolism (PE), such as shortness of breath, chest pain, or arm or leg swelling. If any signs or symptoms of venous thromboembolism are present, the subject will undergo specific laboratory and medical imaging studies to confirm it. The medical imaging study or studies selected will depend on the anatomic site or organ of involvement (e.g., Doppler ultrasound, venography, ventilation perfusion lung scan, angiography, MRI). If the diagnosis is confirmed, appropriate medical care according to standard local clinical practice should be initiated immediately.

Permanently discontinue trametinib for life threatening PE. Withhold trametinib for uncomplicated venous thromboembolism for up to 3 weeks; if improved, trametinib may be resumed at a lower dose level.

6.3.6 Follow-up for toxicities

6.3.6.1 Follow up on potential immune related adverse events

The emergence of irAE may be anticipated based on the mechanism of action of immunomodulatory therapies.

Serologic, histologic (tumor sample) and immunological assessments should be performed as deemed appropriate by the Investigator to verify the immune-related nature of the AE and to exclude alternative explanations. Recommendations (Section 6.3) have been developed to assist investigators in assessing and managing the most frequently occurring irAEs.

Subjects whose treatment is interrupted or permanently discontinued due to an irAE, AE or clinically significant laboratory value, must be followed-up at least once a week (or more frequently if required by institutional practices, or if clinically indicated) for 30 days, and subsequently at approximately 30-day intervals (or more frequently if required by institutional

practices, or if clinically indicated), until resolution or stabilization of the event, whichever comes first. Appropriate clinical experts should be consulted as deemed necessary.

If an AE is suspected to be immune-related the relevant immunological assessments (e.g., rheumatoid factor, anti-DNA Ab, etc.) should be performed. If cytokine release syndrome is suspected, the assessments outlined in Section 7.2.2.6.8 must be performed. All subjects must be followed-up for irAEs, AEs and SAEs for 150 days following the last dose of PDR001/placebo. However, if the subject begins post treatment antineoplastic medication before the 150-Day safety visit the collection of new SAEs and AEs unrelated to study medication will stop and thereafter only suspected SAEs and suspected AEs will continue to be collected to Day 150. If SAEs suspected to be related to study medication occur beyond Day 150, information should also be collected.

6.3.6.2 Follow up on potential drug-induced liver injury (DILI) cases

Subjects with transaminase increase combined with total bilirubin (TBIL) increase may be indicative of potential DILI, and should be considered as clinically important events.

The threshold for potential DILI may depend on the subject's baseline AST/ALT and TBIL value; subjects meeting any of the following criteria will require further follow-up as outlined below:

- For subjects with normal ALT and AST and TBIL value at baseline: AST or ALT > 3.0 x ULN combined with TBIL > 2.0 x ULN
- For subjects with elevated AST or ALT or TBIL value at baseline: [AST or ALT > 2 x baseline AND > 3.0 x ULN] OR [AST or ALT > 8.0 x ULN], combined with [TBIL > 2 x baseline AND > 2.0 x ULN]

Medical review needs to ensure that liver test elevations are not caused by cholestasis, defined as ALP elevation > 2.0 x ULN with R value < 2 in subjects without bone metastasis, or elevation of ALP liver fraction in subjects with bone metastasis.

Note: (The R value is calculated by dividing the ALT by the ALP, using multiples of the ULN for both values. It denotes whether the relative pattern of ALT and/or ALP elevation is due to cholestatic ($R \leq 2$), hepatocellular ($R \geq 5$), or mixed ($R > 2$ and < 5) liver injury).

In the absence of cholestasis, these subjects should be immediately discontinued from study drug treatment, and repeat LFT testing as soon as possible, preferably within 48 hours from the awareness of the abnormal results. The evaluation should include laboratory tests, detailed history, physical assessment and the possibility of liver metastasis or new liver lesions, obstructions/compressions, etc.

1. Laboratory tests should include ALT, AST, albumin, creatine kinase, total bilirubin, direct and indirect bilirubin, GGT, prothrombin time (PT)/INR and alkaline phosphatase.
2. A detailed history, including relevant information, such as review of ethanol, concomitant medications, herbal remedies, supplement consumption, history of any pre-existing liver conditions or risk factors, should be collected.
3. Further testing for acute hepatitis A, B, C or E infection and liver imaging (e.g., biliary tract) may be warranted.

4. Additional testing for other hepatotropic viral infection (e.g., CMV, EBV, Adenovirus, HSV, HHV6, HIV), autoimmune hepatitis or liver biopsy may be considered as clinically indicated or after consultation with specialist/hepatologist.

All cases confirmed on repeat testing meeting the laboratory criteria defined above, with no other alternative cause for LFT abnormalities identified should be considered as "medically significant", thus, met the definition of SAE (Section 8.2.1) and reported as SAE using the term "potential drug-induced liver injury". All events should be followed up with the outcome clearly documented.

6.4 Concomitant medications

The investigator must instruct the subject to notify the study site of any new medication(s) he/she takes after start of study participation. All prescription medications, OTC drugs and significant non-drug therapies (including physical therapy, herbal/natural medications and blood transfusions) taken within 28 days or 5 half-lives, whichever is shorter, including vitamins taken within one week prior to dosing must be recorded on the 'Prior and Concomitant Medications' or 'Surgical and Medical Procedures' eCRF. Prior antineoplastic therapies including medications, radiotherapy, and surgery are to be recorded on the separate 'Prior Antineoplastic Therapy eCRF' page during screening. Medication entries must be specific to trade name, dose and units, frequency and route of administration, start and discontinuation dates, and reason for therapy. For medications administered one time, the frequency column may reflect "once".

Administration of certain concomitant medications may lead to the requirement for subject to be discontinued. Discussions regarding discontinuation of subjects requiring concomitant medication will be discussed with Novartis on a case by case

6.4.1 Permitted concomitant therapy

In general, concomitant medications and therapies deemed necessary for the supportive care (e.g., such as anti-emetics, anti-diarrhea) and safety of the subject are allowed except those prohibited in Section 6.4.3.

Subjects are permitted to use the following medications during study treatment:

- Antivirus medications to manage HBV or HCV infection and/or prevent reactivation (e.g., tenofovir); supportive care.
- Medications to prevent or treat nausea or vomiting.
- Anti-diarrheal medications (e.g., loperamide) for subjects who develop diarrhea.
- Pain medication to allow the subject to be as comfortable as possible.
- Bone targeted therapies (e.g., bisphosphonates, denosumab) to treat bone metastases or to prevent skeletal related events.
- Any radiotherapy must be listed on the 'Concomitant Antineoplastic Therapy - Radiotherapy' eCRF.
- Immunosuppressive agents to treat suspected irAEs.
- Nutritional support or appetite stimulants (e.g., megestrol).
- Oxygen therapy and blood products or transfusions.

- Limited-field palliative radiotherapy to non-target lesion(s) may be allowed after documented discussion with Novartis study physician as concomitant therapy such as local therapies administered during the study treatment. In case of palliative radiotherapy, the subject should interrupt dabrafenib and trametinib for fractionated radiotherapy and stereotactic radiosurgery before and after radiotherapy as outlined below (Anker CJ et al, 2016).
    - Interrupt dabrafenib and trametinib ≥ 3 days before and after fractionated radiation
    - Interrupt dabrafenib and trametinib ≥ 1 day before and after stereotactic radiosurgery (SRS)
- Inactivated vaccines.
- The subject must be told to notify the investigational site about any new medications he/she takes after the start of the study drug. All medications (other than study drug) and significant non-drug therapies (including physical therapy, herbal/natural medications and blood transfusions) administered during the study must be listed on the Concomitant Medications or the Procedures and Surgical and Medical Procedures eCRF.

6.4.2 Permitted concomitant therapy requiring caution and/or action

6.4.2.1 PDR001

- Anticoagulation and anti-aggregation agents are permitted if the subjects are already at stable doses for > 2 weeks at the time of first dose of PDR001 and INR should be monitored as clinically indicated per investigator's discretion. However, ongoing anticoagulant therapy should be temporarily discontinued to allow tumor biopsy sample collection according to the institutional guidelines.

6.4.2.2 Dabrafenib

6.4.2.2.1 Effect of other drugs on dabrafenib

Based on *in vitro* studies, dabrafenib was shown to be primarily metabolized by CYP2C8 and CYP3A4. Medicinal products that are strong inhibitors or inducers of CYP2C8 or CYP3A4 are likely to increase or decrease, respectively, dabrafenib concentrations. Alternative agents should be considered during administration with dabrafenib when possible. Use caution if strong inhibitors (e.g., ketoconazole, nefazodone, clarithromycin, ritonavir, gemfibrozil) or inducers (e.g., rifampin, phenytoin, carbamazepine, phenobarbital, St John's wort) of CYP2C8 or CYP3A4 are co-administered with dabrafenib.

6.4.2.2.2 Effect of dabrafenib on other drugs

Dabrafenib induces CYP3A4- and CYP2C9- mediated metabolism and may induce other enzymes including CYP2B6, CYP2C8, CYP2C19 and UDP glucuronosyltransferases (UGT). Dabrafenib may also induce transporters (e.g., P-glycoprotein (P-gp)). Co-administration of dabrafenib and medicinal products which are affected by the induction of CYP3A4 or CYP2C9 such as hormonal contraceptives, warfarin or dexamethasone may result in decreased concentrations and loss of efficacy. If co-administration of these medications is necessary, monitor subjects for loss of efficacy or consider substitutions of these medicinal products. Use

caution if co-administration of CYP2C or CYP3A4 substrates with narrow therapeutic index is required. Refer to the Tafinlar label for further information.

6.4.2.3 Trametinib

Based on *in vitro* and *in vivo* data, trametinib is unlikely to significantly affect the pharmacokinetics of other medicinal products via interactions with CYP enzymes or transporters.

6.4.3 Prohibited concomitant therapy

6.4.3.1 PDR001/placebo

During the course of the study, subjects must not receive other additional investigational drugs, devices, chemotherapy, or any other therapies that may be active against cancer or modulate the immune responses. However, limited-field palliative radiotherapy to non-target lesion(s) may be allowed as concomitant therapy. Such local therapies administered during the study treatment must be entered into the CRF. Additionally, no other therapeutic monoclonal antibodies and no immunosuppressive medication may be administered while on this study unless given for the management of immune toxicity.

The use of systemic steroid therapy and other immunosuppressive drugs is not allowed except for the treatment of (recurrent) pyrexia, infusion reactions, irAEs, and for prophylaxis against imaging contrast dye allergy or replacement-dose steroids in the setting of adrenal insufficiency (providing this is ≤ 10 mg/day prednisone or equivalent), or transient exacerbations of other underlying diseases such as COPD requiring treatment for ≤ 3 weeks. If systemic corticosteroids are required for the control of infusion reactions, pyrexia or irAEs, it must be tapered and be at non-immunosuppressive doses (≤ 10 mg/day of prednisone or equivalent) before the next administration of study treatment.

The use of live vaccines is not allowed through the whole duration of the study. Inactivated vaccines are allowed.

There are no prohibited therapies during the **post-treatment follow-up period**.

6.4.3.2 Dabrafenib and trametinib

The following medications or non-drug therapies are prohibited while on treatment in this study:

- Other anti-cancer therapies
- Other investigational drugs

Although not contraindicated/prohibited, certain medications drugs should be used with caution due to potential drug-drug interactions (see Section 6.4.2.2).

6.4.4 Use of bisphosphonates (or other concomitant agents)

Localized radiotherapy and treatment with bisphosphonates for pre-existing, painful bone/liver metastases is permitted for non-target lesions. The radiotherapy must be listed on the ‘Concomitant Antineoplastic Therapy – Radiotherapy’ eCRF.

6.5 Subject numbering, treatment assignment or randomization

6.5.1 Subject numbering

Each subject is identified in the study by a 7-digit Subject Number (Subject No.), that is assigned when the subject is first enrolled for screening and is retained as the primary identifier for the subject throughout his/her entire participation in the trial.

- The Subject No. consists of the 4-digit Center Number (Center No.) (as assigned by Novartis to the investigative site) with a sequential 3-digit patient number suffixed to it, so that each subject is numbered uniquely across the entire database.
- Upon signing the informed consent form, the subject is assigned to the next sequential Subject No. available to the investigator through the Oracle Clinical RDC interface.
- The investigator or designated staff will contact the IRT and provide the requested identifying information for the subject to register them into the IRT.
- Once assigned, the Subject No. must not be reused for any other subject and the Subject No. for that individual must not be changed, even if the subject is re-screened.
- If the subject fails to be randomized or start treatment for any reason, the reason will be entered into the Screening Disposition page.
- IRT must be notified within 2 days that the subject was not randomized.

6.5.2 Treatment assignment or randomization

In Part 1 and 2 of this study, the assignment of a subject to a particular cohort will be coordinated by the sponsor via IRT.

In Part 3 of this study, subjects will be randomized via IRT in a ratio of 1:1 to one of the 2 treatment arms (Section 4 and Section 6.1):

- Arm 1: PDR001 in combination with dabrafenib and trametinib
- Arm 2: Placebo in combination with dabrafenib and trametinib

Randomization will be stratified by LDH level (< 1 x ULN vs. ≥ 1 to < 2 x ULN vs. ≥ 2 x ULN) and ECOG PS (0 vs. 1 vs. 2).

Random permuted blocks scheme will be used for this study.

The randomization numbers will be generated using the following procedure to ensure that treatment assignment is unbiased and concealed from subjects and investigator staff. A subject randomization list will be produced by the IRT provider using a validated system that automates the random assignment of subject numbers to randomization numbers. These randomization numbers are linked to the different treatment arms, which in turn are linked to medication numbers. A separate medication randomization list will be produced by or under the responsibility of Novartis Drug Supply Management using a validated system that automates the random assignment of medication numbers to medication packs containing each of the study treatments.

Prior to dosing, all subjects who fulfill all inclusion/exclusion criteria will be randomized via IRT to one of the treatment arms. The investigator or his/her delegate will call or log on to the IRT and confirm that the subject fulfills all the inclusion/exclusion criteria. The IRT will assign

a randomization number to the subject, which will be used to link the subject to a treatment arm and will specify a unique medication number for the first package of study treatment to be dispensed to the subject. The randomization number will not be communicated to the caller.

6.5.3 Treatment blinding

Parts 1 & 2: Safety run-in and biomarker cohort

Not applicable.

Part 3: Double-blind, randomized, placebo-controlled part:

Patients, investigator, site personnel and data analysts will remain blinded to the identity of the treatment from the time of randomization until database lock using the following methods: (1) Randomization data are kept strictly confidential until the time of unblinding and will not be accessible by anyone else involved in the study except for the unblinded pharmacist, unblinded CRAs, independent biostatistician and programmer who will perform DMC analysis and the PK bioanalyst. (2) Any potential visible difference in treatments will be concealed by the use of a non-translucent, opaque sleeve. (3) To the best extent possible the order of assessments will match the one used at each dosing visit, thus normalizing the process to all subjects. Randomization data are strictly confidential and will be accessible only to authorized personnel until unblinding of the trial after database lock. The study bioanalyst will receive a copy of the randomization schedule to facilitate analysis of the samples. The unblinded pharmacist, unblinded CRA, independent biostatistician and programmer and bioanalyst will keep treatment allocation information confidential until clinical database lock.

To limit the impact of unblinding on the scientific validity of the study results, individual patient unblinding will be prohibited, except for medical emergencies, (Section 8.3), for regulatory reporting purposes, or if it is critical to determine subsequent therapy after disease progression. Except in these cases, documented approval by the Novartis study physician is required prior to unblinding a subject's treatment assignment. In case of unblinding, all data is required to be captured in the eCRF prior to unblinding. Data after unblinding will continue to be collected as per protocol. The date of any unblinding and the reason will also be collected.

IMPORTANT: Due to the difference in preparation methods between the active and placebo treatments, an unblinded pharmacist/designee who is independent of the investigational staff will be required. This unblinded pharmacist will receive the appropriate treatment allocations. Appropriate measures must be taken by the unblinded pharmacist to ensure that the investigational staff remains blinded throughout the study. The unblinded pharmacist must not administer the drug to the subject nor have any contact with the study participants. Please refer to the Pharmacist Instruction Manual.

6.6 Study drug preparation and dispensation

The investigator or responsible site personnel must instruct the subject or caregiver to take dabrafenib and trametinib as per protocol. These study drug(s) will be dispensed to the subject by authorized site personnel only. All dosages prescribed to the subject and all dose changes during the study must be recorded on the Dosage Administration Record eCRF.

Dabrafenib and trametinib with instructions for administration will be provided to subjects for self-administration at home, until their next scheduled study visit.

PDR001/placebo will be administered intravenously as a 30 minute infusion (up to 2 hours, if clinically indicated). Subjects should be provided instructions how to notify study personnel if symptoms of infusion reaction occur after any PDR001 infusion. Further instructions for the preparation and dispensation of PDR001 are described in the Pharmacist Instruction Manual.

Placebo will be a Dextrose 5% in water (D5W) infusion supplied by the site.

Study drugs must be received at the study site by a designated person, handled and stored safely and properly, and kept in a secure location to which only the unblinded pharmacist, unblinded CRA, or unblinded designated staff have access. Upon receipt, the study drugs should be stored according to the instructions specified on the drug labels. Storage conditions must be adequately monitored and appropriate temperature logs maintained as source data. Appropriate documentation of the subject specific dispensing process must be maintained. Bulk medication labels will be in the local language, will comply with the legal requirements of each country, and will include storage conditions for the drug but no information about the subject.

Note: To maintain the blind, the investigational treatments will be prepared by an unblinded pharmacist/designee, and the bags will be covered by a non-translucent, opaque sleeve. Only study personnel blinded to treatment allocation must administer PDR001 or placebo.

All drug supplies are to be used only for this protocol and not for any other purpose. Unless specifically instructed by Novartis, the Investigator must not destroy any drug labels, or any partly used or unused drug supply. Only after receiving a written authorization by Novartis, the Investigator/designee will send all the unused and partly used drug supplies as well as the empty containers to the address provided at the time of authorization for destruction.

6.6.1 Study treatment packaging and labeling

PDR001 will be provided as global clinical open label supply and will be packed and labeled under the responsibility of Novartis, Drug Supply Management.

PDR001 in different formulations may be used once available.

PDR001 (powder for solution for infusion or liquid in vial) will be provided as global clinical open label supply and will be packed and labeled under the responsibility of Novartis, Drug Supply Management.

Dabrafenib and trametinib will be either sourced as local commercial supply or provided as global clinical open label supply. Global clinical open label supply will be provided in bottles.

Study treatment labels will comply with the legal requirements of each country and will include storage conditions.

Novartis Drug Supply Management supplied treatments will also contain a unique medication number (corresponding to study treatment and strength).

Responsible site personnel will identify the study treatment package(s) to dispense by the medication number(s) assigned by IRT to the subject. If the label has 2 parts (base plus tear-off

label), immediately before dispensing the package to the subject, site personnel will detach the tear-off part of the label from the package and affix it to the subject's source document.

Table 6-27 Packaging and labeling

Study treatments	Supply	Storage
PDR001	Centrally supplied by Novartis	Refer to study treatment label
Dabrafenib (DRB436)	Novartis or locally	Refer to study treatment label or local product information
Trametinib (TMT212)	Novartis or locally	Refer to study treatment label or local product information

6.6.2 Drug supply and storage

Study treatments must be received by designated personnel at the study site, handled and stored safely and properly, and kept in a secured location to which only the investigator and designated site personnel have access. Upon receipt, the study treatment should be stored according to the instructions specified on the drug labels and in the latest PDR001 Investigator's Brochure, Dabrafenib Investigator's Brochure, and Trametinib Investigator's Brochure.

6.6.3 Study drug compliance and accountability

6.6.3.1 Study drug compliance

Compliance will be assessed by the investigator and/or study personnel at each subject visit and information provided by the subject and/or caregiver will be captured in the Drug Accountability Form. This information must be captured in the source document at each subject visit. Dose changes of dabrafenib and trametinib and interruptions of PDR001, dabrafenib and trametinib must be specifically documented in the subject source documents and eCRF.

6.6.3.2 Study drug accountability

The investigator or designee must maintain an accurate record of the shipment and dispensing of study treatment in a drug accountability log. Drug accountability will be noted by the CRA (parts 1 and 2) or by the unblinded CRA (part 3) during site visits and at the completion of the study. Subjects will be asked to return all unused study treatment and packaging on a regular basis, at the end of the study or at the time of study treatment discontinuation.

At study close-out, and, as appropriate during the course of the study, the investigator will return all used and unused study treatment, packaging, drug labels, and a copy of the completed drug accountability log to the Novartis monitor or to the Novartis address provided in the investigator folder at each site.

6.6.3.3 Handling of other study treatment

Not applicable.

6.6.4 Disposal and destruction

Any waste material should be disposed of in accordance with local regulations and according to institutional guidelines. If local regulations disallow storage of empty or pierced vials at the site, the site is able to destroy the vials as per local requirements and procedures. Documentation regarding the local regulations and the destruction of the empty vials will be provided to the Sponsor to ensure full drug accountability.

7 Visit schedule and assessments

7.1 Study flow and visit schedule

Table 7-1 (Safety run-in part), Table 7-2 (Biomarker cohort), Table 7-3 (Randomized part) and Table 7-4 (Crossover part) list all of the assessments and indicates with an "X" (or otherwise specified) the visits when they are performed. All data obtained from these assessments must be supported in the subject's source documentation. The table indicates which assessments produce data to be entered into the database (D) or remain in source documents only (S) ("Category" column).

Every effort must be made to follow the schedule of assessments within the windows outlined in the protocol.

- For Cycle 1, all assessments have a ± 3 day window unless otherwise indicated.
- After Cycle 1, all assessments have a ± 7 day window, unless otherwise indicated.

Additional assessment may be performed as clinically indicated.

If study drug is being held due to toxicity, then the scheduled visits and assessments should still be performed per protocol (except dose administration), unless otherwise specified.

Table 7-1 Visit evaluation schedule: Safety run-In part (Part 1)

				Treatment (Cycle = 28 days)										Follow-Up Period						
	Category	Protocol Section	Screening/Baseline	Cycle 1			Cycle 2			Cycle 3		Subsequent Cycles Day 1	End of Study Treatment (EoT)	30- Day Safety Follow Up	60-Day Safety Follow Up	90-Day Safety Follow Up	120-Day Safety Follow Up	150 Day Safety Follow Up	End of Post Treatment Follow Up	Survival Follow Up
**Day of cycle**			**-28 to -1**	**1**	**8**	**15**	**1**	**8**	**15**	**1**	**15**	**1**								
**Obtain Informed Consent**																				
Study ICF	D	7.1.2	X																	
Pharmacogenetic ICF	D	7.2.4.2.1	X																	
IRT Registration-Enrollment	S	7.1.2.1	X																	
Treatment beyond progression ICF	D	6.1.5.1		X (subjects who meet the criteria outlined in Section 6.1.5.1)																
**Patient History**																				
Demography	D	7.1.2.3	X																	
Inclusion/exclusion	D	5.2, 5.3	X																	
Past and current medical conditions including cardiovascular medical history and risk factors	D	7.1.2.3	X																	
Diagnosis and extent of cancer	D	7.1.2.3	X																	

Supplemental material BMJ Publishing Group Limited (BMJ) disclaims all liability and responsibility arising from any reliance placed on this supplemental material which has been supplied by the author(s) J Immunother Cancer

	Category	Protocol Section	Screening/Baseline	Treatment (Cycle = 28 days)										Follow-Up Period						
				Cycle 1			Cycle 2			Cycle 3		Subsequent Cycles Day 1	End of Study Treatment (EoT)	30- Day Safety Follow Up	60-Day Safety Follow Up	90-Day Safety Follow Up	120-Day Safety Follow Up	150 Day Safety Follow Up	End of Post Treatment Follow Up	Survival Follow Up
**Day of cycle**			**-28 to -1**	**1**	**8**	**15**	**1**	**8**	**15**	**1**	**15**	**1**								
Alcohol History	D	7.1.2.3	X																	
Smoking History	D	7.1.2.3	X																	
HIV History (Note: for Germany only, a mandatory HIV test is required during screening using a local test)	S	7.1.2.3	X																	
Prior antineoplastic therapies (meds, surgery, radiation)	D	7.1.2.3	X																	
Prior/concomitant medications	D	7.1.2.3	From 30 days prior to starting study treatment until 150 day safety follow-up or start of new antineoplastic medication, whichever occurs first. If new antineoplastic medication is started then only medications relative to the AEs/SAEs that are reported should be collected.																	
Antineoplastic therapies since discontinuation of study treatment	D	7.1.2.3											X	X	X	X	X	X		X
**Enrollment**																				
Drug Supply (IRT)	S	7.1.2.1	X																	

	Category	Protocol Section	Screening/Baseline	Treatment (Cycle = 28 days)										Follow-Up Period						
				Cycle 1			Cycle 2			Cycle 3		Subsequent Cycles Day 1	End of Study Treatment (EoT)	30- Day Safety Follow Up	60-Day Safety Follow Up	90-Day Safety Follow Up	120-Day Safety Follow Up	150 Day Safety Follow Up	End of Post Treatment Follow Up	Survival Follow Up
**Day of cycle**			**-28 to -1**	**1**	**8**	**15**	**1**	**8**	**15**	**1**	**15**	**1**								
Disposition Assessment (at the end of each study phase)	D	7.1.4	X										X						X	
**Physical Examination**																				
Physical Examination (complete)	S	7.2.2.1	X	As clinically indicated																
Physical Examination (short)	S	7.2.2.1		X			X			X		X	X	30 days from the last dose of PDR001 and every month for 6 months from the last dose of dabrafenib						
Performance Status (ECOG)	D	7.2.2.4	X	X			X			X		X	X	X						
Height	D	7.2.2.3	X																	
Weight	D	7.2.2.3	X	X			X			X		X	X	X						
Vital signs	D	7.2.2.2	X	X			X			X		X	X	X						
Ophthalmic examination	D	7.2.2.5	X				X			As clinically indicated										
12-lead ECG for DL1	D	7.2.2.7.1	X				X			As clinically indicated			X							

	Category	Protocol Section	Screening/Baseline	Treatment (Cycle = 28 days)										Follow-Up Period						
				Cycle 1			Cycle 2			Cycle 3		Subsequent Cycles Day 1	End of Study Treatment (EoT)	30- Day Safety Follow Up	60-Day Safety Follow Up	90-Day Safety Follow Up	120-Day Safety Follow Up	150 Day Safety Follow Up	End of Post Treatment Follow Up	Survival Follow Up
**Day of cycle**			**-28 to -1**	**1**	**8**	**15**	**1**	**8**	**15**	**1**	**15**	**1**								
12-lead ECG for DL-1a or DL-1b	D	7.2.2.7.1	X							X		As clinically indicated	X							
Cardiac function (MUGA or ECHO)	D	7.2.2.7.2	X				X					C4D1 and every 12 weeks and as clinically indicated	X							
**Laboratory Assessments**																				
Hematology for DL1	D	7.2.2.6.1	X	X	X	X	X		X	X	X	X	X	X						
Hematology for DL-1a	D	7.2.2.6.1	X	X	X	X	X		X	X	X	X and at C4D15	X	X						
Hematology for DL-1b	D	7.2.2.6.1	X	X		X	X	X	X	X	X	X	X	X						
Chemistry for DL1	D	7.2.2.6.1	X	X	X	X	X		X	X	X	X	X	X						
Chemistry for DL-1a	D	7.2.2.6.1	X	X	X	X	X		X	X	X	X and at C4D15	X	X						
Chemistry for DL-1b	D	7.2.2.6.1	X	X		X	X	X	X	X	X	X	X	X						
Thyroid function- TSH	D	7.2.2.6.3	X	X			X			X		X	X	X						
Thyroid Panel- Free T3 and Free T4	D	7.2.2.6.3	X									Only if TSH is abnormal								
Coagulation	D	7.2.2.6.4	X	C1D1, every 2 cycles for the first 12 cycles and every 3 cycles thereafter as clinically indicated									As clinically indicated							
Urinalysis (microscopy or macroscopic)	S	7.2.2.6.5	X	X			X			X		X	X	X						

	Category	Protocol Section	Screening/Baseline	Treatment (Cycle = 28 days)										Follow-Up Period						
				Cycle 1			Cycle 2			Cycle 3		Subsequent Cycles Day 1	End of Study Treatment (EoT)	30- Day Safety Follow Up	60-Day Safety Follow Up	90-Day Safety Follow Up	120-Day Safety Follow Up	150 Day Safety Follow Up	End of Post Treatment Follow Up	Survival Follow Up
**Day of cycle**			**-28 to -1**	**1**	**8**	**15**	**1**	**8**	**15**	**1**	**15**	**1**								
Urine pregnancy test	S	7.2.2.6.6					X			X		X			X	X	X			
Serum Pregnancy	S	7.2.2.6.6	X										X	X				X		
Hepatitis Testing	D	7.2.2.6.7	X	As Clinically indicated																
For Germany only: local HIV Testing	S	7.2.2.6.9	X																	
Cytokines	D	7.2.2.6.8	X	Anytime when a suspected cytokine release syndrome occurs, immediately after the AE, and one week after occurrence of the AE																

				**Treatment (Cycle = 28 days)**										**Follow-Up Period**						
	**Category**	**Protocol Section**	**Screening/Baseline**	**Cycle 1**			**Cycle 2**			**Cycle 3**		**Subsequent Cycles Day 1**	**End of Study Treatment (EoT)**	**30- Day Safety Follow Up**	**60-Day Safety Follow Up**	**90-Day Safety Follow Up**	**120-Day Safety Follow Up**	**150 Day Safety Follow Up**	**End of Post Treatment Follow Up**	**Survival Follow Up**
**Day of cycle**			**-28 to -1**	**1**	**8**	**15**	**1**	**8**	**15**	**1**	**15**	**1**								
**Imaging**																				
Tumor evaluation per RECIST 1.1	D	7.2.1	X									**During treatment:** C4D1 (± 7 days), every 8 weeks, then from C22D1 (± 7 days), every 12 weeks thereafter<br>**End of Treatment (EOT):** if a scan was not conducted within 30 days prior to end of study treatment<br>**Efficacy follow-up:** Every 12 weeks starting from the last assessment until documented disease progression per RECIST 1.1 (unless meeting criteria for treatment beyond progression), withdrawal of consent, lost to follow-up, or death irrespective of start of new anti-neoplastic therapy.<br>Refer to Section 7.2.1.2 for details on additional tumor assessments to perform in order to confirm response or allow for further central analysis.								

	Category	Protocol Section	Screening/Baseline	Treatment (Cycle = 28 days)										Follow-Up Period						
				Cycle 1			Cycle 2			Cycle 3		Subsequent Cycles Day 1	End of Study Treatment (EoT)	30- Day Safety Follow Up	60-Day Safety Follow Up	90-Day Safety Follow Up	120-Day Safety Follow Up	150 Day Safety Follow Up	End of Post Treatment Follow Up	Survival Follow Up
**Day of cycle**			**-28 to -1**	**1**	**8**	**15**	**1**	**8**	**15**	**1**	**15**	**1**								
Chest, abdomen and pelvis CT or MRI (with contrast enhancement)	D	7.2.1	X									**During treatment:** C4D1 (± 7 days), every 8 weeks, then from C22D1 (± 7 days), every 12 weeks thereafter<br>**End of Treatment (EOT):** if a scan was not conducted within 30 days prior to end of study treatment<br>**Efficacy follow-up:** Every 12 weeks starting from the last assessment until documented disease progression per RECIST 1.1 (unless meeting criteria for treatment beyond progression), withdrawal of consent, lost to follow-up, or death irrespective of start of new anti-neoplastic therapy.<br>Refer to Section 7.2.1.2 for details on additional tumor assessments to perform in order to confirm response or allow for further central analysis.								
Brain imaging (CT or MRI)	D	7.2.1	X	If clinically indicated or positive at screening, follow same schedule as CT/MRI of chest, abdomen and pelvis																
Whole body bone scan	D	7.2.1	If clinically indicated																	
Localized bone CT, MRI or X-ray	D	7.2.1	Only for lesions on whole body scan that are not visible on the chest, abdomen and pelvis scans. If lesions were documented at screening, follow same schedule as CT/MRI of chest, abdomen and pelvis																	
CT or MRI of other metastatic sites (e.g., neck)	D	7.2.1	If clinically indicated or positive at screening, follow same schedule as CT/MRI of chest, abdomen and pelvis																	

	Category	Protocol Section	Screening/Baseline	Treatment (Cycle = 28 days)										Follow-Up Period						
				Cycle 1			Cycle 2			Cycle 3		Subsequent Cycles Day 1	End of Study Treatment (EoT)	30- Day Safety Follow Up	60-Day Safety Follow Up	90-Day Safety Follow Up	120-Day Safety Follow Up	150 Day Safety Follow Up	End of Post Treatment Follow Up	Survival Follow Up
**Day of cycle**			**-28 to -1**	**1**	**8**	**15**	**1**	**8**	**15**	**1**	**15**	**1**								
Color Digital Photography (including a metric ruler) for cutaneous lesions	D	7.2.1	X									**During treatment:** C4D1 (± 7 days), every 8 weeks, then from C22D1 (± 7 days), every 12 weeks thereafter<br>**End of Treatment (EOT):** if a scan was not conducted within 30 days prior to end of study treatment<br>**Efficacy follow-up:** Every 12 weeks starting from the last assessment until documented disease progression per RECIST 1.1 (unless meeting criteria for treatment beyond progression), withdrawal of consent, lost to follow-up, or death irrespective of start of new anti-neoplastic therapy.<br>Refer to Section 7.2.1.2 for details on additional tumor assessments to perform in order to confirm response or allow for further central analysis.								
Body Fluid/Tissue	D	7.2.1	Needle aspiration, biopsy or any other form of collection (e.g., paracentesis, punctuation) performed for any reason																	
**Safety**																				
Adverse events	D	7.2.2, 8.1	Suspected AEs up to Day 150; non-suspected AEs up to Day 150 or start of new post treatment anti-neoplastic medication, whichever is sooner.<br>Monitoring of new cutaneous malignancies should continue every month for 6 months following discontinuation of dabrafenib or until initiation of another antineoplastic therapy (refer to Section 6.3.5.3).																	
Serious adverse events	D	8.2.2	Suspected SAEs up to Day 150 and beyond; non-suspected SAEs up to Day 150 or start of new post treatment anti-neoplastic medication, whichever is sooner.																	

	Category	Protocol Section	Screening/Baseline	Treatment (Cycle = 28 days)										Follow-Up Period						
				Cycle 1			Cycle 2			Cycle 3		Subsequent Cycles Day 1	End of Study Treatment (EoT)	30- Day Safety Follow Up	60-Day Safety Follow Up	90-Day Safety Follow Up	120-Day Safety Follow Up	150 Day Safety Follow Up	End of Post Treatment Follow Up	Survival Follow Up
**Day of cycle**			**-28 to -1**	**1**	**8**	**15**	**1**	**8**	**15**	**1**	**15**	**1**								
**Biomarkers**																				
Blood for germline DNA assessment	D	7.2.4.2.1	X																	
Tumor (archival or new) tissue for central *BRAF* V600 mutation testing, assessment of IO markers and potential companion diagnostic bridging study.<br>Note: if local *BRAF* V600 mutation result is not available, sample will be used for screening and should be sent for central testing by Day -20	D	7.2.4.1.1	X																	

Supplemental material BMJ Publishing Group Limited (BMJ) disclaims all liability and responsibility arising from any reliance placed on this supplemental material which has been supplied by the author(s) J Immunother Cancer

	Category	Protocol Section	Screening/Baseline	Treatment (Cycle = 28 days)										Follow-Up Period						
				Cycle 1			Cycle 2			Cycle 3		Subsequent Cycles Day 1	End of Study Treatment (EoT)	30- Day Safety Follow Up	60-Day Safety Follow Up	90-Day Safety Follow Up	120-Day Safety Follow Up	150 Day Safety Follow Up	End of Post Treatment Follow Up	Survival Follow Up
**Day of cycle**			**-28 to -1**	**1**	**8**	**15**	**1**	**8**	**15**	**1**	**15**	**1**								
Optional newly acquired biopsy	D	7.2.4.1.2	X							X (anytime during Cycle 3)			**Unscheduled:** At disease progression per RECIST 1.1 and/or at a subsequent PD per response criteria for immunotherapy if subject continues treatment post progression, prior to the start of new antineoplastic therapy							
Blood plasma for ctDNA isolation	D	7.2.4.2.2		X			X			X			**Unscheduled:** At disease progression per RECIST 1.1 and/or at a subsequent PD per response criteria for immunotherapy if subject continues treatment post progression, prior to the start of new antineoplastic therapy							
Blood plasma for ctRNA isolation	D	7.2.4.2.3		X			X			X			**Unscheduled:** At disease progression per RECIST 1.1and/or at a subsequent PD per response criteria for immunotherapy if subject continues treatment post progression, prior to the start of new antineoplastic therapy							
Blood for cytokines	D	7.2.4.2.5		X			X			X			**Unscheduled:** At disease progression per RECIST 1.1 and/or at a subsequent PD per response criteria for immunotherapy if subject continues treatment post progression, prior to the start of new antineoplastic therapy							

	Category	Protocol Section	Screening/Baseline	**Treatment (Cycle = 28 days)**										**Follow-Up Period**						
				**Cycle 1**			**Cycle 2**			**Cycle 3**		**Subsequent Cycles Day 1**	**End of Study Treatment (EoT)**	**30- Day Safety Follow Up**	**60-Day Safety Follow Up**	**90-Day Safety Follow Up**	**120-Day Safety Follow Up**	**150 Day Safety Follow Up**	**End of Post Treatment Follow Up**	**Survival Follow Up**
**Day of cycle**			**-28 to -1**	**1**	**8**	**15**	**1**	**8**	**15**	**1**	**15**	**1**								
Blood for PBMCs	D	7.2.4.2.4		X			X			X			**Unscheduled:** At disease progression per RECIST 1.1 and/or at a subsequent PD per response criteria for immunotherapy if subject continues treatment post progression, prior to the start of new antineoplastic therapy							
**Study Administration**																				
PDR001 infusion	D	6.1.1.1		DL1: PDR001 400mg IV every 4 weeks<br>DL-1a: PDR001 400mg IV every 8 weeks<br>DL-1b: at C2D1, PDR001 400mg IV every 4 weeks																
Trametinib/Dabrafenib combination	D	6.1.1.1		Trametinib orally once daily and Dabrafenib orally twice daily for Days 1-28 of a 28-day cycle																
PK sampling of PDR001 (for DL1 or DL-1a)	D	7.2.3.1.1		X	X	X	X		X	X		Day 1 of cycles 4-12, every 6 cycles after C12D1	X	X				X		
PK sampling of PDR001 (for DL-1b)	D	7.2.3.1.1					X		X	X		Day 1 of cycles 4-12, every 6 cycles after C12D1	X	X				X		
PK sampling of trametinib and dabrafenib	D	7.2.3.1.2		X		X	X		X	X		Day 1 of cycles 4-12, every 6 cycles after C12D1	X							

	Category	Protocol Section	Screening/Baseline	Treatment (Cycle = 28 days)										Follow-Up Period						
				Cycle 1			Cycle 2			Cycle 3		Subsequent Cycles Day 1	End of Study Treatment (EoT)	30- Day Safety Follow Up	60-Day Safety Follow Up	90-Day Safety Follow Up	120-Day Safety Follow Up	150 Day Safety Follow Up	End of Post Treatment Follow Up	Survival Follow Up
**Day of cycle**			**-28 to -1**	**1**	**8**	**15**	**1**	**8**	**15**	**1**	**15**	**1**								
Immunogenicity (IG) sampling (For DL1 and DL-1a)	D	7.2.3.1.1		X			X			X		Day 1 of cycles 4-12, every 6 cycles after C12D1	X	X				X		
Immunogenicity (IG) sampling (for DL-1b)	D	7.2.3.1.1					X			X		Day 1 of cycles 4-12, every 6 cycles after C12D1	X	X				X		
Survival Follow-up	D	7.1.8																		X

Table 7-2 Visit evaluation schedule: Biomarker cohort (Part 2)

	Category	Protocol Section	Screening/Baseline	Treatment (Cycle = 28 days) Cycle 1		Cycle 2		Cycle 3		Subsequent Cycles Day 1	End of Study Treatment (EoT)	Follow-Up Period 30-Day Safety Follow Up	60-Day Safety Follow Up	90-Day Safety Follow Up	120-Day Safety Follow Up	150-Day Safety Follow Up	End of Post Treatment Follow Up	Survival Follow Up
**Day of cycle**			**-28 to -1**	**1**	**15**	**1**	**15**	**1**	**15**	**1**								
**Obtain Informed Consent**																		
Study ICF	D	7.1.2	X															
Pharmacogenetic ICF	D	7.2.4.2.1	X															
IRT Registration	S	7.1.2.1	X															
Treatment beyond progression ICF	D	6.1.5.1		X (subjects who meet the criteria outlined in Section 6.1.5.1)														
**Patient History**																		
Demography	D	7.1.2.3	X															
Inclusion/exclusion	D	5.2, 5.3	X															
Past and current medical conditions including cardiovascular medical history and risk factors	D	7.1.2.3	X															
Diagnosis and extent of cancer	D	7.1.2.3	X															
Alcohol History	D	7.1.2.3	X															
Smoking History	D	7.1.2.3	X															

	Category	Protocol Section	Screening/Baseline	Treatment (Cycle = 28 days)								Follow-Up Period						
				Cycle 1		Cycle 2		Cycle 3		Subsequent Cycles Day 1	End of Study Treatment (EoT)	30-Day Safety Follow Up	60-Day Safety Follow Up	90-Day Safety Follow Up	120-Day Safety Follow Up	150-Day Safety Follow Up	End of Post Treatment Follow Up	Survival Follow Up
**Day of cycle**			**-28 to -1**	**1**	**15**	**1**	**15**	**1**	**15**	**1**								
HIV History (Note: for Germany only, a mandatory HIV test is required during screening using a local test)	S	7.1.2.3	X															
Prior antineoplastic therapies (meds, surgery, radiation)	D	7.1.2.3	X															
Prior/concomitant medications;	D	7.1.2.3	From 30 days prior to starting study treatment until 150 Day safety follow-up or start of new antineoplastic medication, whichever is sooner. If new antineoplastic medication is started then only medications relative to the AEs/SAEs that are reported should be collected															
Antineoplastic therapies since discontinuation of study treatment	D	7.1.2.3									X	X	X	X	X	X		X
**Enrollment**																		
Drug Supply (IRT)	S	7.1.2.1	X															
Disposition Assessment (at the end of each study phase)	D	7.1.4	X								X						X	
**Physical Examination**																		
Physical Examination (complete)	S	7.2.2.1	X	As clinically indicated														

	Category	Protocol Section	Screening/Baseline	**Treatment (Cycle = 28 days)**								**Follow-Up Period**						
				**Cycle 1**		**Cycle 2**		**Cycle 3**		**Subsequent Cycles Day 1**	**End of Study Treatment (EoT)**	**30-Day Safety Follow Up**	**60-Day Safety Follow Up**	**90-Day Safety Follow Up**	**120-Day Safety Follow Up**	**150-Day Safety Follow Up**	**End of Post Treatment Follow Up**	**Survival Follow Up**
**Day of cycle**			**-28 to -1**	**1**	**15**	**1**	**15**	**1**	**15**	**1**								
Physical Examination (short)	S	7.2.2.1		X		X		X		X	X	30 days from the last dose of PDR001 and every month for 6 months from the last dose of dabrafenib						
Performance Status (ECOG)	D	7.2.2.4	X	X		X		X		X	X	X						
Height	D	7.2.2.3	X															
Weight	D	7.2.2.3	X	X		X		X		X	X	X						
Vital signs	D	7.2.2.2	X	X		X		X		X	X	X						
Ophthalmic examination	D	7.2.2.5	X			X		As clinically indicated										
12-lead ECG if DL1 is determined to be the regimen	D	7.2.2.7.1	X			X		As clinically indicated			X							
12-lead ECG if DL-1a or DL-1b is determined to be the regimen	D	7.2.2.7.1	X					X		As clinically indicated	X							
Cardiac function (MUGA or ECHO)	D	7.2.2.7.2	X			X				X C4D1, every 12 weeks and as clinically indicated	X							

Supplemental material BMJ Publishing Group Limited (BMJ) disclaims all liability and responsibility arising from any reliance placed on this supplemental material which has been supplied by the author(s) J Immunother Cancer

	Category	Protocol Section	Screening/Baseline	Treatment (Cycle = 28 days)								Follow-Up Period						
				Cycle 1		Cycle 2		Cycle 3		Subsequent Cycles Day 1	End of Study Treatment (EoT)	30-Day Safety Follow Up	60-Day Safety Follow Up	90-Day Safety Follow Up	120-Day Safety Follow Up	150-Day Safety Follow Up	End of Post Treatment Follow Up	Survival Follow Up
**Day of cycle**			**-28 to -1**	**1**	**15**	**1**	**15**	**1**	**15**	**1**								
**Laboratory Assessments**																		
Hematology	D	7.2.2.6.1	X	X	X	X	X	X	X	X	X	X						
Chemistry	D	7.2.2.6.2	X	X	X	X	X	X	X	X	X	X						
Thyroid function-TSH	D	7.2.2.6.3	X	X		X		X		X	X	X						
Thyroid Panel- FreeT3 and Free T4	D	7.2.2.6.3	X							Only if TSH is abnormal								
Coagulation	D	7.2.2.6.4	X	C1D1, every 2 cycles for the first 12 cycles and every 3 cycles thereafter as clinically indicated							As clinically indicated							
Urinalysis (microscopy and macroscopic)	S	7.2.2.6.5	X	X		X		X		X	X	X						
Serum Pregnancy Test	S	7.2.2.6.6	X								X	X				X		
Urine Pregnancy Test	S	7.2.2.6.6				X		X		X			X	X	X			
Hepatitis testing	D	7.2.2.6.7	X	As clinically indicated														
Cytokines	D	7.2.2.6.8	X	Anytime when a suspected cytokine release syndrome occurs, immediately after the AE, and one week after occurrence of the AE														
For Germany only: local HIV testing	S	7.2.2.6.9	X															

				**Treatment (Cycle = 28 days)**								**Follow-Up Period**						
	**Category**	**Protocol Section**	**Screening/Baseline**	**Cycle 1**		**Cycle 2**		**Cycle 3**		**Subsequent Cycles Day 1**	**End of Study Treatment (EoT)**	**30-Day Safety Follow Up**	**60-Day Safety Follow Up**	**90-Day Safety Follow Up**	**120-Day Safety Follow Up**	**150-Day Safety Follow Up**	**End of Post Treatment Follow Up**	**Survival Follow Up**
**Day of cycle**			**-28 to -1**	**1**	**15**	**1**	**15**	**1**	**15**	**1**								
**Imaging**																		
Tumor evaluation per RECIST 1.1	D	7.2.1	X							**During treatment:** C4D1 (± 7 days), every 8 weeks, then from C22D1 (± 7 days), every 12 weeks thereafter<br>**End of Treatment (EOT):** if a scan was not conducted within 30 days prior to end of study treatment.<br>**Efficacy follow-up:** Every 12 weeks starting from the last assessment until documented disease progression per RECIST 1.1 (unless meeting criteria for treatment beyond progression), withdrawal of consent, lost to follow-up, or death irrespective of start of new anti-neoplastic therapy<br>Refer to Section 7.2.1.2 for details on additional tumor assessments to perform in order to confirm response or allow for further central analysis.								

				**Treatment (Cycle = 28 days)**								**Follow-Up Period**						
	**Category**	**Protocol Section**	**Screening/Baseline**	**Cycle 1**		**Cycle 2**		**Cycle 3**		**Subsequent Cycles Day 1**	**End of Study Treatment (EoT)**	**30-Day Safety Follow Up**	**60-Day Safety Follow Up**	**90-Day Safety Follow Up**	**120-Day Safety Follow Up**	**150-Day Safety Follow Up**	**End of Post Treatment Follow Up**	**Survival Follow Up**
**Day of cycle**			**-28 to -1**	**1**	**15**	**1**	**15**	**1**	**15**	**1**								
Chest, abdomen and pelvis CT or MRI (with contrast enhancement)	D	7.2.1	X							**During treatment:** C4D1 (± 7 days), every 8 weeks, then from C22D1 (± 7 days), every 12 weeks thereafter<br>**End of Treatment (EOT):** if a scan was not conducted within 30 days prior to end of study treatment<br>**Efficacy follow-up:** Every 12 weeks starting from the last assessment until documented disease progression per RECIST 1.1 (unless meeting criteria for treatment beyond progression), withdrawal of consent, lost to follow-up, or death irrespective of start of new anti-neoplastic therapy<br>Refer to Section 7.2.1.2 for details on additional tumor assessments to perform in order to confirm response or allow for further central analysis.								
Brain imaging (CT or MRI)	D	7.2.1	X	If clinically indicated or positive at screening, follow same schedule as CT/MRI of chest, abdomen and pelvis														
Whole body bone scan	D	7.2.1	If clinically indicated															
Localized bone CT, MRI or X-ray	D	7.2.1	Only for lesions on whole body scan that are not visible on the chest, abdomen and pelvis scans. If lesions were documented at screening, follow same schedule as CT/MRI of chest, abdomen and pelvis															
CT or MRI of other metastatic sites (e.g., neck)	D	7.2.1	If clinically indicated or positive at screening, follow same schedule as CT/MRI of chest, abdomen and pelvis															

				Treatment (Cycle = 28 days)								Follow-Up Period						
				Cycle 1		Cycle 2		Cycle 3										
	**Category**	**Protocol Section**	**Screening/Baseline**							**Subsequent Cycles Day 1**	**End of Study Treatment (EoT)**	**30-Day Safety Follow Up**	**60-Day Safety Follow Up**	**90-Day Safety Follow Up**	**120-Day Safety Follow Up**	**150-Day Safety Follow Up**	**End of Post Treatment Follow Up**	**Survival Follow Up**
**Day of cycle**			**-28 to -1**	**1**	**15**	**1**	**15**	**1**	**15**	**1**								
Color Digital Photography (including a metric ruler) of skin lesions	D	7.2.1	X							**During treatment:** C4D1 (± 7 days), every 8 weeks, then from C22D1 (± 7 days), every 12 weeks thereafter<br>**End of Treatment (EOT):** if a scan was not conducted within 30 days prior to end of study treatment.<br>**Efficacy follow-up:** Every 12 weeks starting from the last assessment until documented disease progression per RECIST 1.1 (unless meeting criteria for treatment beyond progression), withdrawal of consent, lost to follow-up, or death irrespective of start of new anti-neoplastic therapy<br>Refer to Section 7.2.1.2 for details on additional tumor assessments to perform in order to confirm response or allow for further central analysis.								
Body Fluid/Tissue	D	7.2.1	Needle aspiration, biopsy or any other form of collection (e.g., paracentesis, punctuation) performed for any reason															
**Safety**																		
Adverse events	D	7.2.2, 8.1	Suspected AEs up to Day 150; non-suspected AEs up to Day 150 or start of new post treatment anti-neoplastic medication, whichever is sooner.<br>Monitoring of new cutaneous malignancies should continue every month for 6 months following discontinuation of dabrafenib or until initiation of another antineoplastic therapy (refer to Section 6.3.5.3).															
Serious adverse events	D	8.2	Suspected SAEs up to Day 150 and beyond; non-suspected SAEs up to Day 150 or start of new post treatment anti-neoplastic medication, whichever is sooner.															

				Treatment (Cycle = 28 days)								Follow-Up Period						
	Category	Protocol Section	Screening/Baseline	Cycle 1		Cycle 2		Cycle 3		Subsequent Cycles Day 1	End of Study Treatment (EoT)	30-Day Safety Follow Up	60-Day Safety Follow Up	90-Day Safety Follow Up	120-Day Safety Follow Up	150-Day Safety Follow Up	End of Post Treatment Follow Up	Survival Follow Up
**Day of cycle**			**-28 to -1**	**1**	**15**	**1**	**15**	**1**	**15**	**1**								
**Biomarkers**																		
Newly acquired tumor tissue for central *BRAF* V600 mutation testing, assessment of IO markers and potential companion diagnostic bridging study. Sampling schedule if DL1 or DL-1a is determined to be the regimen.<br>Note: if local *BRAF* V600 mutation result is not available, the sample collected at screening/baseline will be used for screening and should be sent for central testing by Day -20	D	7.2.4.1.1	X		X			X (Anytime during Cycle 3)			**Unscheduled:** At disease progression per RECIST 1.1 and/or at a subsequent PD per response criteria for immunotherapy if subject continues treatment post progression, prior to the start of new antineoplastic therapy							

	Category	Protocol Section	Screening/Baseline	Treatment (Cycle = 28 days)								Follow-Up Period						
				Cycle 1		Cycle 2		Cycle 3		Subsequent Cycles Day 1	End of Study Treatment (EoT)	30-Day Safety Follow Up	60-Day Safety Follow Up	90-Day Safety Follow Up	120-Day Safety Follow Up	150-Day Safety Follow Up	End of Post Treatment Follow Up	Survival Follow Up
**Day of cycle**			**-28 to -1**	**1**	**15**	**1**	**15**	**1**	**15**	**1**								
Newly acquired tumor tissue for central *BRAF* V600 mutation testing, assessment of immunemarkers and potential companion diagnostic bridging study. Sampling schedule if DL-1b is determined to be the regimen.	D	7.2.4.1.1	X		X					X (Anytime during Cycle 4)	**Unscheduled:** At disease progression per RECIST 1.1 and/or at a subsequent PD per response criteria for immunotherapy if subject continues treatment post progression, prior to the start of new antineoplastic therapy							
Blood for germline DNA	D	7.2.4.2.1	X															
Blood for cytokines sampling (if DL1 or DL-1a is determined to be the regimen)	D	7.2.4.2.5		X		X		X			**Unscheduled:** At disease progression per RECIST 1.1 and/or at a subsequent PD per response criteria for immunotherapy if subject continues treatment post progression, prior to the start of new antineoplastic therapy							
Blood for cytokines sampling (if DL-1b is determined to be the regimen)	D	7.2.4.2.5		X		X				X C4D1 only	**Unscheduled:** At disease progression per RECIST 1.1 and/or at a subsequent PD per response criteria for immunotherapy if subject continues treatment post progression, prior to the start of new antineoplastic therapy							

				**Treatment (Cycle = 28 days)**								**Follow-Up Period**						
	**Category**	**Protocol Section**	**Screening/Baseline**	**Cycle 1**		**Cycle 2**		**Cycle 3**		**Subsequent Cycles Day 1**	**End of Study Treatment (EoT)**	**30-Day Safety Follow Up**	**60-Day Safety Follow Up**	**90-Day Safety Follow Up**	**120-Day Safety Follow Up**	**150-Day Safety Follow Up**	**End of Post Treatment Follow Up**	**Survival Follow Up**
**Day of cycle**			**-28 to -1**	**1**	**15**	**1**	**15**	**1**	**15**	**1**								
Blood for PBMCs sampling (if DL1 or DL-1a is determined to be the regimen)	D	7.2.4.2.4		X		X		X			**Unscheduled:** At disease progression per RECIST 1.1 and/or at a subsequent PD per response criteria for immunotherapy if subject continues treatment post progression, prior to the start of new antineoplastic therapy							
Blood for PBMCs sampling (if DL-1b is determined to be the regimen)	D	7.2.4.2.4		X		X				X C4D1 only	**Unscheduled:** At disease progression per RECIST 1.1 and/or at a subsequent PD per response criteria for immunotherapy if subject continues treatment post progression, prior to the start of new antineoplastic therapy							
Blood for ctDNA isolation sampling (if DL1 or DL-1a is determined to be the regimen)	D	7.2.4.2.2		X	X			X			**Unscheduled:** At disease progression per RECIST 1.1 and/or at a subsequent PD per response criteria for immunotherapy if subject continues treatment post progression, prior to the start of new antineoplastic therapy							
Blood for ctDNA isolation sampling (if DL-1b is determined to be the regimen)	D	7.2.4.2.2		X	X					X C4D1 only	**Unscheduled:** At disease progression per RECIST 1.1 and/or at a subsequent PD per response criteria for immunotherapy if subject continues treatment post progression, prior to the start of new antineoplastic therapy							

				**Treatment (Cycle = 28 days)**								**Follow-Up Period**						
	**Category**	**Protocol Section**	**Screening/Baseline**	**Cycle 1**		**Cycle 2**		**Cycle 3**		**Subsequent Cycles Day 1**	**End of Study Treatment (EoT)**	**30-Day Safety Follow Up**	**60-Day Safety Follow Up**	**90-Day Safety Follow Up**	**120-Day Safety Follow Up**	**150-Day Safety Follow Up**	**End of Post Treatment Follow Up**	**Survival Follow Up**
**Day of cycle**			**-28 to -1**	**1**	**15**	**1**	**15**	**1**	**15**	**1**								
Blood for ctRNA isolation sampling (if DL1 or DL-1a is determined to be the regimen)	D	7.2.4.2.3		X	X			X			**Unscheduled:** At disease progression per RECIST 1.1 and/or at a subsequent PD per response criteria for immunotherapy if subject continues treatment post progression, prior to the start of new antineoplastic therapy							
Blood for ctRNA isolation sampling (if DL-1b is determined to be the regimen)	D	7.2.4.2.3		X	X					X C4D1 only	**Unscheduled:** At disease progression per RECIST 1.1 and/or at a subsequent PD per response criteria for immunotherapy if subject continues treatment post progression, prior to the start of new antineoplastic therapy							
Optional Tumor biopsy on treatment	D	7.2.4.1.2	Anytime – depending on investigator assessment															
**Study Administration**																		
PDR001 infusion	D	6.1.1.2		PDR001 400mg IV every 4 weeks (It is possible for additional dosing regimens to be used based on emerging data from part 1.)														
Trametinib/ Dabrafenib combination	D	6.1.1.2		Trametinib orally once daily and Dabrafenib orally twice daily every 28 days														
PK sampling of PDR001 (if DL1 or DL-1a is determined to be the regimen)	D	7.2.3.1.1		X	X	X	X	X		Day 1 of cycles 4 to 12, every 6 cycles after C12D1	X	X				X		

				**Treatment (Cycle = 28 days)**								**Follow-Up Period**						
	**Category**	**Protocol Section**	**Screening/Baseline**	**Cycle 1**		**Cycle 2**		**Cycle 3**		**Subsequent Cycles Day 1**	**End of Study Treatment (EoT)**	**30-Day Safety Follow Up**	**60-Day Safety Follow Up**	**90-Day Safety Follow Up**	**120-Day Safety Follow Up**	**150-Day Safety Follow Up**	**End of Post Treatment Follow Up**	**Survival Follow Up**
**Day of cycle**			**-28 to -1**	**1**	**15**	**1**	**15**	**1**	**15**	**1**								
PK sampling of PDR001 (if DL-1b is determined to be the regimen)	D	7.2.3.1.1				X	X	X		Day 1 of cycles 4 to 12, every 6 cycles after C12D1	X	X				X		
PK sampling of trametinib and dabrafenib	D	7.2.3.1.2		X	X	X	X	X		Day 1 of cycles 4 to 12, every 6 cycles after C12D1	X							
Immunogenicity (IG) sampling	D	7.2.3.1.1		X		X		X		Day 1 of cycles 4 to 12, every 6 cycles after C12D1	X	X				X		
Survival Follow-up	D	7.1.8																X

Table 7-3 Visit evaluation schedule: Double-blind, randomized, placebo-controlled part (Part 3)

	Category	Protocol Section	Screening/Baseline	Treatment (Cycle = 28 days)						Follow-Up Period						
				Cycle 1		Cycle 2		Subsequent cycles Day 1	End of Study Treatment (EoT)	30 Day Safety Follow Up	60- Day Safety Follow Up	90- Day Safety Follow Up	120- Day Safety Follow Up	150- Day Safety Follow Up	End of Post Treatment Follow Up	Survival Follow Up
**Day of cycle**			**-28 to -1**	**1**	**15**	**1**	**15**	**1**	**1**							
**Obtain Informed Consent**																
Study ICF	D	7.1.2	X													
Pharmacogenetic ICF	D	7.2.4.2.1	X													
IRT Registration-Randomization		7.1.2.1	X													
Treatment beyond progression ICF	D	6.1.5.1		X (subjects who meet the criteria outlined in Section 6.1.5.1)												
**Patient History**																
Demography	D	7.1.2.3	X													
Inclusion/exclusion	D	5.2, 5.3	X													
Past and current medical conditions including cardiovascular medical history and risk factors	D	7.1.2.3	X													
Diagnosis and extent of cancer	D	7.1.2.3	X													
Alcohol History	D	7.1.2.3	X													
Smoking History	D	7.1.2.3	X													

	Category	Protocol Section	Screening/Baseline	Treatment (Cycle = 28 days)						Follow-Up Period						
				Cycle 1		Cycle 2		Subsequent cycles Day 1	End of Study Treatment (EoT)	30 Day Safety Follow Up	60- Day Safety Follow Up	90- Day Safety Follow Up	120- Day Safety Follow Up	150- Day Safety Follow Up	End of Post Treatment Follow Up	Survival Follow Up
**Day of cycle**			**-28 to -1**	**1**	**15**	**1**	**15**	**1**	**1**							
HIV History (Note: for Germany only, a mandatory HIV test is required during screening using a local test)	S	7.1.2.3	X													
Prior antineoplastic therapies (meds, surgery, radiation)	D	7.1.2.3	X													
Prior/concomitant medications	D	7.1.2.3	From 30 days prior to starting study treatment until 150 Day safety follow-up or start of new antineoplastic medication, whichever is sooner. If new antineoplastic medication is started then only medications relative to the AEs/SAEs that are reported should be collected													
Antineoplastic therapies since discontinuation of study treatment	D	7.1.2.3								X	X	X	X	X		X
**Enrollment**																
Drug Supply (IRT)	S	7.1.2.1	X													
End of Phase Disposition	D	7.1.4	X						X						X	
**Patient Reported Outcomes**<br>PRO questionnaires should be administered at beginning of the scheduled study visit, prior to any tests performed, treatments administered or receipt of test results.																
EORTC QLQ-C30	D	7.2.5.1	X					**During treatment:** C4D1, every 8 weeks, then from C22D1, every 12 weeks thereafter until documented disease progression per RECIST 1.1<br>**End of treatment (EOT):** if not conducted within 30 days prior to end of study treatment<br>**Unscheduled:** 30 and 60 days post disease progression per RECIST 1.1								

	Category	Protocol Section	Screening/Baseline	Treatment (Cycle = 28 days)						Follow-Up Period						
				Cycle 1		Cycle 2		Subsequent cycles Day 1	End of Study Treatment (EoT)	30 Day Safety Follow Up	60- Day Safety Follow Up	90- Day Safety Follow Up	120- Day Safety Follow Up	150- Day Safety Follow Up	End of Post Treatment [illegible]	Survival Follow Up
**Day of cycle**			**-28 to -1**	**1**	**15**	**1**	**15**	**1**	**1**							
EQ-5D-5L	D	7.2.5.2	X					**During treatment:** C4D1, every 8 weeks, then from C22D1, every 12 weeks thereafter until documented disease progression per RECIST 1.1<br>**End of treatment (EOT):** if not conducted within 30 days prior to end of study treatment<br>**Unscheduled:** 30 and 60 days post disease progression per RECIST 1.1								
FACT-M subscale (16 questions)	D	7.2.5.3	X					**During treatment:** C4D1, every 8 weeks, then from C22D1, every 12 weeks thereafter until documented disease progression per RECIST 1.1<br>**End of treatment (EOT):** if not conducted within 30 days prior to end of study treatment<br>**Unscheduled:** 30 and 60 days post disease progression per RECIST 1.1								
**Physical Examination**																
Physical Examination (complete)	S	7.2.2.1	X	As clinically indicated												
Physical Examination (short)	S	7.2.2.1		X		X		X	X	30 days from the last dose of PDR001/placebo and every month for 6 months from the last dose of dabrafenib						
Performance Status (ECOG)	D	7.2.2.4	X	X		X		X	X	X						
Height	D	7.2.2.3	X													
Weight	D	7.2.2.3	X	X		X		X	X	X						
Vital signs	D	7.2.2.2	X	X		X		X	X	X						

	Category	Protocol Section	Screening/Baseline	Treatment (Cycle = 28 days)						Follow-Up Period						
				Cycle 1		Cycle 2		Subsequent cycles Day 1	End of Study Treatment (EoT)	30 Day Safety Follow Up	60- Day Safety Follow Up	90- Day Safety Follow Up	120- Day Safety Follow Up	150- Day Safety Follow Up	End of Post Treatment Follow Up	Survival Follow Up
**Day of cycle**			**-28 to -1**	**1**	**15**	**1**	**15**	**1**	**1**							
Ophthalmic examination	D	7.2.2.5	X			X		As clinically indicated								
12-lead ECG if DL1 is determined to be the regimen	D	7.2.2.7.1	X			X		As clinically indicated	X							
12-lead ECG if DL-1a or DL-1b is determined to be the regimen	D	7.2.2.7.1	X					C3D1, and as clinically indicated	X							
Cardiac function (MUGA or ECHO)	D	7.2.2.7.2	X			X		C4D1 and every 12 weeks and as clinically indicated	X							
**Laboratory Assessments**																
Hematology	D	7.2.2.6.1	X	X	X	X	X	X	X	X						
Chemistry	D	7.2.2.6.2	X	X	X	X	X	X	X	X						
Thyroid function-TSH	D	7.2.2.6.3	X	X		X		X	X	X						
Thyroid Panel Free T3 and Free T4	D	7.2.2.6.3	X					Only if TSH is abnormal								
Coagulation	D	7.2.2.6.4	X	C1D1, every 2 cycles for the first 12 cycles and every 3 cycles thereafter as clinically indicated					As clinically indicated							
Urinalysis (microscopy and macroscopic)	S	7.2.2.6.5	X	X		X		X	X	X						
Serum Pregnancy Test	S	7.2.2.6.6	X						X	X				X		

	Category	Protocol Section	Screening/Baseline	Treatment (Cycle = 28 days)						Follow-Up Period						
				Cycle 1		Cycle 2		Subsequent cycles Day 1	End of Study Treatment (EoT)	30 Day Safety Follow Up	60- Day Safety Follow Up	90- Day Safety Follow Up	120- Day Safety Follow Up	150- Day Safety Follow Up	End of Post Treatment Follow Up	Survival Follow Up
**Day of cycle**			**-28 to -1**	**1**	**15**	**1**	**15**	**1**	**1**							
Urine Pregnancy Test	S	7.2.2.6.6				X		X			X	X	X			
Hepatitis testing	D	7.2.2.6.7	X	As clinically indicated												
Cytokine analysis for safety	D	7.2.2.6.8	X	Anytime when a suspected cytokine release syndrome occurs, immediately after the AE, and one week after occurrence of the AE												
For Germany only: local HIV Testing	S	7.2.2.6.9	X													
**Imaging**																
Tumor evaluation per RECIST 1.1	D	7.2.1	X					**During treatment:** C4D1 (± 7 days), every 8 weeks, then from C22D1 (± 7 days), every 12 weeks thereafter<br>**End of Treatment (EOT):** if a scan was not conducted within 30 days prior to end of study treatment.<br>**Efficacy follow-up:** Every 12 weeks starting from the last assessment until documented disease progression per RECIST 1.1 (unless meeting criteria for treatment beyond progression), withdrawal of consent, lost to follow-up, or death irrespective of start of new anti-neoplastic therapy<br>Refer to Section 7.2.1.2 for details on additional tumor assessments to perform in order to confirm response or allow for further central analysis.								

	Category	Protocol Section	Screening/Baseline	**Treatment (Cycle = 28 days)** Cycle 1		Cycle 2		Subsequent cycles Day 1	End of Study Treatment (EoT)	**Follow-Up Period** 30 Day Safety Follow Up	60- Day Safety Follow Up	90- Day Safety Follow Up	120- Day Safety Follow Up	150- Day Safety Follow Up	End of Post Treatment Follow Up	Survival Follow Up
**Day of cycle**			**-28 to -1**	**1**	**15**	**1**	**15**	**1**	**1**							
Chest, abdomen and pelvis CT or MRI (with contrast enhancement)	D	7.2.1	X					**During treatment:** C4D1 (± 7 days), every 8 weeks, then from C22D1 (± 7 days), every 12 weeks thereafter<br>**End of Treatment (EOT):** if a scan was not conducted within 30 days prior to end of study treatment.<br>**Efficacy follow-up:** Every 12 weeks starting from the last assessment until documented disease progression per RECIST 1.1 (unless meeting criteria for treatment beyond progression), withdrawal of consent, lost to follow-up, or death irrespective of start of new anti-neoplastic therapy<br>Refer to Section 7.2.1.2 for details on additional tumor assessments to perform in order to confirm response or allow for further central analysis.								
Brain imaging (CT or MRI)	D	7.2.1	X	If clinically indicated or positive at screening, follow same schedule as CT/MRI of chest, abdomen and pelvis												
Whole body bone scan	D	7.2.1	If clinically indicated													
Localized bone CT, MRI or X-ray	D	7.2.1	Only for lesions on whole body scan that are not visible on the chest, abdomen and pelvis scans. If lesions were documented at screening, follow same schedule as CT/MRI of chest, abdomen and pelvis													
CT or MRI of other metastatic sites (e.g., neck)	D	7.2.1	If clinically indicated or positive at screening, follow same schedule as CT/MRI of chest, abdomen and pelvis													

	Category	Protocol Section	Screening/Baseline	**Treatment (Cycle = 28 days)** Cycle 1		Cycle 2		Subsequent cycles Day 1	End of Study Treatment (EoT)	**Follow-Up Period** 30 Day Safety Follow Up	60- Day Safety Follow Up	90- Day Safety Follow Up	120- Day Safety Follow Up	150- Day Safety Follow Up	End of Post Treatment Follow Up	Survival Follow Up
**Day of cycle**			**-28 to -1**	**1**	**15**	**1**	**15**	**1**	**1**							
Color Digital Photography (including a metric ruler) of skin lesions	D	7.2.1	X					**During treatment:** C4D1 (± 7 days), every 8 weeks, then from C22D1 (± 7 days), every 12 weeks thereafter<br>**End of Treatment (EOT):** if a scan was not conducted within 30 days prior to end of study treatment.<br>**Efficacy follow-up**: Every 12 weeks starting from the last assessment until documented disease progression per RECIST 1.1 (unless meeting criteria for treatment beyond progression), withdrawal of consent, lost to follow-up, or death irrespective of start of new anti-neoplastic therapy<br>Refer to Section 7.2.1.2 for details on additional tumor assessments to perform in order to confirm response or allow for further central analysis.								
Body Fluid/Tissue	D	7.2.1	Needle aspiration, biopsy or any other form of collection (e.g., paracentesis, punctuation) performed for any reason													
**Safety**																
Adverse events	D	7.2.2, 8.1	Suspected AEs up to Day 150; non-suspected AEs up to Day 150 or start of new post treatment anti-neoplastic medication, whichever is sooner.<br>Monitoring of new cutaneous malignancies should continue every month for 6 months following discontinuation of dabrafenib or until initiation of another antineoplastic therapy (refer to Section 6.3.5.3).														
Serious adverse events	D	8.2	Suspected SAEs up to Day 150 and beyond; non-suspected SAEs up to Day 150 or start of new post treatment anti-neoplastic medication, whichever is sooner.														

	Category	Protocol Section	Screening/Baseline	Treatment (Cycle = 28 days)						Follow-Up Period						
				Cycle 1		Cycle 2		Subsequent cycles Day 1	End of Study Treatment (EoT)	30 Day Safety Follow Up	60- Day Safety Follow Up	90- Day Safety Follow Up	120- Day Safety Follow Up	150- Day Safety Follow Up	End of Post Treatment Follow Up	Survival Follow Up
**Day of cycle**			**-28 to -1**	**1**	**15**	**1**	**15**	**1**	**1**							
**Biomarkers**																
Tumor tissue (archival or new) for central BRAF V600 mutation testing, assessment of IO markers and potential companion diagnostic bridging study.<br>Note: if local BRAF V600 mutation result is not available, sample will be used for screening and should be sent for central testing by Day -20	D	7.2.4.1.1	X													
Blood for germline DNA	D	7.2.4.2.1	X													
Optional newly acquired biopsy sampling (if DL1 or DL-1a is determined to be the regimen)	D	7.2.4.1.2	X		X			X (anytime during Cycle 3)	**Unscheduled:** At disease progression per RECIST 1.1 and/or at a subsequent PD per response criteria for immunotherapy if subject continues treatment post progression, prior to the start of new antineoplastic therapy							
Optional newly acquired biopsy sampling (if DL-1b is determined to be the regimen)	D	7.2.4.1.2	X		X			X (anytime during Cycle 4)	**Unscheduled:** At disease progression per RECIST 1.1 and/or at a subsequent PD per response criteria for immunotherapy if subject continues treatment post progression, prior to the start of new antineoplastic therapy							

	Category	Protocol Section	Screening/Baseline	Treatment (Cycle = 28 days)						Follow-Up Period						
				Cycle 1		Cycle 2		Subsequent cycles Day 1	End of Study Treatment (EoT)	30 Day Safety Follow Up	60- Day Safety Follow Up	90- Day Safety Follow Up	120- Day Safety Follow Up	150- Day Safety Follow Up	End of Post Treatment Follow Up	Survival Follow Up
**Day of cycle**			**-28 to -1**	**1**	**15**	**1**	**15**	**1**	**1**							
Blood for ctDNA isolation sampling (For if DL1 or DL-1a is determined to be the regimen)	D	7.2.4.2.2		X		X		C3D1 **only**	**Unscheduled:** At disease progression per RECIST 1.1 and/or at a subsequent PD per response criteria for immunotherapy if subject continues treatment post progression, prior to the start of new antineoplastic therapy							
Blood for ctDNA isolation sampling (if DL-1b is determined to be the regimen)	D	7.2.4.2.2		X		X		C4D1 only	**Unscheduled:** At disease progression per RECIST 1.1 and/or at a subsequent PD per response criteria for immunotherapy if subject continues treatment post progression, prior to the start of new antineoplastic therapy							
Blood for ctRNA isolation sampling (if DL1 or DL-1a is determined to be the regimen)	D	7.2.4.2.3		X		X		C3D1 **only**	**Unscheduled:** At disease progression per RECIST 1.1 and/or at a subsequent PD per response criteria for immunotherapy if subject continues treatment post progression, prior to the start of new antineoplastic therapy							
Blood for ctRNA isolation sampling (if DL-1b is determined to be the regimen)	D	7.2.4.2.3		X		X		C4D1 only	**Unscheduled:** At disease progression per RECIST 1.1 and/or at a subsequent PD per response criteria for immunotherapy if subject continues treatment post progression, prior to the start of new antineoplastic therapy							

	Category	Protocol Section	Screening/Baseline	**Treatment (Cycle = 28 days)**						**Follow-Up Period**						
				**Cycle 1**		**Cycle 2**		**Subsequent cycles Day 1**	**End of Study Treatment (EoT)**	**30 Day Safety Follow Up**	**60- Day Safety Follow Up**	**90- Day Safety Follow Up**	**120- Day Safety Follow Up**	**150- Day Safety Follow Up**	**End of Post Treatment Follow Up**	**Survival Follow Up**
**Day of cycle**			**-28 to -1**	**1**	**15**	**1**	**15**	**1**	**1**							
Blood for cytokines sampling (if DL1 or DL-1a is determined to be the regimen)	D	7.2.4.2.5		X		X		C3D1 **only**	**Unscheduled:** At disease progression per RECIST 1.1 and/or at a subsequent PD per response criteria for immunotherapy if subject continues treatment post progression, prior to the start of new antineoplastic therapy							
Blood for cytokines sampling (if DL-1b is determined to be the regimen)	D	7.2.4.2.5		X		X		C4D1 only	**Unscheduled:** At disease progression per RECIST 1.1 and/or at a subsequent PD per response criteria for immunotherapy if subject continues treatment post progression, prior to the start of new antineoplastic therapy							
Blood for PBMCs sampling (if DL1, DL-1a is determined to be the regimen)	D	7.2.4.2.4		X		X		C3D1 **only**	**Unscheduled:** At disease progression per RECIST 1.1 and/or at a subsequent PD per response criteria for immunotherapy if subject continues treatment post progression, prior to the start of new antineoplastic therapy							
Blood for PBMCs sampling (if DL-1b is determined to be the regimen)	D	7.2.4.2.4		X		X		C4D1 only	**Unscheduled:** At disease progression per RECIST 1.1 and/or at a subsequent PD per response criteria for immunotherapy if subject continues treatment post progression, prior to the start of new antineoplastic therapy							

	Category	Protocol Section	Screening/Baseline	**Treatment (Cycle = 28 days)**						**Follow-Up Period**						
				**Cycle 1**		**Cycle 2**		**Subsequent cycles Day 1**	**End of Study Treatment (EoT)**	**30 Day Safety Follow Up**	**60- Day Safety Follow Up**	**90- Day Safety Follow Up**	**120- Day Safety Follow Up**	**150- Day Safety Follow Up**	**End of Post Treatment Follow Up**	**Survival Follow Up**
**Day of cycle**			**-28 to -1**	**1**	**15**	**1**	**15**	**1**	**1**							
**Study Administration**																
PDR001 or placebo infusion	D	6.1.1		**Regimen as determined by Safety Run In as one of the below options:**<br>**DL1:** PDR001 400mg IV every 4 weeks<br>**DL-1a:** PDR001 400mg IV every 8 weeks<br>**DL-1b:** at C2D1, PDR001 400mg IV every 4 weeks												
Trametinib/Dabrafenib combination	D	6.1.1		Trametinib orally once daily and Dabrafenib orally twice daily												
PK sampling of PDR001 or placebo infusion (if DL1 or DL-1a is determined to be the regimen)	D	7.2.3.1.1		X		X		Day 1 of cycles 3-12, every 6 cycles after C12D1	X	X				X		
PK sampling of PDR001 or placebo infusion (if DL-1b is determined to be the regimen)	D	7.2.3.1.1				X		Day 1 of cycles 3-12, every 6 cycles after C12D1	X	X				X		
PK sampling of trametinib and dabrafenib	D	7.2.3.1.2		X		X		Day 1 of cycles 3-12, every 6 cycles after C12D1	X							
Immunogenicity (IG) sampling (if DL1 or DL-1a is determined to be the regimen)	D	7.2.3.1.1		X		X		Day 1 of cycles 3-12, every 6 cycles after C12D1	X	X				X		

				Treatment (Cycle = 28 days)						Follow-Up Period						
	Category	Protocol Section	Screening/Baseline	Cycle 1		Cycle 2		Subsequent cycles Day 1	End of Study Treatment (EoT)	30 Day Safety Follow Up	60- Day Safety Follow Up	90- Day Safety Follow Up	120- Day Safety Follow Up	150- Day Safety Follow Up	End of Post Treatment Follow Up	Survival Follow Up
**Day of cycle**			**-28 to -1**	**1**	**15**	**1**	**15**	**1**	**1**							
Immunogenicity (IG) sampling (if DL-1b is determined to be the regimen)	D	7.2.3.1.1				X		Day 1 of cycles 3-12, every 6 cycles after C12D1	X	X				X		
Survival Follow-up	D	7.1.8														X

Supplemental material BMJ Publishing Group Limited (BMJ) disclaims all liability and responsibility arising from any reliance placed on this supplemental material which has been supplied by the author(s) J Immunother Cancer

Table 7-4 Visit evaluation schedule: Crossover (only if statistically significant interim or final OS results are demonstrated)

			Treatment (Cycle = 28 days)						Follow-Up Period						
	Category	Protocol Section	Crossover Cycle 1		Crossover Cycle 2		Subsequent crossover cycles Day 1	Crossover End of Study Treatment (EoT)	30 Day Safety Follow Up	60- Day Safety Follow Up	90- Day Safety Follow Up	120- Day Safety Follow Up	150- Day Safety Follow Up	End of Post Treatment Follow Up	Survival Follow Up
**Day of cycle**			**1**	**15**	**1**	**15**	**1**	**1**							
**Obtain Informed Consent**															
IRT Registration		7.1.2.1	X												
Treatment beyond progression ICF	D	6.1.5.1	X (subjects who meet the criteria outlined in Section 6.1.5.1)												
**Patient History**															
Prior/concomitant medications	D	7.1.2.3	From 30 days prior to starting study treatment until 150 Day safety follow-up or start of new antineoplastic medication, whichever is sooner. If new antineoplastic medication is started then only medications relative to the AEs/SAEs that are reported should be collected												
Antineoplastic therapies since discontinuation of study treatment	D	7.1.2.3							X	X	X	X	X		X
**Enrollment**															
Disposition Assessment (at the end of each study phase)	D	7.1.4						X						X	

	Category	Protocol Section	Treatment (Cycle = 28 days)						Follow-Up Period						
			Crossover Cycle 1		Crossover Cycle 2		Subsequent crossover cycles Day 1	Crossover End of Study Treatment (EoT)	30 Day Safety Follow Up	60- Day Safety Follow Up	90- Day Safety Follow Up	120- Day Safety Follow Up	150- Day Safety Follow Up	End of Post Treatment Follow Up	Survival Follow Up
**Day of cycle**			**1**	**15**	**1**	**15**	**1**	**1**							
**Patient Reported Outcomes**<br>PRO questionnaires should be administered at beginning of the scheduled study visit, prior to any tests performed, treatments administered or receipt of test results.															
EORTC QLQ-C30	D	7.2.5.1						**Crossover Cycle 1 Day 1:** if last visit/EOT scan from randomized part was not conducted within 30 days prior to Crossover Cycle 1 Day 1<br>**During treatment:** C4D1, every 8 weeks, then from C22D1, every 12 weeks thereafter until documented disease progression per RECIST 1.1<br>**End of Treatment (EOT):** if a scan was not conducted within 30 days prior to end of study treatment<br>**Unscheduled:** 30 days and 60 days post disease progression per RECIST 1.1							
EQ-5D-5L	D	7.2.5.2						**Crossover Cycle 1 Day 1:** if last visit/EOT scan from randomized part was not conducted within 30 days prior to Crossover Cycle 1 Day 1<br>**During treatment:** C4D1, every 8 weeks, then from C22D1, every 12 weeks thereafter until documented disease progression per RECIST 1.1<br>**End of Treatment (EOT):** if a scan was not conducted within 30 days prior to end of study treatment<br>**Unscheduled:** 30 days and 60 days post disease progression per RECIST 1.1							

	Category	Protocol Section	**Treatment (Cycle = 28 days)**						**Follow-Up Period**						
			**Crossover Cycle 1**		**Crossover Cycle 2**		**Subsequent crossover cycles Day 1**	**Crossover End of Study Treatment (EoT)**	**30 Day Safety Follow Up**	**60- Day Safety Follow Up**	**90- Day Safety Follow Up**	**120- Day Safety Follow Up**	**150- Day Safety Follow Up**	**End of Post Treatment Follow Up**	**Survival Follow Up**
**Day of cycle**			**1**	**15**	**1**	**15**	**1**	**1**							
FACT-M subscale	D	7.2.5.3						**Crossover Cycle 1 Day 1:** if last visit/EOT scan from randomized part was not conducted within 30 days prior to Crossover Cycle 1 Day 1<br>**During treatment:** C4D1, every 8 weeks, then from C22D1, every 12 weeks thereafter until documented disease progression per RECIST 1.1<br>**End of Treatment (EOT):** if a scan was not conducted within 30 days prior to end of study treatment<br>**Unscheduled:** 30 days and 60 days post disease progression per RECIST 1.1							
**Physical Examination**															
Physical Examination (complete)	S	7.2.2.1	If clinically indicated												
Physical Examination (short)	S	7.2.2.1	X		X		X	X	30 days from the last dose of PDR001 and every month for 6 months from the last dose of dabrafenib						
Performance Status (ECOG)	D	7.2.2.4	X		X		X	X	X						
Height	D	7.2.2.3													
Weight	D	7.2.2.3	X		X		X	X	X						
Vital signs	D	7.2.2.2	X		X		X	X	X						
Ophthalmic examination	D	7.2.2.5	As clinically indicated												

			**Treatment (Cycle = 28 days)**						**Follow-Up Period**						
	**Category**	**Protocol Section**	**Crossover Cycle 1**		**Crossover Cycle 2**		**Subsequent crossover cycles Day 1**	**Crossover End of Study Treatment (EoT)**	**30 Day Safety Follow Up**	**60- Day Safety Follow Up**	**90- Day Safety Follow Up**	**120- Day Safety Follow Up**	**150- Day Safety Follow Up**	**End of Post Treatment Follow Up**	**Survival Follow Up**
**Day of cycle**			**1**	**15**	**1**	**15**	**1**	**1**							
12-lead ECG if DL1 is determined to be the regimen	D	7.2.2.7.1	X		X		As clinically indicated	X							
12-lead ECG if DL-1a or DL-1b is determined to be the regimen	D	7.2.2.7.1	X				C3D1 and as clinically indicated	X							
Cardiac function (MUGA or ECHO)	D	7.2.2.7.2	X				X C4D1, every 12 weeks and as clinically indicated	X							
**Laboratory Assessments**															
Hematology	D	7.2.2.6.1	X	X	X	X	X	X	X						
Chemistry	D	7.2.2.6.2	X	X	X	X	X	X	X						
Thyroid function-TSH	D	7.2.2.6.3	X		X		X	X	X						
Thyroid Panel Free T3 and Free T4	D	7.2.2.6.3					Only if TSH is abnormal								
Coagulation	D	7.2.2.6.4	C1D1, every 2 cycles for the first 12 cycles and every 3 cycles thereafter as clinically indicated					As clinically indicated							
Urinalysis (microscopy and macroscopic)	S	7.2.2.6.5	X		X		X	X	X						
Serum Pregnancy Test	S	7.2.2.6.6	X					X	X				X		
Urine Pregnancy Test	S	7.2.2.6.6			X		X			X	X	X			
Hepatitis testing	D	7.2.2.6.7	As clinically indicated perform testing as needed												

	Category	Protocol Section	**Treatment (Cycle = 28 days)**						**Follow-Up Period**						
			**Crossover Cycle 1**		**Crossover Cycle 2**		**Subsequent crossover cycles Day 1**	**Crossover End of Study Treatment (EoT)**	**30 Day Safety Follow Up**	**60- Day Safety Follow Up**	**90- Day Safety Follow Up**	**120- Day Safety Follow Up**	**150- Day Safety Follow Up**	**End of Post Treatment Follow Up**	**Survival Follow Up**
**Day of cycle**			**1**	**15**	**1**	**15**	**1**	**1**							
Cytokine analysis for safety	D	7.2.2.6.8	Anytime when a suspected cytokine release syndrome occurs, immediately after the AE, and one week after occurrence of the AE												
**Imaging**															
Tumor evaluation per RECIST 1.1	D	7.2.1	**Crossover Cycle 1 Day 1:** if last visit/EOT scan from randomized part was not conducted within 30 days prior to Crossover Cycle 1<br>**During treatment:** C4D1 (± 7 days), every 8 weeks, then from C22D1 (± 7 days), every 12 weeks thereafter<br>**End of Treatment (EOT):** if a scan was not conducted within 30 days prior to end of study treatment.<br>**Efficacy follow-up:** Every 12 weeks starting from the last assessment until documented disease progression per RECIST 1.1 (unless meeting criteria for treatment beyond progression), withdrawal of consent, lost to follow-up, or death irrespective of start of new anti-neoplastic therapy<br>Refer to Section 7.2.1.2 for details on additional tumor assessments to perform in order to confirm response or allow for further central analysis.												
Chest, abdomen and pelvis CT or MRI (with contrast enhancement)	D	7.2.1	**Crossover Cycle 1 Day 1:** if last visit/EOT scan from randomized part was not conducted within 30 days prior to Crossover Cycle 1<br>**During treatment:** C4D1 (± 7 days), every 8 weeks, then from C22D1 (± 7 days), every 12 weeks thereafter<br>**End of Treatment (EOT):** if a scan was not conducted within 30 days prior to end of study treatment.<br>**Efficacy follow-up:** Every 12 weeks starting from the last assessment until documented disease progression per RECIST 1.1 (unless meeting criteria for treatment beyond progression), withdrawal of consent, lost to follow-up, or death irrespective of start of new anti-neoplastic therapy<br>Refer to Section 7.2.1.2 for details on additional tumor assessments to perform in order to confirm response or allow for further central analysis.												

	Category	Protocol Section	Treatment (Cycle = 28 days)						Follow-Up Period						
			Crossover Cycle 1		Crossover Cycle 2		Subsequent crossover cycles Day 1	Crossover End of Study Treatment (EoT)	30 Day Safety Follow Up	60- Day Safety Follow Up	90- Day Safety Follow Up	120- Day Safety Follow Up	150- Day Safety Follow Up	End of Post Treatment Follow Up	Survival Follow Up
**Day of cycle**			**1**	**15**	**1**	**15**	**1**	**1**							
Brain imaging (CT or MRI)	D	7.2.1	**Crossover Cycle 1 Day 1:** if last visit/EOT scan from randomized part was not conducted within 30 days prior to Crossover Cycle 1<br>If clinically indicated or positive at baseline, follow same schedule as CT/MRI of chest, abdomen and pelvis												
Whole body bone scan	D	7.2.1	If clinically indicated												
Localized bone CT, MRI or X-ray	D	7.2.1	Only for lesions on whole body scan that are not visible on the chest, abdomen and pelvis scans. If lesions were documented at baseline, follow same schedule as CT/MRI of chest, abdomen and pelvis												
CT or MRI of other metastatic sites (e.g., neck)	D	7.2.1	If clinically indicated or positive at baseline, follow same schedule as CT/MRI of chest, abdomen and pelvis												
Color Digital Photography (including a metric ruler) of skin lesions	D	7.2.1	**Crossover Cycle 1 Day 1:** if last visit/EOT scan from randomized part was not conducted within 30 days prior to Crossover Cycle 1<br>**During treatment:** C4D1 (± 7 days), every 8 weeks, then from C22D1 (± 7 days), every 12 weeks thereafter<br>**End of Treatment (EOT):** if a scan was not conducted within 30 days prior to end of study treatment.<br>**Efficacy follow-up:** Every 12 weeks starting from the last assessment until documented disease progression per RECIST 1.1 (unless meeting criteria for treatment beyond progression), withdrawal of consent, lost to follow-up, or death irrespective of start of new anti-neoplastic therapy<br>Refer to Section 7.2.1.2 for details on additional tumor assessments to perform in order to confirm response or allow for further central analysis.												
Body Fluid/Tissue	D	7.2.1	Needle aspiration, biopsy or any other form of collection (e.g., paracentesis, punctuation) performed for any reason												

	Category	Protocol Section	Treatment (Cycle = 28 days)						Follow-Up Period						
			Crossover Cycle 1		Crossover Cycle 2		Subsequent crossover cycles Day 1	Crossover End of Study Treatment (EoT)	30 Day Safety Follow Up	60- Day Safety Follow Up	90- Day Safety Follow Up	120- Day Safety Follow Up	150- Day Safety Follow Up	End of Post Treatment Follow Up	Survival Follow Up
**Day of cycle**			**1**	**15**	**1**	**15**	**1**	**1**							
**Safety**															
Adverse events	D	7.2.2, 8.1	Suspected AEs up to Day 150; non-suspected AEs up to Day 150 or start of new post treatment anti-neoplastic medication, whichever is sooner. Monitoring of new cutaneous malignancies should continue every month for 6 months following discontinuation of dabrafenib or until initiation of another antineoplastic therapy (refer to Section 6.3.5.3).												
Serious adverse events	D	8.2	Suspected SAEs up to Day 150 and beyond; non-suspected SAEs up to Day 150 or start of new post treatment anti-neoplastic medication, whichever is sooner.												
**Biomarkers**															
Blood for cytokines	D	7.2.4.2.5	X				C3D1 only	**Unscheduled:** At disease progression per RECIST 1.1 and/or at a subsequent PD per response criteria for immunotherapy if subject continues treatment post progression, prior to the start of new antineoplastic therapy							
Blood for PBMCs	D	7.2.4.2.4	X				C3D1 only	**Unscheduled:** At disease progression per RECIST 1.1 and/or at a subsequent PD per response criteria for immunotherapy if subject continues treatment post progression, prior to the start of new antineoplastic therapy							
Optional newly acquired biopsy	D	7.2.4.1.2					X (anytime during Cycle 3)	**Unscheduled:** At disease progression per RECIST 1.1 and/or at a subsequent PD per response criteria for immunotherapy if subject continues treatment post progression, prior to the start of new antineoplastic therapy							

	Category	Protocol Section	Treatment (Cycle = 28 days)						Follow-Up Period						
			Crossover Cycle 1		Crossover Cycle 2		Subsequent crossover cycles Day 1	Crossover End of Study Treatment (EoT)	30 Day Safety Follow Up	60- Day Safety Follow Up	90- Day Safety Follow Up	120- Day Safety Follow Up	150- Day Safety Follow Up	End of Post Treatment Follow Up	Survival Follow Up
**Day of cycle**			**1**	**15**	**1**	**15**	**1**	**1**							
**Study Administration**															
PDR001	D	6.1.1.4	**Regimen as determined by Safety Run-In as one of the below options:**<br>**DL1:** PDR001 400mg IV every 4 weeks<br>**DL-1a:** PDR001 400mg IV every 8 weeks<br>**DL-1b:** at C2D1, PDR001 400mg IV every 4 weeks												
Trametinib/Dabrafenib combination	D	6.1.1.5	Trametinib orally once daily and Dabrafenib orally twice daily												
PK sampling of PDR001 (if DL1 or DL-1a is determined to be the regimen)	D	7.2.3.1.1	X		X		Day 1 of cycles 3-12, every 6 cycles after C12D1	X	X				X		
PK sampling of PDR001 (if DL-1b is determined to be the regimen)	D	7.2.3.1.1			X		Day 1 of cycles 3-12, every 6 cycles after C12D1	X	X				X		
PK sampling of trametinib and dabrafenib	D	7.2.3.1.2	X		X		Day 1 of cycles 3-12, every 6 cycles after C12D1	X							
Immunogenicity (IG) sampling (if DL1 or DL-1a is determined to be the regimen)	D	7.2.3.1.1	X		X		Day 1 of cycles 3-12, every 6 cycles after C12D1	X	X				X		

	Category	Protocol Section	Treatment (Cycle = 28 days)						Follow-Up Period						
			Crossover Cycle 1		Crossover Cycle 2		Subsequent crossover cycles Day 1	Crossover End of Study Treatment (EoT)	30 Day Safety Follow Up	60- Day Safety Follow Up	90- Day Safety Follow Up	120- Day Safety Follow Up	150- Day Safety Follow Up	End of Post Treatment Follow Up	Survival Follow Up
**Day of cycle**			**1**	**15**	**1**	**15**	**1**	**1**							
Immunogenicity (IG) sampling (if DL-1b is determined to be the regimen)	D	7.2.3.1.1			X		Day 1 of cycles 3-12, every 6 cycles after C12D1	X	X				X		
Survival	D	7.1.8													X

7.1.1 Molecular pre-screening

Not applicable.

7.1.2 Screening

Written informed consent/assent must be obtained prior to any screening procedures. Screening assessments to confirm eligibility into the study should be performed as per visit evaluation schedule between Day-28 and Day-1. Serum pregnancy test must be conducted within 72 hours prior to start of study treatment and must be confirmed negative before the first dose of study treatment. Physical examinations and laboratory assessments performed as part of the screening evaluations will not require to be repeated prior to dosing if performed within 7 days prior to the first dose of study treatment. Laboratory results from the central laboratory will be used to determine subject's eligibility. However, the site does not need to wait for the results of centrally-analyzed laboratory assessments when an immediate clinical decision needs to be made and in those cases locally unscheduled testing may be performed and used for eligibility assessments.

The cardiac eligibility criteria should be assessed with the local ECG report.

A subject who has a laboratory test result(s) that does not satisfy the entrance criteria may have the test(s) repeated. These test(s) may be repeated as soon as the investigator believes the re-test result(s) is/are likely to be within the acceptable range to satisfy the entrance criteria, but should be completed within approximately 3 weeks of the original screening visit date. In this case, the subject will not be required to sign another ICF, and the original subject ID number assigned by the investigator will be used. In the event that the laboratory test(s) cannot be performed within 3 weeks of the original screening visit, or the re-test(s) do not meet the entrance criteria, or other eligibility criteria have changed and are not met anymore, the subject is considered a screen failure.

A new ICF will need to be signed if the investigator chooses to re-screen the subject after a subject has screen failed, however, the subject ID number will remain the same. All required screening activities must be performed when the subject is re-screened for participation in the study. An individual subject may only be re-screened once for the study. Once the number of subjects screened and enrolled is likely to ensure target enrollment, the Sponsor may close the study to further screening. In this case, the subjects who screen failed will not be permitted to re-screen.

Subjects will be enrolled based on local *BRAF* V600 mutation result as part of study inclusion criteria 9 (applicable to all study cohorts). The *BRAF* V600 mutation will be assessed using a validated tissue-based molecular test for *BRAF* V600 mutations. Immunohistochemistry is not an accepted method and liquid biopsy based *BRAF* V600 results cannot be used to enroll patients. The eCRF page for methods of local BRAF mutation testing must be completed for subjects whose tumor sample was tested by a local institutional assay. Verification of locally documented *BRAF* V600 mutation result must be completed prior to randomization. If the local lab uses a non-FDA approved BRAF V600 assay (FDA approved tests for BRAF V600 mutations include: biomerieux THxID® BRAF Detection Kit and Roche Cobas 4800 BRAF

V600 Mutation Test), information about the assay must be provided to the Sponsor or representative for review.

BRAF V600 mutation results will be subjected to retrospective central confirmation by a Novartis designated laboratory and FDA approved assay (e.g., Biomerieux THxID™-BRAF).

For the retrospective central confirmation all subjects will be required to provide a tumor tissue sample at screening prior to study treatment, as either a tumor block or a minimum of 25 FFPE slides (see Section 7.2.4). Central confirmation of *BRAF* V600 mutation status is not required for enrollment if other inclusion and exclusion criteria are fulfilled. Subjects will not be excluded if centralized testing is later found to be discordant or uninformative (e.g., inadequate sample).

For subjects who do not have a *BRAF* V600 mutation result documented locally, **a tumor sample (tumor block or minimum 25 slides)** must be submitted for central testing of *BRAF* mutation status by day -20. The test to confirm *BRAF* V600 activating mutation will be performed either using archival tissue, or preferably, using a new biopsy prior to study entry. Some of the tissue material obtained from all subjects (including screen failures) will be retained by Novartis, for exploratory biomarker testing and to allow the development of additional companion diagnostic test such as Next Generation Sequencing (NGS). Please see Table 7-16 and Table 7-17 for details on the tumor sample requirement for central *BRAF* V600 mutation testing and additional biomarker studies.

7.1.2.1 Eligibility screening

Following registering in the IRT for screening, subject eligibility will be checked once all screening procedures are completed. The eligibility check will be embedded in the IRT system. Please refer and comply with detailed guidelines in the IRT manual.

7.1.2.2 Information to be collected on screening failures

Subject who signed an Informed Consent Form but failed to be started on treatment for any reason will be considered a screen failure. Subjects who are found not eligible after signing the main study consent will be considered as screening failures, and data will be handled in the same manner.

The following eCRFs should be completed for screen failures:

- Study part
- Demography
- Informed Consent
- Screening Phase Disposition
- Inclusion/Exclusion Criteria
- Biomarker Assessment - BRAF V600 Mutation Local Result

No other data will be entered into the clinical database for subjects who are screen failures, unless the subject experienced a Serious Adverse Event during the Screening Phase (see Section 8 for SAE reporting details).

7.1.2.3 Subject demographics and other baseline characteristics

The data to be collected on subject characteristics at screening includes:

- Demography (age, gender, childbearing potential, race and ethnicity, or as allowed by local regulations)
- Past and current medical conditions including cardiovascular medical history and risk factors
- Diagnosis and extent of cancer using AJCC edition 7 and 8
- Alcohol history
- Smoking history
- HIV history (for Germany only: HIV test is required during screening using a local test)
- Prior antineoplastic therapies (medications, radiation, surgeries)
- Prior and current concomitant medications, surgical and medical procedures and significant non-drug therapies

Note: All other medications taken within 28 days or 5 half-lives, whichever is shorter before the first dose of study treatment is administered must be recorded on the Prior and current concomitant medication eCRF page and updated on an ongoing basis if there is new change to the medication.

Assessments to be performed at screening/baseline include;

- Physical examination (e.g., performance status (ECOG), height, weight, vital signs, ophthalmic and skin examination)
- Cardiovascular assessments (e.g., ECG, ECHO or MUGA)
- Laboratory assessments (e.g., hematology, chemistry, thyroid function, coagulation, urinalysis, serum pregnancy test, hepatitis B, C test, and cytokine analysis)
- Tumor assessment (RECIST 1.1, color digital photography (including metric ruler for skin lesions)
- Biomarker assessments in blood and tumor tissue

7.1.3 Treatment period

- Study treatment will begin on Day 1 of Cycle 1.
- Study treatment will continue until the subject experiences any of the following: disease progression according to RECIST 1.1 as determined by investigator, unacceptable toxicity that precludes further treatment, pregnancy, start of a new anti-cancer therapy, discontinuation at the discretion of the Investigator or subject, lost to follow-up, death, or study termination by the sponsor.
- Subjects may continue study treatment beyond disease progression by RECIST 1.1 until disease progression per response criteria for immunotherapy (if criteria stipulated in Section 6.1.5.1 are fulfilled), unacceptable toxicity, start of new anti-neoplastic therapy, withdrawal of consent, physician’s decision, lost to follow-up, death, or study is terminated by the sponsor. In case of continuation of study treatment beyond disease progression by RECIST 1.1, the subject will continue tumor assessments as outlined in Section 7.

- Subjects randomized to receive dabrafenib, trametinib, and placebo will only be permitted to crossover to dabrafenib, trametinib, and PDR001 in the case that statistically significant interim or final OS results are demonstrated Section 4.1.2). For these patients, physical examinations, laboratory, ECG, and ECHO assessments performed as part of the EOT visit in part 3 will not be required to be repeated at Crossover C1D1 if performed within 7 days prior to the first dose of study treatment in crossover part. Tumor and PRO assessments will not be required to be repeated at Crossover C1D1 if performed within 30 days prior to the first dose of study treatment in crossover part.

Visit frequency

- Safety run-in part: visits will be scheduled for C1D1, (C1D8 for DL1 and DL-1a only), C1D15, C2D1, (C2D8 for DL-1b only), C2D15, C3D1, C3D15, C4D1, (C4D15 for DL-1a only), C5D1, and every 4 weeks thereafter. For details of assessment, refer to Table 7-1.
- Biomarker cohort: visits will be scheduled for C1D1, C1D15, C2D1, C2D15, C3D1, C3D15, C4D1, and every 4 weeks thereafter. For details of assessment, refer to Table 7-2.
- Randomized part: visits will be scheduled for C1D1, C1D15, C2D1, C2D15, C3D1, and every 4 weeks thereafter. For details of assessment, refer to Table 7-3.
- Crossover part: visits will be scheduled for crossover C1D1, C1D15, C2D1, C2D15, C3D1, and every 4 weeks thereafter. For details of assessment, refer to Table 7-4.

Windows for scheduling assessments

- Every effort must be made to follow the schedule of assessments within the windows outline in the protocol.
- For Cycle 1, all assessments have a ± 3 day window, unless otherwise indicated.
- After Cycle 1, all assessments have a ± 7 day window, unless otherwise indicated.
- Subjects who discontinue study treatment must have an End of Treatment (EOT) visit performed ≤ 7 days after stopping study treatment.

7.1.4 Discontinuation of study treatment

Subjects may voluntarily discontinue from the study treatment for any reason at any time. If a subject decides to discontinue from the study treatment, the investigator should make a reasonable effort (e.g., telephone, e-mail, letter) to understand the primary reason for this decision and record this information in the subject's chart and on the appropriate eCRF pages. They may be considered withdrawn if they state an intention to withdraw, fail to return for visits, or become lost to follow-up for any other reason.

The investigator may discontinue study treatment for a given subject if, he/she believes that continuation would be detrimental to the subject's well-being.

Study treatment must be discontinued under the following circumstances:

- Emergence of specific adverse events or laboratory abnormalities under some circumstances as outlined in Section 6.3.
- Pregnancy (pregnancy will be followed for outcome)
- Use of prohibited treatment
- Any protocol deviation that results in a significant risk to the subject's safety

The appropriate personnel from the site and Novartis will assess whether study treatment should be discontinued for any subject whose treatment code has been broken inadvertently for any reason.

Subjects who become pregnant during the trial must be withdrawn (Section 8.4). Subjects who become pregnant must cease all tumor assessments regardless of whether or not they developed Progressive Disease according to investigator.

Subjects who discontinue study treatment should NOT be considered withdrawn from the study. They should return for the assessments indicated in Section 7.2. If they fail to return for these assessments for unknown reasons, every effort (e.g., telephone, email, and letter) should be made to contact them as specified in Section 7.1.9.

Subjects who discontinue study treatment should be scheduled for an EOT visit as soon as possible and within 7 days of the last dose of study treatment, at which time all of the assessments listed for the EOT visit will be performed. If a subject is discontinued from treatment at a study visit, the assessments performed at that visit will be considered as EOT assessments and do not need to be repeated. An End of Treatment Disposition eCRF page should be completed, giving the date and reason for stopping treatment.

For subjects who discontinue treatment for reasons other than documented disease progression per RECIST 1.1 or response criteria for immunotherapy, death, lost to follow-up, or withdrawal of consent, tumor assessments must continue to be performed every 12 weeks starting from the last assessment until documented disease progression per RECIST 1.1 or response criteria for immunotherapy, death, lost to follow-up, or withdrawal of consent.

An End of Treatment Disposition eCRF page should be completed, specifying the date of the subject’s last treatment and reason for stopping study treatment.

The investigator must also contact the IRT to register the subject’s discontinuation from study treatment.

In some circumstances subjects may be allowed to continue to receive study treatment beyond disease progression as per RECIST 1.1 criteria (Section 6.1.4). These subjects will continue assessments as outlined in Section 7, and will complete the EOT visit only after permanent discontinuation of study treatment.

7.1.4.1 Replacement policy

Part 1: Safety run-in

If a subject is considered as non-evaluable for the Dose-Determining Set (DDS), a new subject will be enrolled until at least the minimum number of 6 evaluable subjects is achieved within the cohort.

Part 2: Biomarker cohort

If not possible to collect at least three tumor biopsies (at screening, C1D15 and one additional biopsy on treatment), a new subject will be enrolled to allow for a sufficient number of evaluable subjects for the biomarker studies.

Part 3: Double-blind, randomized, placebo-controlled part

Subjects will not be replaced.

7.1.5 Withdrawal of study consent

Subjects may voluntarily withdraw consent to participate in the study for any reason at any time. Withdrawal of consent occurs only when a subject does not want to participate in the study any longer, and does not allow any further collection of personal data.

If a subject withdraws consent, the investigator should make a reasonable effort (e.g., telephone, e-mail, letter) to understand the primary reason for the subject's decision to withdraw his/her consent and record this information.

Study treatment must be discontinued and no further assessments conducted, and the data that would have been collected at subsequent visits will be considered missing.

Further attempts to contact the subject are not allowed unless safety findings require communication or follow up.

All efforts should be made to complete the assessments prior to study withdrawal. A final evaluation at the time of the subject's study withdrawal should be made as detailed in the assessment table.

Novartis will continue to keep and use collected study information (including any data resulting from the analysis of a subject's samples until their time of withdrawal) according to applicable law.

For US and Japan: All biological samples not yet analyzed at the time of withdrawal may still be used for further testing/analysis in accordance with the terms of this protocol and of the informed consent form.

For EU and Rest of World (RoW): All biological samples not yet analyzed at the time of withdrawal will no longer be used, unless permitted by applicable law. They will be stored according to applicable legal requirements.

7.1.6 Follow up for safety evaluations

All subjects must be followed for safety up to 150 days after the last dose of PDR001/placebo. After the 30-day onsite safety follow-up visit, subjects will be followed (via telephone call or onsite visit if subject happens to be visiting the site) at 60, 90, 120 and 150 days after the last dose of PDR001/placebo (Appendix 3).

In case where individual drug components of the study treatment are discontinued at different times, subjects must be followed for safety evaluation up to 150 days following the last dose of PDR001/placebo or up to 30 days after the last dose of dabrafenib or 120 days after the last dose of trametinib, whichever is longer. In addition, subjects will be followed for skin related toxicity every month for 6 months from the last dose of dabrafenib.

All safety assessments should be completed outlined in Section 7.1. However, if the subject begins post treatment antineoplastic medication before the 150-Day safety follow-up visit the collection of new SAEs and AEs unrelated to study medication will stop and thereafter only suspected AEs and suspected SAEs will continue to be collected up to Day 150. Suspected

SAEs will continue to be collected beyond the 150-Day safety visit. Data collected should be added to the appropriate eCRF pages. For female subjects of child bearing potential, a pregnancy test will be performed at the time points listed in Section 7.1.

Data collected should be added to the Adverse Events eCRF and the Concomitant Medications eCRF.

7.1.7 Follow up for efficacy evaluations

All subjects who discontinue study treatment without disease progression by RECIST 1.1 will continue tumor assessments as outlined in Section 7.1 until documented disease progression by RECIST 1.1, withdrawal of consent, lost to follow-up, or death irrespective of start of new anti-neoplastic therapy.

All subjects who continue treatment beyond disease progression per RECIST 1.1 (see Section 6.1.5.1) will continue tumor assessments as outlined in Section 7 until confirmed progression per response criteria for immunotherapy, unacceptable toxicity, start of new anti-neoplastic therapy, withdrawal of consent, physician's decision, lost to follow-up, death or study is terminated by sponsor.

Refer to Section 7.2.1.2 for details on additional tumor assessments that should be performed to confirm response or allow for further central analysis.

7.1.8 Survival follow-up

Subjects will enter the survival follow-up period once they complete the safety follow-up period or have disease progression as per RECIST 1.1 or response criteria for immunotherapy whichever is longer. Subjects will then be contacted by telephone every 12 weeks to follow-up on their survival status. Any new antineoplastic medications that have been started since the last contact date will also be collected during these phone calls.

7.1.9 Lost to follow-up

For subjects whose status is unclear because they fail to appear for study visits without stating an intention to withdraw consent, the investigator should show "due diligence" by contacting the subject, family or family physician as agreed in the informed consent and by documenting in the source documents steps taken to contact the subject, e.g., dates of telephone calls, registered letters, etc. A subject should not be considered lost to follow-up until due diligence has been completed. Subjects lost to follow up should be recorded as such on the appropriate Disposition eCRF.

7.2 Assessment types

7.2.1 Efficacy assessments

Tumor response will be assessed locally according to the Novartis guideline version 3.2 based on RECIST 1.1 (Appendix 1). The imaging assessment collection plan is presented in Table 7-5. Details of the central review process will be described in the independent review charter.

Imaging data will be centrally collected and centrally checked for quality by an imaging CRO designated by Novartis. The local investigator's assessment will be used for the primary

endpoint analysis and for treatment decision making. Central review of the imaging data using RECIST 1.1 and tumor response criteria based on guidelines for immunotherapy (e.g., Seymour 2017) will be performed for sensitivity analysis and completed prior to planned database locks for the interim and final PFS/interim OS analyses.

Information regarding prior interventions (e.g., radiotherapy), pre-existing radiographic findings that mimic metastatic disease at baseline/screening and prior interventions should be transmitted to the imaging CRO via the Baseline Clinical Form along with the baseline images. Sites must ensure the data entered on the form is consistent with the data entered in the clinical database.

Clinical data (e.g., physical exam, biopsy results, surgical procedures and pathology data (e.g., histology, cytology, fluid collections)) captured at baseline and on-study will be transmitted to the imaging CRO for review by a medical oncologist. Sites must ensure the data entered on the baseline form is consistent with the data entered in the clinical database.

Table 7-5 Imaging assessment collection plan for Parts 1, 2, 3 and Crossover

Procedure	Screening/Baseline (Day -28 to Day -1)	During Treatment/Follow-up and Crossover (± 7 day window)
Chest, abdomen and pelvis CT or MRI (with contrast enhancement)	Mandated	• During treatment: C4D1 (± 7 days), every 8 weeks, then from C22D1 (± 7 days) every 12 weeks<br>• End of Treatment (EOT): if a scan was not conducted within 30 days prior to end of study treatment.<br>• Efficacy follow-up: Every 12 weeks starting from the last assessment until documented disease progression per RECIST 1.1 (unless meeting criteria for treatment beyond progression), withdrawal of consent, lost to follow-up, or death irrespective of start of new anti-neoplastic therapy<br>• Refer to Section 7.2.1.2 for details on additional tumor assessments to perform in order to confirm response or allow for further central analysis.<br>• Crossover Cycle 1 Day 1 (unless last visit/EOT scan from randomized part was conducted within 30 days prior to Crossover Cycle 1)
Brain imaging (CT or MRI)	Mandated	• If lesions were documented at baseline or if clinically indicated, follow same schedule as CT/MRI of chest, abdomen, and pelvis.
Whole body bone scan	If clinically indicated	• If clinically indicated
Localized bone CT, MRI or X-ray	For any lesions identified on the whole body bone scan that are not visible on the chest, abdomen and pelvis CT or MRI	• If lesions were documented at baseline or if clinically indicated, follow same schedule as CT/MRI of chest, abdomen, and pelvis
Color digital photography (with scale/ruler) of skin lesions	For any skin lesions present	• If skin lesions were documented at baseline or present on study, follow same schedule as CT/MRI of chest, abdomen, and pelvis.
CT or MRI of other metastatic sites (e.g., neck)	If clinically indicated	• If lesions were documented at baseline or if clinically indicated, follow same schedule as CT/MRI of chest, abdomen, and pelvis.

7.2.1.1 Baseline imaging assessments

Imaging assessments will be performed at screening/baseline within 28 days of start of treatment (Day -28 to Day -1 prior to Cycle 1 Day 1).

Any imaging assessments already completed during the regular work-up of the subject within 28 days prior to start of treatment, including before signing the main study ICF, can be considered as the baseline images for this study. Any imaging assessments obtained after randomization cannot be considered baseline images. The following assessments are required at screening/baseline:

- Chest, abdomen and pelvis CT or MRI
- Brain CT or MRI
- Whole body bone scan, if clinically indicated
- Localized bone CT, MRI or x-ray, for any lesions identified on the whole body bone scan that are not visible on the chest, abdomen and pelvis CT or MRI
- Color photography, including a metric ruler to measure the size of the lesion, for any skin lesions present
- CT or MRI of other metastatic sites (e.g., neck), if clinically indicated

If a subject is known to have a contraindication to CT intravenous (IV) contrast media or develops a contraindication during the trial, a non-contrast CT of the chest (MRI is not recommended due to respiratory artifacts, however if CT is not feasible per local regulations, MRI can be performed instead) plus a contrast-enhanced MRI (if possible) of the abdomen and pelvis should be performed.

Contrast enhanced brain MRI is preferred, however, if MRI contrast is contraindicated, then MRI without contrast or CT with/without contrast is acceptable.

If clinically indicated, a whole body bone scan must be performed per institutional standard of care [e.g., Tc-99 bone scan, whole body bone MRI, Fluorodeoxyglucose positron emission tomography (FDG-PET) or sodium fluoride (NaF) PET]. Localized CT, MRI or X-rays must be acquired for all skeletal lesions identified on the screening whole body bone scan, which are not visible on the chest, abdomen and pelvis CT/MRI.

If clinically indicated, CT or MRI of other areas (e.g., neck) of disease as appropriate should be performed.

If skin lesions are present at screening, color photography should be acquired using a digital camera in clear focus, including a scale/ruler, in such a way that the size of the lesion(s) can be determined from the photograph.

Any potentially measurable lesion that has been previously treated with radiotherapy should be considered as a non-measurable lesion. However, if a lesion previously treated with radiotherapy has clearly progressed since the radiotherapy, it can be considered as a measurable lesion.

Chest x-rays and ultrasound should not be used to measure tumor lesions.

7.2.1.2 Post-baseline imaging assessments

Imaging assessments as described in Table 7-5 should be performed using the same imaging modality used at baseline, irrespective of study treatment interruption or actual dosing. Imaging assessments for response evaluation will be performed at Cycle 4 Day 1 (± 7 days) and every 8 weeks during the first 18 months from the first dose of study treatment, and then from Cycle 22

Day 1 (± 7 days), every 12 weeks thereafter until disease progression per RECIST 1.1, and/or response criteria for immunotherapy, death, lost to follow-up or withdrawal of consent. Imaging assessments should be scheduled using the randomization date as the reference date (not the date of the previous tumor assessment), and should be respected regardless of whether treatment with study treatment is temporarily withheld or unscheduled assessments performed.

An additional tumor assessment must be performed to confirm response (CR or PR) no less than 4 weeks after the criteria for response are first met.

Subsequent disease progression per RECIST 1.1:

- For subjects who discontinue study treatment without disease progression per RECIST 1.1, imaging assessments will be performed until disease progression per RECIST 1.1, death, lost to follow-up or withdrawal of consent. Subsequent to disease progression per RECIST 1.1, at least two additional tumor assessments that are at least 4 weeks apart are recommended (for further central analysis) no less than 4 weeks after criteria for PD are first met. While tumor assessments after start of new anti-neoplastic therapy should be performed up to and including disease progression per RECIST 1.1, tumor assessments after start of new anti-neoplastic therapy post-progression are recommended however not required.
- For subjects who continue treatment beyond progression, at least two additional tumor assessments that are at least 4 weeks apart must be performed (for further central analysis) no less than 4 weeks after criteria for PD per RECIST 1.1 are first met. Tumor assessments after start of new anti-neoplastic therapy are recommended however not required.
- For subjects who discontinue treatment due to disease progression per RECIST 1.1, at least two additional tumor assessments that are at least 4 weeks apart are recommended (for further central analysis) no less than 4 weeks after the criteria for PD are first met. Tumor assessments after start of new anti-neoplastic therapy are recommended however not required.

Additional imaging assessments may be performed at any time during the study at the investigator's discretion to support the efficacy evaluations for a subject, as necessary. Clinical suspicion of disease progression at any time requires a physical examination and imaging assessments to be performed promptly rather than waiting for the next scheduled imaging assessment.

Each lesion that is measured at baseline must be measured by the same method (either same imaging method or by photography, including a metric ruler) and when possible, by the same local radiologist/physician throughout the study so that the comparison is consistent. The same is true for any new lesions that occur after start of treatment. They should be imaged using the same modality at each subsequent time point. New cutaneous lesions need to be color photographed including a metric ruler to measure the size of the lesion. If an off-schedule imaging assessment is performed because progression is suspected, subsequent imaging assessments should be performed in accordance with the original imaging schedule.

Combined PET/CT may be used only if the CT is of similar diagnostic quality as a CT performed without PET, including the utilization of IV contrast media. At the discretion of the

Investigators, FDG-PET scans may be performed to document progressive disease per RECIST 1.1 (Appendix 1).

All study imaging (including any off-schedule imaging studies) should be submitted to the designated imaging CRO for quality control.

7.2.2 Safety and tolerability assessments

Safety will be monitored by assessing physical examination, performance status, laboratory examinations, ECGs, cardiac imaging as well as collecting of the adverse events at every visit. For details on AE collection and reporting, refer to Section 8.

More frequent examinations may be performed at the investigator's discretion, if clinically indicated.

7.2.2.1 Physical examination

A complete physical examination will be performed at screening and later as clinically indicated and will include the examination of general appearance, skin, neck (including thyroid), eyes, ears, nose, throat, lungs, heart, abdomen, back, lymph nodes, extremities, vascular, and neurological assessments. If indicated based on medical history and/or symptoms, rectal, external genitalia, breast, and pelvic exams will be performed.

A short physical exam will be performed as per schedule in Table 7-1, Table 7-2, Table 7-3, and Table 7-4 and will include the examination of general appearance, skin (including a full skin exam every 8 weeks), and vital signs (blood pressure and pulse).

Significant findings that were present prior to the signing of informed consent must be included in the Medical History page on the subject's CRF. Significant new findings that begin or worsen after informed consent must be recorded on the Adverse Event page of the subject's CRF.

7.2.2.2 Vital signs

Vital signs include blood pressure (supine position preferred when ECG is collected), pulse measurement, and body temperature and will be measured at screening and at subsequent time points as specified in Table 7-1, Table 7-2, Table 7-3 and Table 7-4.

7.2.2.3 Height and weight

Height will be measured at screening.

Body weight (in indoor clothing, but without shoes) will be measured at screening and at subsequent time points as specified in Table 7-1, Table 7-2, Table 7-3, and Table 7-4.

7.2.2.4 Performance status

Performance status will be assessed as described in the Table 7-1, Table 7-2, Table 7-3, and Table 7-4. More frequent examinations may be performed at the investigator's discretion, if medically indicated. ECOG Performance status scale will be used as described in the Table 7-6.

Table 7-6 ECOG performance status scale

Score	Performance Status
0	Fully active, able to carry on all pre-disease performance without restriction
1	Restricted in physically strenuous activity but ambulatory and able to carry out work of a light or sedentary nature, e.g., light housework, office work
2	Ambulatory and capable of all self-care but unable to carry out any work activities. Up and about more than 50% of waking hours
3	Capable of only limited self-care, confined to bed or chair more than 50% of waking hours
4	Completely disabled. Cannot carry on any self-care. Totally confined to bed or chair
5	Dead

7.2.2.5 Ophthalmic examination

A standard ophthalmic examination by an ophthalmologist will be performed as per schedule in Table 7-1, Table 7-2, Table 7-3, and Table 7-4. The exam will include best corrected visual acuity, tonometry, slit lamp biomicroscopic examination, visual field examination, and dilated indirect fundoscopy with special attention to retinal abnormalities (dilation only required if clinically indicated). Optical coherence tomography is recommended at scheduled visits, and mandated if retinal abnormalities are suspected. Other types of ancillary testing including color fundus photography and fluorescein angiography are also recommended if clinically indicated.

7.2.2.6 Laboratory evaluations

A central laboratory will be used for analysis of scheduled hematology, biochemistry, and other blood specimens collected as part of safety monitoring (as detailed in Table 7-7), except urinalysis and pregnancy tests. The frequency of the assessments is indicated in the relevant Visit Evaluation Schedule (Table 7-1, Table 7-2, Table 7-3, and Table 7-4).

For assessment of subjects' eligibility to the study, laboratory results from the central laboratory will be used. However, the site does not need to wait for the results of centrally-analyzed laboratory assessments when an immediate clinical decision needs to be made and in those cases locally unscheduled testing may be performed and used for eligibility assessments.

Local laboratory assessments may be performed if medically indicated or when the treating physician cannot wait for central laboratory results for decision making. In this particular situation, the blood sample obtained at the same time point should be submitted to the central laboratory for analysis in parallel with local analysis.

The results of the local laboratory will be recorded in the eCRF if the following criteria are met:

- a treatment decision was made based on the local results, or
- there are no concomitant central results available

Details on the collections, shipment of samples and reporting of results by the central laboratory are provided to investigators in a separate Laboratory manual.

The CRA will obtain the local laboratory reference ranges and a copy of the laboratory certification for all laboratory results that are entered into the eCRF.

Table 7-7 Clinical laboratory parameters collection plan for all 3 parts

Test Category	Test Name
Hematology	Hemoglobin, Platelets, White blood cells, Differential (Basophils, Eosinophils, Lymphocytes, Monocytes, Neutrophils, Bands, Other)
Chemistry	Albumin, Alkaline phosphatase, ALT, AST, Lactate dehydrogenase (LDH), Calcium, Magnesium, Phosphorus, Sodium, Potassium, Chloride, Creatinine, Creatine kinase, Direct Bilirubin, Total Bilirubin, Total Cholesterol, Blood Urea Nitrogen (BUN) or Urea, Uric Acid, Amylase, Lipase, Glucose (fasting or non-fasting)
Thyroid	At baseline: TSH (Thyroid Stimulation Hormone), Free T3 and Free T4<br>At the subsequent visits as indicated in Table 7-1, Table 7-2, Table 7-3, and Table 7-4: TSH only.<br>If TSH is abnormal, central lab will test Free T3 and Free T4
Coagulation	International normalized ratio (INR), Activated partial thromboplastin time (APTT)
Urinalysis	Local Laboratory: Macroscopic Panel (Dipstick) (Color, Bilirubin, Blood, Glucose, Ketones, Leukocytes esterase, Nitrite, pH, Protein, Specific Gravity, Urobilinogen)<br>If dipstick is abnormal then perform local laboratory Microscopic Panel (Red Blood Cells, White Blood Cells, Casts, Crystals, Bacteria, Epithelial cells)
Pregnancy Test	A serum pregnancy test must be performed at screening (at the local laboratory) ≤ 72 hours before first dose of study treatment, at EOT, at 30-day and 150 day safety follow-up.<br>A local laboratory urine pregnancy test must be performed at day 1 of every cycle beginning with cycle 2, and at home every 30-days after the 30-day safety follow-up visit until 120-day follow-up.
Hepatitis markers	HBV-DNA, HbsAg, HbsAb, HbcAb, HCV RNA-PCR
Virology	For Germany only: Local HIV testing at screening
Cytokines	IFN-γ, IL-6, IL-1,TNF-α

7.2.2.6.1 Hematology

Hematology assessments listed in Table 7-7 will be measured according to the relevant Visit Evaluation Schedule (Table 7-1, Table 7-2, Table 7-3, or Table 7-4). More frequent hematology testing may also be performed as medically necessary. Additional results from unscheduled hematology lab evaluations should be recorded on the appropriate Unscheduled Visit eCRF.

7.2.2.6.2 Clinical chemistry

Blood chemistry assessments listed in Table 7-7 will be measured according to the relevant Visit Evaluation Schedule (Table 7-1, Table 7-2, Table 7-3, or Table 7-4).

More frequent clinical chemistry testing may also be performed as medically necessary. Additional results from unscheduled clinical chemistry lab evaluations should be recorded on the appropriate Unscheduled Visit eCRF.

It should be noted in the subject's eCRF if the subject was fasting at the time of blood sampling.

7.2.2.6.3 Thyroid function

Thyroid function assessments outlined in Table 7-7 will be performed according to the relevant Visit Evaluation Schedule (Table 7-1, Table 7-2, Table 7-3, or Table 7-4).

Thyroid function will be performed at:

- Screening/baseline: TSH, Free T3, Free T4

- Day 1 of subsequent cycles, EoT, 30-day safety follow-up: TSH only
    - If TSH is abnormal, central lab will test Free T3 and Free T4.

7.2.2.6.4 Coagulation

Coagulation assessments listed in Table 7-7 will be performed according to the relevant Visit Evaluation Schedule (Table 7-1, Table 7-2, Table 7-3, or Table 7-4).

7.2.2.6.5 Urinalysis

Urinalysis panel outlined in Table 7-7 will be assessed according to the relevant Visit Evaluation Schedule (Table 7-1, Table 7-2, Table 7-3, or Table 7-4).

Abnormal findings will be followed up with a microscopic evaluation and/or additional assessments as clinically indicated. A microscopic evaluation (WBC/HPF, RBC/HPF, and any other evaluations depending on macroscopic findings) need only to be performed if the urinalysis result is significantly abnormal.

7.2.2.6.6 Pregnancy and assessments of fertility

Women of child-bearing potential will have serum pregnancy tests within 72 hours prior to the first dose of study treatment. Monthly urine pregnancy tests will then be required to be performed on day 1 of every cycle beginning with cycle 2, followed by a serum pregnancy test at the End of Treatment visit, 30-Day and 150-Day Safety follow-up visit. After the 30-Day Safety Follow-up visit, women of child-bearing potential will perform at-home urine pregnancy testing every 30 days using kits provided until the 120-Day follow-up telephone call visit. Every effort must be made for the women of child bearing potential to return to the site for the final pregnancy test. However, if the subject is unable to return then the subject will administer the urine pregnancy test at home using the kit provided. For all pregnancy tests performed at home, the site personnel will follow-up with the subject via telephone call to collect the date and the test results and document the information in the subject’s source documents. If the subject returns to the site for the serum pregnancy test at the 150 day safety follow up the results must be documented in the subject’s source documents.

Women of child-bearing potential will be instructed to contact the site immediately at any time during the study (on-treatment or during follow-up) should they have a positive pregnancy test.

7.2.2.6.7 Hepatitis marker

Hepatitis panels listed in Table 7-7 will be performed at screening and as clinically indicated while on study treatment.

HBV-DNA serology (including HBV-DNA, HBsAg, HBsAb, HBcAb) and HCV RNA-PCR test will be performed at baseline screening (≤ 28 days prior to start of study treatment) and as clinically indicated (hepatitis markers should be evaluated for precautionary safety monitoring of viral re-activation while on study treatment).

During the screening period, subjects must be screened for HBV and HCV (current or past history of infection). Careful medical history must be taken for all subjects to look for risk factors (family history of HBV and HCV, intravenous drug abuse, unprotected sex, dialysis,

blood transfusions, etc.), and any past or present HBV symptoms (e.g., jaundice, dark urine, light colored stools, right upper quadrant pain).

Hepatitis B

At screening, all subjects will be tested for:

- HBV-DNA level
- Hepatitis B surface antigen (HBsAg)
- Hepatitis B core antibody (HBcAb)
- Hepatitis B surface antibody (HBsAb)

Hepatitis C

At screening, all subjects will be tested for quantitative HCV RNA-PCR.

After start of the study treatment and until 150-day safety follow-up, testing for HBV and HCV should be performed if clinically indicated (for example: rule out viral causality in case of DILI).

7.2.2.6.8 Cytokine analysis

Samples for cytokine analysis listed in Table 7-7 will be collected at the following time points:

- Screening/Baseline
- On an ad-hoc basis in case a subject has an adverse event suspected to be a cytokine release syndrome. In such case, this assessment should be performed at the following time points:
  - within 5 hours (or as soon as possible) after the occurrence of the adverse event,
  - one week after the occurrence of the adverse event.

Samples for cytokine panel at screening will be stored below -70°C.

7.2.2.6.9 HIV testing

For subjects in Germany only, a local test for HIV must be performed at screening according to the relevant Visit Evaluation Schedule (Table 7-1, Table 7-2, or Table 7-3).

7.2.2.7 Cardiac assessments

7.2.2.7.1 Electrocardiogram (ECG)

A standard 12 lead ECG will be performed according to the relevant Visit Evaluation Schedule (Table 7-1, Table 7-2, Table 7-3, or Table 7-4).

Standard 12 lead ECG (single or triplicate) will be performed after the subject has been resting for approximately 10 min prior to each ECG collection time point according to the relevant assessment schedule (Table 7-8 or Table 7-9). Additional, unscheduled, ECGs may be performed at the discretion of the investigator at any time during the study as clinically indicated. Unscheduled ECGs with clinically significant findings should be collected in triplicate.

Triplicate ECGs should be recorded approximately 2 minutes apart. The mean QTcF value should be calculated from each of the triplicate ECGs assessments. All ECGs need to be collected and copies kept in the medical record for central review, if requested by the sponsor.

Table 7-8 ECG collection plan for safety run-in (part 1), biomarker cohort (part 2), and randomized part (part 3)

Cycle	Day	Time	ECG Type	Number of ECG
Screening/baseline	-28 to -1	Anytime	12 Lead	3
2 [a]	1	1 hr after PDR001 (or placebo) administration	12 Lead	3
3 [b]	1	1 hr after PDR001 (or placebo) administration	12 Lead	3
Unscheduled	As clinical indicated	Anytime	12 Lead	1 (3 if clinically significant)
End of treatment		Anytime	12 Lead	1

[a.] Part 1: DL1 cohort only; Parts 2 & 3: if DL1 is determined to be the regimen
[b.] Part 1: DL-1a or DL-1b cohort only; Parts 2 & 3: if DL-1a or DL-1b is determined to be the regimen

Table 7-9 ECG collection plan for crossover

Crossover cycle	Day	Time	ECG Type	Number of ECG
1	1	Pre-dose	12 Lead	3
2 [a]	1	1 hr after PDR001 administration	12 Lead	3
3 [b]	1	1 hr after PDR001 administration	12 Lead	3
Unscheduled	As clinically indicated	Anytime	12 Lead	1 (3 if clinically significant)
Crossover End of treatment		Anytime	12 Lead	1

[a] If DL1 is determined to be the regimen
[b] If DL-1a or DL-1b is determined to be the regimen

Twelve (12)-lead ECGs will be obtained using an ECG machine that automatically calculates heart rate and measures PR, QRS, QT, and QTcF intervals. All ECG assessments will be performed in the supine position. ECG data will be read and interpreted locally.

Interpretation of the tracing must be made by a qualified physician and documented on the ECG eCRF page. Each ECG tracing should be labeled with the study number, subject initials (where regulations permit), subject number, date, and kept in the source documents at the study site.

Clinically significant abnormalities present when the subject signed informed consent should be reported on the Medical History eCRF page. Clinically significant findings must be discussed with Novartis prior to enrolling the subject in the study. New or worsened clinically significant findings occurring after informed consent must be recorded on the Adverse Events eCRF page.

7.2.2.7.2 Cardiac imaging - MUGA (multiple gated acquisition) scan or echocardiogram

Decreases of the LVEF have been observed in subjects receiving trametinib. Therefore, ECHO/MUGAs must be performed to assess cardiac ejection fraction in regular intervals according to the relevant schedule (Table 7-1, Table 7-2, Table 7-3, or Table 7-4).

The same procedure (either ECHO or MUGA, although ECHO is preferred) should be performed at baseline and at follow-up visit(s). Dose modification guidance and stopping criteria for LVEF decrease are provided in Table 6-22.

7.2.3 Pharmacokinetics

7.2.3.1 Pharmacokinetic blood collection and handling

Blood samples for PK analysis of PDR001, dabrafenib and trametinib will be collected according to the relevant Visit Evaluation Schedule (Table 7-1, Table 7-2, Table 7-3, or Table 7-4) and time points (Table 7-10, Table 7-11, Table 7-12, Table 7-13, Table 7-14, Table 7-15).

Blood samples will be taken by either direct venipuncture or an indwelling cannula inserted in a forearm vein opposite to the arm used for infusion.

A total of 4 mL of blood will be collected at specified time points for dabrafenib and trametinib analysis in plasma.

A minimum of 2 mL of blood will be collected for PDR001 analysis in serum. For time points when both PDR001 (mAb) PK and IG are to be measured, a single blood sample of approximately 5 mL will be collected (2 mL for PDR001 PK and 3 mL for IG). After clotting, the resulting serum will be separated in aliquots and will be stored frozen until analysis.

The exact date and clock times of drug administration and blood draw for PK and IG assessment will be recorded on the appropriate eCRF.

If vomiting occurs within 4 hours following dabrafenib and trametinib administration on the day of post dose PK blood sampling, the clock time of vomiting should be recorded on the Dose Administration Record PK eCRF page.

If subjects experience an SAE or an AE leading to the discontinuation of the study treatment, an unscheduled PK blood sample should be obtained as close as possible to the event occurrence. The date and time of the last dose and the time of PK blood draw should be recorded. If subjects experience suspected immunologically related AE such as infusion-related reaction, hypersensitivity, cytokine release syndrome and anaphylaxis, an unscheduled IG blood sample should be obtained as close as possible to the event occurrence. The date and time of the last dose and the time of PK blood draw should be recorded.

The residual PK and immunogenicity plasma or serum samples may be used for exploratory PK analysis such as metabolite analysis and exploratory biomarker analysis.

Refer to Study Laboratory Manual for detailed instructions for the collection, handling, and shipment of PK samples.

7.2.3.1.1 Pharmacokinetic and immunogenicity (IG) sampling for PDR001

PK and IG blood sampling schedule for PDR001 is outlined in Table 7-10, Table 7-11, and Table 7-12.

Table 7-10 Parts 1 & 2 Safety run-in and biomarker cohorts: Blood (serum) collection schedule for PDR001 PK and IG

Cycle	Day	Scheduled time point (hours)	Dose reference ID	PK Sample number	IG Sample number
1	1	Predose/0 hr [a]	1	101	201
1	1	End-of-Infusion [a] (within 2 hr)	1	102	
1	8	Anytime [a]	1	103	
1	15	Anytime [a]	1	104	
2	1	672 (±24h) hr / 0 hr pre-C2D1 dose	1/2 [b]	105	202
2	15	Anytime	2	106	
3	1	672 (±24h) hr / 0 hr pre-C3D1 dose	2/3 [b]	107	203
3	1	End-of-Infusion (within 2 hr)	3	108	
4	1	672 (±24h) hr / 0 hr pre-C4D1 dose	3/4 [b]	109	204
5	1	Predose/0 hr	5	110	205
6	1	Predose/0 hr	6	111	206
7	1	Predose/0 hr	7	112	207
8	1	Predose/0 hr	8	113	208
9	1	Predose/0 hr	9	114	209
10	1	Predose/0 hr	10	115	210
11	1	Predose/0 hr	11	116	211
12	1	Predose/0 hr	12	117	212
Every 6 Cycle after C12D1	1	Predose/0 hr	13+	118+	213+
EOT	-	Anytime	N/A	197	297
30-day safety follow-up visit [c]	-	Anytime	N/A	198	298
150-day safety follow-up visit [c]	-	Anytime	N/A	199	299
Unscheduled and at the time of progression or AE	N/A	Anytime	N/A	1001+	2001+

[a] PK and immunogenicity samples for PDR001 will not be collected during Cycle 1 for the DL-1b dosing regimen during Part 1, or if DL-1b is the selected dosing regimen for Part 2, and at Cycle 1 Day 8 for Part 2.

[b] The first Dose Reference ID (DRID) is for last dose the patient received prior to the collection of the PK sample, while the second DRID is for the current dose

[c] If PDR001 is permanently discontinued prior to dabrafenib and/or trametinib, 30 day and 150 day safety follow up visits should be based on the last dose of PDR001.

- All predose samples must be collected within 30 min before the infusion begins
- An unscheduled PK samples and immunogenicity sample should be collected up on confirmed disease progression.
- Unscheduled PK and immunogenicity samples may be collected at any time if clinically indicated or at the Investigator's discretion and must be sequentially numbered as 1001, 1002, 1003, etc. for PK samples, and 2001, 2002, 2003, etc. for immunogenicity samples.
- After the primary Clinical Study Report (CSR) data cut-off date is reached, no additional PK and IG samples will be collected for the subjects still on-going on the study.

Table 7-11 Part 3 Double-blind, randomized, placebo-controlled part: Blood (serum) collection schedule for PDR001/placebo PK and IG

Cycle	Day	Scheduled time point (hours)	Dose reference ID	PK Sample number	IG Sample number
1	1	Pre-C1D1 dose [a]	1	301	401
1	1	End-of-Infusion [a] (within 2 hr)	1	302	
2	1	Predose/0 hr	2	303	402
3	1	Predose/0 hr	3	304	403
3	1	End-of-Infusion (within 2 hr)	3	305	
4	1	Predose/0 hr	4	306	404
5	1	Predose/0 hr	5	307	405
6	1	Predose/0 hr	6	308	406
7	1	Predose/0 hr	7	309	407
8	1	Predose/0 hr	8	310	408
9	1	Predose/0 hr	9	311	409
10	1	Predose/0 hr	10	312	410
11	1	Predose/0 hr	11	313	411
12	1	Predose/0 hr	12	314	412
Every 6 Cycle after C12D1	1	Predose/0 hr	13+	315+	413+
EOT	-	Anytime	N/A	397	497
30-day safety follow-up visit [b.]	-	Anytime	N/A	398	498
150-day safety follow-up visit [b.]	-	Anytime	N/A	399	499
Unscheduled and at the time of progression or AE	N/A	Anytime	N/A	3001+	4001+

[a] If DL-1b is the selected dosing regimen for the randomized part (part 3), PK and IG samples for PDR001 will not be collected during Cycle 1.

[b].If PDR001/placebo is permanently discontinued prior to dabrafenib and/or trametinib, 30 day and 150 day safety follow up visits should be based on the last dose of PDR001/placebo.

- All predose samples must be collected within 30 min before the infusion begins
- An unscheduled PK samples and IG samples should be collected up on confirmed disease progression.
- Unscheduled PK and IG samples may be collected at any time if clinically indicated or at the Investigator's discretion and must be sequentially numbered as 3001, 3002, 3003, etc. for PK samples, and 4001, 4002, 4003, etc. for IG samples.
- After the primary CSR data cut-off date is reached, no additional PK and IG samples will be collected for the subjects still on-going on the study.

Table 7-12 Crossover subjects only: Blood (serum) collection schedule for PDR001 PK and IG

Crossover Cycle	Day	Scheduled time point (hours)	Dose reference ID	PK Sample number	IG Sample number
1	1	Pre-C1D1 dose [a]	1	501	601
1	1	End-of-Infusion [a] (within 2 hr)	1	502	
2	1	Predose/0 hr	2	503	602
3	1	Predose/0 hr	3	504	603
3	1	End-of-Infusion (within 2 hr)	3	505	
4	1	Predose/0 hr	4	506	604
5	1	Predose/0 hr	5	507	605
6	1	Predose/0 hr	6	508	606
7	1	Predose/0 hr	7	509	607
8	1	Predose/0 hr	8	510	608
9	1	Predose/0 hr	9	511	609
10	1	Predose/0 hr	10	512	610
11	1	Predose/0 hr	11	513	611
12	1	Predose/0 hr	12	514	612
Every 6 Cycle after C12D1	1	Predose/0 hr	13+	515+	613+
EOT	-	Anytime	N/A	597	697
30-day safety follow-up visit [b]	-	Anytime	N/A	598	698
150-day safety follow-up visit [b]	-	Anytime	N/A	599	699
Unscheduled	N/A	Anytime	N/A	5001+	6001+

[a] If DL-1b is the selected dosing regimen for the randomized part (part 3), PK and IG samples for PDR001 will not be collected during Cycle 1.

[b] If PDR001 is permanently discontinued prior to dabrafenib and/or trametinib, 30 day and 150 day safety follow up visits should be based on the last dose of PDR001.

- All predose samples must be collected within 30 min before the infusion begins
- An unscheduled PK samples and IG samples should be collected up on confirmed disease progression.
- Unscheduled PK and IG samples may be collected at any time if clinically indicated or at the Investigator's discretion and must be sequentially numbered as 5001, 5002, 5003, etc. for PK samples, and 6001, 6002, 6003, etc. for IG samples.
- After the primary CSR data cut-off date is reached, no additional PK and IG samples will be collected for the subjects still on-going on the study.

7.2.3.1.2 Pharmacokinetic sampling for dabrafenib and trametinib

PK blood sampling schedule for dabrafenib and trametinib is outlined in Table 7-13, Table 7-14, and Table 7-15.

Table 7-13 Part 1 & 2 Safety run-in and biomarker cohorts: Pharmacokinetic blood (plasma) collection schedule for trametinib and dabrafenib

Cycle	Day	Scheduled time point (hours)	Trametinib Dose reference ID	Dabrafenib Dose reference ID	Trametinib Dose reference ID	Dabrafenib Dose reference ID	PK Sample number
			Refers to the last dose taken before PK collection		Refers to the dose taken on the day of PK collection		
1	1	Predose/0 hr			31	61	701
1	15	Predose/0 hr	321	621	32	62	702
1	15	Between 1 and 3 hr post-dose			32	62	703
2	1	Predose/0 hr	331	631	33	63	704
2	15	Predose/0 hr	341	641	34	64	705
3	1	Predose/0 hr	351	651	35	65	706
3	1	Between 2 and 12 hr post-dose			35	65	707
4	1	Predose/0hr	361	661	36	66	708
4	1	Between 2 and 12 hr post-dose			36	66	709
5	1	Predose/0 hr	371	671	37	67	710
6	1	Predose/0 hr	381	681	38	68	711
7	1	Predose/0 hr	391	691	39	69	712
8	1	Predose/0 hr	401	701	40	70	713
9	1	Predose/0 hr	411	711	41	71	714
10	1	Predose/0 hr	421	721	42	72	715
11	1	Predose/0 hr	431	731	43	73	716
12	1	Predose/0 hr	441	741	44	74	717
Every 6 Cycle after C12D1	1	Predose/0 hr	451+	751+	45+	75+	718+
EOT	1	Anytime	N/A	N/A	N/A	N/A	799
Unscheduled	N/A	Anytime	N/A	N/A	N/A	N/A	7001+

- All predose samples must be collected prior to dosing on PK day. For pre-dose samples, the date and time of the last dose taken before PK collection and the date and time of the first dose taken after PK collection should be recorded in Dose Administration Record (DAR) for dabrafenib and trametinib CRF page. An unscheduled PK sample should be collected up on confirmed disease progression.
- Unscheduled PK sample may be collected at any time if clinically indicated or at the Investigator's discretion and must be sequentially numbered as 7001, 7002, 7003, etc.
- After the primary CSR data cut-off date is reached, no additional PK samples will be collected for the subjects still on-going on the study.

Table 7-14 Part 3 Double-blind, randomized, placebo-controlled part: Pharmacokinetic blood (plasma) collection schedule for trametinib and dabrafenib

Cycle	Day	Scheduled time point (hours)	Trametinib Dose reference ID	Dabrafenib Dose reference ID	Trametinib Dose reference ID	Dabrafenib Dose reference ID	PK Sample number
			Refers to the last dose taken before PK collection		Refers to the dose taken on the day of PK collection		
1	1	Predose/0 hr			31	61	801
2	1	Predose/0 hr	321	621	32	62	802
2	1	Between 1 and 3 hr post-dose			32	62	803
3	1	Predose/0 hr	331	631	33	63	804
3	1	Between 2 and 12 hr post-dose			33	63	805
4	1	Predose/0 hr	341	641	34	64	806
4	1	Between 2 and 12 hr post-dose			34	64	807
5	1	Predose/0 hr	351	651	35	65	808
6	1	Predose/0 hr	361	661	36	66	809
7	1	Predose/0 hr	371	671	37	67	810
8	1	Predose/0 hr	381	681	38	68	811
9	1	Predose/0 hr	391	691	39	69	812
10	1	Predose/0 hr	401	701	40	70	813
11	1	Predose/0 hr	411	711	41	71	814
12	1	Predose/0 hr	421	721	42	72	815
Every 6 Cycle after C12D1	1	Predose/0 hr	431+	731+	43+	73+	816+
EOT	1	Anytime	N/A	N/A	N/A	N/A	899
Unscheduled	N/A	Anytime	N/A	N/A	N/A	N/A	8001+

- All predose samples must be collected prior to dosing on PK day. For pre-dose samples, the date and time of the last dose taken before PK collection and the date and time of the first dose taken after PK collection should be recorded in DAR for dabrafenib and trametinib CRF page. An unscheduled PK sample should be collected up on confirmed disease progression.
- Unscheduled PK sample may be collected at any time if clinically indicated or at the Investigator's discretion and must be sequentially numbered as 3001, 3002, 3003, etc.
- After the primary CSR data cut-off date is reached, no additional PK samples will be collected for the subjects still on-going on the study.

Table 7-15 Crossover subjects only: Pharmacokinetic blood (plasma) collection schedule for trametinib and dabrafenib

Crossover Cycle	Day	Scheduled time point (hours)	Trametinib Dose reference ID	Dabrafenib Dose reference ID (	Trametinib Dose reference ID	Dabrafenib Dose reference ID	PK Sample number
			Refers to the last dose taken before PK collection		Refers to the dose taken on the day of PK collection		
1	1	Predose/0 hr			31	61	901
2	1	Predose/0 hr	321	621	32	62	902
2	1	Between 1 and 3 hr post-dose			32	62	903
3	1	Predose/0 hr	331	631	33	63	904
3	1	Between 2 and 12 hr post-dose			33	63	905
4	1	Predose/0hr	341	641	34	64	906
4	1	Between 2 and 12 hr post-dose			34	64	907
5	1	Predose/0 hr	351	651	35	65	908
6	1	Predose/0 hr	361	661	36	66	909
7	1	Predose/0 hr	371	671	37	67	910
8	1	Predose/0 hr	381	681	38	68	911
9	1	Predose/0 hr	391	691	39	69	912
10	1	Predose/0 hr	401	701	40	70	913
11	1	Predose/0 hr	411	711	41	71	914
12	1	Predose/0 hr	421	721	42	72	915
Every 6 Cycle after C12D1	1	Predose/0 hr	431+	731+	43+	73+	916+
EOT	1	Anytime	N/A	N/A	N/A	N/A	999
Unscheduled	N/A	Anytime	N/A	N/A	N/A	N/A	10001+

- All predose samples must be collected prior to dosing on PK day. For pre-dose samples, the date and time of the last dose taken before PK collection and the date and time of the first dose taken after PK collection should be recorded in DAR for dabrafenib and trametinib CRF page. An unscheduled PK sample should be collected up on confirmed disease progression.
- Unscheduled PK sample may be collected at any time if clinically indicated or at the Investigator's discretion and must be sequentially numbered as 10001, 10002, 10003, etc.
- After the primary CSR data cut-off date is reached, no additional PK samples will be collected for the subjects still on-going on the study.

7.2.3.2 Analytical method

Bioanalysis for PK and IG assessment will employ the validated assays:

1. The assay to quantify PDR001 is a validated LC/MS assay.
2. The assays to quantify and assess IG will be a validated three-tiered ELISA assays (screening, confirmatory, and titer assays).
3. Plasma concentrations of dabrafenib and its metabolites hydroxy-dabrafenib (GSK2285403), carboxy-dabrafenib (GSK2298683), and desmethyl-dabrafenib

(GSK2167542) as well as trametinib will be measured using validated liquid chromatography/mass spectrometry/ mass spectrometry (LC/MS/MS) methods.

The details of the assays will be documented in the Study Laboratory Manual.

7.2.4 Biomarkers

Biomarker analyses will be used to investigate the effect of the PDR001 at the molecular and cellular level as well as to determine how changes in the biomarkers may relate to exposure and clinical outcomes. In addition, potential predictive biomarkers of efficacy, as well as mechanisms of resistance to PDR001 treatment will also be explored. Samples and data may be also used to support development of future companion diagnostic tests.

During the study, both blood and tumor samples will be collected to centrally confirm the *BRAF* V600 mutation status and to perform exploratory biomarker assessments. These are summarized in Table 7-16 and Table 7-17 .

All assessments will be performed by a Novartis designated laboratory. Instructions for collection, storage and shipment of all biomarker samples will be provided in the CPDR001F2301 laboratory manual. Required sample collection information must be entered on the appropriate eCRF pages and requisition forms.

While the goal of the biomarker study is to provide supportive data for the clinical study, there may be circumstances when a decision is made to stop a collection, or not perform or discontinue an analysis due to either practical or strategic reasons. For example, there may be inadequate sample number, issues related to the quality of the sample or issues related to the assay that preclude analysis. Alternatively, there may be insufficient efficacy to allow for correlative analyses. Therefore, depending on the results obtained during the study, collection/analysis of some samples may be omitted at the discretion of the sponsor.

Table 7-16 Biomarker sample collection plan for Safety run-in (part 1) and double-blind, randomized, placebo-controlled parts (part 3)

**Biomarker sample collection plan**			
**Sample Type**	**Volume**	**Visit**	**Time Point**
**Tumor samples**			
Mandatory<br>Newly acquired tumor biopsy (preferred) or archival tumor biopsy (if newly acquired tumor sample cannot be collected) must be provided for central *BRAF* V600 mutation testing/confirmation, assessment of IO markers and a potential companion diagnostic bridging study<br>(Core, excisional and incisional biopsies are acceptable. Fine needle aspirates are not acceptable sample types.)	A formalin-fixed new biopsy (preferred) or formalin fixed paraffin embedded (FFPE) archival tumor block. If tumor block is unavailable, a minimum of 25 slides must be submitted.	Screening	Anytime during Screening prior to study treatment on C1D1.<br>Note:<br>1. If local *BRAF* V600 mutation test result is not available, samples should be sent for testing by **Day -20**.<br>2. If an archival sample is being submitted, then samples collected within **3 months** prior to study treatment initiation is strongly recommended.<br>3. If slides are being

Biomarker sample collection plan			
**Sample Type**	**Volume**	**Visit**	**Time Point**
**Tumor samples**			
			provided, only freshly cut slides are acceptable. Slide cut date must be provided on the requisition form.
Optional<br>Newly acquired tumor biopsy<br>(Core, excisional and incisional biopsies are acceptable. Fine needle aspirates are not acceptable sample types.)	Please refer to the lab manual	Screening	Anytime during Screening prior to study treatment on C1D1.
		Cycle 1 Day 15<br>(Part 3)	C1D15 + 7 day window
		Cycle 3<br>(Part 1)<br>(Part 3 only if DL1 or DL-1a is determined to be the regimen)	Anytime
		Cycle 4<br>(Part 3 only if DL-1b is determined to be the regimen)	Anytime
		Unscheduled (at disease progression per RECIST 1.1 and/or at a subsequent PD per response criteria for immunotherapy if subject continues treatment post progression).	At the time of disease progression, prior to the start of new antineoplastic therapy
**Blood samples**			
Mandatory<br>Blood for germline DNA	6 mL	Anytime	Anytime
Mandatory<br>Blood for circulating tumor DNA (ctDNA) isolation	10 mL	Cycle 1 Day 1	Pre-dose
	10 mL	Cycle 2 Day 1	Pre-dose
	10 mL	Cycle 3 Day 1<br>(Part 1)<br>(Part 3 only if DL1 or DL-1a is determined to be the regimen)	Pre-dose
		Cycle 4 Day 1<br>(Part 3 only if DL-1b is determined to be the regimen)	Pre-dose

Biomarker sample collection plan			
**Sample Type**	**Volume**	**Visit**	**Time Point**
**Tumor samples**			
	10 mL	Unscheduled (at disease progression per RECIST 1.1 and/or at a subsequent PD per response criteria for immunotherapy if subject continues treatment post progression).	At the time of disease progression, prior to the start of new antineoplastic therapy
Mandatory<br>Blood for circulating tumor RNA (ctRNA) isolation	10 mL	Cycle 1 Day 1	Pre-dose
	10 mL	Cycle 2 Day 1	Pre-dose
	10 mL	Cycle 3 Day 1<br>(Part 1)<br>(Part 3 only if DL1 or DL-1a is determined to be the regimen)	Pre-dose
		Cycle 4 Day 1<br>(Part 3 only if DL-1b is determined to be the regimen)	Pre-dose
	10 mL	Unscheduled (at disease progression per RECIST 1.1 and/or at a subsequent PD per response criteria for immunotherapy if subject continues treatment post progression).	At the time of disease progression, prior to the start of new antineoplastic therapy
Mandatory<br>Blood for cytokines	10 mL	Cycle 1 Day 1	Pre-dose
	10 mL	Cycle 2 Day 1	Pre-dose
	10 mL	Cycle 3 Day 1<br>(Part 1)<br>(Part 3 only if DL1 or DL-1a is determined to be the regimen)	Pre-dose
		Cycle 4 Day 1<br>(Part 3 only if DL-1b is determined to be the regimen)	Pre-dose
	10 mL	Unscheduled (at disease progression per RECIST 1.1 and/or at a subsequent PD per response criteria for immunotherapy if subject continues treatment post progression).	At the time of disease progression, prior to the start of new antineoplastic therapy

Biomarker sample collection plan			
**Sample Type**	**Volume**	**Visit**	**Time Point**
**Tumor samples**			
Mandatory Blood for PBMCs (for TCR profiling)	10 mL (Part 1)<br>12 mL (Part 3)	Cycle 1 Day 1	Pre-dose
	10 mL (Part 1)<br>12 mL (Part 3)	Cycle 2 Day 1	Pre-dose
	10 mL (Part 1)<br>12 mL (Part 3)	Cycle 3 Day 1<br>(Part 1)<br>(Part 3 only if DL1 or DL-1a is determined to be the regimen)	Pre-dose
		Cycle 4 Day 1<br>(Part 3 only if DL-1b is determined to be the regimen)	Pre-dose
	10 mL (Part 1)<br>12 mL (Part 3)	Unscheduled (at disease progression per RECIST 1.1 and/or at a subsequent PD per response criteria for immunotherapy if subject continues treatment post progression).	At the time of disease progression, prior to the start of new antineoplastic therapy
**Applicable for Crossover subjects only**			
**Sample Type**	**Volume**	**Visit**	**Time Point**
Mandatory Blood for PBMCs	12 mL	Cross over Cycle 1 Day 1	Pre-dose
	12 mL	Cross over Cycle 3 Day 1	Pre-dose
	12 mL	Unscheduled (at disease progression per RECIST 1.1 and/or at a subsequent PD per response criteria for immunotherapy if subject continues treatment post progression).	At the time of disease progression, prior to the start of new antineoplastic therapy
Mandatory Blood for cytokines	10 mL	Crossover Cycle 1 Day 1	Pre-dose
	10 mL	Crossover Cycle 3 Day 1	Pre-dose

Supplemental material
BMJ Publishing Group Limited (BMJ) disclaims all liability and responsibility arising from any reliance placed on this supplemental material which has been supplied by the author(s)

Biomarker sample collection plan			
**Sample Type**	**Volume**	**Visit**	**Time Point**
**Tumor samples**			
	10 mL	Unscheduled (at disease progression per RECIST 1.1 and/or at a subsequent PD per response criteria for immunotherapy if subject continues treatment post progression).	At the time of disease progression, prior to the start of new antineoplastic therapy
Optional<br>Newly acquired tumor biopsy for IO assessment<br>(Core, excisional and incisional biopsies are acceptable. Fine needle aspirates are not acceptable sample types.)	Please refer to the lab manual	Crossover Cycle 3	Anytime
		Unscheduled (at disease progression per RECIST 1.1 and/or at a subsequent PD per response criteria for immunotherapy if subject continues treatment post progression).	At the time of disease progression, prior to the start of new antineoplastic therapy

Table 7-17 Biomarker sample collection plan for Biomarker cohort (part 2)

Biomarker sample collection plan			
**Sample Type**	**Volume**	**Visit**	**Time Point**
**Tumor samples**			
Mandatory<br>Newly acquired tumor biopsy must be provided for central *BRAF* V600 mutation testing/confirmation, assessment of immune biomarkers and potential companion diagnostic bridging study.<br>(Core, excisional and incisional biopsies are acceptable. Fine needle aspirates are not acceptable sample types.)	A formalin-fixed new biopsy must be provided. Please refer to the lab manual provided.	Screening	Anytime during Screening prior to study treatment on C1D1.<br>Note: If local result is not available, sample should be sent for testing by **Day -20**.
Mandatory<br>Newly acquired tumor biopsy must be provided for assessment of IO markers.<br>(Core, excisional and incisional biopsies are acceptable. Fine needle	Please refer to the lab manual	Cycle 1 Day 15	C1D15 + 7 day window
		Cycle 3 (if DL1 or DL-1a is determined to be the regimen)<br>Cycle 4 (if DL-1b is determined to be the regimen)	Anytime

Biomarker sample collection plan			
**Sample Type**	**Volume**	**Visit**	**Time Point**
aspirates are not acceptable sample types.)		Unscheduled (at disease progression per RECIST 1.1 and/or at a subsequent PD per response criteria for immunotherapy if subject continues treatment post progression).	At the time of disease progression prior to the start of new antineoplastic therapy
Optional<br>Newly acquired tumor biopsy for IO assessment<br>(Core, excisional and incisional biopsies are acceptable. Fine needle aspirates are not acceptable sample types.)	Please refer to the lab manual	Anytime<br>Depending on investigator assessment	
**Blood samples**			
Mandatory<br>Blood for germline DNA	6 mL	Anytime	Anytime
Mandatory<br>Blood for circulating tumor DNA (ctDNA) isolation	10 mL	Cycle 1 Day 1	Pre-dose
	10 mL	Cycle 1 Day 15	Pre-dose
	10 mL	Cycle 3 Day 1 (if DL1 or DL-1a is determined to be the regimen)<br>Cycle 4 day 1 (if DL-1b is determined to be the regimen)	Pre-dose
	10 mL	Unscheduled (at disease progression per RECIST 1.1 and/or at a subsequent PD per response criteria for immunotherapy if subject continues treatment post progression).	At the time of disease progression, prior to the start of new antineoplastic therapy
Mandatory<br>Blood for circulating tumor RNA (ctRNA) isolation	10 mL	Cycle 1 Day 1	Pre-dose
	10 mL	Cycle 1 Day 15	Pre-dose
	10 mL	Cycle 3 Day 1 (if DL1 or DL-1a is determined to be the regimen)<br>Cycle 4 day 1 (if DL-1b is determined to be the regimen)	Pre-dose

Biomarker sample collection plan			
**Sample Type**	**Volume**	**Visit**	**Time Point**
	10 mL	Unscheduled (at disease progression per RECIST 1.1 and/or at a subsequent PD per response criteria for immunotherapy if subject continues treatment post progression).	At the time of disease progression, prior to the start of new antineoplastic therapy
Mandatory<br>Blood for cytokines	10 mL	Cycle 1 Day 1	Pre-dose
	10 mL	Cycle 2 Day 1	Pre-dose
	10 mL	Cycle 3 Day 1 (if DL1 or DL-1a is determined to be the regimen)<br>Cycle 4 Day 1 (if DL-1b is determined to be the regimen)	Pre-dose
	10mL	Unscheduled (at disease progression per RECIST 1.1 and/or at a subsequent PD per response criteria for immunotherapy if subject continues treatment post progression).	At the time of disease progression, prior to the start of new antineoplastic therapy
Mandatory<br>Blood for PBMCs (for TCR profiling)	10 mL	Cycle 1 Day 1	Pre-dose
	10 mL	Cycle 2 Day 1	Pre-dose
	10 mL	Cycle 3 Day 1 (if DL1 or DL-1a is determined to be the regimen)<br>Cycle 4 Day 1(if DL-1b is determined to be the regimen)	Pre-dose
	10 mL	Unscheduled (at disease progression per RECIST 1.1 and/or at a subsequent PD per response criteria for immunotherapy if subject continues treatment post progression).	At the time of disease progression, prior to the start of new antineoplastic therapy

7.2.4.1 Biomarker assessments in tumor tissue

7.2.4.1.1 Mandatory tumor sample collection

A mandatory newly acquired biopsy (preferred) or an archival specimen obtained at or since the time of diagnosis (within **3 months** prior to study treatment start is strongly recommended) must be provided during screening prior to the first dose of study treatment.

CNS and bone are excluded organs for biopsy and biomarker analyses.

For biomarker cohort only: A mandatory **newly acquired biopsy** specimen must be provided from cutaneous or subcutaneous lesions or nodal lesions during screening, at Cycle 1 Day 15 (+ 1 week window), 8-12 weeks after initiation of PDR001 therapy and at PD (at disease progression per RECIST 1.1 and/or at a subsequent disease progression per response criteria for immunotherapy if subject continues treatment post progression). If the newly acquired biopsy sample provided is determined to be insufficient for the proposed biomarker analyses, additional archival tumor tissue sample may be requested for the central BRAF V600 mutation confirmation.

All subjects will be required to provide a tumor tissue sample at screening prior to study treatment, as either a tumor block or a minimum of 25 FFPE slides. The minimum number of slides is indicated in order to have sufficient material for the central *BRAF* V600 mutation testing/confirmation and additional exploratory biomarker studies as described below:

- If local documentation of *BRAF* V600 mutation status is available, *BRAF* V600 mutation results will be subjected to retrospective central confirmation by a Novartis designated laboratory and FDA approved assay (e.g., Biomerieux THxID™-BRAF).
- In cases where an acceptable local result for *BRAF* V600 mutation is not available, a central test must be prospectively performed and the tumor sample should be sent to a Novartis designated central laboratory for testing by Day -20.

The remaining tissue material will be used to perform exploratory biomarker assessments, for instance to identify biomarkers that may be predictive of benefit from the combination of dabrafenib and trametinib with PDR001. Markers of the immune microenvironment including but not limited to PD-L1 levels and CD8+ cells in addition to other melanoma related markers will be investigated. These analyses may include, but are not limited to specific immune cell populations (e.g., CD8 positive T-cells, Tregs, macrophages) and immunohistochemistry assays. Biopsies may be also used for gene expression profiling (e.g., Nanostring platform, RNA sequencing) to correlate selected immune gene expression signatures with the efficacy data.

In addition, samples may be used for sequencing of selected genes or for whole exome sequencing to study the mutational landscape and tumor mutation burden as a measure of the potential neoantigenic potential for being recognized by immune cells.

Tumor tissue collected (irrespective of the local and central *BRAF* V600 status) may be used for development of a potential future companion or complementary diagnostic assays.

In the biomarker cohort, additional mandatory newly acquired biopsies will be collected at Cycle 1 Day 15 (+7 day window), 8 – 12 weeks after initiation of PDR001 therapy and at PD (at disease progression per RECIST 1.1 and/or at a subsequent disease progression per response criteria for immunotherapy if subject continues treatment post progression). The samples will

be used to assess the impact of dabrafenib, trametinib and PDR001 treatment on markers of the immune micro-environment. Similar biomarker analyses as in the baseline sample may be performed (e.g., selected IHC markers, gene expression profiling or sequencing) to assess quantitative changes as well as the presence or localization of immunological cells and markers in relation to the treatment.

The biopsy at disease progression will be used to unveil potential mechanism/marker of resistance to the treatment. For these purposes, analysis similar to the ones described above performed on the baseline and on treatment biopsies may also be performed on these samples. The goal of this analysis is to detect the presence of specific acquired somatic mutations or other genetic aberration that could account for acquired resistance to therapy, or to highlight changes in the neoantigenic potential of the tumor under the selective pressure of the treatment.

These analyses may also be used to support development of future companion diagnostic tests.

7.2.4.1.2 Optional tumor sample collection

In addition to the mandatory tissue collection, newly acquired biopsies are requested upon signature of the relevant section of the ICF. CNS and bone are excluded organs for biopsy and biomarker analyses.

In the safety run-in and randomized cohort, an optional newly acquired tumor tissue can be collected at screening (unless sufficient tissue from a newly acquired biopsy was already provided for *BRAF* V600 central testing), on Cycle 1 day 15 (+ 7 day window) (Part 3 only), 8-12 weeks after initiation of PDR001 therapy and at PD (at disease progression per RECIST 1.1 and/or at a subsequent PD per response criteria for immunotherapy if subject continues treatment post progression).

In the biomarker cohort, an additional optional new tumor tissue may be collected during the course of the study depending on investigator assessment (e.g., responding/shrinking lesion needs to be collected prior to cycle 3).

Tumor samples will be assessed by techniques such as IHC, Nanostring based gene expression profiling and sequencing to unveil potential mechanisms of sensitivity and resistance to the combination therapy.

7.2.4.2 Biomarker assessments in blood samples

7.2.4.2.1 Germline DNA analysis

A mandatory blood sample (6 mL) will be collected at any time during study from all subjects participating in the study and used to extract germline DNA. This DNA will be used to analyze the same sequences analyzed in tumor DNA derived from tumor tissue. This will allow to determine if eventual mutations found in tumor DNA are somatic gene alterations characteristic of tumors but are not present in germline DNA. Additional retrospective pharmacogenetic analysis may be conducted on germline DNA samples, but only in DNA of subjects that sign an additional Pharmacogenetic ICF. These additional analyses may be conducted to attempt identifying predictive genetic markers of suspected immune related AEs observed in the course of the trial.

7.2.4.2.2 Circulating tumor DNA assessment

All subjects participating in the study will be requested to provide whole blood samples, collected at study treatment baseline (Pre-dose) and at specific time point during treatment. Approximately 10 mL of whole blood will be collected to isolate circulating tumor DNA (ctDNA) at the visits as specified in Table 7-16 and Table 7-17. CtDNA can harbor genetic alterations (mutations, microsatellite alterations, aberrant methylation), which are generally consistent with the tumor DNA derived from the tumor tissue. CtDNA may also be explored to correlate increasing ctDNA levels with increasing tumor burden. Baseline levels and longitudinal changes over time of mutation load and selected ctDNAs may be assessed with respect to clinical endpoints. Assessment of ctDNA will allow additional testing for mutations of genes that are relevant to melanoma or PDR001. The ctDNA will be used to assess whether there are mutations at baseline or new mutations appearing during treatment and at time of progression, which are associated with treatment response.

7.2.4.2.3 Circulating tumor RNA assessment

Approximately 10 mL of whole blood will be collected at study treatment baseline (Pre-dose) and at specific time point during treatment at the visits as specified in Table 7-16 and Table 7-17 to investigate expression of circulating markers of the immune microenvironment utilizing evolving technologies such as in but not limited to circulating tumor RNA (ctRNA).

7.2.4.2.4 Peripheral blood mononuclear cells (PBMCs) assessment

PBMCs will be analyzed for characterization of T cell phenotypes in an effort to understand how baseline characteristics and changes from baseline may correlate with clinical response. Approximately 10 mL (or 12 mL in Part 3) of blood will be collected for PBMC assessment at the visits as indicated in Table 7-16 and Table 7-17 .

7.2.4.2.5 Plasma for cytokine assessment

Approximately 10 mL of blood will be collected for cytokine (and/or other circulating markers) assessment at the visits as indicated in Table 7-16 and Table 7-17. Correlation of plasma cytokine levels and/or other circulating markers with anti-tumor activity of the combination therapies may be explored.

All blood samples collected on the study for biomarker analysis may be interchangeably used for analyzing ctDNA, ctRNA, cytokines and other circulating markers of interest to address scientific questions related to PDR001, melanoma, treatment response or clinical outcome.

7.2.4.2.6 Optional additional exploratory biomarker assessments

If the subject agrees, the remaining biomarker samples (tumor, blood) may be stored for up to 15 years and further analyzed to address scientific questions related to PDR001 or cancer, including research to help develop ways to detect, monitor or treat cancer. A decision to perform such exploratory biomarker research studies would be based on the outcome of this study or from new scientific findings related to the drug class or disease, as well as reagent and assay availability.

7.2.4.2.7 Optional additional biomarkers assessments upon adverse event

If FFPE tissue is obtained as part of the evaluation of an AE that is suspected to be treatment-related (e.g., skin biopsy for rash), Novartis may request that remaining tissue to be submitted for central evaluation to gain further understanding of the AE, or other safety findings associated with that AE. For these reasons, additional analyses (similar to the ones described above for the baseline, on treatment and at disease progression biopsies) may be conducted on this material. In addition, an unscheduled blood sample collection may be requested in case of suspected adverse events and once the suspected adverse event is resolved.

7.2.5 Patient reported outcomes

The European Organization for Research and Treatment of Cancer's core quality of life questionnaire (EORTC-QLQ-C30, version 3.0), the EuroQoL 5-level instrument (EQ-5D-5L, tablet version), and the Melanoma Subscale of the Functional Assessment of Cancer Therapy–Melanoma (FACT-M) will be used to evaluate patient-reported outcome measures of health-related quality-of-life, functioning, disease symptoms, treatment-related side effects, and global health status. The EORTC QLQ-C30, EQ-5D-5L and Melanoma Subscale of the FACT-M are recognized as reliable and valid measures (Aaronson 1993, Rabin 2001, Cormier 2008) frequently used in clinical trials of patients with advanced or metastatic cancer.

All patient-reported outcomes (PRO) data will be collected using an electronic tablet device. All PRO assessments should be administered in the patients' local language according to the Visit Evaluation Schedule in Table 7-3 and Table 7-4, prior to any tests, treatments or receipt of results from any test to avoid biasing the patient's perspective.

Patients should be given sufficient space and time to complete all study questionnaires and all administered questionnaires should be reviewed for completeness. If missing responses are noted, patients should be encouraged to complete any missing responses. Attempts should be made to collect responses to all questionnaires for all patients, including from those who discontinue prior to the study evaluation completion visit, however, if patients refuse to complete questionnaires, this should be documented in study source records. Patient's refusal to complete study questionnaires are not protocol deviations.

Completed questionnaires, including both responses to the questions and any unsolicited comments written by the patient, should be reviewed and assessed by the investigator before the clinical examination for responses which may indicate potential AEs or SAEs. This review should be documented in study source records.

If an AE or SAE is confirmed then the physician should record the event as instructed in Section 8 of this protocol. Investigators should not encourage the patients to change responses reported in questionnaires.

7.2.5.1 EORTC QLQ-C30

The EORTC QLQ-C30 contains 30 items and is composed of both multi-item scales and single-item measures. These include five functional scales (physical, role, emotional, cognitive and social functioning), three symptom scales (fatigue, nausea/vomiting, and pain), six single items (dyspnea, insomnia, appetite loss, constipation, diarrhea and financial impact) and a global health status/QoL scale (Aaronson 1993).

All of the scales and single-item measures range in score from 0 to 100. A high scale score represents a higher response level. Thus, a high score for a functional scale represents a high / healthy level of functioning; a high score for the global health status / QoL represents a high QoL, but a high score for a symptom scale / item represents a high level of symptomatology / problems. All scoring will follow the scoring procedures defined by the EORTC Scoring Manual (Fayers 2001).

7.2.5.2 EQ-5D-5L

The EQ-5D-5L (tablet version) is a standardized measure of health utility that provides a single index value for one's health status. The EQ-5D-5L is frequently used for economic evaluations of health care and has been shown to be a valid and reliable instrument (The EuroQol Group 1990, Rabin 2001). The EQ-5D-5L contains one item for each of five dimensions of Health Related QoL (HRQoL) (i.e., mobility, self-care, usual activities, pain or discomfort, and anxiety or depression). Response options for each item vary from having no problems (e.g., "…no problems walking about"), moderate problems (e.g., "…some problems walking about"), or extreme problems (e.g., "…unable to walk about"). Subject responses to the five dimensions of HRQoL reflect a specific health state that corresponds to a population preference weight for that state on a continuous scale of 0 (death) to 1 (perfect health). A visual analog scale (ranging from 0 to 100) is also included to capture subject's rating of their overall health status. Higher scores of the EQ-5D-5L represent better health states. All scoring and handling of data will follow the User's Guide defined by the EuroQoL Group (Reenen 2015).

7.2.5.3 Functional Assessment of Cancer Therapy–Melanoma (FACT-M)

The Functional Assessment of Cancer Therapy–Melanoma (FACT-M) quality of life questionnaire consists of the FACT-General (FACT-G) plus the Melanoma Subscale and the Melanoma Surgery Subscale, which complement the general scale with items specific to quality of life (QoL) in melanoma. Higher scores on all the FACT-M scales indicate a higher quality of life. In psychometric testing FACT-M questionnaire has been shown to be a reliable and valid instrument for subjects with melanoma that can be used for the assessment of QoL in clinical trials (Cormier 2008). For the purpose of this study the 16 items that comprise the Melanoma Subscale of the FACT-M are used.

8 Safety monitoring and reporting

8.1 Adverse events

8.1.1 Definitions and reporting

An adverse event is defined as the appearance of (or worsening of any pre-existing) undesirable sign(s), symptom(s), or medical condition(s) that occur after subject's signed informed consent has been obtained.

Subjects whose *BRAF* V600 mutation status is known will sign the main study ICF.

Abnormal laboratory values or test results occurring after informed consent constitute adverse events only if they induce clinical signs or symptoms, are considered clinically significant,

require therapy (e.g., hematologic abnormality that requires transfusion or hematological stem cell support), or require changes in study medication(s).

Adverse events that begin or worsen after informed consent should be recorded in the Adverse Events eCRF. Conditions that were already present at the time of informed consent should be recorded in the Medical History page of the subject's CRF. Adverse event monitoring should be continued for at least:

- 150 days following the last dose of PDR001/placebo
- until the start of a new post-treatment antineoplastic medication if sooner than the 150 days mentioned above. If a subject starts a post-treatment antineoplastic therapy, then only adverse events suspected to be related to study treatment should be collected out to 150 days after discontinuation of PDR001/placebo.
- 30 days after the last dose of dabrafenib or 120 days after the last dose of trametinib, if the subject continued the combination partner more than 150 days after the last dose of PDR001/placebo)

Adverse events (including lab abnormalities that constitute AEs) should be described using a diagnosis whenever possible, rather than individual underlying signs and symptoms. When a clear diagnosis cannot be identified, each sign or symptom should be reported as a separate Adverse Event.

Adverse events will be assessed and graded according to the CTCAE v4.03 (**...//ctep.cancer.gov**).

If CTCAE grading does not exist for an adverse event, the severity of mild, moderate, severe, and life-threatening, death related to the AE corresponding respectively to Grades 1 - 5, will be used. Information about any deaths (related to an Adverse Event or not) will also be collected though a Death form.

The occurrence of adverse events should be sought by non-directive questioning of the subject during the screening process after signing informed consent and at each visit during the study. Adverse events also may be detected when they are volunteered by the subject during the screening process or between visits, or through physical examination, laboratory test, or other assessments. As far as possible, each adverse event should be evaluated to determine:

1. The severity grade (CTCAE v4.03 Grade 1-5)
2. Its duration (Start and end dates)
3. Its relationship to the study treatment (Reasonable possibility that AE is related: No, Yes, investigational treatment, Yes, the study treatment (non-investigational), Yes, both and/or indistinguishable)
4. Action taken with respect to study or investigational treatment (none, dose adjusted, temporarily interrupted, permanently discontinued, unknown, not applicable)
5. Whether medication or therapy was given (no concomitant medication/non-drug therapy, concomitant medication/non-drug therapy)
6. Whether it is serious, where a serious adverse event (SAE) is defined as in Section 8.2.1 and which seriousness criteria have been met
7. Outcome (not recovered/not resolved, recovered/resolved, recovering/resolving, recovered/resolved with sequelae, fatal, unknown)

If the event worsens the event should be reported a second time in the eCRF noting the start date when the event worsens in toxicity. For Grade 3 and 4 adverse events only, if improvement to a lower grade is determined a new entry for this event should be reported in the eCRF noting the start date when the event improved from having been Grade 3 or Grade 4.

For safety run-in part, any AE that constitutes a DLT should be reported like a Grade 3 and 4 adverse event.

All adverse events should be treated appropriately. If a concomitant medication or non-drug therapy is given, this action should be recorded on the Adverse Event eCRF.

Once an adverse event is detected, it should be followed until its resolution or until it is judged to be permanent, and assessment should be made at each visit (or more frequently, if necessary) of any changes in severity, the suspected relationship to the study treatment, the interventions required to treat it, and the outcome.

Progression of malignancy (including fatal outcomes), if documented by use of appropriate method (for example, as per RECIST criteria for solid tumors), should not be reported as a serious adverse event.

Adverse events separate from the progression of malignancy (example, deep vein thrombosis at the time of progression or hemoptysis concurrent with finding of disease progression) will be reported as per usual guidelines used for such events with proper attribution regarding relatedness to the drug.

8.1.2 Laboratory test abnormalities

8.1.2.1 Definitions and reporting

Laboratory abnormalities that constitute an Adverse event in their own right (are considered clinically significant, induce clinical signs or symptoms, require concomitant therapy or require changes in study treatment), should be recorded on the Adverse Events eCRF. Whenever possible, a diagnosis, rather than a symptom should be provided (e.g., anemia instead of low hemoglobin). Laboratory abnormalities that meet the criteria for Adverse Events should be followed until they have returned to normal or an adequate explanation of the abnormality is found. When an abnormal laboratory or test result corresponds to a sign/symptom of an already reported adverse event, it is not necessary to separately record the lab/test result as an additional event.

Laboratory abnormalities, that do not meet the definition of an adverse event, should not be reported as adverse events. A Grade 3 or 4 event (severe) as per CTCAE v4.03 does not automatically indicate a SAE unless it meets the definition of serious as defined below and/or as per investigator's discretion. A dose hold or medication for the lab abnormality may be required by the protocol in which case the lab abnormality would still, by definition, be an adverse event and must be reported as such.

8.1.3 Adverse events of special interest

Adverse events of special interest (AESI) are defined as events (serious or non-serious) which are ones of scientific and medical concern specific to the sponsor's product or program, for

which ongoing monitoring and rapid communication by the investigator to the sponsor may be appropriate. Such events may require further investigation in order to characterize and understand them.

Adverse events of special interest are defined on the basis of an ongoing review of the safety data. AESIs are discussed in detail in the Investigator Brochure, and a list of Medical Dictionary for Regulatory Activities (MedDRA) preferred terms to flag as AESI's will be included in the SAP.

AESI for dabrafenib include:

- Hypersensitivity
- Pyrexia
- cuSCC including keratoacanthoma
- Non-cutaneous treatment emergent malignancies
- New primary melanoma
- pre-renal and intrinsic renal failure
- Uveitis
- Hyperglycemia
- Pancreatitis
- Neutropenia (in combination with trametinib)

AESI for trametinib include:

- Skin related toxicities
- Ocular events
- Cardiac related events
- Hepatic disorders
- Pneumonitis/interstitial lung disease
- Bleeding events
- Diarrhea
- Hypertension
- Edema
- Hypersensitivity
- Deep vein thrombosis/pulmonary embolism

AESI for PDR001 include:

- Endocrinopathies
- Pneumonitis
- Colitis
- Hepatitis
- Nephritis
- Encephalitis
- Rash

- Infusion reaction
- Other immune disorders

8.2 Serious adverse events

8.2.1 Definitions

Serious adverse event (SAE) is defined as one of the following:

- Is fatal or life-threatening
- Results in persistent or significant disability/incapacity
- Constitutes a congenital anomaly/birth defect
- Is medically significant, i.e., defined as an event that jeopardizes the subject or may require medical or surgical intervention to prevent one of the outcomes listed above
- Requires inpatient hospitalization or prolongation of existing hospitalization,
- Note that hospitalizations for the following reasons should not be reported as serious adverse events:
    - Routine treatment or monitoring of the studied indication, not associated with any deterioration in condition
    - Elective or pre-planned treatment for a pre-existing condition that is unrelated to the indication under study and has not worsened since signing the informed consent
    - Social reasons and respite care in the absence of any deterioration in the subject's general condition
- Note that treatment on an emergency outpatient basis that does not result in hospital admission and involves an event not fulfilling any of the definitions of a SAE given above is not a serious adverse event

8.2.2 Reporting

For subjects with known *BRAF* V600 mutation status who sign the main study ICF, SAE collection starts at time of main study informed consent whether the subject is a screen failure or not.

To ensure subject safety, every SAE, regardless of suspected causality, occurring after the subject has provided informed consent and until at least 30 days after the subject has stopped study treatment must be reported to Novartis within 24 hours of learning of its occurrence and until

- at least 150 days following the last dose of PDR001/placebo (or all patients in a double blind study)
- 30 days after the last dose of dabrafenib or 120 days after the last dose of trametinib, if the **subject** continued the combination partner more than 150 days after the last dose of PDR001) OR
- the start of a new post-treatment antineoplastic medication if sooner that the 150 days mentioned above. If a subject starts a post-treatment antineoplastic therapy, then only SAEs suspected to be related to study treatment should be collected out to 150 days after

discontinuation of PDR001/placebo. SAEs suspected to be related to PDR001/placebo will continue to be collected beyond the 150-Day safety visit.

Any additional information for the SAE including complications, progression of the initial SAE, and recurrent episodes must be reported as follow-up to the original episode within 24 hours of the investigator receiving the follow-up information. An SAE occurring at a different time interval or otherwise considered completely unrelated to a previously reported one should be reported separately as a new event.

Any SAEs experienced after the reporting period described above should only be reported to Novartis if the investigator suspects a causal relationship to the study treatment.

Information about all SAEs is collected and recorded on the Serious Adverse Event Report Form; all applicable sections of the form must be completed in order to provide a clinically thorough report. The investigator must assess and record the relationship of each SAE to each specific study treatment (if there is more than one study treatment), complete the SAE Report Form in English, and submit the completed form within 24 hours to Novartis. Detailed instructions regarding the SAE submission process and requirements for signatures are to be found in the investigator folder provided to each site

Follow-up information is submitted in the same way as the original SAE Report. Each re-occurrence, complication, or progression of the original event should be reported as a follow-up to that event regardless of when it occurs. The follow-up information should describe whether the event has resolved or continues, if and how it was treated, whether the blind was broken or not, and whether the subject continued or withdrew from study participation.

If the SAE is not previously documented in the Investigator's Brochure or Package Insert (new occurrence) and is thought to be related to the Novartis study treatment, an oncology Novartis Chief Medical Office and Patient Safety (CMO&PS) department associate may urgently require further information from the investigator for Health Authority reporting. If the SAE is confirmed as Suspected Unexpected Serious Adverse Reaction (SUSAR) according to the Reference Safety Information in the IB, drug safety will be unblinded to the treatment according to the Novartis SOP for unblinding. Novartis may need to issue an Investigator Notification (IN), to inform all investigators involved in any study with the same drug that this SAE has been reported. In this case the subject will be unblinded to the treatment. SUSARs will be collected and reported to the competent authorities and relevant ethics committees in accordance with Directive 2001/20/EC or as per national regulatory requirements in participating countries.

8.3 Emergency unblinding of treatment assignment

Parts 1 & 2: Safety run-in and biomarker cohort

Since these parts are open-label, there is no need for treatment unblinding instructions.

Part 3: Double-blind, randomized, placebo-controlled part

Emergency unblinding decisions must only be undertaken by the investigator when it is essential for effective treatment of the subject. Most often, study treatment discontinuation and knowledge of the possible treatment assignments are sufficient to treat a study subject who presents with an emergency condition. Emergency code breaks are performed using the IRT.

When the investigator/designee contacts the IRT to unblind a subject, he/she must provide the requested subject identifying information and confirm the necessity to unblind the subject. The investigator/designee will then receive details of the drug treatment for the specified subject and a fax confirming this information. The system will automatically inform the Novartis monitor for the site and the Study Lead that the code has been broken.

It is the investigator's responsibility to ensure that there is a procedure in place to allow access to the IRT in case of emergency. The investigator will inform the subject how to contact his/her backup in cases of emergency when he/she is unavailable. The protocol number, study treatment name if available, subject number, and instructions for contacting the local Novartis CPO (or any entity to which it has delegated responsibility for emergency code breaks) will be provided to the subject in case emergency unblinding is required at a time when the investigator and backup are unavailable. However, if a mechanism is already in place to ensure that the investigator and/or back-up can always be reached in case of emergency then the procedure above is not required.

Except for medical emergencies, for regulatory reporting purposes, or if it is critical to determine subsequent therapy after disease progression, documented approval by the Novartis study physician is required prior to unblinding a subject's treatment assignment.

Study treatment must be discontinued once emergency unblinding has occurred.

If a subject is unblinded, he/she must be discontinued from the study treatment and must be followed for efficacy and survival, as applicable per Section 7.1.7 and Section 7.1.8.

8.4 Pregnancies

To ensure subject safety, each pregnancy occurring while the subject is on study treatment must be reported to Novartis within 24 hours of learning of its occurrence. The pregnancy should be followed up to determine outcome, including spontaneous or voluntary termination, details of the birth, and the presence or absence of any birth defects, congenital abnormalities, or maternal and/or newborn complications.

Pregnancy should be recorded on a Clinical Trial Pregnancy Form and reported by the investigator to the oncology Novartis CMO&PS. Pregnancy follow-up should be recorded on the same form and should include an assessment of the possible relationship to the study treatment any pregnancy outcome. Any SAE experienced during pregnancy must be reported on the SAE Report Form.

Pregnancy outcomes must be collected for the female partners of any males who took study treatment in this study. Consent to report information regarding these pregnancy outcomes should be obtained from the mother.

8.5 Warnings and precautions

No evidence available at the time of the approval of this study protocol indicated that special warnings or precautions were appropriate, other than those noted in the provided Investigator Brochure. Additional safety information collected between IB updates will be communicated in the form of Investigator Notifications. This information will be included in the subject informed consent and should be discussed with the subject during the study as needed.

8.6 Data Monitoring Committee

This study will institute a data monitoring committee (DMC) which will function independently of all other individuals associated with the conduct of this clinical trial, including the site investigators participating in the study. The DMC will be constituted prior to the randomization of the first subject. The DMC will be responsible to review safety data approximately every 3-6 months (after the first randomized subject has started study treatment). This includes but does not limit the role of the DMC to evaluate these data and to provide recommendations to the sponsor to continue modify or stop the study early. The DMC will be informed and convened quickly in the event of unexpected results that raise concerns to permit DMC evaluation and input.

It is expected that the DMC will consist at a minimum of two physicians with appropriate disease area qualifications and one statistician. There will be a meeting with the DMC describing their roles and responsibilities and discussing potential data format and process issues prior to the finalization of DMC charter and the interim SAP.

It is envisioned that the DMC may make the following types of recommendations, namely:

- No safety issues, ethical to continue the study as planned
- Serious safety concerns precluding further study treatment, regardless of efficacy
- Recommendation to continue the study but proposing an amendment to the protocol (e.g., incorporate an additional safety assessments)

Furthermore, the DMC will be responsible for reviewing the efficacy results from the interim PFS analysis (at which point the first interim OS data will be analyzed as well), as well overseeing the safety data accruing in the trial at regular intervals. The interim PFS analysis will be performed by an independent external statistician. The results will be made available to the DMC who will then make a recommendation to the Sponsor. Details will be provided in the DMC charter. If the unblinded results are not made available at this point, the study will proceed as normal and the final PFS analysis will be performed by the Sponsor's clinical team when the approximate target number of events has been observed.

8.7 Steering Committee

The steering committee will be established comprising investigators participating in the trial, i.e. not being members of the DMC and Novartis representatives from the Clinical Trial Team.

The SC will ensure transparent management of the study according to the protocol through recommending and approving modifications as circumstances require. The SC will review protocol amendments as appropriate. Together with the clinical trial team, the SC will also develop recommendations for publications of study results including authorship rules. The details of the role of the Steering Committee will be defined in a Steering Committee charter.

9 Data collection and management

9.1 Data confidentiality

Information about study subjects will be kept confidential and managed under the applicable laws and regulations. Those regulations require a signed subject authorization informing the subject of the following:

- What protected health information (PHI) will be collected from subjects in this study
- Who will have access to that information and why
- Who will use or disclose that information
- The rights of a research subject to revoke their authorization for use of their PHI.

In the event that a subject revokes authorization to collect or use PHI, the investigator, by regulation, retains the ability to use all information collected prior to the revocation of subject authorization. For subjects that have revoked authorization to collect or use PHI, attempts should be made to obtain permission to collect follow-up safety information (e.g., has the subject experienced any new or worsened AEs) at the end of their scheduled study period.

The data collection system for this study uses built-in security features to encrypt all data for transmission in both directions, preventing unauthorized access to confidential participant information. Access to the system will be controlled by a sequence of individually assigned user identification codes and passwords, made available only to authorized personnel who have completed prerequisite training.

Prior to entering key sensitive personally identifiable information (Subject Initials and exact Date of Birth), the system will prompt site to verify that this data is allowed to be collected. If the site indicates that country rules or ethics committee standards do not permit collection of these items, the system will not solicit Subject Initials. Year of birth will be solicited (in the place of exact date of birth) to establish that the subject satisfies protocol age requirements and to enable appropriate age-related normal ranges to be used in assessing laboratory test results.

9.2 Site monitoring

Before study initiation, at a site initiation visit or at an investigator's meeting, Novartis personnel (or designated CRO) will review the protocol and CRFs with the investigators and their staff. During the study, the field monitor will visit the site regularly to check the completeness of subject records, the accuracy of entries on the CRFs, the adherence to the protocol to Good Clinical Practice, the progress of enrollment, and to ensure that study treatment is being stored, dispensed, and accounted for according to specifications. Key study personnel must be available to assist the field monitor during these visits.

The investigator must maintain source documents for each subject in the study, consisting of case and visit notes (hospital or clinic medical records) containing demographic and medical information, laboratory data, electrocardiograms, and the results of any other tests or assessments. All information recorded on CRFs must be traceable to source documents in the subject's file. The investigator must also keep the original signed informed consent form (a signed copy is given to the subject).

The investigator must give the monitor access to all relevant source documents to confirm their consistency with the CRF entries. Novartis monitoring standards require full verification for the presence of informed consent, adherence to the inclusion/exclusion criteria and documentation of SAEs. Additional checks of the consistency of the source data with the CRFs are performed according to the study-specific monitoring plan.

9.3 Data collection

For studies using Electronic Data Capture (EDC), the designated investigator staff will enter the data required by the protocol into the Electronic Case Report Forms (eCRF). The eCRFs have been built using fully validated secure web-enabled software that conforms to 21 CFR Part 11 requirements, Investigator site staff will not be given access to the EDC system until they have been trained. Automatic validation programs check for data discrepancies in the eCRFs and, allow modification or verification of the entered data by the investigator staff.

The Principal Investigator is responsible for assuring that the data entered into eCRF is complete, accurate, and that entry and updates are performed in a timely manner.

Designated investigational site staff will enter the information required by the protocol into the appropriate eCRF and/or designated laboratory requisition forms. Field monitors will review the eCRFs and laboratory paper requisition forms for accuracy and completeness and instruct site personnel to make any required corrections or additions. One copy of the requisition form will be forwarded to each analytical laboratory with the respective sample(s) by the field monitor or by the designated investigational site staff; and one copy will be retained at the investigational site.

PK and biomarker (blood, serum, plasma and/or tissue) samples obtained during the course of the study will be collected and shipped by the site to a Novartis designated laboratory for sample management and/or analysis. The Laboratory results will be sent electronically to Novartis.

ECG data will be collected via 12-lead digital ECG machines and reviewed by the site investigator. ECG data should be entered in to the eCRFs.

Radiological imaging and photography data will be acquired by the sites and interpreted locally. Additionally, radiological and photography data will be transmitted by the sites to a CRO designated by Novartis to undergo quality checks and central review. The decision regarding subject management will remain with the local investigator.

Data entered into IRT will be transferred electronically to Novartis as described in the Data Transfer Specifications for designated IRT vendor.

9.4 Database management and quality control

For studies using eCRFs, Novartis personnel (or designated CRO) will review the data entered by investigational staff for completeness and accuracy. Electronic data queries stating the nature of the problem and requesting clarification will be created for discrepancies and missing values and sent to the investigational site via the EDC system. Designated investigator site staff are required to respond promptly to queries and to make any necessary changes to the data.

Concomitant treatments and prior medications entered into the database will be coded using the WHO Drug Reference List, which employs the Anatomical Therapeutic Chemical classification

system. Medical history/current medical conditions and adverse events will be coded using the MedDRA terminology.

Samples and/or data will be processed centrally and the results will be sent electronically to Novartis (or a designated CRO). Data that will be processed centrally include:

- IRT data including information regarding screening, drug assignment and discontinuation
- Centrally analyzed laboratory data including clinical, PK, tumor biomarker and safety parameters
- Central imaging review (if required)

Randomization codes and data about all study treatments dispensed to the subject and all IRT assigned dosage changes will be tracked using an Interactive Response Technology. The system will be supplied by a vendor(s), who will also manage the database. The data will be sent electronically to Novartis personnel (or designated CRO).

At the conclusion of the study, the occurrence of any emergency code breaks will be determined after return of all code break reports and unused drug supplies to Novartis personnel (or designated CRO). The occurrence of any protocol violations will be determined. After these actions have been completed and the data has been verified to be complete and accurate, the database will be declared locked and the treatment codes will be unblinded and made available for data analysis. Authorization is required prior to making any database changes to locked data, by joint written agreement between the Global Head of Biostatistics and Data Management and the Global Head of Clinical Development.

10 Statistical methods and data analysis

For the safety run-in (part 1) and biomarker cohort (part 2), efficacy and safety analyses will be conducted on all subject data at the time that each part is completed. For the randomized part of the study (part 3), the final PFS efficacy and safety analysis will be performed after observing approximately 352 PFS events.

As the rate of events may decrease with longer follow-up, achieving the target number of events for the final PFS analysis might be challenging to obtain within a reasonable period of follow-up. To allow for this scenario the data cutoff date for the final PFS analysis will be chosen at the time when the target number of approximately 352 PFS events has been observed or at approximately 24 months after the last patient has been randomized, whichever occurs first.

In addition, an interim PFS analysis for the primary endpoint will be performed after approximately 260 events have been reported. The primary CSR will be produced after the final PFS analysis (or after the interim PFS analysis if the test of the PFS endpoint is significant and Novartis decides to make the unblinded results publicly available) and will summarize data from all 3 parts of the study.

The additional data for any patients continuing to receive study treatment past this time, as allowed by the protocol, will be further summarized in a final study report once these patients complete the study.

Categorical data will be presented as contingency tables (frequencies and percentages). For continuous data summary statistics of mean, standard deviation, median, minimum, and

maximum will be presented. For selected parameters, 25th and 75th percentiles will also be presented.

Screen failure subjects are those who signed the informed consent, but never started the study treatment for any reason. For these subjects, the eCRF data collected will not be included in analyses, but will be reported in CSR as separate listings.

10.1 Analysis sets

10.1.1 Full Analysis Set

Parts 1 & 2: Safety run-in and biomarker cohort

For each part, the full analysis set (FAS) and safety set are defined in the same way and comprise all subjects to whom study treatment has been assigned and who received at least one dose of any study treatment (i.e. at least one dose of any component of PDR001 (including incomplete infusion), dabrafenib, or trametinib). Subjects will be analyzed according to the study treatment they have been assigned to.

Part 3: Double-blind, randomized, placebo-controlled part

The FAS comprises all subjects to whom study treatment has been assigned by randomization, regardless of whether or not treatment was administered. According to the intent to treat principle, subjects will be analyzed according to the treatment and strata they have been assigned to during the randomization procedure. This population will be the primary population for efficacy analyses.

10.1.2 Safety set

Parts 1 & 2: Safety run-in and biomarker cohort

See definition of FAS.

Part 3: Double-blind, randomized, placebo-controlled part

The Safety Set includes all subjects who received at least one dose of study treatment (i.e. at least one dose of any component of PDR001 or placebo (including incomplete infusion), dabrafenib, or trametinib). Subjects will be analyzed according to the study treatment received, where treatment received is defined as the randomized treatment if the subject took at least one dose of that treatment or the first treatment received if the randomized treatment was never received.

10.1.3 Per-Protocol set

Parts 1 & 2: Safety run-in and biomarker cohort

Not applicable.

Part 3: Double-blind, randomized, placebo-controlled part

The Per-Protocol Set (PPS) will only be used for sensitivity analyses on the primary variables. The PPS consists of a subset of the subjects in the FAS who are compliant with requirements of the clinical study protocol (CSP).

Oncology standards for protocol deviations **potentially** leading to exclusion from the PPS are:

- type of indication different from those required by the CSP (e.g., incorrect histology/cytology, not refractory, not metastatic, different grade of cancer, etc.)
- if prior therapy does not match with CSP requirements in terms of number and types of previous therapy regimens
- missing or incomplete documentation of stage of disease (as required in the CSP)
- if ECOG performance status at least 2 categories worse than protocol-defined inclusion criteria
- another anti-neoplastic therapy administered after start of study treatment and prior to first tumor assessment
- study treatment received different from treatment assigned by randomization

Full specifications of deviations leading to exclusion will be described in the Study Specific Document (SSD) document.

10.1.4 Dose-determining analysis set

Part 1: Safety run-in

The Dose-Determining Set (DDS) includes all subjects who received at least one dose of study treatment who either 1) met the minimum exposure criterion and had sufficient safety evaluations, or 2) experienced a DLT during the first 8 weeks (56 days) of PDR001 in combination with dabrafenib and trametinib dosing.

A subject meets the minimum exposure criterion if the subject receives at least 1 dose of PDR001, and takes at least 50% of the planned cumulative doses of dabrafenib and trametinib within the first 8 weeks (56 days) of treatment. Subjects who do not experience a DLT during the first 8 weeks are considered to have sufficient safety evaluations if they have been observed for at least 8 weeks following the first dose of PDR001 in combination with dabrafenib and trametinib, and are considered by both the Sponsor and Investigators to have enough safety data to conclude that a DLT did not occur. Patients who do not meet the minimum exposure criterion due to an AE will be counted as having a DLT. Patients who do not meet the minimum exposure criterion for reasons other than AE’s (e.g., rapid disease progression or non-compliance) will not be included in the DDS. **Parts 2 & 3: Biomarker cohort and double-blind, randomized, placebo-controlled part**

Not applicable.

10.1.5 Pharmacokinetic analysis set

The following analysis sets will be derived separately for each of the 3 parts. The PDR001 pharmacokinetic analysis set (PAS-PDR001) includes all subjects who provide at least one evaluable PDR001 PK concentration. For a concentration to be evaluable, subjects are required to:

- Received one of the planned treatments of PDR001 prior to sampling
- For pre-dose samples, have the samples collected before the next dose administration
- For end-of-infusion samples, have the samples collected within 2 hours post infusion

The dabrafenib and trametinib Pharmacokinetic analysis set (PAS-D+T) includes all subjects who provide at least one evaluable dabrafenib or trametinib PK concentration. For a concentration to be evaluable, subjects are required to:

- Receive a dose of dabrafenib and trametinib prior to sampling
- For pre-dose samples, have the samples collected before the next dose administration
- For post-dose samples, do not vomit within 4 hours after the dosing of dabrafenib and trametinib

10.1.6 Other analysis sets

The *Immunogenicity prevalence set* includes all subjects in the FAS with a determinant baseline IG sample **or** at least one determinant post-baseline IG sample.

The *Immunogenicity incidence set* includes all subjects in the Immunogenicity prevalence set with a determinant baseline IG sample **and** at least one determinant post-baseline IG sample.

Additional details, including definition of determinant, will be provided in the SAP.

10.1.6.1 Efficacy/evaluable set

Not applicable.

10.2 Subject demographics/other baseline characteristics

Parts 1 & 2: Safety run-in and biomarker cohort

Demographic and other baseline data including disease characteristics will be listed and summarized descriptively by dose cohort for the FAS. Relevant medical histories and current medical condition at baseline will be summarized separately by system organ class and preferred term, by dose cohort.

Part 3: Double-blind, randomized, placebo-controlled part

Demographic and other baseline data including disease characteristics will be listed and summarized descriptively by treatment group and for all subjects for the FAS and safety set. Relevant medical histories and current medical at baseline will be summarized separately by system organ class and preferred term, by treatment group and for all subjects.

10.3 Treatments (study treatment, concomitant therapies, compliance)

The safety set will be used to summarize treatment data. Categorical data will be summarized as frequencies and percentages. For continuous data, mean, standard deviation, median, 25th and 75th percentiles, minimum, and maximum will be presented.

Parts 1 & 2: Safety run-in and biomarker cohort

The duration of exposure to PDR001, dabrafenib, and trametinib as well as the dose intensity (computed as the ratio of actual cumulative dose received and actual duration of exposure) and the relative dose intensity (computed as the ratio of dose intensity and planned dose intensity)

will be summarized by means of descriptive statistics using the safety set. The duration of exposure will also be presented for the study treatment by arm.

The number of subjects with dose adjustments (reductions, interruption, or permanent discontinuation) and the reasons will be summarized by dose cohort, and all dosing data will be listed.

Concomitant medications and significant non-drug therapies prior to and after the start of the study treatment will be listed and summarized according to the Anatomical Therapeutic Chemical (ATC) classification system, by dose cohort.

Part 3: Double-blind, randomized, placebo-controlled part

The duration of exposure to PDR001 (or matching placebo), dabrafenib, and trametinib as well as the dose intensity (computed as the ratio of actual cumulative dose received and actual duration of exposure) and the relative dose intensity (computed as the ratio of dose intensity and planned dose intensity) will be summarized by means of descriptive statistics using the safety set. The duration of exposure will also be presented for the study treatment by arm.

The number of subjects with dose adjustments (reductions, interruption, or permanent discontinuation) and the reasons will be summarized by treatment group and for all subjects, and all dosing data will be listed.

Concomitant medications and significant non-drug therapies prior to and after the start of the study treatment will be listed and summarized according to the ATC classification system, by treatment group and for all subjects.

10.4 Primary objective

Part 1: Safety run-in

The primary objective is to determine the recommended regimen of PDR001 in combination with dabrafenib and trametinib for the randomized part (part 3)

Part 2: Biomarker cohort

The primary objective is to evaluate changes in PD-L1 levels and CD8+ cells upon treatment with PDR001 in combination with dabrafenib and trametinib.

Part 3: Double-blind, randomized, placebo-controlled part

The primary objective is to evaluate and compare PFS per RECIST 1.1 of PDR001 in combination with dabrafenib and trametinib versus dabrafenib and trametinib plus placebo.

10.4.1 Variables

Part 1: Safety run-in

The primary variable is the incidence of DLTs during the first 8 weeks (56 days) of treatment for each dose level associated with administration of PDR001 in combination of dabrafenib and trametinib.

Part 2: Biomarker cohort

The primary variable is PD-L1 levels and CD8+ cells and changes from baseline.

Part 3: Double-blind, randomized, placebo-controlled part

The primary efficacy variable is PFS, defined as the time from the date of randomization to the date of the first documented progression per RECIST 1.1 or death due to any cause. PFS will be assessed via local review according to RECIST 1.1 (see Appendix 1 for further details). Censoring conventions are provided below in Section 10.4.3.

Local scans will be sent to a blinded, independent central reviewer who will provide assessments using both RECIST 1.1 and tumor response criteria based on guidelines for immunotherapy, and these centrally reviewed data will be used for sensitivity analysis.

10.4.2 Statistical hypothesis, model, and method of analysis

Part 1: Safety run-in

Identification of recommended regimen

Estimation of the recommended regimen will be based upon the estimation of the probability of DLT in the first 8 weeks (56 days) for subjects in the DDS. A lower recommended regimen may be identified based on other safety and PK data from the current study (Section 6.2.3).

Bayesian adaptive approach

The dose determination part of this study will be guided by a Bayesian analysis of DLT data for the first 8 weeks (56 days) that subjects receive the combination of PDR001, dabrafenib, and trametinib. The Bayesian analysis to assess the triple combination will be based on a separate 10-parameter model for each dose regimen that comprises single-agent toxicity parts and interaction parts to describe both two-way and three-way drug safety interactions. Single agent toxicity is modelled using logistic regression for the probability of a subject experiencing a DLT against log-dose. The odds of a DLT for each dose regimen are then calculated under no interaction for the three single agent toxicities, and interaction is accounted for by adjusting these odds with additional model parameters (odds multipliers). Details of the model are given in Appendix 2.

Assessment of subject risk

After each cohort of subjects, the posterior distribution for the risk of DLT for new subjects at combination doses of interest will be evaluated. The posterior distributions will be summarized to provide the posterior probability that the risk of DLT for each dose regimen lies within the following intervals:

- Under-dosing: [0, 16%)
- Targeted toxicity: [16%, 33%)
- Excessive toxicity: [33%, 100%]

The escalation with overdose control (EWOC) principle

Dosing regimen decisions are guided by the escalation with overdose control principle (Rogatko 2007). A dosing regimen may only be used for newly enrolled subjects if the risk of excessive toxicity at that dosing regimen is less than 25%.

Prior distributions

A meta-analytic-predictive (MAP) approach was used to derive the prior distribution for the single-agent PDR001, dabrafenib, and trametinib model parameters. The MAP prior for the logistic model parameters for this study is the conditional distribution of the parameters given the historical data (Spiegelhalter 2004, Neuenschwander 2010, Neuenschwander 2014). MAP priors are derived from hierarchical models, which take into account possible differences between the studies. For dabrafenib and trametinib, 100% MAP priors will be used. For PDR001, this is then robustified by creating a mixture prior including both a component derived from the MAP prior and a weakly informative robustification component. This robustification allows for the possibility that the dose/toxicity relationship for PDR001 in combination differs substantially from that of the single agent. A full description of the application of the MAP approach to derive the prior distributions of the single agent PDR001, dabrafenib, and trametinib model parameters for each dose regimen is given in Appendix 2.

The prior distributions for the interaction parameters were based upon prior understanding of possible drug safety interactions. These priors allow for the possibility of either synergistic or antagonistic interaction, and are fully described in Appendix 2.

Additional DLT information from completed studies assessing the combination of dabrafenib and trametinib (i.e. Phase I study BRF113220, Phase III study MEK115306 (COMBI-d), and Phase III study MEK116513 (COMBI-v)) are included using discounted weighting. Full details of the discounted weighting approach are described in Appendix 2.

Starting dose

The starting dosing regimen is 400 mg i.v. Q4W PDR001, 150 mg BID dabrafenib, and 2 mg QD trametinib (Section 6.2.1). For this dosing regimen, the prior risk of excessive toxicity is 12%, which satisfies the EWOC criterion. A full assessment of the prior risk to subjects for all dosing regimens is given in Appendix 2. Historical data were reviewed to account for modified DLT criteria (protocol amendment 2). Prior specifications for the BLRM were not impacted, and therefore the starting dose of Part 1 remained unchanged.

Listing of DLT's

DLTs will be listed, and their incidence summarized by primary system organ class and preferred term and worst grade (CTCAE v4.03). Listings and summaries will be based on the DDS.

Part 2: Biomarker cohort

Descriptive statistics of the primary endpoint, PD-L1 levels and CD8+ cell values and changes from baseline, will be summarized by visit.

Part 3: Double-blind, randomized, placebo-controlled part

The following statistical hypotheses will be tested to address the primary efficacy objective for PFS (based on investigator assessment of RECIST 1.1 criteria):

$H_{01} : \theta_1 \geq 1$ vs. $H_{A1} : \theta_1 < 1$

where θ_1 is the PFS hazard ratio (PDR001 combined with dabrafenib and trametinib versus dabrafenib and trametinib plus placebo). The primary efficacy analysis to test these hypotheses

and compare the two treatment groups will consist of a stratified log-rank test at an overall one-sided 2.5% level of significance. The stratification will be based on the randomization stratification factors, i.e., (LDH level: < 1 x ULN vs ≥1 to < 2 x ULN vs ≥ 2 x ULN; ECOG PS: 0 vs 1 vs 2).

Analyses will be based on the FAS population according to the randomized treatment group and strata assigned at randomization. The PFS distribution will be estimated using the Kaplan-Meier method, and Kaplan-Meier curves, quartiles and associated 95% confidence intervals will be presented for each treatment group. The hazard ratio for PFS will be calculated, along with its 95% confidence interval, from a stratified Cox model using the same stratification factors as for the log-rank test.

The PFS analysis will be performed as part of a two-look group sequential design using a conservative Gamma alpha spending function with Gamma parameter = -9.7 (Hwang, Shih and DeCani, 1990). Analyses will be based on the FAS population according to the randomized treatment group and strata assigned at randomization. The PFS distribution will be estimated using the Kaplan-Meier method, and Kaplan-Meier curves, quartiles and associated 95% confidence intervals will be presented for each treatment group. The hazard ratio for PFS will be calculated, along with its 95% confidence interval, from a stratified Cox model using the same stratification factors as for the log-rank test.

If the interim PFS analysis is significant, the subsequent planned final PFS analysis will still be performed to obtain more mature PFS data and additional follow-up PFS analyses may also be performed earlier than planned final PFS analysis at the request of health authorities or to facilitate health authority interactions.

The data cutoff date for the final PFS analysis will occur at the time when the target number of approximately 352 PFS events has been observed or at approximately 24 months after the last patient has been randomized, whichever occurs first.

10.4.3 Handling of missing values/censoring/discontinuations

Part 1: Safety run-in

Subjects who are ineligible for the DDS will be excluded from the primary analysis (incidence of DLT during first 8 weeks of PDR001 in combination with dabrafenib and trametinib), although their data will be used for all remaining analyses.

Other missing data will simply be noted as missing on appropriate tables/listings.

Part 2: Biomarker cohort

Missing data will simply be noted as missing on appropriate tables/listings.

Part 3: Double-blind, randomized, placebo-controlled part

In the primary analysis, PFS will be censored at the date of the last adequate tumor assessment if no PFS event is observed prior to the analysis cut-off date.

PFS events documented after the initiation of new anti-neoplastic therapy (i.e. RECIST 1.1 documented disease progression or death) will be considered for the primary analysis provided tumor assessments continue after initiation of new cancer therapy.

If a PFS event is observed after two or more missing or non-adequate tumor assessments, then PFS will be censored at the last adequate tumor assessment before the PFS event. If a PFS event is observed after a single missing or non-adequate tumor assessment, the actual date of event will be used (RECIST 1.1 in Appendix 1).

10.4.4 Supportive and sensitivity analyses

Parts 1 & 2: Safety run-in and biomarker cohort

Not applicable.

Part 3: Double-blind, randomized, placebo-controlled part

As a supportive analysis, PFS based on RECIST 1.1 criteria as per blinded independent central review will be analyzed using a stratified Cox model, with the same analysis conventions as the primary efficacy analysis (based on investigator assessment), and the treatment effect will be summarized by the hazard ratio with its 95% confidence interval. Kaplan-Meier curves, quartiles and associated 95% confidence intervals will be presented for each treatment group.

Similarly, the above analysis will be performed for PFS based on tumor response criteria based on guidelines for immunotherapy as per blinded independent central review.

As sensitivity analyses for PFS and OS in the FAS, the hazard ratio and 95% confidence interval will be obtained from:

- An unstratified and covariate unadjusted Cox model
- A stratified and covariate adjusted Cox model that may include the following covariates: gender, age group (< 65, ≥ 65 years), LDH group (< 1 x ULN, ≥1 to < 2 x ULN, ≥ 2 x ULN), ECOG PS (0, 1, 2), sites of disease at baseline (< 3, ≥ 3), stage at screening (IIIC or IVM1a vs IVM1b or IVM1c), BRAF mutation (V600E, V600K), and prior adjuvant checkpoint inhibitor therapy (yes, no). A final list of covariates of interest will be defined in the SAP.
- PFS and OS will also be analyzed based on the PPS, using the same analysis conventions as in the primary efficacy analysis (with the exception of the log-rank test, which will not be performed).

If the primary PFS analysis is statistically significant, subgroup analyses to assess the homogeneity of the treatment effect across demographic and baseline disease characteristics will be performed. The following subgroups may be considered: gender (male, female), age group (< 65, ≥ 65 years), LDH group (< 1 x ULN, ≥1 to < 2 x ULN, ≥ 2 x ULN), ECOG PS (0, 1, 2), sites of disease at baseline (<3, ≥ 3), stage at screening (IIIC or IVM1a vs IVM1b or IVM1c), BRAF mutation (V600E, V600K), and prior adjuvant checkpoint inhibitor therapy (yes, no). An additional subgroup will be derived for those who experienced at least 1 immune-related AE (yes, no). A final list of subgroups of interest will be defined in the SAP.

The number of subjects censored for PFS and OS and reasons for censoring will be summarized by treatment group using descriptive statistics.

Additional key supportive analyses for PFS and OS will be performed to take into account delayed treatment effect leading to non-proportional hazards. Due to the expected non-

proportional hazards the treatment effect will be estimated by means of a restricted mean survival time (RMST) approach (Uno et al., 2014).

The RMST and 95% CIs will be estimated for each treatment group separately and the treatment effect will be derived as the difference in the treatment group RMSTs with the 95% CI for the difference also being provided. Additional supportive analyses will include methods that provide alternative tests and treatment effect estimates in the presence of non-proportional hazards, e.g. weighted log-rank test with Fleming-Harrington class of weight (Fleming & Harrington, 1991), landmark analyses, piece-wise Cox regression to obtain HR by period. Details of all these analyses will be outlined in the SAP.

Subjects randomized to receive dabrafenib, trametinib, and placebo will only be permitted to crossover to dabrafenib, trametinib, and PDR001 in the case that statistically significant interim or final OS results are demonstrated. The SAP will pre-specify analyses that will be conducted using crossover data, if applicable.

Additional supportive or exploratory analyses may be conducted to support the primary objective of the safety run-in, biomarker cohort, or randomized part, if appropriate, and the details of these analyses will be defined in the SAP.

10.5 Secondary objectives

Part 1: Safety run- in and Part 2: Biomarker cohort

The secondary objectives for these parts of the study are to determine the safety and tolerability, evaluate preliminary anti-tumor activity, characterize the pharmacokinetics, and evaluate the prevalence and incidence of immunogenicity of PDR001 in combination with dabrafenib and trametinib.

Part 3: Double-blind, randomized, placebo-controlled part

The secondary objectives in this part of the study are to compare the two treatment groups with respect to OS, tumor related efficacy variables (ORR, DOR, and DCR based on RECIST 1.1), safety and tolerability, quality of life, pharmacokinetics, and immunogenicity.

OS is identified as the key secondary endpoint. A hierarchical testing strategy will be used to control the overall type I error rate, where OS will only be formally tested and interpreted if the primary analysis of PFS is statistically significant.

10.5.1 Key secondary objectives

Part 1: Safety run-in and Part 2: Biomarker cohort

Not applicable.

Part 3: Double-blind, randomized, placebo-controlled part

The key secondary objective is to determine whether treatment with PDR001 in combination with dabrafenib and trametinib prolongs OS compared with dabrafenib and trametinib plus placebo. OS is defined as the time from date of randomization to date of death due to any cause. If a patient is not known to have died, then OS will be censored at the latest date the patient was known to be alive (on or before the cut-off date).

The following statistical hypotheses will be tested to address the key secondary efficacy objective for OS:

$H_{02} : \theta_2 \geq 1$ vs. $H_{12} : \theta_2 < 1$

where θ_2 is the OS hazard ratio (PDR001 combined with dabrafenib and trametinib versus dabrafenib and trametinib plus placebo). The analysis to test these hypotheses and compare the two treatment groups will consist of a stratified log-rank test at an overall one-sided 2.5% level of significance. The stratification will be based on the randomization stratification factors, i.e., (LDH level: < 1 x ULN vs ≥ 1 to < 2 x ULN vs ≥ 2 x ULN; ECOG PS: 0 vs 1 vs 2).

OS analyses will be conducted as a part of a three-look group sequential design using a Lan-DeMets (O'Brien-Fleming) alpha spending function. Analyses will be based on the FAS population according to the randomized treatment group and strata assigned at randomization. The OS distribution will be estimated using the Kaplan-Meier method, and Kaplan-Meier curves, quartiles and associated 95% confidence intervals will be presented for each treatment group. The hazard ratio for OS will be calculated, along with its 95% confidence interval, using a stratified Cox model.

OS data will be presented at each analysis but will be formally tested hierarchically as follows:

If PFS is statistically significant at the interim PFS analysis:

Interim OS data will be tested at the time of the interim and final PFS analyses.

A final (inferential) analysis for OS is planned when approximately 245 deaths have occurred (or earlier if specifically requested by health authorities).

If PFS is not statistically significant at the interim PFS analysis:

Interim OS data will not be tested at the time of the interim PFS analysis.

If PFS is statistically significant at the final PFS analysis:

Interim OS data will be tested at the time of the final PFS analysis. If OS is not statistically significant at this stage, a final OS analysis will be planned after approximately 245 deaths.

If PFS is not statistically significant at the final PFS analysis:

Interim OS data will not be tested and there will also not be testing of OS data at the planned final OS analysis.

In the case of statistically significant interim or final OS results, additional OS estimates at relevant annual milestones up to 5 years will be summarized.

10.5.2 Other secondary efficacy objectives

Part 1: Safety run-in and Part 2: Biomarker cohort

The secondary objectives in these parts of the study are to determine the safety and tolerability, evaluate preliminary anti-tumor activity, characterize the pharmacokinetics, and evaluate the prevalence and incidence of immunogenicity of PDR001 in combination with dabrafenib and trametinib.

The incidence and severity of adverse events (AEs) and serious adverse events (SAEs), as well as changes in laboratory values, ECOG PS, vital signs, and liver and cardiac parameters will be

summarized. For all safety and tolerability descriptive analyses, the safety set will be used. All listings and tables will be presented by dose cohort (for part 1) and overall (for part 2). Refer to Section 10.5.3 for more details.

To assess preliminary clinical activity, descriptive statistics for PFS, OS, ORR, DCR, and DOR will be summarized by dose cohort (for part 1) and overall (for part 2), using the local review result where applicable. For definitions of these endpoints, refer to Section 10.4, Section 10.5, and the text in Section 10.5.1 for the randomized part of the study. As a supportive analysis, results as per blinded independent central review using RECIST 1.1 and tumor response criteria based on guidelines for immunotherapy will also be presented by dose cohort (for part 1) and overall (for part 2). For details on how censoring will be handled, the same methods described in Section 10.4.3 will be applied.

Refer to Section 10.5.3 for details on the analysis of secondary PK objectives and Section 10.5.4 for details on the analysis of secondary immunogenicity objectives.

Part 3: Double-blind, randomized, placebo-controlled part

Other secondary objectives in this part of the study are to compare the two treatment groups with respect to tumor related efficacy variables (ORR, DOR, and DCR based on RECIST 1.1), safety and tolerability, quality of life, and pharmacokinetics. No multiplicity adjustments are planned for other secondary endpoints.

Overall response rate (ORR) is defined as the proportion of subjects with confirmed best overall response (BOR) of complete response (CR) or partial response (PR), as per local review. ORR will be evaluated according to RECIST 1.1 (Appendix 1). Complete and partial responses must be confirmed by repeat assessments that should be performed not less than 4 weeks after the criteria for response are first met. ORR based on local review will be calculated based on the FAS. ORR and its 95% confidence interval will be presented by treatment group. The Cochran-Mantel-Haenszel chi-square test, stratified by the randomization stratification factors, will be used to compare ORR between the two treatment groups, at the one-sided 2.5% level of significance. As a supportive analysis, ORR as per blinded independent central review (using RECIST 1.1 as well as tumor response criteria based on guidelines for immunotherapy) will be presented by treatment group, along with 95% confidence intervals.

Duration of response (DOR) only applies to subjects whose best overall response is complete response (CR) or partial response (PR) based on tumor response data per local review. DOR will be evaluated according to RECIST 1.1 (Appendix 1). The start date is the date of first documented response of CR or PR (i.e., the start date of response, not the date when response was confirmed), and the end date is defined as the date of the first documented progression or death due to underlying cancer. Subjects continuing without progression or death due to underlying cancer will be censored at the date of their last adequate tumor assessment. DOR based on RECIST 1.1 will be listed and summarized by treatment group for all subjects in the FAS with confirmed BOR of CR or PR, as well as for the subgroup of FAS subjects with confirmed BOR of CR only.

DCR is defined as the proportion of subjects with CR or PR or subjects with stable disease (SD) lasting for a duration of at least 24 weeks as per local review. CR, PR and SD are defined according to RECIST 1.1 (see Appendix 1 for details). DCR will be calculated based on the

FAS. DCR and its 95% confidence interval will be presented by treatment group. As a supportive analysis, DCR as per blinded independent central review (using RECIST 1.1 as well as tumor response criteria based on guidelines for immunotherapy) will be presented by treatment group, along with 95% confidence intervals.

In order to characterize the potential for PD-L1 expression to identify subjects with an enhanced response to PDR001, PFS and OS analyses will be performed by PD-L1 subgroup (positive, negative) where a positive status is defined as having ≥ 1% expression and a negative status is defined as having < 1% expression as determined by immunohistochemistry. Additionally PD-L1 subgroups will also be assessed using ≥ 5% and ≥ 10% as cut-offs for PD-L1 positivity.

Refer to Section 10.5.3 for details on the analysis of secondary safety and tolerability objectives, Section 10.5.4 for secondary PK objectives, Section 10.5.5 for secondary immunogenicity objectives and Section 10.5.8 for secondary quality of life objectives.

10.5.3 Safety objective(s)

10.5.3.1 Analysis set and grouping for the analyses

For all safety analyses, the safety set will be used and data will be presented separately for each part; no pooled safety analyses are planned. For the safety run-in part and biomarker cohort, listings and tables for each part will be presented by dose cohort. For the randomized part, all listings will be presented by treatment group and tables will be presented by treatment group and all subjects.

The overall observation period will be divided into three mutually exclusive segments:

1. pre-treatment period: from day of subject's informed consent to the day before first dose of study medication
2. on-treatment period: from day of first dose of study medication to 30 days after the date of last actual administration of any study medication
3. post-treatment period: starting at day 31 after last dose of study medication.

If dates are incomplete in a way that clear assignment to pre-, on-, or post-treatment period cannot be made, then the respective data will be assigned to the on-treatment period. Additional details to address incomplete AE and/or dosing dates will be addressed in the SAP.

Additional summaries will be displayed to report deaths, all AEs, AEs related to study treatment, all SAEs and SAEs related to study treatment collected up to 150 days after last administration of PDR001/placebo.

Subjects randomized to receive dabrafenib, trametinib, and placebo may be permitted to crossover to dabrafenib, trametinib, and PDR001 in the case that statistically significant interim or final OS results are demonstrated. In this case, a fourth crossover period will be defined as starting on the day of crossover treatment. The SAP will pre-specify details for this scenario.

10.5.3.2 Adverse events (AEs)

Summary tables for adverse events (AEs) will include only AEs that started or worsened during the on-treatment period, the **treatment-emergent** AEs.

The incidence of treatment-emergent adverse events (new or worsening from baseline) will be summarized by system organ class and or preferred term, severity (based on CTCAE v4.03 grades), type of adverse event, and relation to study treatment.

Serious adverse events, non-serious adverse events, and adverse events of special interest (AESI; see Section 8.1.3) during the on-treatment period will be tabulated. The list of AESI's will also include relevant events for dabrafenib and trametinib which will be defined in the SAP.

All deaths (on-treatment and post-treatment) will be summarized.

All AEs, deaths and serious adverse events (including those from the pre and post-treatment periods) will be listed and those starting during the post-treatment period will be flagged.

10.5.3.3 Laboratory abnormalities

Grading of laboratory values will be assigned programmatically as per NCI CTCAE version 4.03. The calculation of CTCAE v4.03 grades will be based on the observed laboratory values only, clinical assessments will not be taken into account.

CTCAE v4.03 Grade 0 will be assigned for all non-missing values not graded as 1 or higher. Grade 5 will not be applicable.

For laboratory tests where grades are not defined by CTCAE v4.03, results will be categorized as low/normal/high based on laboratory normal ranges.

The following listing/summaries will be generated separately for hematology, and biochemistry tests:

- Listing of all laboratory data with values flagged to show the corresponding CTCAE v4.03 grades if applicable and the classifications relative to the laboratory normal ranges

For laboratory tests where grades are defined by CTCAE v4.03

- Worst post-baseline CTCAE v4.03 grade (regardless of the baseline status). Each subject will be counted only once for the worst grade observed post-baseline.
- Shift tables using CTCAE v4.03 grades to compare baseline to the worst on-treatment value

For laboratory tests where grades are not defined by CTCAE v4.03,

- Shift tables using the low/normal/high/ (low and high) classification to compare baseline to the worst on-treatment value.

In addition to the above mentioned tables and listings, other exploratory analyses, for example figures plotting time course of raw or change in laboratory tests over time or box plots might be specified in the SAP.

10.5.3.4 Other safety data

Data from other tests, such as ECG and vital signs, will be listed and summarized accordingly. Notable values will be flagged in the listing. Definitions of notably abnormal results will be specified in the SAP.

ECG

12-lead ECGs including PR, QRS, QT, and QTcF intervals and heart rate will be obtained for each subject during the study. ECG data will be read and interpreted locally.

Categorical analysis of QT/QTc interval data based on the number of subjects meeting or exceeding predefined limits in terms of absolute QT/QTc intervals or changes from baseline will be presented. In addition, a listing of these subjects will be produced by treatment group.

Vital signs

Data on vital signs will be tabulated and listed, and notable values will be flagged.

10.5.3.5 Supportive analyses for secondary objectives

Not applicable.

10.5.3.6 Tolerability

Tolerability of study treatment will be assessed by summarizing the number of dose interruptions and dose reductions. Reasons for dose interruptions and dose reduction will be listed and summarized (see Section 10.3).

10.5.4 Pharmacokinetics

The respective PAS for each study drug will be used in the pharmacokinetic data analysis. Descriptive statistics (n, m (number of non-zero concentrations), mean, CV%, SD, median, geometric mean, geometric CV%, minimum and maximum) for PDR001, dabrafenib and trametinib concentrations will be presented at each scheduled time point by treatment. Pre-dose concentrations collected before dose administration on Day 1 of Cycle 2+ are Ctrough for PDR001. In the cases where dosing of PDR001 starts at Cycle 2 only, Ctrough are considered to be pre-dose concentrations collected before dose administration on Day 1 of Cycle 3+. Pre-dose concentrations collected before dose administration on Day 15 of Cycle 1+ are Ctrough for dabrafenib and trametinib. All concentration data for PDR001, dabrafenib, and trametinib will be displayed graphically by treatment.

10.5.4.1 Data handling principles

Missing values for any PK parameters or concentrations will not be imputed and will be treated as missing.

Below the limit of quantitation (BLQ) values will be set to zero by the Bioanalyst, and will be displayed in the listings as zero and flagged. BLQ values will be treated as missing for the calculation of the geometric means and geometric CV%.

10.5.4.2 Population pharmacokinetic analysis

The pharmacokinetics of dabrafenib and trametinib will be determined using a non-linear mixed effects modeling approach. Population pharmacokinetic parameters including CL/F, V/F, and absorption rate constant (ka) will be estimated, if data permit. Dependent on the final structural

pharmacokinetic model, additional pharmacokinetic parameters may also be estimated. Sources of variability in pharmacokinetic parameters will be investigated during population modeling.

If data permit, a mixed-effects model may be applied to the serum PDR001 concentration-time data to generate *post hoc* estimates of pharmacokinetic parameters using NONMEM to characterize PDR001 exposure in subjects with advanced melanoma.

If there are sufficient data for analysis, the details of the population pharmacokinetic analyses will be provided in a separate SAP, and the results may be reported in a separate population pharmacokinetic report. Data from this and other studies may be pooled for analysis.

10.5.5 Immunogenicity

Immunogenicity will be characterized descriptively by tabulating ADA prevalence at baseline and ADA incidence on-treatment.

10.5.6 Biomarkers

The statistical goals of exploratory biomarker analyses should be considered as promoting the generation of new scientific hypotheses and are generally not powered to assess specific biomarker–related hypotheses. These hypotheses may be compared with results found in literature as well as verified with data derived from subsequent clinical trials. No adjustment for multiple comparisons is usually planned for exploratory analyses. Furthermore, additional post hoc exploratory assessments are expected and may be performed.

There may be circumstances when a decision is made to stop sample collection, or not perform or discontinue the analysis of blood / archival tumor samples / fresh tumor biopsies due to either practical or strategic reasons (e.g., issues related to the quality and/or quantity of the samples or issues related to the assay). Under such circumstances, the number of samples may be inadequate to perform a rigorous data analysis and the available data will only be listed and potentially summarized.

10.5.6.1 Outline of the data analysis

The proposed data analysis will be aligned with the exploratory biomarker objectives of the protocol. Additional analyses that may be performed after the completion of the end-of-study CSR will be documented in separate reports. These analyses may include but are not limited to the meta-analysis of data from this study combined with data from other studies or the analysis of biomarkers generated from samples collected during the study but analyzed after the database lock and completion of the CSR. The data analysis will be described in an addendum of the SAP or in a stand-alone SAP document, as appropriate.

The following exploratory endpoints may be assessed and the details of the analysis will be outlined in a biomarker SAP.

- Protein expression of selected immune biomarkers (e.g., PD-L1 levels and CD8+ cells) in tumor cells (and/or immune cells) and relationship to antitumor activity of PDR001 in combination with dabrafenib and trametinib.

- Presence/absence and localization of specific immune cells and/or specific proteins (e.g., CD8 positive T cells, Treg cells, Macrophages) and their correlation with antitumor activity of the combination PDR001, dabrafenib and trametinib.
- Changes from baseline of tissue and/or plasma derived markers over the course of therapy may be also correlated with antitumor activity of PDR001 in combination with dabrafenib and trametinib.
- Expression (or / absence) of specific genes and gene expression signatures and their correlation with antitumor activity of the combination PDR001, dabrafenib and trametinib, based.
- Correlation of serum cytokines levels (and/or other circulating markers) with antitumor activity of the combination PDR001, dabrafenib and trametinib, in samples collected at baseline and on-treatment.
- Assessment of specific gene alteration(s) or T-cell phenotypes in relation to antitumor activity of the combination PDR001, dabrafenib and trametinib. Additional genomics analyses might be incorporated if sequencing of the biopsies is performed.

10.5.6.2 Data handling principles

Data preprocessing and transformations will be described in detail in the Programming Dataset Specifications document.

10.5.6.3 Data analysis principles

10.5.6.3.1 Analysis sets

Unless otherwise specified, all statistical analyses of biomarker data will be performed on subjects with biomarker data.

10.5.6.3.2 Basic tables, figures and listings

Unless otherwise stated, as project standard, all biomarker data collected will be listed and summarized. In the event of the collection of large biomarker data such as next generation sequencing, gene expression or protein expression panels, some pragmatic considerations will be applied to limit output, as these may easily top thousands, if not tens of thousands of pages offering little to no scientific value.

Depending on the endpoint of interest, graphical displays such as box plots or strip plots may be used to assess the relationship of different biomarkers with clinical benefit. These may be separated by treatment cohort and include either baseline or change from baseline values, where applicable.

For categorical markers such as mutation status, 2x2 contingency tables may be used to assess the relationship with clinical benefit and or Kaplan – Meier curves may be generated given the number of PFS events warrant such an assessment.

10.5.6.3.3 Advanced analysis methods

Not applicable.

10.5.7 Resource utilization

Not applicable.

10.5.8 Patient-reported outcomes

Three patient-reported outcomes questionnaires will be assessed: EORTC QLQ-C30, EQ-5D-5L, and FACT-M Melanoma subscale. Scoring of PRO data and methods for handling of missing items or missing assessments will be handled according to the scoring manual and user guide for each respective patient questionnaire (Fayers 2001, Reenen 2015). No imputation procedures will be applied for missing items or missing assessments.

Descriptive statistics will be used to summarize the scored scales and subscales of the QLQ-C30, EQ-5D-5L and FACT- M Melanoma Subscale at each scheduled assessment time point for each treatment group. Additionally, change from baseline in the scale and subscale values at the time of each assessment will be summarized. Subjects with an evaluable baseline score and at least one evaluable post baseline score during the treatment period will be included in the change from baseline analyses.

The number of subjects completing each questionnaire and the number of missing or incomplete assessments will be summarized by treatment group for each scheduled assessment time point. No formal statistical tests will be performed for PRO data and hence no multiplicity adjustment will be applied. The FAS will be used for analyzing PRO data.

In addition, a repeated measurement analysis model will be used to compare the treatment groups with respect to changes in all 15 domain scores of the EORTC QLQ-C30, the thermometer and utility scores of the EQ-5D-5L, and the FACT-M Melanoma Subscale, longitudinally over time. The repeated measures model will include terms for treatment, stratification factors, time of visit (in weeks, counting from time of randomization to the time of a particular post-baseline measurement in time windows), treatment by time of visit interaction, and baseline score. The differences in least square means between the treatment arms and corresponding 95% confidence interval at selected time points will be presented. The repeated measures model assumes that the missing scores at any time point are missing at random. Additional sensitivity analysis may be performed to assess the possible violation of missing at random assumption for the missing data if deemed appropriate. Details will be specified in the SAP.

Time to definitive 10 point deterioration in the global health status will be assessed for each treatment group. The time to definitive 10 point deterioration is defined as the time from the date of randomization to the date of event, which is defined as at least 10 points relative to baseline worsening of the corresponding scale score or death due to any cause. If a subject has not had an event, time to deterioration will be censored at the date of the last adequate PRO assessment. The distribution will be presented descriptively using Kaplan-Meier curves. Summary statistics from Kaplan-Meier distributions will be determined, including the median time to definitive 10 point deterioration along with two-sided 95% confidence interval. Additionally, time to definitive deterioration with different cut-off definitions (e.g., 5 points, 15 points) may be specified in the RAP as deemed appropriate. A stratified Cox regression will be used to estimate the hazard ratio (HR), along with two-sided confidence interval.

10.6 Exploratory PK and immunogenicity objectives

If feasible, exploratory PK and PK/Pharmacodynamic analysis may be performed, using appropriate methods, to assess the relationship between PDR001 exposure and efficacy, safety, and/or biomarkers. These analyses may be defined in a separate SAP and the results may be reported separately from the CSR.

The impact of ADA on the occurrence of safety endpoints, efficacy endpoints, and PDR001 PK will be assessed using appropriate methods. Further details will be provided in the SAP.

10.7 Interim analysis

Part 1: Safety run-in

No formal interim analysis is planned for this part of the trial. However, the safety run-in design foresees that decisions based on the current data are taken before the end of the study. More precisely, after each cohort of subjects the next dose will be chosen depending on the observed data (based on safety, PK, tolerability data, guided by the recommendations from the BLRM of DLT using EWOC, and recommendations from participating investigators). Details of this procedure and the process for communication with Investigators are provided in Section 6.2.3.

Part 2: Biomarker cohort

No formal interim analysis is planned for this part of the trial.

Part 3: Double-blind, randomized, placebo-controlled part

10.7.1 Progression-Free Survival

An interim PFS analysis is planned after approximately 260 events, which will allow for an early significance claim for a superior PFS result. If PFS is significant at this analysis an interim analysis of OS will also be performed at this time.

The timing and significance boundary of this interim PFS analysis has been chosen so that the efficacy threshold is met only when the PFS treatment effect is sufficiently large and clinically relevant (i.e. using a stringent significance level at interim PFS analysis with a small penalty for the final PFS analysis). The final PFS analysis, if performed at 352 events, will provide an 80% cumulative power to detect a statistically significant result if the delayed effect is indeed 5 months long as observed in KEYNOTE-022 and followed by an effect as currently assumed (i.e. HR=0.60).

A Gamma alpha spending function will be used to control the type 1 error probability with Gamma parameter = -9.7 (Hwang, Shih and DeCani, 1990). EAST version 6.4 will be used to determine the critical thresholds for the analysis based on the actual number of observed events at the time of the analysis. This particular Gamma alpha spending function was selected due to its conservative nature to ensure that statistical significance at interim PFS analysis will only be declared for a clinically relevant PFS treatment effect.

The exact nominal p-values that will need to be observed to declare statistical significance at the time of these analyses for PFS will depend on the number of PFS events that have been observed at the time of these analyses and for the final PFS analysis the α already spent at the time of the interim PFS analysis.

If the number of PFS events at the interim anlaysis is exactly 260 then a significant result will be obtained if p-value <0.00198 (or equivalently if HR<0.700). Similarly assuming 260 events have previously been observed at the interim, if there are exactly 352 events at the final PFS analysis then a significant result will be obtained if p-value <0.02483 (or equivalently if HR<0.811). The interim PFS analysis will be performed by an external independent statistician. This will include an analysis of the primary PFS endpoint plus other critical efficacy endpoints and safety data. The results will be made available to the DMC who will then make a recommendation to Novartis (see Section 8.6).

The DMC recommendation at the interim PFS analysis will be based on whether the predefined efficacy threshold for primary endpoint PFS by investigator was met and will also take into account PFS results by central radiology review. The details on criteria used will be provided in the DMC charter.

If the DMC recommendation is to consider making the unblinded results available, key Novartis personnel will review the (unblinded) interim data and make the final decision on what steps to take. Full details of this decision-making process including who will have access to the unblinded results will be specified in the DMC Charter.

The projected timing of the interim and final PFS analysis based on current study data is summarized in Table 10-1. However, note that due to the limited follow-up the predictions for timing of analyses is highly uncertain. Indeed, doublet data from two pivotal randomized Phase III studies (MEK115306 [COMBI-d]) and MEK116513 [COMBI-v]) indicate that there could be a rapid reduction in the event rate with longer follow-up which would lead to the targeted events occurring much later than predicted from the current study data alone.

Table 10-1 Estimated timelines for interim and final PFS analyses

Months after randomization of the first patient (prediction based on actual study data at Protocol Amendment 5)	# PFS Events (information fraction)	Cumulative PFS Power against a hazard ratio of 1 for first x months and a hazard ratio 0.60 after x months	
		x = 3 months Delayed Effect	x = 5 months Delayed Effect
19	260 (74%)	41.5%	15.5%
25	352 (100%)	93.8%	80.6%
Calculated using EAST 6.4			

10.7.2 Overall Survival (OS)

Interim analyses for OS are planned at the time of the interim and final PFS analyses. A hierarchical testing procedure will be adopted and the statistical tests for OS will be performed only if the primary efficacy endpoint PFS is statistically significant.

A maximum of three analyses are planned for OS;

1. at the time of the interim analysis for PFS (provided PFS is significant),.
2. at the time of the final analysis for PFS (provided interim or final PFS is significant),
3. a final analysis for OS when approximately 245 deaths are expected (expected approximately 36 months from date of first patient to be randomized according to a

prediction analysis using actual study data, although at this point this prediction based on limited study follow-up is still highly uncertain).

An α-spending function according to Lan-DeMets (O'Brien-Fleming) as implemented in EAST (6.4) (Lan and DeMets 1983), along with the testing strategy outlined below will be used to maintain the overall type I error probability. This guarantees the protection of the 2.5% overall level of significance across the repeated testing of the OS hypotheses in the interim and the final analysis (Glimm 2010).

The trial allows for an early significance claim for efficacy for a superior OS result, provided the primary endpoint PFS has already been shown to be statistically significant favoring the test treatment arm. Further, the exact nominal p-values that will need to be observed to declare statistical significance at the time of these analyses for OS will depend on the number of OS events that have been observed at the time of these analyses and the α for OS already spent at the time of earlier analyses.

At the time of final PFS analysis, both PFS and interim OS analyses will be performed by the Sponsor's clinical team. Investigators and patients will remain blinded to study treatment (unless OS reaches significance at any of the PFS analyses) and all patients will continue to be followed for OS until study closure.

10.8 Sample size calculation

Part 1: Safety run-in

No formal statistical power calculations to determine sample size were performed for this part of the study. In the case that the starting dose (PDR001 400 mg i.v. every 4 weeks with the fixed dose combination of 150 mg BID dabrafenib and 2 mg QD trametinib) is confirmed to be safe and tolerated, the safety run-in part is expected to enroll 6 evaluable subjects (i.e. who met the minimum exposure criterion and had sufficient safety evaluations during the first 8 weeks of PDR001 in combination with dabrafenib and trametinib dosing). Otherwise, up to 18 additional subjects are foreseen to be enrolled to assess additional cohorts.

Part 2: Biomarker cohort

No formal statistical power calculations to determine sample size were performed for this part of the study. Approximately 20 evaluable subjects (i.e. who have at least one tumor biopsy at screening and at least two during triple combination therapy) will be enrolled in this cohort.

Part 3: Double-blind, randomized, placebo-controlled part

The sample size calculation is based on the primary variable PFS. The hypotheses to be tested and details of the testing strategy are described in Section 10.4.2.

Based on data from two pivotal randomized Phase III studies (MEK115306 [COMBI-d]) and MEK116513 [COMBI-v]), the median PFS time in the control arm is expected to be 11 months. Ignoring delayed treatment effect, it would be expected that the experimental treatment response would result in a hazard ratio of 0.60 (which corresponds to an increase in median PFS to 18.33 months under the exponential model assumption). Given knowledge of a potential delayed treatment effect, it is hypothesized that there will be no difference between treatment arms until 5 months after the start of treatment for PFS. Therefore, it is assumed that the HR

Supplemental material
BMJ Publishing Group Limited (BMJ) disclaims all liability and responsibility arising from any reliance placed on this supplemental material which has been supplied by the author(s)
J Immunother Cancer

between the groups will be equal to 1 for the first 5 months. Thereafter, exponential survival distributions are assumed, with an HR of 0.60. This will result in an overall HR=0.739 (Kalbfleisch 1981) at the time of the final PFS analysis (given the assumed 5 month delayed treatment effect, this equates to median PFS times of 15.0 and 11 months in the PDR001 in combination with dabrafenib and trametinib combination arm and the dabrafenib and trametinib plus placebo group, respectively).

In order to ensure 80% power for PFS using the above assumptions for HR=1 for the first 5 months and HR=0.60 thereafter, it is calculated that a total of 352 PFS events need to be observed. This calculation was made using the software package EAST 6.4 and assumes analysis by a one-sided log-rank test at the overall 2.5% level of significance where subjects are randomized to the two treatments in a 1:1 ratio.

The calculation also assumes a two-look group sequential design (i.e. that there will be an interim PFS analysis performed after 260 events (73.9% of total)) with a Gamma alpha spending function used to control the type 1 error probability, with Gamma parameter = -9.7 (Hwang, Shih and DeCani, 1990).

It should be noted that the cumulative 80% power refers to the overall HR of 0.739 that combines both the period of no effect and the subsequent delayed effect period and this represents ‘alternative hypothesis’ value.

Based on predictions using actual blinded PFS data from the 532 patients randomized to part 3 of the study which were available at the time of Protocol Amendment 5, the 352 PFS events are expected to occur approximately 25 months after the randomization date of the first subject. The data cutoff for interim PFS analysis (260 events) is expected to occur approximately 19 months after the first patient was randomized. It should be noted that those future predictions are associated with reletively high level of uncertainty since they are based on the data with limited follow up.

The total of approximately 352 PFS events targeted for the final anlaysis represent a large percentage of the patients randomized in the study (66.2%) and therefore the rate of PFS events is likely to decrease with longer follow-up. In addition, formation of a plateau in the PFS Kaplan-Meier curve might occur in the combination arm with PDR001 based on patterns seen in studies with other immune therapy agents and might lead to additional delay in the event accrual.

For those reasons it might actually take significantly longer to obtain the targeted number of PFS event and this is why the final anlaysis cutoff will be chosen based on both event numbers and calendar time.

10.9 Power for analysis of key secondary variables

OS, as the key secondary variable, will be formally statistically tested, provided that the primary variable PFS is statistically significant. The hypotheses to be tested and details of the testing strategy are provided in Section 10.5.1 and Section 10.7.1.

Based on data from two pivotal randomized Phase III studies (MEK115306 [COMBI-d]) and MEK116513 [COMBI-v]), the median OS time in the control arm is expected to be 25 months. Ignoring delayed treatment effect, it would be expected that the experimental treatment

response would result in a hazard ratio of 0.60 (which corresponds to an increase in median OS to 41.67 months under the exponential model assumption). Given knowledge of a potential delayed treatment effect, it is hypothesized that there will be no difference between treatment arms until 5 months after the start of treatment for OS. Therefore, it is assumed that the HR between the groups will be equal to 1 for the first 5 months. Thereafter, exponential survival distributions are assumed, with an HR of 0.60. This will result in an overall HR=0.693 (Kalbfleisch 1981) at the time of final analysis (given the assumed 5 month delayed treatment effect, this equates to median OS times of 38.3 and 25 months in the PDR001 in combination with dabrafenib and trametinib combination arm and the dabrafenib and trametinib plus placebo group, respectively).

In order to ensure 80% power for OS using the above assumptions for HR=1 for the first 5 months and HR=0.60 thereafter, it is calculated that a total of 245 deaths need to be observed. This calculation assumes analysis by a one-sided log-rank test at the overall 2.5% level of significance, subjects randomized to the two treatments in a 1:1 ratio, and a 3-look group sequential design with a Lan-DeMets (O'Brien-Fleming) alpha spending function

At the moment it is inappropriate to give the number of deaths and information fractions expected at the interim OS analyses due to the high uncertainty of event predictions for both PFS (which directly determines the timing of the interim OS analyses) and for OS itself.

These calculations were made using the software package EAST 6.4.

It should be noted that the cumulative 80% power refers to the overall HR of 0.693 that combines both the period of no effect and the subsequent delayed effect period and this represents 'alternative hypothesis' value.

At the final OS analysis 245 deaths will need to be observed. The final OS analysis is expected to be performed after approximately 36 months from the date of first subject randomized based on a prediction analysis using actual study data. However, note that this prediction is also associated with high uncertainty as described above.

11 Ethical considerations and administrative procedures

11.1 Regulatory and ethical compliance

This clinical study was designed, shall be implemented and reported in accordance with the ICH Harmonized Tripartite Guidelines for Good Clinical Practice, with applicable local regulations (including European Directive 2001/20/EC and US Code of Federal Regulations Title 21), and with the ethical principles laid down in the Declaration of Helsinki.

11.2 Responsibilities of the investigator and IRB/IEC/REB

The protocol and the proposed informed consent form must be reviewed and approved by a properly constituted Institutional Review Board/Independent Ethics Committee/Research Ethics Board (IRB/IEC/REB) before study start. Prior to study start, the investigator is required to sign a protocol signature page confirming his/her agreement to conduct the study in accordance with these documents and all of the instructions and procedures found in this protocol and to give access to all relevant data and records to Novartis monitors, auditors,

Novartis Clinical Quality Assurance representatives, designated agents of Novartis, IRBs/IECs/REBs and regulatory authorities as required.

11.3 Informed consent procedures

Eligible subjects may only be included in the study after providing written (witnessed, where required by law or regulation), IRB/IEC/REB-approved informed consent

Informed consent must be obtained before conducting any study-specific procedures (i.e. all of the procedures described in the protocol). The process of obtaining informed consent should be documented in the subject source documents. The date when a subject's Informed Consent was actually obtained will be captured in their CRFs.

Novartis will provide to investigators, in a separate document, a proposed informed consent form (ICF) that is considered appropriate for this study and complies with the ICH GCP guideline and regulatory requirements. Any changes to this ICF suggested by the investigator must be agreed to by Novartis before submission to the IRB/IEC/REB, and a copy of the approved version must be provided to the Novartis monitor after IRB/IEC/REB approval.

Women of child bearing potential should be informed that taking the study medication may involve unknown risks to the fetus if pregnancy were to occur during the study and agree that in order to participate in the study they must adhere to the contraception requirement for the duration of the study. If there is any question that the subject will not reliably comply, they should not be entered in the study.

Additional consent form

Sub-studies and studies with an optional Exploratory Biomarker component will have a separate consent form or an option (check box) under the study main ICF covering those studies. This form will be adapted for each Study based on a standard template used globally for all Studies. These informed consent forms will be submitted for ethical approval together with the Study Protocol and the main informed consent form of the Study. If a subject opts not to participate in the optional assessments, this in no way affects the subject's ability to participate in the main research study.

11.4 Discontinuation of the study

Novartis reserves the right to discontinue this study under the conditions specified in the clinical study agreement. Specific conditions for terminating the study are outlined in Section 4.4.

11.5 Publication of study protocol and results

Novartis is committed to following high ethical standards for reporting study results for its innovative medicine, including the timely communication and publication of clinical trial results, whatever their outcome. Novartis assures that the key design elements of this protocol will be posted on the publicly accessible database, e.g., www.clinicaltrials.gov before study start. In addition, results of interventional clinical trials in adult subjects are posted on www.novartisclinicaltrials.com, a publicly accessible database of clinical study results within 1 year of study completion (i.e., LPLV), those for interventional clinical trials involving pediatric subjects within 6 months of study completion.

Novartis follows the ICMJE authorship guidelines (www.icmje.org) and other specific guidelines of the journal or congress to which the publication will be submitted.

Authors will not receive remuneration for their writing of a publication, either directly from Novartis or through the professional medical writing agency. Author(s) may be requested to present their writing of a publication, either directly from Novartis or through the professional medical writing agency. Author(s) maybe requested to present poster or oral presentation at scientific congress; however, there will be no honorarium provided for such presentations.

As part of its commitment to full transparency in publications, Novartis supports the full disclosure of all funding sources for the study and publications, as well as any actual and potential conflicts of interest of financial nature by all authors, including medical writing and editorial support, if applicable.

For the Novartis Guidelines for the Publication of Results from Novartis-sponsored Research, please refer to www.novartis.com.

Publication section text for Novartis-sponsored Phase I healthy volunteer studies:

In general, Phase I healthy volunteer studies will not be registered on publicly accessible database. However, Novartis will comply with local country regulations for trial registration and results posting, as needed.

According to Novartis policy, authors of publication will not receive remuneration for their writing of a publication, either directly from Novartis or through the professional medical writing agency. Author(s) may be requested to present poster or oral presentation at scientific congress; however, there will be no honorarium provided for such presentations.

For the Novartis Guidelines for the Publication of Results from Novartis-sponsored Research, please refer to www.novartis.com.

11.6 Study documentation, record keeping and retention of documents

Each participating site will maintain appropriate medical and research records for this trial, in compliance with Section 4.9 of the ICH E6 GCP, and regulatory and institutional requirements for the protection of confidentiality of subjects. As part of participating in a Novartis-sponsored study, each site will permit authorized representatives of the sponsor(s) and regulatory agencies to examine (and when required by applicable law, to copy) clinical records for the purposes of quality assurance reviews, audits and evaluation of the study safety and progress.

Source data are all information, original records of clinical findings, observations, or other activities in a clinical trial necessary for the reconstruction and evaluation of the trial. Examples of these original documents and data records include, but are not limited to, hospital records, clinical and office charts, laboratory notes, memoranda, subjects' diaries or evaluation checklists, pharmacy dispensing records, recorded data from automated instruments, copies or transcriptions certified after verification as being accurate and complete, microfiches, photographic negatives, microfilm or magnetic media, x-rays, and subject files and records kept at the pharmacy, at the laboratories, and medico-technical departments involved in the clinical trial.

Data collection is the responsibility of the clinical trial staff at the site under the supervision of the site Principal Investigator. The study CRF is the primary data collection instrument for the study. The investigator should ensure the accuracy, completeness, legibility, and timeliness of the data reported in the CRFs and all other required reports. Data reported on the CRF, that are derived from source documents, should be consistent with the source documents or the discrepancies should be explained. All data requested on the CRF must be recorded. Any missing data must be explained. Any change or correction to a paper CRF should be dated, initialed, and explained (if necessary) and should not obscure the original entry. For electronic CRFs an audit trail will be maintained by the system. The investigator should retain records of the changes and corrections to paper CRFs.

The investigator/institution should maintain the trial documents as specified in Essential Documents for the Conduct of a Clinical Trial (ICH E6 Section 8) and as required by applicable regulations and/or guidelines. The investigator/institution should take measures to prevent accidental or premature destruction of these documents.

Essential documents (written and electronic) should be retained for a period of not less than fifteen (15) years from the completion of the Clinical Trial unless Sponsor provides written permission to dispose of them or, requires their retention for an additional period of time because of applicable laws, regulations and/or guidelines.

11.7 Confidentiality of study documents and subject records

The investigator must ensure anonymity of the subjects; subjects must not be identified by names in any documents submitted to Novartis. Signed informed consent forms and subject enrollment log must be kept strictly confidential to enable subject identification at the site.

11.8 Audits and inspections

Source data/documents must be available to inspections by Novartis or designee or Health Authorities.

11.9 Financial disclosures

Financial disclosures should be provided by study personnel who are directly involved in the treatment or evaluation of subjects at the site - prior to study start.

12 Protocol adherence

Investigators ascertain they will apply due diligence to avoid protocol deviations. Under no circumstances should the investigator contact Novartis or its agents, if any, monitoring the study to request approval of a protocol deviation, as no authorized deviations are permitted. If the investigator feels a protocol deviation would improve the conduct of the study this must be considered a protocol amendment, and unless such an amendment is agreed upon by Novartis and approved by the IRB/IEC/REB it cannot be implemented. All significant protocol deviations will be recorded and reported in the CSR.

12.1 Amendments to the protocol

Any change or addition to the protocol can only be made in a written protocol amendment that must be approved by Novartis, Health Authorities where required, and the IRB/IEC/REB. Only amendments that are required for subject safety may be implemented prior to IRB/IEC/REB approval. Notwithstanding the need for approval of formal protocol amendments, the investigator is expected to take any immediate action required for the safety of any subject included in this study, even if this action represents a deviation from the protocol. In such cases, Novartis should be notified of this action and the IRB/IEC at the study site should be informed according to local regulations (e.g., UK requires the notification of urgent safety measures within 3 days) but not later than 10 working days.

13 References (available upon request)

Aaronson NK, Ahmedzai S, Bergman B, et al (1993). The European Organization for Research and Treatment of Cancer QLQ-C30: A quality-of-life instrument for use in international clinical trials in oncology. Journal of the National Cancer Institute; 85: 365-376

Anker CJ, Grossmann KF, Atkins MB, et al (2016). Avoiding severe toxicity from combined BRAF inhibitor and radiation treatment: consensus guidelines from the Eastern cooperative oncology group (ECOG). Int J Radiat Oncol Biol Phys; 95(2): 632 - 646

Ascierto PA, Ferrucci PF, Fisher R, et al (2018a). KEYNOTE-022 Part 3: Phase 2 Randomized Study of First-Line Dabrafenib and Trametinib Plus Pembrolizumab or Placebo for BRAF-Mutant Advanced Melanoma. Presentation by P.A. Ascierto at ESMO 2018.

Ascierto PA, Ferrucci PF, Stephens R, et al. (2018b). KEYNOTE 022 Part 3: Phase II randomized study of 1L dabrafenib (D) and trametinib (T) plus pembrolizumab (Pembro) or placebo (PBO) for BRAF mutant advanced melanoma. Annals of Oncology 29 (Supplement 8): viii442 viii466, 2018, doi:10.1093/annonc/mdy289

AWMF S3-Leitlinie Melanom (2016). http://www.awmf.org/leitlinien/detail/ll/032-024OL.html

Balch CM, Buzaid AC, Soong SJ, et al (2001). Final version of the American Joint Committee on Cancer staging system for cutaneous melanoma. J Clin Oncol; 19:3635-3648

Boni A, Cogdill AP, Dang P, et al (2010). Selective *BRAF* V600E inhibition enhances T-cell recognition of melanoma without affecting lymphocyte function. Cancer Res; 70(13):5213-5219

Brahmer, J. R., Lacchetti, C., Schneider, B. J., et al (2018). Management of Immune-Related Adverse Events in Patients Treated With Immune Checkpoint Inhibitor Therapy: American Society of Clinical Oncology Clinical Practice Guideline. *J Clin Oncol, 36*(17), 1714-1768. doi:10.1200/jco.2017.77.6385

Callahan MK, Masters G, Pratilas CA, et al (2014). Paradoxical activation of T cells via augmented ERK signaling mediated by a RAF inhibitor. Cancer Immunol Res; 2(1):70-79

Carnahan J, Beltran PJ, Babij C, et al (2010). Selective and potent Raf inhibitors paradoxically stimulate normal cell proliferation and tumor growth. Molecular Cancer Therapeutics; 9:2399-2410

Chapman PB, Hauschild A, Robert C, et al (2011). Improved survival with vemurafenib in melanoma with *BRAF* V600E mutation. N Engl J Med; 364: 2507-2516

Corcoran RB, Dias-Santagata D, Bergethon K, et al (2010). *BRAF* gene amplification can promote acquired resistance to MEK inhibitors in cancer cells harboring the *BRAF* V600E mutation. Sci Signal; 3(149):ra84

Cormier JN, Ross MI, Gershenwald JE, et al (2008). Prospective assessment of the reliability, validity, and sensitivity to change of the Functional Assessment of Cancer Therapy-Melanoma questionnaire. Cancer; 112:2249-2257

Cutaneous Melanoma: ESMO Guideline Committee (2015). Cutaneous Melanoma: ESMO Clinical Practice Guidelines for diagnosis, treatment adn follow up. Ann Oncol 26 (suppl 5): v126-v132

Dhillon AS, Hagan S, Rath O, et al (2007). MAP kinase signalling pathways in cancer. Oncogene; 26(22):3279-3290

Eggermont AM, Chiarion-Sileni V, Grobb JJ, et al (2015). Adjuvant ipilimumab versus placebo after complete resection of high-risk stage III melanoma (EORTC 18071): a randomized, double-blind, phase 3 trial. Lancet; 16: 522-530

Emery CM, Vijayendran KG, Zipser MC, et al (2009). MEK1 mutations confer resistance to MEK and B-RAF inhibition. Proc Natl Acad Sci U S A; 106:20411-20416

EUCAN (2012). Estimated incidence, mortaility & prevalence in men, 2012. Cancer factsheets http://eco.iarc.fr/eucan/Cancer.aspx?Cancer=20

Eton O, Legha SS, Moon TE, et al (1998). Prognostic factors for survival of patients treated systemically for disseminated melanoma. J Clin Oncol; 16:1103-1111

Fayers PM, Aaronson NK, Bjordal K, et al (2001). The EORTC QLQ-C30 Scoring Manual (3rd Edition). European Organisation for Research and Treatment of Cancer

FDA 2011 https//www.accessdata.fda.gov/scripts/cder/drugsatfda/index.cfm?fuseaction=Search.DrugDetails

Ferlay J, Soerjomataram I, Dikshit R, et al (2015). Cancer incidence and mortality worldwide: sources, methods and major patterns in GLOBOCAN 2012. Int J Cancer; 136(5):E359-386

Flaherty KT, Infante JR, Daud A, et al (2012). Combined *BRAF* and MEK inhibition in melanoma with *BRAF* V600 mutations. N Engl J Med; 367: 1694-1703

Fleming TR, Harrington DP (1991). Counting Processes and Survival Analysis. John Wiley & Sons: New York

Flaherty KT, Puzanov I, Kim KB, et al (2010). Inhibition of mutated, activated *BRAF* in metastatic melanoma. N Engl J Med; 363:809-819

Frederick DT, Piris A, Cogdill AP, et al (2013). *BRAF* inhibition is associated with enhanced melanoma antigen expression and a more favorable tumor microenvironment in patients with metastatic melanoma.Clin Cancer Res; 19(5):1225-1231

Freeman G (2008). Structures of PD-1 with its ligands: Sideways and dancing cheek to cheek. Proc Natl Acad Sci USA; 105(30): 10275-10276

Friedman CF, Proverbs-Singh TA, Postow MA (2016). Treatment of the Immune-Related Adverse Effects of Immune Checkpoint Inhibitors. JAMA Oncol; 2(10):1346-1353

Gershenwald JE, Scolyer RA, Hess KR, et al. Melanoma of the Skin. In: AJCC Cancer Staging Manual: Eighth Edition, Amin MB. (Ed), American Joint Committee on Cancer, Chicago 2017.p.577-578.

Hauschild A, Grob JJ, Demidov LV, et al (2012). Dabrafenib in *BRAF*-mutated metastatic melanoma: a multicentre, open-label, phase 3 randomised controlled trial. Lancet; 380: 358-365

Hodi FS, O'Day SJ, McDermott DF, et al (2010). Improved survival with ipilimumab in patients with metastatic melanoma. N Engl J Med; 363(8): 711-723

Hofmann L, Forschner A, Loquai C, et al (2016). Cutaneous, gastrointestinal, hepatic, endocrine, and renal side-effects of anti-PD-1 therapy. Eur J Cancer; 60:190-209

Hoos, A., Eggermont, A. M., Janetzki, S., et al (2010). Improved endpoints for cancer immunotherapy trials. *J Natl Cancer Inst, 102*(18), 1388-1397. doi:10.1093/jnci/djq310

Hu-Lieskovan S, Mok S, Homet Moreno B, et al (2015). Improved antitumor activity of immunotherapy with *BRAF* and MEK inhibitors in *BRAF* (V600E) melanoma. Sci Transl Med; 7(279):279ra41

Hwang IK, Shih WJ, and DeCani JS (1990). Group sequential designs using a family of type I error probability spending functions. Statistics in Medicine, 9, 1439-1445.

Jemal A, Siegel R, Ward E, et al (2006). Cancer statistics. CA Cancer J Clin; 56(2):106-130

Kakavand H, Willmott JS, Menzies AM, et al (2015). PD-L1 Expression and Tumor-Infiltrating Lymphocytes Define Different Subsets of MAPK Inhibitor-Treated Melanoma Patients. Clin Cancer Res; 15; 21(14):3140-3148

Kalbfleisch JD, Prentice RL (1981). Estimation of the Average Hazard Ratio. Biometrika, 68; 105-112

Keytruda® (pembrolizumab) USPI 2015

Kono M, Dunn IS, Durda PJ, et al (2006). Role of the mitogen-activated protein kinase signaling pathway in the regulation of human melanocytic antigen expression. Mol Cancer Rest; 4(10):779-792

Larkin J, Ascierto PA, Dréno B, et al (2014). Combined Vemurafenib and Cobimetinib in *BRAF*-Mutated Melanoma. New Engl J Med; 371:1867-1876

Larkin J, Chiarion-Sileni V, Gonzalez R, et al (2015). Combined Nivolumab and Ipilimumab or Monotherapy in Untreated Melanoma. New Engl J Med; 373(1): 23-34

Liu L, Mayes PA, Eastman S (2015). The *BRAF* and MEK Inhibitors Dabrafenib and Trametinib: Effects on Immune Function and in Combination with Immunomodulatory Antibodies Targeting PD-1, PD-L1, and CTLA-4. Clin Cancer Res; 21(7):1639-1651

Long GV, Sosman JA, Daud AI et al (2012). Phase II three-arm randomised study of the BRAF inhibitor dabrafenib alone vs combination with MEK1/2 inhibitor trametinib in patients with BRAF V600 mutation-positive metastatic melanoma. 2012 ESMO Congress. Abstract LBA27

Long GV, Hamid O, Hodi FS, et al (2016). J Clin Oncol No 15_suppl (May 20 Supplement) 2016 Abstract TPS9596 Source URL ..://meeting.ascopubs.org/cgi/content/short/34/15_suppl/TPS9596?rss=1

Long GV, Stroyakovskiy D, Gogas H, et al (2014). Combined *BRAF* and MEK inhibition versus *BRAF* inhibition alone in melanoma. N Engl J Med; 371(20): 1877-1888

Maio M, Grob JJ, Aamdal S, et al (2015). Five-year survival rates for treatment-naive patients with advanced melanoma who received ipilimumab plus dacarbazine in a phase III trial. J Clin Oncol; 33(10):1191-1196

Naing A, Gelderblom H, et al (2016). A first-in-human phase I/II study of the anti-PD-1 antibody PDR001 in patients with advanced solid tumors, American Society of Clinical Oncology abstract # 3060

NCCN Guidelines for Patients® Melanoma V1 (2017). Clinical Practice Guidelines Oncology, Melanoma Version 1.2017. Source URL nccn.org/professionals/physician_gls/PDF/melanoma.pdf

Neuenschwander B, Capkun-Niggli G, Branson M, et al (2010). Summarizing historical information on controls in clinical trials. Clin Trials; 7:5-18

Neuenschwander B, Matano A, Tang Z, et al (2014). A Bayesian Industry Approach to Phase I Combination Trials in Oncology. Statistical Methods in Drug Combination Studies

Nishino M, Gargano M, Suda M, et al (2014). Optimizing immune-related tumor response assessment: does reducing the number of lesions impact response assessment in melanoma patients treated with ipilimumab? J Immunother Cancer; 2:17

Nissan MH, Solit DB (2011). The "SWOT" of *BRAF* inhibition in melanoma: RAF inhibitors, MEK inhibitors or both? Curr Oncol Rep; 13(6):479-487

Opdivo (Nivolumab) USPI 2015

Pardoll DM (2012). The blockade of immune checkpoints in cancer immunotherapy. Nat Rev Cancer; 12(4):252-264

Patnaik A, Kang SP, Rasco D, et al (2015). Phase I Study of Pembrolizumab (MK-3475; Anti-PD-1 Monoclonal Antibody) in Patients with Advanced Solid Tumors. Clin Cancer Res; 21(19):4286-4293

Postow MA, Chesney J, Pavlick AC, et al (2015). Nivolumab and ipilimumab versus ipilimumab in untreated melanoma. N Engl J Med; 372(21):2006-2017

Puzanov, I., Diab, A., Abdallah, K., et al (2017). Managing toxicities associated with immune checkpoint inhibitors: consensus recommendations from the Society for Immunotherapy of Cancer (SITC) Toxicity Management Working Group. *J Immunother Cancer, 5*(1), 95. doi:10.1186/s40425-017-0300-z

Rabin R, de Charro F (2001). EQ-5D: a measure of health status from the EuroQol Group. Ann Med; 33(5):337-343

Reenen MV, Janssen B (2015). EQ-5D-5L User Guide: Basic information on how to use the EQ-5D-5L instrument. Version 2.1. The EuroQoL Group

Ribas A (2015). Pembrolizumab versus investigator-choice chemotherapy for ipilimumab-refractory melanoma (KEYNOTE-002): a randomised, controlled, phase 2 trial. The Lancet Oncology; 16(8):908–918

Ribas AF, Hodi S, Lawrence DP, et al (2016). Pembrolizumab (pembro) in combination with dabrafenib (D) and trametinib (T) for *BRAF*-mutant advanced melanoma: Phase 1 KEYNOTE-022 study. J Clin Oncol 34, 2016 (suppl; abstr 3014) ASCO Abstract 3014 Source URL: ..://meetinglibrary.asco.org/content/167137-176

Riley JL (2009). PD-1 signaling in primary T cells. Immunol Rev; 229:114-125

Robert C, Arnault JP, Mateus C (2011). RAF inhibition and induction of cutaneous squamous cell carcinoma. Curr Opin Oncol; 23:177-182

Robert C, Karaszewska B, Schachter J, et al (2015). Improved overall survival in melanoma with combined dabrafenib and trametinib. N Engl J Med; 372:320-330

Robert, C., Long, G. V., Brady, B., et al (2015). Nivolumab in previously untreated melanoma without BRAF mutation. *N Engl J Med, 372*(4), 320-330. doi:10.1056/NEJMoa1412082

Robert C, Ribas A, Wolchok JD, et al (2014). Anti-programmed-death-receptor-1 treatment with pembrolizumab in ipilimumab-refractory advanced melanoma: a randomised dose-comparison cohort of a phase 1 trial. Lancet; 384(9948):1109-1117

Rogatko A, Schoeneck D, Jonas W, et al (2007). Translation of innovative designs into phase I trials. J Clin oncol; 25(31):4982-4986

Schadendorf D, Amonkar MM, Stroyakovskiy D (2015). Health-related quality of life impact in a randomised phase III study of the combination of dabrafenib and trametinib versus dabrafenib monotherapy in patients with *BRAF* V600 metastatic melanoma. Eur J Cancer; 51:833– 840

Schachter, J., Ribas, A., Long, G. V., et al (2017). Pembrolizumab versus ipilimumab for advanced melanoma: final overall survival results of a multicentre, randomised, open-label phase 3 study (KEYNOTE-006). *Lancet, 390*(10105), 1853-1862. doi:10.1016/s0140-6736(17)31601-x

Schilling B, Sondermann W, Zhao F, et al (2014). Differential influence of vemurafenib and dabrafenib on patients' lymphocytes despite similar clinical efficacy in melanoma. Ann Oncol; 25(3):747-753

Seymour L, Bogaerts J, Perrone A, et al (2017). iRECIST: guidelines for response criteria for use in trials testing immunotherapeutics. Lancet Oncol 2017; 18:e143-52

Sirott MN, Bajorin DF, Wong GY, et al (1993). Prognostic factors in patients with metastatic malignant melanoma: a multivariate analysis. Cancer; 72:3091-3098

Spiegelhalter DJ, Abrams KR, Myles JP (2004). Bayesian Approaches to Clinical Trials and Health-Care Evaluation. John Wiley & Sons, New York

Sumimoto H, Imabayashi F, Iwata T, et al (2006). The *BRAF*-MAPK signaling pathway is essential for cancer-immune evasion in human melanoma cells. J Exp Med; 203(7):1651-1656

Surveillance, epidemiology, and end results program (SEER) (2016). SEER Stat Fact Sheets: Melanoma of the Skin. National Cancer Institute Source URL ...://seer.cancer.gov/statfacts/html/melan/html

The EuroQol Group (1990). EuroQol - a new facility for the measurement of health-related quality of life. Health Policy 16(3):199-208

Uno, H., Claggett, B., Tian, L., et al (2014). Moving beyond the hazard ratio in quantifying the between-group difference in survival analysis. J Clin Oncol, 32(22), 2380-2385. doi:10.1200/jco.2014.55.2208

Vella LJ, Pasam A, Dimopoulos N, et al (2014). MEK inhibition, alone or in combination with *BRAF* inhibition, affects multiple functions of isolated normal human lymphocytes and dendritic cells.Cancer Immunol Res; 2(4):351-360

Wargo JA, Cooper ZA, Flaherty KT (2014). Universes collide: combining immunotherapy with targeted therapy for cancer. Cancer Discov; 4(12):1377-1386

Weber JS, D'Angelo SP, Minor D, et al (2015). Nivolumab versus chemotherapy in patients with advanced melanoma who progressed after anti-CTLA-4 treatment (CheckMate 037): a randomised, controlled, open-label, phase 3 trial.Lancet Oncol; 16(4):375-384

Wilmott JS, Long GV, Howle JR, et al (2012). Selective *BRAF* inhibitors induce marked T-cell infiltration into human metastatic melanoma.Clin Cancer Res; 18(5):1386-1394

Wolchok, J. D., Chiarion-Sileni, V., Gonzalez, R., et al (2017). Overall Survival with Combined Nivolumab and Ipilimumab in Advanced Melanoma. *N Engl J Med, 377*(14), 1345-1356. doi:10.1056/NEJMoa1709684

Wolchok, J. D., Hoos, A., O'Day, S., et al (2009). Guidelines for the evaluation of immune therapy activity in solid tumors: immune-related response criteria. *Clin Cancer Res, 15*(23), 7412-7420. doi:10.1158/1078-0432.ccr-09-1624

Yervoy™ (Ipilimumab) USPI 2011

14 Appendices

14.1 Appendix 1: RECIST 1.1

Harmonization of Efficacy Analysis of Solid Tumor Studies

Guidelines for Response, Duration of Overall Response, TTF, TTP, Progression-Free Survival, and Overall Survival (based on RECIST 1.1)

Document type:	TA Specific Guideline
Document status:	Version 3.2: February 11, 2016<br>Version 3.1: November 29, 2011<br>Version 3: October 19, 2009<br>Version 2: January 18, 2007<br>Version 1: December 13, 2002
Release date:	11-Feb-2016
Authors (Version 3.2):	[redacted]
Authors (Version 3.1):	[redacted]
Authors (Version 3):	[redacted]
Authors (Version 2):	[redacted]
Authors (Version 1):	[redacted]

Glossary

CR	Complete response
CRF	Case Report Form
CSR	Clinical Study Report
CT	Computed tomography
DFS	Disease-free survival
eCRF	Electronic Case Report Form
FPFV	First patient first visit
GBM	Glioblastoma multiforme
MRI	Magnetic resonance imaging
LPLV	Last patient last visit
OS	Overall survival
PD	Progressive disease
PFS	Progression-free survival
PR	Partial response
RAP	Reporting and Analysis Plan
RECIST	Response Evaluation Criteria in Solid Tumors
SD	Stable disease
SOD	Sum of Diameter
TTF	Time to treatment failure
TTP	Time to progression
UNK	Unknown

14.1.1 Introduction

The purpose of this document is to provide the working definitions and rules necessary for a consistent and efficient analysis of efficacy for oncology studies in solid tumors. This document is based on the RECIST criteria for tumor responses (Therasse et al 2000) and the revised RECIST 1.1 guidelines (Eisenhauer et al 2009).

The efficacy assessments described in Section 14.1.2 and the definition of best response in Section 14.1.3.1 are based on the RECIST 1.1 criteria but also give more detailed instructions and rules for determination of best response. Section 14.1.3.2 is summarizing the "time to event" variables and rules which are mainly derived from internal discussions and regulatory consultations, as the RECIST criteria do not define these variables in detail. Section 14.1.4 of this guideline describes data handling and programming rules. This section is to be referred to in the SAP (Statistical Analysis Plan) to provide further details needed for programming.

14.1.2 Efficacy assessments

Tumor evaluations are made based on RECIST criteria (Therasse et al 2000), New Guidelines to Evaluate the Response to Treatment in Solid Tumors, Journal of National Cancer Institute, Vol. 92; 205-16 and revised RECIST guidelines (version 1.1) (Eisenhauer et al 2009) European Journal of Cancer; 45:228-247.

14.1.2.1 Definitions

14.1.2.1.1 Disease measurability

In order to evaluate tumors throughout a study, definitions of measurability are required in order to classify lesions appropriately at baseline. In defining measurability, a distinction also needs to be made between nodal lesions (pathological lymph nodes) and non-nodal lesions.

- **Measurable disease** - the presence of at least one measurable nodal or non-nodal lesion. If the measurable disease is restricted to a solitary lesion, its neoplastic nature should be confirmed by cytology/histology.

For patients without measurable disease see Section 14.1.3.2.8.

Measurable lesions (both nodal and non-nodal)

- Measurable non-nodal - As a rule of thumb, the minimum size of a measurable non-nodal target lesion at baseline should be no less than double the slice thickness or 10mm whichever is greater - e.g., the minimum non-nodal lesion size for CT/MRI with 5mm cuts will be 10 mm, for 8 mm contiguous cuts the minimum size will be 16 mm.
- Lytic bone lesions or mixed lytic-blastic lesions with identifiable soft tissue components, that can be evaluated by CT/MRI, can be considered as measurable lesions, if the soft tissue component meets the definition of measurability.
- Measurable nodal lesions (i.e. lymph nodes) - Lymph nodes ≥15 mm in short axis can be considered for selection as target lesions. Lymph nodes measuring ≥10 mm and <15 mm are considered non-measurable. Lymph nodes smaller than 10 mm in short axis at baseline, regardless of the slice thickness, are normal and not considered indicative of disease.

- Cystic lesions:
  - Lesions that meet the criteria for radiographically defined simple cysts (i.e., spherical structure with a thin, non-irregular, non-nodular and non-enhancing wall, no septations, and low CT density [water-like] content) should not be considered as malignant lesions (neither measurable nor non-measurable) since they are, by definition, simple cysts.
  - 'Cystic lesions' thought to represent cystic metastases can be considered as measurable lesions, if they meet the definition of measurability described above. However, if noncystic lesions are present in the same patient, these are preferred for selection as target lesions.
- Non-measurable lesions - all other lesions are considered non-measurable, including small lesions (e.g., longest diameter <10 mm with CT/MRI or pathological lymph nodes with ≥ 10 to < 15 mm short axis), as well as truly non-measurable lesions e.g., blastic bone lesions, leptomeningeal disease, ascites, pleural/pericardial effusion, inflammatory breast disease, lymphangitis cutis/pulmonis, abdominal masses/abdominal organomegaly identified by physical exam that is not measurable by reproducible imaging techniques.

14.1.2.2 Methods of tumor measurement - general guidelines

In this document, the term "contrast" refers to intravenous (i.v.) contrast.

The following considerations are to be made when evaluating the tumor:

- All measurements should be taken and recorded in metric notation (mm), using a ruler or calipers. All baseline evaluations should be performed as closely as possible to the beginning of treatment and never more than 4 weeks before the beginning of the treatment.
- Imaging-based evaluation is preferred to evaluation by clinical examination when both methods have been used to assess the antitumor effect of a treatment.
- For optimal evaluation of patients, the same methods of assessment and technique should be used to characterize each identified and reported lesion at baseline and during follow-up. Contrast-enhanced CT of chest, abdomen and pelvis should preferably be performed using a 5 mm slice thickness with a contiguous reconstruction algorithm. CT/MRI scan slice thickness should not exceed 8 mm cuts using a contiguous reconstruction algorithm. If, at baseline, a patient is known to have a medical contraindication to CT contrast or develops a contraindication during the trial, the following change in imaging modality will be accepted for follow up: a non-contrast CT of chest (MRI not recommended due to respiratory artifacts) plus contrast-enhanced MRI of abdomen and pelvis.
- A change in methodology can be defined as either a change in contrast use (e.g., keeping the same technique, like CT, but switching from with to without contrast use or vice-versa, regardless of the justification for the change) or a major change in technique (e.g., from CT to MRI, or vice-versa), or a change in any other imaging modality. A change from conventional to spiral CT or vice versa will not constitute a major "change in method" for the purposes of response assessment. A change in methodology will result by default in a UNK overall lesion response assessment as per Novartis calculated response. However, another response assessment than the Novartis calculated UNK response may be accepted

from the investigator or the central blinded reviewer if a definitive response assessment can be justified, based on the available information.

- FDG-PET: can complement CT scans in assessing progression (particularly possible for 'new' disease). New lesions on the basis of FDG-PET imaging can be identified according to the following algorithm:
  - Negative FDG-PET at baseline, with a positive FDG-PET at follow-up is a sign of PD based on a new lesion.
  - No FDG-PET at baseline with a positive FDG-PET at follow-up:
- If new disease is indicated by a positive PET scan but is not confirmed by CT (or some other conventional technique such as MRI) at the same assessment, then follow-up assessments by CT will be needed to determine if there is truly progression occurring at that site. In all cases PD will be the date of confirmation of new disease by CT (or some other conventional technique such as MRI) rather than the date of the positive PET scan. If there is a positive PET scan without any confirmed progression at that site by CT, then a PD cannot be assigned.
  - If the positive FDG-PET at follow-up corresponds to a pre-existing site of disease on CT that is not progressing on the basis of the anatomic images, this is not PD.
  - Chest x-ray: Lesions on chest x-ray are acceptable as measurable lesions when they are clearly defined and surrounded by aerated lung. However, CT is preferable.
  - Physical exams: Evaluation of lesions by physical examination is accepted when lesions are superficial, with at least 10mm size, and can be assessed using calipers.
  - Ultrasound: When the primary endpoint of the study is objective response evaluation, ultrasound (US) should not be used to measure tumor lesions, unless pre-specified by the protocol. It is, however, a possible alternative to clinical measurements of superficial palpable lymph nodes, subcutaneous lesions and thyroid nodules. US might also be useful to confirm the complete disappearance of superficial lesions usually assessed by clinical examination.
  - Endoscopy and laparoscopy: The utilization of endoscopy and laparoscopy for objective tumor evaluation has not yet been fully and widely validated. Their uses in this specific context require sophisticated equipment and a high level of expertise that may only be available in some centers. Therefore, the utilization of such techniques for objective tumor response should be restricted to validation purposes in specialized centers. However, such techniques can be useful in confirming complete pathological response when biopsies are obtained.
  - Tumor markers: Tumor markers alone cannot be used to assess response. However, some disease specific and more validated tumor markers (e.g., CA-125 for ovarian cancer, PSA for prostate cancer, alpha-FP, LDH and Beta-hCG for testicular cancer) can be integrated as non-target disease. If markers are initially above the upper normal limit they must normalize for a patient to be considered in complete clinical response when all lesions have disappeared.
- **Cytology and histology:** Cytology and histology can be used to differentiate between PR and CR in rare cases (i.e., after treatment to differentiate between residual benign lesions and residual malignant lesions in tumor types such as germ cell tumors). Cytologic

confirmation of neoplastic nature of any effusion that appears or worsens during treatment is required when the measurable tumor has met the criteria for response or stable disease. Under such circumstances, the cytologic examination of the fluid collected will permit differentiation between response and stable disease (an effusion may be a side effect of the treatment) or progressive disease (if the neoplastic origin of the fluid is confirmed).

- **Clinical examination:** Clinical lesions will only be considered measurable when they are superficial (i.e., skin nodules and palpable lymph nodes). For the case of skin lesions, documentation by color photography, including a ruler to estimate the size of the lesion, is recommended.

14.1.2.3 Baseline documentation of target and non-target lesions

For the evaluation of lesions at baseline and throughout the study, the lesions are classified at baseline as either target or non-target lesions:

- Target lesions: All measurable lesions (nodal and non-nodal) up to a maximum of five lesions in total (and a maximum of two lesions per organ), representative of all involved organs should be identified as target lesions and recorded and measured at baseline. Target lesions should be selected on the basis of their size (lesions with the longest diameter) and their suitability for accurate repeated measurements (either by imaging techniques or clinically). Each target lesion must be uniquely and sequentially numbered on the CRF (even if it resides in the same organ).

Minimum target lesion size at baseline

- **Non-nodal target:** Non-nodal target lesions identified by methods for which slice thickness is not applicable (e.g., clinical examination, photography) should be at least 10 mm in longest diameter. See Section 14.1.2.1.1.
- **Nodal target:** See Section 14.1.2.1.1.

A sum of diameters (long axis for non-nodal lesions, short axis for nodal) for all target lesions will be calculated and reported as the baseline sum of diameters (SOD). The baseline sum of diameters will be used as reference by which to characterize the objective tumor response. Each target lesion identified at baseline must be followed at each subsequent evaluation and documented on eCRF.

- **Non-target lesions:** All other lesions are considered non-target lesions, i.e. lesions not fulfilling the criteria for target lesions at baseline. Presence or absence or worsening of non-target lesions should be assessed throughout the study; measurements of these lesions are not required. Multiple non-target lesions involved in the same organ can be assessed as a group and recorded as a single item (i.e. multiple liver metastases). Each non-target lesion identified at baseline must be followed at each subsequent evaluation and documented on eCRF.

14.1.2.4 Follow-up evaluation of target and non-target lesions

To assess tumor response, the sum of diameters for all target lesions will be calculated (at baseline and throughout the study). At each assessment response is evaluated first separately for the target (Table 14-1) and non-target lesions (Table 14-2) identified at baseline. These

evaluations are then used to calculate the overall lesion response considering both the target and non-target lesions together (Table 14-3) as well as the presence or absence of new lesions.

14.1.2.4.1 Follow-up and recording of lesions

At each visit and for each lesion the actual date of the scan or procedure which was used for the evaluation of each specific lesion should be recorded. This applies to target and non-target lesions as well as new lesions that are detected. At the assessment visit all of the separate lesion evaluation data are examined by the investigator in order to derive the overall visit response. Therefore all such data applicable to a particular visit should be associated with the same assessment number.

Non-nodal lesions

Following treatment, lesions may have longest diameter measurements smaller than the image reconstruction interval. Lesions smaller than twice the reconstruction interval are subject to substantial “partial volume” effects (i.e., size may be underestimated because of the distance of the cut from the longest diameter; such lesions may appear to have responded or progressed on subsequent examinations, when, in fact, they remain the same size).

If the lesion has completely disappeared, the lesion size should be reported as 0 mm.

Measurements of non-nodal target lesions that become 5 mm or less in longest diameter are likely to be non-reproducible. Therefore, it is recommended to report a default value of 5 mm, instead of the actual measurement. This default value is derived from the 5 mm CT slice thickness (but should not be changed with varying CT slice thickness). Actual measurement should be given for all lesions larger than 5 mm in longest diameter irrespective of slice thickness/reconstruction interval.

In other cases where the lesion cannot be reliably measured for reasons other than its size (e.g., borders of the lesion are confounded by neighboring anatomical structures), no measurement should be entered and the lesion cannot be evaluated.

Nodal lesions

A nodal lesion less than 10 mm in size by short axis is considered normal. Lymph nodes are not expected to disappear completely, so a “non-zero size” will always persist.

Measurements of nodal target lesions that become 5 mm or less in short axis are likely to be non-reproducible. Therefore, it is recommended to report a default value of 5 mm, instead of the actual measurement. This default value is derived from the 5 mm CT slice thickness (but should not be changed with varying CT slice thickness).Actual measurement should be given for all lesions larger than 5 mm in short axis irrespective of slice thickness/reconstruction interval.

However, once a target nodal lesion shrinks to less than 10 mm in its short axis, it will be considered normal for response purpose determination. The lymph node measurements will continue to be recorded to allow the values to be included in the sum of diameters for target lesions, which may be required subsequently for response determination.

14.1.2.4.2 Determination of target lesion response

Table 14-1 Response criteria for target lesions

Response Criteria	Evaluation of target lesions
Complete Response (CR):	Disappearance of all non-nodal target lesions. In addition, any pathological lymph nodes assigned as target lesions must have a reduction in short axis to < 10 mm.[1]
Partial Response (PR):	At least a 30% decrease in the sum of diameter of all target lesions, taking as reference the baseline sum of diameters.
Progressive Disease (PD):	At least a 20% increase in the sum of diameter of all measured target lesions, taking as reference the smallest sum of diameter of all target lesions recorded at or after baseline (nadir). In addition to the relative increase of 20%, the sum must also demonstrate an absolute increase of at least 5 mm.[2]
Stable Disease (SD):	Neither sufficient shrinkage to qualify for PR or CR nor an increase in lesions which would qualify for PD.
Unknown (UNK)	Progression has not been documented and one or more target lesions have not been assessed or have been assessed using a different method than baseline.[3]

1. SOD for CR may not be zero when nodal lesions are part of target lesions
Following an initial CR, a PD cannot be assigned if all non-nodal target lesions are still not present and all nodal lesions are <10 mm in size. In this case, the target lesion response is CR
In exceptional circumstances an UNK response due to change in method could be over-ruled by the investigator or central reviewer using expert judgment based on the available information (see Notes on target lesion response and methodology change in Section 14.1.2.2).

Notes on target lesion response

Reappearance of lesions: If the lesion appears at the same anatomical location where a target lesion had previously disappeared, it is advised that the time point of lesion disappearance (i.e., the “0 mm” recording) be re-evaluated to make sure that the lesion was not actually present and/or not visualized for technical reasons in this previous assessment. If it is not possible to change the 0 value, then the investigator/radiologist has to decide between the following possibilities:

- The lesion is a new lesion, in which case the overall tumor assessment will be considered as progressive disease
- The lesion is clearly a reappearance of a previously disappeared lesion, in which case the size of the lesion has to be entered in the eCRF and the tumor assessment will remain based on the sum of tumor measurements as presented in Table 14-1 above (i.e., a PD will be determined if there is at least 20% increase in the sum of diameters of **all** measured target lesions, taking as reference the smallest sum of diameters of all target lesions recorded at or after baseline with at least 5 mm increase in the absolute sum of the diameters). Proper documentation should be available to support this decision. This applies to patients who have not achieved target response of CR. For patients who have achieved CR, please refer to last bullet in this section.
- For those patients who have only one target lesion at baseline, the reappearance of the target lesion which disappeared previously, even if still small, is considered a PD.
- Missing measurements: In cases where measurements are missing for one or more target lesions it is sometimes still possible to assign PD based on the measurements of the remaining lesions. For example, if the sum of diameters for 5 target lesions at baseline is 100 mm at baseline and the sum of diameters for 3 of those lesions at a post-baseline visit

is 140 mm (with data for 2 other lesions missing) then a PD should be assigned. However, in other cases where a PD cannot definitely be attributed, the target lesion response would be UNK.

- Nodal lesion decrease to normal size: When nodal disease is included in the sum of target lesions and the nodes decrease to "normal" size they should still have a measurement recorded on scans. This measurement should be reported even when the nodes are normal in order not to overstate progression should it be based on increase in the size of nodes.
- Lesions split: In some circumstances, disease that is measurable as a target lesion at baseline and appears to be one mass can split to become two or more smaller sub-lesions. When this occurs, the diameters (long axis - non-nodal lesion, short axis - nodal lesions) of the two split lesions should be added together and the sum recorded in the diameter field on the case report form under the original lesion number. This value will be included in the sum of diameters when deriving target lesion response. The individual split lesions will not be considered as new lesions, and will not automatically trigger a PD designation.
- Lesions coalesced: Conversely, it is also possible that two or more lesions which were distinctly separate at baseline become confluent at subsequent visits. When this occurs a plane between the original lesions may be maintained that would aid in obtaining diameter measurements of each individual lesion. If the lesions have truly coalesced such that they are no longer separable, the maximal diameters (long axis - non-nodal lesion, short axis - nodal lesions) of the "merged lesion" should be used when calculating the sum of diameters for target lesions. On the case report form, the diameter of the "merged lesion" should be recorded for the size of one of the original lesions while a size of "0"mm should be entered for the remaining lesion numbers which have coalesced.
- The **measurements for nodal lesions**, even if less than 10 mm in size, will contribute to the calculation of target lesion response in the usual way with slight modifications.
- Since lesions less than 10 mm are considered normal, a CR for target lesion response should be assigned when all nodal target lesions shrink to less than 10 mm and all non-nodal target lesions have disappeared.
- Once a CR target lesion response has been assigned a CR will continue to be appropriate (in the absence of missing data) until progression of target lesions.
- Following a CR, a PD can subsequently only be assigned for target lesion response if either a non-nodal target lesion "reappears" or if any single nodal lesion is at least 10 mm and there is at least 20% increase in sum of the diameters of all nodal target lesions relative to nadir with at least 5 mm increase in the absolute sum of the diameters.
- A change in method for the evaluation of one or more lesions will usually lead to an UNK target lesion response unless there is progression indicated by the remaining lesions which have been evaluated by the same method. In exceptional circumstances an investigator or central reviewer might over-rule this assignment to put a non-UNK response using expert judgment based on the available information. E.g., a change to a more sensitive method might indicate some tumor shrinkage of target lesions and definitely rule out progression in which case the investigator might assign an SD target lesion response; however, this should be done with caution and conservatively as the response categories have well defined criteria.

14.1.2.4.3 Determination of non-target lesion response

Table 14-2 Response criteria for non-target lesions

Response Criteria	Evaluation of non-target lesions
Complete Response (CR):	Disappearance of all non-target lesions. In addition, all lymph nodes assigned a non-target lesions must be non-pathological in size (< 10 mm short axis)
Progressive Disease (PD):	Unequivocal progression of existing non-target lesions.[1]
Non-CR/Non-PD:	Neither CR nor PD
Unknown (UNK)	Progression has not been documented and one or more non-target lesions have not been assessed or have been assessed using a different method than baseline[2.]

1. The assignment of PD solely based on change in non-target lesions in light of target lesion response of CR, PR or SD should be exceptional. In such circumstances, the opinion of the investigator or central reviewer does prevail.
2. It is recommended that the investigator and/or central reviewer should use expert judgment to assign a Non-UNK response wherever possible (see notes section for more details)

Notes on non-target lesion response

- The investigator and/or central reviewer can use expert judgment to assign a non-UNK response wherever possible, even where lesions have not been fully assessed or a different method has been used. In many of these situations it may still be possible to identify equivocal progression (PD) or definitively rule this out (non-CR/Non-PD) based on the available information. In the specific case where a more sensitive method has been used indicating the absence of any non-target lesions, a CR response can also be assigned.
- The response for non-target lesions is **CR** only if all non-target non-nodal lesions which were evaluated at baseline are now all absent and with all non-target nodal lesions returned to normal size (i.e. < 10 mm). If any of the non-target lesions are still present, or there are any abnormal nodal lesions (i.e. ≥ 10 mm) the response can only be '**Non-CR/Non-PD**' unless there is unequivocal progression of the non-target lesions (in which case response is **PD**) or it is not possible to determine whether there is unequivocal progression (in which case response is UNK).
- Unequivocal progression: To achieve "unequivocal progression" on the basis of non-target disease there must be an overall level of substantial worsening in non-target disease such that, even in presence of CR, PR or SD in target disease, the overall tumor burden has increased sufficiently to merit discontinuation of therapy. A modest "increase" in the size of one or more non-target lesions is usually not sufficient to qualify for unequivocal progression status. The designation of overall progression solely on the basis of change in non-target disease in the face of CR, PR or SD of target disease is therefore expected to be rare. In order for a PD to be assigned on the basis of non-target lesions, the increase in the extent of the disease must be substantial even in cases where there is no measurable disease at baseline. If there is unequivocal progression of non-target lesion(s), then at least one of the non-target lesions must be assigned a status of "Worsened". Where possible, similar rules to those described in Section 14.1.2.4.2 for assigning PD following a CR for the non-target lesion response in the presence of non-target lesions nodal lesions should be applied.

14.1.2.4.4 New lesions

The appearance of a new lesion is always associated with Progressive Disease (PD) and has to be recorded as a new lesion in the New Lesion eCRF page.

- If a new lesion is **equivocal**, for example because of its small size, continued therapy and follow-up evaluation will clarify if it represents truly new disease. If repeat scans confirm there is definitely a new lesion, then progression should be declared using the date of the first observation of the lesion
- If new disease is observed in a region which was **not scanned at baseline** or where the particular baseline scan is not available for some reason, then this should be considered as a PD. The one exception to this is when there are no baseline scans at all available for a patient in which case the response should be UNK, as for any of this patient's assessment (see Section 14.1.2.5).
- A **lymph node is considered as a "new lesion"** and, therefore, indicative of progressive disease if the short axis increases in size to ≥ 10 mm for the first time in the study plus 5 mm absolute increase.
  FDG-PET: can complement CT scans in assessing progression (particularly possible for 'new' disease). See Section 14.1.2.2.

14.1.2.5 Evaluation of overall lesion response

The evaluation of overall lesion response at each assessment is a composite of the target lesion response, non-target lesion response and presence of new lesions as shown below in Table 14-3.

Table 14-3 Overall lesion response at each assessment

Target lesions	Non-target lesions	New Lesions	Overall lesion response
CR	CR	No	CR[1]
CR	Non-CR/Non-PD[3]	No	PR
CR, PR, SD	UNK	No	UNK
PR	Non-PD and not UNK	No	PR[1]
SD	Non-PD and not UNK	No	SD[1, 2]
UNK	Non-PD or UNK	No	UNK[1]
PD	Any	Yes or No	PD
Any	PD	Yes or No	PD
Any	Any	Yes	PD

1. This overall lesion response also applies when there are no non-target lesions identified at baseline.
2. Once confirmed PR was achieved, all these assessments are considered PR.
3. As defined in Section 14.1.2.4.

If there are no baseline scans available at all, then the overall lesion response at each assessment should be considered Unknown (UNK).

In some circumstances it may be difficult to distinguish residual disease from normal tissue. When the evaluation of complete response depends on this determination, it is recommended that the residual lesion be investigated (fine needle aspirate/biopsy) to confirm the CR.

14.1.3 Efficacy definitions

The following definitions primarily relate to patients who have measurable disease at baseline. Section 14.1.3.2.8 outlines the special considerations that need to be given to patients with no measurable disease at baseline in order to apply the same concepts.

14.1.3.1 Best overall response

The best overall response is the best response recorded from the start of the treatment until disease progression/recurrence (taking as reference for PD the smallest measurements recorded since the treatment started). In general, the patient's best response assignment will depend on the achievement of both measurement and confirmation criteria.

The best overall response will usually be determined from response assessments undertaken while on treatment. However, if any assessments occur after treatment withdrawal the protocol should specifically describe if these will be included in the determination of best overall response and/or whether these additional assessments will be required for sensitivity or supportive analyses. As a default, any assessments taken more than 30 days after the last dose of study treatment will not be included in the best overall response derivation. If any alternative cancer therapy is taken while on study any subsequent assessments would ordinarily be excluded from the best overall response determination. If response assessments taken after withdrawal from study treatment and/or alternative therapy are to be included in the main endpoint determination, then this should be described and justified in the protocol.

Where a study requires confirmation of response (PR or CR), changes in tumor measurements must be confirmed by repeat assessments that should be performed not less than 4 weeks after the criteria for response are first met.

Longer intervals may also be appropriate. However, this must be clearly stated in the protocol. The main goal of confirmation of objective response is to avoid overestimating the response rate observed. In cases where confirmation of response is not feasible, it should be made clear when reporting the outcome of such studies that the responses are not confirmed.

- For non-randomized trials where response is the primary endpoint, confirmation is needed.
- For trials intended to support accelerated approval, confirmation is needed
- For all other trials, confirmation of response may be considered optional.

The best overall response for each patient is determined from the sequence of overall (lesion) responses according to the following rules:

- CR = at least two determinations of CR at least 4 weeks apart before progression where confirmation required or one determination of CR prior to progression where confirmation not required
- PR = at least two determinations of PR or better at least 4 weeks apart before progression (and not qualifying for a CR) where confirmation required or one determination of PR prior to progression where confirmation not required
- SD = at least one SD assessment (or better) > 12 weeks after randomization/start of treatment (and not qualifying for CR or PR).
- PD = progression ≤ 12 weeks after randomization/ start of treatment (and not qualifying for CR, PR or SD).

- UNK = all other cases (i.e. not qualifying for confirmed CR or PR and without SD after more than 12 weeks or early progression within the first 12 weeks)

The time durations specified in the SD/PD/UNK definitions above are defaults based on a 8 week tumor assessment frequency. However these may be modified for specific indications which are more or less aggressive. In addition, it is envisaged that the time duration may also take into account assessment windows. E.g., if the assessment occurs every 6 weeks with a time window of ± 7 days, a BOR of SD would require a SD or better response longer than 5 weeks after randomization/start of treatment.

Overall lesion responses of CR must stay the same until progression sets in, with the exception of a UNK status. A patient who had a CR cannot subsequently have a lower status other than a PD, e.g., PR or SD, as this would imply a progression based on one or more lesions reappearing, in which case the status would become a PD.

Once an overall lesion response of PR is observed (which may have to be a confirmed PR depending on the study) this assignment must stay the same or improve over time until progression sets in, with the exception of an UNK status. However, in studies where confirmation of response is required, if a patient has a single PR (≥30% reduction of tumor burden compared to baseline) at one assessment, followed by a <30% reduction from baseline at the next assessment (but not ≥20% increase from previous smallest sum), the objective status at that assessment should be SD. Once a confirmed PR was seen, the overall lesion response should be considered PR (or UNK) until progression is documented or the lesions totally disappear in which case a CR assignment is applicable. In studies where confirmation of response is not required after a single PR the overall lesion response should still be considered PR (or UNK) until progression is documented or the lesion totally disappears in which case a CR assignment is applicable.

Example: In a case where confirmation of response is required the sum of lesion diameters is 200 mm at baseline and then 140 mm - 150 mm - 140 mm - 160 mm - 160 mm at the subsequent visits. Assuming that non-target lesions did not progress, the overall lesion response would be PR - SD - PR - PR - PR. The second assessment with 140 mm confirms the PR for this patient. All subsequent assessments are considered PR even if tumor measurements decrease only by 20% compared to baseline (200 mm to 160 mm) at the following assessments.

If the patient progressed but continues study treatment, further assessments are not considered for the determination of best overall response.

Note: these cases may be described as a separate finding in the CSR but not included in the overall response or disease control rates.

The best overall response for a patient is always calculated, based on the sequence of overall lesion responses. However, the overall lesion response at a given assessment may be provided from different sources:

- Investigator overall lesion response
- Central Blinded Review overall lesion response
- Novartis calculated overall lesion response (based on measurements from either Investigator or Central Review)

The primary analysis of the best overall response will be based on the sequence of investigator/central blinded review/calculated (investigator)/calculated (central) overall lesion responses.

Based on the patients' best overall response during the study, the following rates are then calculated:

Overall response rate (ORR) is the proportion of patients with a best overall response of CR or PR. This is also referred to as 'Objective response rate' in some protocols or publications.

Disease control rate (DCR) is the proportion of patients with a best overall response of CR or PR or SD. The objective of this endpoint is to summarize patients with signs of "activity" defined as either shrinkage of tumor (regardless of duration) or slowing down of tumor growth.

Clinical benefit rate (CBR) is the proportion of patients with a best overall response of CR or PR, or an overall lesion response of SD or Non-CR/Non-PD which lasts for a minimum time duration (with a default of at least 24 weeks in breast cancer studies). This endpoint measures signs of activity taking into account duration of disease stabilization.

Another approach is to summarize the progression rate at a certain time point after baseline. In this case, the following definition is used:

Early progression rate (EPR) is the proportion of patients with progressive disease within 8 weeks of the start of treatment.

The protocol should define populations for which these will be calculated. The timepoint for EPR is study specific. EPR is used for the multinomial designs of Dent and Zee (2001) and counts all patients who at the specified assessment (in this example the assessment would be at 8 weeks ± window) do not have an overall lesion response of SD, PR or CR. Patients with an unknown (UNK) assessment at that time point and no PD before, will not be counted as early progressors in the analysis but may be included in the denominator of the EPR rate, depending on the analysis population used. Similarly when examining overall response and disease control, patients with a best overall response assessment of unknown (UNK) will not be regarded as "responders" but may be included in the denominator for ORR and DCR calculation depending on the analysis population (e.g., populations based on an ITT approach).

14.1.3.2 Time to event variables

The protocol should state which of the following variables is used in that study.

14.1.3.2.1 Progression-free survival

Usually in all Oncology studies, patients are followed for tumor progression after discontinuation of study medication for reasons other than progression or death. If this is not used, e.g., in Phase I or II studies, this should be clearly stated in the protocol. Note that randomized trials (preferably blinded) are recommended where PFS is to be the primary endpoint.

Progression-free survival (PFS) is the time from date of randomization/start of treatment to the date of event defined as the first documented progression or death due to any cause. If a

patient has not had an event, progression-free survival is censored at the date of last adequate tumor assessment.

PFS rate at x weeks is an additional measure used to quantify PFS endpoint. It is recommended that a Kaplan Meier estimate is used to assess this endpoint.

14.1.3.2.2 Overall survival

All patients should be followed until death or until patient has had adequate follow-up time as specified in the protocol whichever comes first. The follow-up data should contain the date the patient was last seen alive / last known date patient alive, the date of death and the reason of death (“Study indication” or “Other”).

Overall survival (OS) is defined as the time from date of randomization/start of treatment to date of death due to any cause. If a patient is not known to have died, survival will be censored at the date of last known date patient alive.

14.1.3.2.3 Time to progression

Some studies might consider only death related to underlying cancer as an event which indicates progression. In this case the variable “Time to progression” might be used. TTP is defined as PFS except for death unrelated to underlying cancer.

Time to progression (TTP) is the time from date of randomization/start of treatment to the date of event defined as the first documented progression or death due to underlying cancer. If a patient has not had an event, time to progression is censored at the date of last adequate tumor assessment.

14.1.3.2.4 Time to treatment failure

This endpoint is often appropriate in studies of advanced disease where early discontinuation is typically related to intolerance of the study drug. In some protocols, time to treatment failure may be considered as a sensitivity analysis for time to progression. The list of discontinuation reasons to be considered or not as treatment failure may be adapted according to the specificities of the study or the disease.

Time to treatment failure (TTF) is the time from date of randomization/start of treatment to the earliest of date of progression, date of death due to any cause, or date of discontinuation due to reasons other than ‘Protocol violation’ or ‘Administrative problems’. The time to treatment failure for patients who did not experience treatment failure will be censored at last adequate tumor assessment.

14.1.3.2.5 Duration of response

The analysis of the following variables should be performed with much caution when restricted to responders since treatment bias could have been introduced. There have been reports where a treatment with a significantly higher response rate had a significantly shorter duration of response but where this probably primarily reflected selection bias which is explained as follows: It is postulated that there are two groups of patients: a good risk group and a poor risk group. Good risk patients tend to get into response readily (and relatively quickly) and tend to remain in response after they have a response. Poor risk patients tend to be difficult to achieve

a response, may have a longer time to respond, and tend to relapse quickly when they do respond. Potent agents induce a response in both good risk and poor risk patients. Less potent agents induce a response mainly in good risk patients only. This is described in more detail by Morgan (1988).

It is recommended that an analysis of all patients (both responders and non-responders) be performed whether or not a "responders only" descriptive analysis is presented. An analysis of responders should only be performed to provide descriptive statistics and even then interpreted with caution by evaluating the results in the context of the observed response rates… If an inferential comparison between treatments is required this should only be performed on all patients (i.e. not restricting to "responders" only) using appropriate statistical methods such as the techniques described in Ellis et al (2008). It should also be stated in the protocol if duration of response is to be calculated in addition for unconfirmed response.

For summary statistics on "responders" only the following definitions are appropriate. (Specific definitions for an all-patient analysis of these endpoints are not appropriate since the status of patients throughout the study is usually taken into account in the analysis).

Duration of overall response (CR or PR): For patients with a CR or PR (which may have to be confirmed the start date is the date of first documented response (CR or PR) and the end date and censoring is defined the same as that for time to progression.

The following two durations might be calculated in addition for a large Phase III study in which a reasonable number of responders is seen.

Duration of overall complete response (CR): For patients with a CR (which may have to be confirmed) the start date is the date of first documented CR and the end date and censoring is defined the same as that for time to progression.

Duration of stable disease (CR/PR/SD): For patients with a CR or PR (which may have to be confirmed) or SD the start and end date as well as censoring is defined the same as that for time to progression.

14.1.3.2.6 Time to response

Time to overall response (CR or PR) is the time between date of randomization/start of treatment until first documented response (CR or PR). The response may need to be confirmed depending on the type of study and its importance. Where the response needs to be confirmed then time to response is the time to the first CR or PR observed.

Although an analysis on the full population is preferred a descriptive analysis may be performed on the "responders" subset only, in which case the results should be interpreted with caution and in the context of the overall response rates, since the same kind of selection bias may be introduced as described for duration of response in Section 14.1.3.2.4. It is recommended that an analysis of all patients (both responders and non-responders) be performed whether or not a "responders only" descriptive analysis is presented. Where an inferential statistical comparison is required, then all patients should definitely be included in the analysis to ensure the statistical test is valid. For analysis including all patients, patients who did not achieve a response (which may have to be a confirmed response) will be censored using one of the following options.

- at maximum follow-up (i.e. FPFV to LPLV used for the analysis) for patients who had a PFS event (i.e. progressed or died due to any cause). In this case the PFS event is the worst possible outcome as it means the patient cannot subsequently respond. Since the statistical analysis usually makes use of the ranking of times to response it is sufficient to assign the worst possible censoring time which could be observed in the study which is equal to the maximum follow-up time (i.e. time from FPFV to LPLV)
- at last adequate tumor assessment date otherwise. In this case patients have not yet progressed so they theoretically still have a chance of responding

Time to overall complete response (CR) is the time between dates of randomization/start of treatment until first documented CR. Similar analysis considerations including (if appropriate) censoring rules apply for this endpoint described for the time to overall response endpoint.

14.1.3.2.7 Definition of start and end dates for time to event variables

Assessment date

For each assessment (i.e. evaluation number), the **assessment date** is calculated as the latest of all measurement dates (e.g., X-ray, CT-scan) if the overall lesion response at that assessment is CR/PR/SD/UNK. Otherwise - if overall lesion response is progression - the assessment date is calculated as the earliest date of all measurement dates at that evaluation number.

In the calculation of the assessment date for time to event variables, any unscheduled assessment should be treated similarly to other evaluations.

Start dates

For all “time to event” variables, other than duration of response, the randomization/ date of treatment start will be used as the start date.

For the calculation of duration of response the following start date should be used:

- Date of first documented response is the assessment date of the first overall lesion response of CR (for duration of overall complete response) or CR / PR (for duration of overall response) respectively, when this status is later confirmed.

End dates

The end dates which are used to calculate ‘time to event’ variables are defined as follows:

- Date of death (during treatment as recorded on the treatment completion page or during follow-up as recorded on the study evaluation completion page or the survival follow-up page).
- Date of progression is the first assessment date at which the overall lesion response was recorded as progressive disease.
- Date of last adequate tumor assessment is the date the last tumor assessment with overall lesion response of CR, PR or SD which was made before an event or a censoring reason occurred. In this case the last tumor evaluation date at that assessment is used. If no post-baseline assessments are available (before an event or a censoring reason occurred) the date of randomization/start of treatment is used.

- Date of next scheduled assessment is the date of the last adequate tumor assessment plus the protocol specified time interval for assessments. This date may be used if back-dating is considered when the event occurred beyond the acceptable time window for the next tumor assessment as per protocol (see Section 14.1.3.2.7).

Example (if protocol defined schedule of assessments is 3 months): tumor assessments at baseline - 3 months - 6 months - missing - missing - PD. Date of next scheduled assessment would then correspond to 9 months.

- Date of discontinuation is the date of the end of treatment visit.
- Date of last contact is defined as the last date the patient was known to be alive. This corresponds to the latest date for either the visit date, lab sample date or tumor assessment date. If available, the last known date patient alive from the survival follow-up page is used. If no survival follow-up is available, the date of discontinuation is used as last contact date.
- Date of secondary anti-cancer therapy is defined as the start date of any additional (secondary) antineoplastic therapy or surgery.

14.1.3.2.8 Sensitivity analyses

This section outlines the possible event and censoring dates for progression, as well as addresses the issues of missing tumor assessments during the study. For instance, if one or more assessment visits are missed prior to the progression event, to what date should the progression event be assigned? And should progression event be ignored if it occurred after a long period of a patient being lost to follow-up? It is important that the protocol and RAP specify the primary analysis in detail with respect to the definition of event and censoring dates and also include a description of one or more sensitivity analyses to be performed.

Based on definitions outlined in Section 14.1.3.2.6, and using the draft FDA guideline on endpoints (Clinical Trial Endpoints for the Approval of Cancer Drugs and Biologics April 2005) as a reference, the following analyses can be considered:

Table 14-4 Options for event dates used in PFS, TTP, duration of response

Situation		Options for end-date (progression or censoring)[1]<br>(1) = default unless specified differently in the protocol or RAP	Outcome
A	No baseline assessment	(1) Date of randomization/start of treatment[3]	Censored
B	Progression at or before next scheduled assessment	(1) Date of progression<br>(2) Date of next scheduled assessment[2]	Progressed<br>Progressed
C1	Progression or death after **exactly one** missing assessment	(1) Date of progression (or death)<br>(2) Date of next scheduled assessment[2]	Progressed<br>Progressed
C2	Progression or death after **two or more** missing assessments	(1) Date of last adequate assessment[2]<br>(2) Date of next scheduled assessment[2]<br>(3) Date of progression (or death)	Censored<br>Progressed<br>Progressed
D	No progression	(1) Date of last adequate assessment	Censored
E	Treatment discontinuation due to 'Disease progression' without documented progression, i.e. clinical progression based on investigator claim	(1) Ignore clinical progression and follow situations above<br>(2) Date of discontinuation (visit date at which clinical progression was determined)	As per above situations<br>Progressed
F	New anticancer therapy given	(1) Ignore the new anticancer therapy and follow situations above (ITT approach)<br>(2) Date of last adequate assessment prior to new anticancer therapy<br>(3) Date of secondary anti-cancer therapy<br>(4) Date of secondary anti-cancer therapy	As per above situations<br>Censored<br>Censored<br>Event
G	Deaths due to reason other than deterioration of 'Study indication'	(1) Date of last adequate assessment	Censored (only TTP and duration of response)

1. =Definitions can be found in Section 14.1.3.2.6.
2. =After the last adequate tumor assessment. "Date of next scheduled assessment" is defined in Section 14.1.3.2.6.
3. =The rare exception to this is if the patient dies no later than the time of the second scheduled assessment as defined in the protocol in which case this is a PFS event at the date of death.

The primary analysis and the sensitivity analyses must be specified in the protocol. Clearly define if and why options (1) are not used for situations C, E and (if applicable) F.

Situations C (C1 and C2): Progression or death after one or more missing assessments: The primary analysis is usually using options (1) for situations C1 and C2, i.e.

- (C1) taking the actual progression or death date, in the case of only one missing assessment.
- (C2) censoring at the date of the last adequate assessment, in the case of two or more consecutive missing assessments.

In the case of two or missing assessments (situation C2), option (3) may be considered jointly with option (1) in situation C1 as sensitivity analysis. A variant of this sensitivity analysis consists of backdating the date of event to the next scheduled assessment as proposed with option (2) in situations C1 and C2.

Situation E: Treatment discontinuation due to 'Disease progression' without documented progression: By default, option (1) is used for situation E as patients without documented PD should be followed for progression after discontinuation of treatment. However, option (2) may be used as sensitivity analysis. If progression is claimed based on clinical deterioration instead of tumor assessment by e.g., CT-scan, option (2) may be used for indications with high early progression rate or difficulties to assess the tumor due to clinical deterioration.

Situation F: New cancer therapy given: the handling of this situation must be specified in detail in the protocol. However, option (1) (ITT) is the recommended approach; events documented after the initiation of new cancer therapy will be considered for the primary analysis i.e. progressions and deaths documented after the initiation of new cancer therapy would be included as events. This will require continued follow-up for progression after the start of the new cancer therapy. In such cases, it is recommended that an additional sensitivity analysis be performed by censoring at last adequate assessment prior to initiation of new cancer therapy.

Option (2), i.e. censoring at last adequate assessment may be used as a sensitivity analysis. If a high censoring rate due to start of new cancer therapy is expected, a window of approximately 8 weeks performed after the start of new cancer therapy can be used to calculate the date of the event or censoring. This should be clearly specified in the SAP.

In some specific settings, local treatments (e.g., radiation/surgery) may not be considered as cancer therapies for assessment of event/censoring in PFS/TTP/DoR analysis. For example, palliative radiotherapy given in the trial for analgesic purposes or for lytic lesions at risk of fracture will not be considered as cancer therapy for the assessment of BOR and PFS analyses. The protocol should clearly state the local treatments which are not considered as antineoplastic therapies in the PFS/TTP/DoR analysis.

The protocol should state that tumor assessments will be performed every x weeks until radiological progression irrespective of initiation of new antineoplastic therapy. It is strongly recommended that a tumor assessment is performed before the patient is switched to a new cancer therapy.

Additional suggestions for sensitivity analyses

Other suggestions for additional sensitivity analyses may include analyses to check for potential bias in follow-up schedules for tumor assessments, e.g., by assigning the dates for censoring and events only at scheduled visit dates. The latter could be handled by replacing in Table 14-4 the "Date of last adequate assessment" by the "Date of previous scheduled assessment (from baseline)", with the following definition:

- **Date of previous scheduled assessment (from baseline)** is the date when a tumor assessment would have taken place, if the protocol assessment scheme was strictly followed from baseline, immediately before or on the date of the last adequate tumor assessment.

In addition, analyses could be repeated using the Investigators' assessments of response rather than the calculated response. The need for these types of sensitivity analyses will depend on the individual requirements for the specific study and disease area and have to be specified in the protocol or RAP documentation.

14.1.4 Data handling and programming rules

The following section should be used as guidance for development of the protocol, data handling procedures or programming requirements (e.g., on incomplete dates).

14.1.4.1 Study/project specific decisions

For each study (or project) various issues need to be addressed and specified in the protocol or RAP documentation. Any deviations from protocol must be discussed and defined at the latest in the RAP documentation.

The proposed primary analysis and potential sensitivity analyses should be discussed and agreed with the health authorities and documented in the protocol (or at the latest in the RAP documentation before database lock).

14.1.4.2 End of treatment phase completion

Patients **may** voluntarily withdraw from the study treatment or may be taken off the study treatment at the discretion of the investigator at any time. For patients who are lost to follow-up, the investigator or designee should show "due diligence" by documenting in the source documents steps taken to contact the patient, e.g., dates of telephone calls, registered letters, etc.

The end of treatment visit and its associated assessments should occur within 7 days of the last study treatment.

Patients may discontinue study treatment for any of the following reasons:

- Adverse event(s)
- Lost to follow-up
- Physician decision
- Pregnancy
- Protocol deviation
- Technical problems
- Subject/guardian decision
- Progressive disease
- Study terminated by the sponsor
- Non-compliant with study treatment
- No longer requires treatment
- Treatment duration completed as per protocol (optional, to be used if only a fixed number of cycles is given)

Death is a reason which "**must**" lead to discontinuation of patient from trial.

14.1.4.3 End of post-treatment follow-up (study phase completion)

End of post-treatment follow-up visit will be completed after discontinuation of study treatment and post-treatment evaluations but prior to collecting survival follow-up.

Patients may provide study phase completion information for one of the following reasons:

- Adverse event
- Lost to follow-up
- Physician decision
- Pregnancy
- Protocol deviation
- Technical problems
- Subject/guardian decision
- Death
- Progressive disease
- Study terminated by the sponsor

14.1.4.4 Medical validation of programmed overall lesion response

In order to be as objective as possible the RECIST programmed calculated response assessment is very strict regarding measurement methods (i.e. any assessment with more or less sensitive method than the one used to assess the lesion at baseline is considered UNK) and not available evaluations (i.e. if any target or non-target lesion was not evaluated the whole overall lesion response is UNK unless remaining lesions qualified for PD). This contrasts with the slightly more flexible guidance given to local investigators (and to the central reviewers) to use expert judgment in determining response in these type of situations, and therefore as a consequence discrepancies between the different sources of response assessment often arise. To ensure the quality of response assessments from the local site and/or the central reviewer, the responses may be re-evaluated by clinicians (based on local investigator data recorded in eCRF or based on central reviewer data entered in the database) at Novartis or external experts. In addition, data review reports will be available to identify assessments for which the investigators' or central reader's opinion does not match the programmed calculated response based on RECIST criteria. This may be queried for clarification. However, the investigator or central reader's response assessment will never be overruled.

If Novartis elect to invalidate an overall lesion response as evaluated by the investigator or central reader upon internal or external review of the data, the calculated overall lesion response at that specific assessment is to be kept in a dataset. This must be clearly documented in the RAP documentation and agreed before database lock. This dataset should be created and stored as part of the 'raw' data.

Any discontinuation due to 'Disease progression' without documentation of progression by RECIST criteria should be carefully reviewed. Only patients with documented deterioration of symptoms indicative of progression of disease should have this reason for discontinuation of treatment or study evaluation.

14.1.4.5 Programming rules

The following should be used for programming of efficacy results:

14.1.4.5.1 Calculation of 'time to event' variables

Time to event = end date - start date + 1 (in days)

When no post-baseline tumor assessments are available, the date of randomization/start of treatment will be used as end date (duration = 1 day) when time is to be censored at last tumor assessment, i.e. time to event variables can never be negative.

14.1.4.5.2 Incomplete assessment dates

All investigation dates (e.g., X-ray, CT scan) must be completed with day, month and year.

If one or more investigation dates are incomplete but other investigation dates are available, this/these incomplete date(s) are not considered for calculation of the assessment date (and assessment date is calculated as outlined in Section 14.1.3.2.6). If all measurement dates have no day recorded, the 1st of the month is used.

If the month is not completed, for any of the investigations, the respective assessment will be considered to be at the date which is exactly between previous and following assessment. If a previous and following assessment is not available, this assessment will not be used for any calculation.

14.1.4.5.3 Incomplete dates for last known date patient alive or death

All dates must be completed with day, month and year. If the day is missing, the 15th of the month will be used for incomplete death dates or dates of last contact.

14.1.4.5.4 Non-target lesion response

If no non-target lesions are identified at baseline (and therefore not followed throughout the study), the non-target lesion response at each assessment will be considered ‘not applicable (NA)’.

14.1.4.5.5 Study/project specific programming

The standard analysis programs need to be adapted for each study/project.

14.1.4.5.6 Censoring reason

In order to summarize the various reasons for censoring, the following categories will be calculated for each time to event variable based on the treatment completion page, the study evaluation completion page and the survival page.

For survival the following censoring reasons are possible:

- Alive
- Lost to follow-up

For PFS and TTP (and therefore duration of responses) the following censoring reasons are possible:

- Ongoing without event
- Lost to follow-up
- Withdrew consent
- Adequate assessment no longer available*
- Event documented after two or more missing tumor assessments (optional, see Table 14-4)

- Death due to reason other than underlying cancer *(only used for TTP and duration of response)*
- Initiation of new anti-cancer therapy

* Adequate assessment is defined in Section 14.1.3.2.6. This reason is applicable when adequate evaluations are missing for a specified period prior to data cut-off (or prior to any other censoring reason) corresponding to the unavailability of two or more planned tumor assessments prior to the cut-off date. The following clarifications concerning this reason should also be noted:

- This may be when there has been a definite decision to stop evaluation (e.g., reason="Sponsor decision" on study evaluation completion page), when patients are not followed for progression after treatment completion or when only UNK assessments are available just prior to data cut-off).
- The reason "Adequate assessment no longer available" also prevails in situations when another censoring reason (e.g., withdrawal of consent, loss to follow-up or alternative anti-cancer therapy) has occurred more than the specified period following the last adequate assessment.
- This reason will also be used to censor in case of no baseline assessment.

14.1.5 References (available upon request)

Dent S, Zee (2001). application of a new multinomial phase II stopping rule using response and early progression, J Clin Oncol; 19: 785-791

Eisenhauer E, et al (2009). New response evaluation criteria in solid tumors: revised RECIST guideline (version 1.1). European Journal of Cancer, Vol.45: 228-47

Ellis S, et al (2008). Analysis of duration of response in oncology trials. Contemp Clin Trials 2008; 29: 456-465

EMA Guidance: 2012 Guideline on the evaluation of anticancer medicinal products in man

FDA Guidelines: 2005 Clinical Trial Endpoints for the Approval of Cancer Drugs and Biologics, April 2005

FDA Guidelines: 2007 Clinical Trial Endpoints for the Approval of Cancer Drugs and Biologics, May 2007

Morgan TM (1988). Analysis of duration of response: a problem of oncology trials. Cont Clin Trials; 9: 11-18

Therasse P, Arbuck S, Eisenhauer E, et al (2000). New Guidelines to Evaluate the Response to Treatment in Solid Tumors, Journal of National Cancer Institute, Vol. 92; 205-16

14.2 Appendix 2: The Bayesian logistic regression model, prior, and dosing regimen recommendations in hypothetical scenarios

Clinical Development

CPDR001F

CPDR001F2301

Protocol post-text supplement: 02
The Bayesian logistic regression model, prior, and dosing regimen recommendations in hypothetical scenarios

Document type: Protocol post-text supplement

Development phase: III

Release date: 29-Sep-2016

Property of Novartis
Confidential
May not be used, divulged, published or otherwise disclosed
without the consent of Novartis

14.2.1 Introduction

This appendix provides details of the statistical model, the derivation of prior distributions from historical data, and the results of the Bayesian analyses and respective dosing decisions for hypothetical data scenarios.

14.2.2 Statistical model

For each of the k dosing regimens (k = 1, 2, 3), a separate statistical model will comprise of single-agent toxicity parts and interaction parts to describe both two-way and three-way drug safety interactions.

- Model 1 will incorporate DLT data from the first 8 weeks of triple combination treatment in cohort 1 (400 mg i.v. PDR001 Q4W + 150 mg BID dabrafenib + 2 mg QD trametinib)
- Model 2 will incorporate DLT data from the first 8 weeks of triple combination treatment in cohort -1a (400 mg i.v. PDR001 Q8W + 150 mg BID dabrafenib + 2 mg QD trametinib)
- Model 3 will incorporate DLT data from the first 8 weeks of triple combination treatment in cohort -1b (4 weeks run-in of 150 mg BID dabrafenib + 2 mg QD trametinib, followed by 400 mg i.v. PDR001 Q4W + 150 mg BID dabrafenib + 2 mg QD trametinib)

14.2.2.1 Single agent parts

Let $\pi_{1k}(d_1)$ be the risk of DLT for dabrafenib given as a single agent BID at dose d_1 for the kth model (k = 1, 2, 3); $\pi_{2k}(d_2)$ be the risk of DLT for trametinib given as a single agent QD at dose d_2 for the kth model; and $\pi_{3k}(d_3)$ be the risk of DLT for PDR001 given as a single agent Q4W at dose d_3 for the kth model. These single agent dose-DLT models are logistic:

$$\text{Dabrafenib:} \quad \text{logit}(\pi_{1k}(d_1)) = \log(\alpha_{1k}) + \beta_{1k}\log(d_1/150)$$

$$\text{Trametinib:} \quad \text{logit}(\pi_{2k}(d_2)) = \log(\alpha_{2k}) + \beta_{2k}\log(d_2/2)$$

$$\text{PDR001:} \quad \text{logit}(\pi_{3k}(d_3)) = \log(\alpha_{3k}) + \beta_{3k}\log(d_3/400)$$

where 150 mg, 2 mg, and 400 mg are used to scale the doses of dabrafenib, trametinib, and PDR001, respectively. Hence, for the kth model, α_{1k}, α_{2k}, and α_{3k} (>0) are the single agent odds of a DLT at 150 mg BID, 2 mg QD, and 400 mg Q4W, respectively; and β_{1k}, β_{2k}, and β_{3k} (>0) are the increase in the log-odds of a DLT by a unit increase in log-dose.

14.2.2.2 Interaction

Under no interaction, the risk of a DLT for the kth model (k = 1, 2, 3) at dose d_1 of dabrafenib, dose d_2 of trametinib, and dose d_3 of PDR001 is:

$$\pi^0_{123k}(d_1, d_2, d_3) = 1 - \left(1 - \pi_{1k}(d_1)\right)\left(1 - \pi_{2k}(d_2)\right)\left(1 - \pi_{3k}(d_3)\right)$$

To allow for interaction between dabrafenib, trametinib, and PDR001, odds multipliers are introduced:

η_{12k}	Two-way interaction between dabrafenib and trametinib
η_{13k}	Two-way interaction between dabrafenib and PDR001
η_{23k}	Two-way interaction between trametinib and PDR001
η_{123k}	Three-way interaction between dabrafenib, trametinib, and PDR001

The risk of DLT for combination dose (d_1, d_2, d_3) is then given by:

$$\begin{aligned} \text{odds}(\pi_{123k}(d_1,d_2,d_3)) \quad = \quad & \exp(\eta_{12k} \times d_1/150 \times d_2/2) \\ \times \quad & \exp(\eta_{13k} \times d_1/150 \times d_3/400) \\ \times \quad & \exp(\eta_{23k} \times d_2/2 \times d_3/400) \\ \times \quad & \exp(\eta_{123k} \times d_1/150 \times d_2/2 \times d_3/400) \\ \times \quad & \text{odds}(\pi^0_{123k}(d_1,d_2,d_3)) \end{aligned}$$

where odds(π) = π / (1 - π); η_{ijk} is the kth model log-odds ratio between the interaction and no interaction model at the reference dose for treatments i and j and a zero dose of the third treatment; and ($\eta_{12k} + \eta_{23k} + \eta_{32k} + \eta_{123k}$) is the kth model log-odds ratio between the interaction and no interaction model, at the reference dose for all three treatments. Here $\eta_{ijk} = 0$ corresponds to no interaction, with $\eta_{ijk} > 0$ and $\eta_{ijk} < 0$ representing synergistic and antagonistic toxicity, respectively.

14.2.3 Prior specifications

The Bayesian approach requires the specification of prior distributions for all model parameters which include, for the kth model (k = 1, 2, 3), the single agent parameters log(α_{1k}) and log(β_{1k}) for dabrafenib, log(α_{2k}) and log(β_{2k}) for trametinib, log(α_{3k}) and log(β_{3k}) for PDR001, and interaction parameters η_{12k}, η_{13k}, η_{23k}, and η_{123k}. A meta-analytic-predictive (MAP) approach is used to derive a prior distribution for the single agent model parameters based upon available DLT data. For dabrafenib and trametinib, 100% MAP priors will be used. For PDR001, this is then robustified by creating a mixture prior including both a component derived from the MAP prior and a weakly informative robustification component. This robustification allows for the possibility that the dose/toxicity relationship for PDR001 in combination differs substantially from that of the single agent.

14.2.3.1 Prior distribution for the logistic parameters

Description of the meta-analytic-predictive (MAP) approach

The aim of the MAP approach is to derive a prior distribution from the logistic parameters (log(α^*), log(β^*)) of the new trial using DLT data from historical studies.

Gp

The corresponding probability of a DLT is π_{ds}. The model specifications for the derivation of the MAP prior are as follows:

$$r_{ds} \mid \pi_{ds} \sim \text{Bin}(\pi_{ds}, n_{ds})$$

$$\text{logit}(\pi_{ds}) = \log(\alpha_s) + \beta_s \log(d/d_{ref})$$

$$(\log(\alpha_s), \log(\beta_s)) \mid \mu, \psi \sim \text{BVN}(\mu, \psi), s = 1,\ldots, S$$

$$(\log(\alpha^*), \log(\beta^*)) \mid \mu, \psi \sim \text{BVN}(\mu, \psi)$$

where d_{ref} is the reference dose. The parameters $\mu = (\mu_1, \mu_2)$ and ψ are the mean and between-trial covariance matrix for the logistic parameters, the latter with standard deviations τ_1 and τ_2 and correlation ρ. The parameters τ_1 and τ_2 quantify the degree of between-trial heterogeneity. The following priors are used for these parameters:

- normal priors for μ_1 and μ_2,
- log-normal priors for τ_1 and τ_2, and
- a uniform prior for ρ

The MAP prior for single agent model parameters in the new trial, $(\log(\alpha^*), \log(\beta^*))$, is the predictive distribution

$$(\log(\alpha^*), \log(\beta^*)) \mid (r_{ds}, n_{ds}: s = 1,\ldots, S)$$

Since the predictive distribution is not available analytically, MCMC is used to simulate values from this distribution. This is implemented using JAGS version 4.1.0.

Single agent dabrafenib:

The prior distributions for the kth model (k = 1, 2, 3) of dabrafenib single agent BLRM model parameters $(\log(\alpha_{1k}), \log(\beta_{1k}))$ are based 100% on MAP.

MAP component for dabrafenib

For the MAP model for dabrafenib, reference dose d_{ref} = 150 mg (BID) is used, and data from S = 1 history study are available.

Weakly informative normal priors are assumed for μ_{11k} and μ_{12k}, with means corresponding to an assumed 10% risk of DLT at the reference dose of 150 mg, and a double in dose leading to a doubling in the odds of a DLT, respectively. Priors for τ_{11k} and τ_{12} are assigned such that (1) their medians correspond to moderate between-trial heterogeneity, and (2) their uncertainty (95% prior interval) cover plausible between-trial standard deviations (Neuenschwander 2014).

The prior distributions for the model used for deriving the MAP priors for dabrafenib are specified in Table 14-5.

Table 14-5 Prior distributions for the parameters of the MAP model used to derive the prior for the single agent dabrafenib model parameters

Parameter	Prior distribution
μ_{11k}	N(mean = logit(0.1), sd = 2)
μ_{12k}	N(mean = logit(0.5), sd=1)
τ_{11k}	log-normal(mean = 0.250, sd = log(2)/1.96)
τ_{12k}	log-normal(mean = 0.125, sd = log(2)/1.96)
ρ_{1k}	uniform(-1,1)

Historical data for dabrafenib

The dose-DLT data of dabrafenib single agent from the following clinical study are considered as relevant information (Table 14-6). Only BID dosing data from this study were used to derive the prior distribution for the BLRM parameters $(\log(\alpha_{1k}), \log(\beta_{1k}))$ for model k (k = 1, 2, 3).

- **BRF112680:** open-label, multi-center, phase I dose-escalation study to investigate the safety, pharmacokinetics, and pharmacodynamics of dabrafenib in subjects with solid tumors or BRAF-mutation positive melanoma

The DLT observation window in the phase I dose escalation part of this trial was 3 weeks.

Table 14-6 Historical data from dabrafenib single agent clinical study

Dose level	Number of evaluable patients	Number of DLTs
35 mg BID	4	0
70 mg BID	4	0
100 mg BID	6	0
150 mg BID	6	0
200 mg BID	7	1
350 mg BID	6	1

Single agent trametenib:

The prior distributions for the kth model (k = 1, 2, 3) of trametinib single agent BLRM model parameters ($\log(\alpha_{2k})$, $\log(\beta_{2k})$) are based 100% on MAP.

MAP component for trametinib

For the MAP model for trametinib, reference dose d_{ref} = 2 mg (QD) is used, and data from S = 1 history study are available.

Weakly informative normal priors are assumed for μ_{21k} and μ_{22k}, with means corresponding to an assumed 10% risk of DLT at the reference dose of 2 mg, and a double in dose leading to a doubling in the odds of a DLT, respectively. Priors for τ_{21k} and τ_{22k} are assigned such that (1) their medians correspond to moderate between-trial heterogeneity, and (2) their uncertainty (95% prior interval) cover plausible between-trial standard deviations.

The prior distributions for the model used for deriving the MAP priors for trametinib are specified in Table 14-7.

Table 14-7 Prior distributions for the parameters of the MAP model used to derive the prior for the single agent trametinib model parameters

Parameter	Prior distribution
μ_{21k}	N(mean = logit(0.1), sd = 2)
μ_{22k}	N(mean = logit(0.5), sd=1)
τ_{21k}	log-normal(mean = 0.250, sd = log(2)/1.96)
τ_{22k}	log-normal(mean = 0.125, sd = log(2)/1.96)
ρ_{2k}	uniform(-1,1)

Historical data for trametinib

The dose-DLT data of trametinib single agent from the following clinical study are considered as relevant information (Table 14-8) and were used to derive the prior distribution for the BLRM parameters ($\log(\alpha_{2k})$, $\log(\beta_{2k})$) for model k (k = 1, 2, 3).

- **MEK111054:** open-label, multi-center, phase I dose-escalation study to investigate the safety, pharmacokinetics, and pharmacodynamics of trametinib in subjects with solid tumors or BRAF-mutation positive melanoma

The DLT observation window in the phase I dose escalation part of this trial was 4 weeks.

Table 14-8 Historical data from trametinib single agent clinical study

Dose level	Number of evaluable patients	Number of DLTs
2.5 mg QD	9	0
3 mg QD	12	0
4 mg QD	3	1

Single agent PDR001 Q4W:

The prior distributions for the kth model (k = 1, 2, 3) of PDR001 Q4W single agent BLRM model parameters ($\log(\alpha_{3k})$, $\log(\beta_{3k})$) are a mixture of two components: a MAP component and a robustification component used to allow for a dose/toxicity relationship for PDR001 in a combination that differs substantially from that in single agent. The components form a mixture prior with respective weights (0.95, 0.05).

MAP component for PDR001 for model 1 (dosing regimen 1)

For the MAP model for dosing regimen 1 of PDR001, reference dose d_{ref} = 400 mg (Q4W) is used, and data from S = 1 historical study are available.

Weakly informative normal priors are assumed for μ_{311} and μ_{321}, with means corresponding to an assumed 10% risk of DLT at the reference dose of 400 mg, and a double in dose leading to a doubling in the odds of a DLT, respectively. Priors for τ_{311} and τ_{321} are assigned such that (1) their medians correspond to moderate between-trial heterogeneity, and (2) their uncertainty (95% prior interval) cover plausible between-trial standard deviations.

The prior distributions for the model used for deriving the MAP priors for dosing regimen 1 of PDR001 are specified in Table 14-9.

Table 14-9 Prior distributions for the parameters of the MAP model used to derive the prior for the single agent PDR001 model parameters for model 1 (dosing regimen 1)

Parameter	Prior distribution
μ_{311}	N(mean = logit(0.1), sd = 2)
μ_{321}	N(mean = logit(0.5), sd=1)
τ_{311}	log-normal(mean = 0.250, sd = log(2)/1.96)
τ_{321}	log-normal(mean = 0.125, sd = log(2)/1.96)
ρ_{31}	uniform(-1,1)

MAP component for PDR001 for model 2 (dosing regimen -1a)

For the MAP model for dosing regimen -1a of PDR001, reference dose d_{ref} = 400 mg (Q4W) is used, and data from S = 1 historical study are available.

Weakly informative normal priors are assumed for μ_{312} and μ_{322}, with means corresponding to an assumed 10% risk of DLT at the reference dose of 400 mg, and a double in dose leading to a doubling in the odds of a DLT, respectively. Priors for τ_{312} and τ_{322} are assigned such that (1) their medians correspond to substantial between-trial heterogeneity, and (2) their uncertainty (95% prior interval) cover plausible between-trial standard deviations. As less is information is known about Q8W versus Q4W dosing of PDR001, substantial between-trial heterogeneity is

assumed for PDR001 in dosing regimen -1a. The prior distributions for the model used for deriving the MAP priors for dosing regimen -1a of PDR001 are specified in Table 14-10.

Table 14-10 Prior distributions for the parameters of the MAP model used to derive the prior for the single agent PDR001 model parameters for model 2 (dosing regimen -1a)

Parameter	Prior distribution
μ_{312}	N(mean = logit(0.1), sd = 2)
μ_{322}	N(mean = logit(0.5), sd=1)
τ_{312}	log-normal(mean = 0.50, sd = log(2)/1.96)
τ_{322}	log-normal(mean = 0.25, sd = log(2)/1.96)
ρ_{32}	uniform(-1,1)

MAP component for PDR001 for model 3 (dosing regimen -1b)

For the MAP model for dosing regimen -1b of PDR001, reference dose d_{ref} = 400 mg (Q4W) is used, and data from S = 1 historical study are available.

Weakly informative normal priors are assumed for μ_{313} and μ_{323}, with means corresponding to an assumed 10% risk of DLT at the reference dose of 400 mg, and a double in dose leading to a doubling in the odds of a DLT, respectively. Priors for τ_{313} and τ_{323} are assigned such that (1) their medians correspond to substantial between-trial heterogeneity, and (2) their uncertainty (95% prior interval) cover plausible between-trial standard deviations. As less is information is known about Q4W dosing of PDR001 after a 4 week run-in of dabrafenib and trametinib versus immediate Q4W dosing, substantial between-trial heterogeneity is assumed for PDR001 in dosing regimen -1b. The prior distributions for the model used for deriving the MAP priors for dosing regimen -1b of PDR001 are specified in Table 14-11.

Table 14-11 Prior distributions for the parameters of the MAP model used to derive the prior for the single agent PDR001 model parameters for model 3 (dosing regimen -1b)

Parameter	Prior distribution
μ_{313}	N(mean = logit(0.1), sd = 2)
μ_{323}	N(mean = logit(0.5), sd=1)
τ_{313}	log-normal(mean = 0.50, sd = log(2)/1.96)
τ_{323}	log-normal(mean = 0.25, sd = log(2)/1.96)
ρ_{33}	uniform(-1,1)

Historical data for PDR001

The dose-DLT data of PDR001 single agent from the following clinical study are considered as relevant information (Table 14-12) and were used to derive the prior distribution for the BLRM parameters ($\log(\alpha_{3k})$, $\log(\beta_{3k})$) for model k (k = 1, 2, 3).

- **CPDR001X201:** open-label, multi-center, phase I/II study of the safety and efficacy of PDR001 administered to patients with advanced malignancies

The DLT observation window in the phase I dose escalation part of this trial was 4 weeks. A review of AE records of the patients did not indicate any significant additional toxicity in the 2nd cycle of treatment, suggesting data from the 1 cycle DLT evaluation period of study

CPDR001X2101 is also information for a 2 cycle DLT evaluation period. Assuming the average weight of a patients is 80 kg, each dose level in the historical data d_{Q4W}[mg/kg] can be converted to a flat dose level, using the following:

$$d_{Q4W}\ [mg] = d_{Q4W}[mg/kg] * 80[kg]$$

Table 14-12 Historical data from PDR001 Q4W single agent clinical study

Dosing schedule	Dose level (mg/kg)	Converted to dose level in Q4W (mg)	Number of evaluable patients	Number of DLTs
q4w	3	240	6	0
q4w	5	400	10	0

Robustification component of PDR001

To take into account the potential situation that PDR001 in combination is substantially more toxic than when administered as single agent, and that the longer DLT period of 8 weeks (56 days) may increase the chance of experiencing toxicity, a second prior component with vague bivariate normal distribution centered around a higher toxicity is added to improve the robustness of the final prior. The parameters of this weakly information prior distribution are described below:

- The mean ($\log(\alpha_{3k})$, $\log(\beta_{3k})$) = (logit(0.33), 0), i.e. the median DLT rate at the reference dose (400 mg), was assumed to be 0.33 and doubling in dose was assumed to double odds of DLT.
- To complete the specification, the prior standard deviation, sd($\log(\alpha^{3k})$, $\log(\beta^{3k})$), was set equal to (2,1) which allows for considerable prior uncertainty for the dose/toxicity profile.
- The correlation, corr($\log(\alpha_{3k})$, $\log(\beta_{3k})$), was set equal to 0.

14.2.3.2 Prior distribution for interaction parameters

Based on the available information, there is a possibility of an antagonistic or synergistic interaction between dabrafenib and trametinib that will be taken into account for model 1 (dosing regimen 1) and model 2 (dosing regimen -1a). For model 3 (dosing regimen -1b), the interaction is centered at a 0% increase in odds of DLT, given that DLT's for the triple combination in this dosing regimen will be assessed after a 4 week run-in with dabrafenib an and trametinib. No interaction is expected for the other two-way combinations, and for the three-way combination no interaction is expected over and above what is already described. However, in all cases, considerable uncertainty remains as to the true interaction, and normal priors are used that allow for both synergistic and antagonistic toxicity. The following assumptions will be used for interaction parameters for model k (k = 1, 2, 3).

Dabrafenib and trametinib for model 1 (dosing regimen 1) and 2 (dosing regimen -1a):

- η_{12k} is normally distributed and centered at 1.2, i.e. a 20% increase in odds of DLT over independence at the combination starting dose
- 97.5^{th} percentile is log(3), i.e. a 3-fold increase in odds of DLT over independence at the combination starting dose

Dabrafenib and trametinib for model 3 (dosing regimen -1b):

- η_{123} is normally distributed and centered at 0, i.e. a 0% increase in odds of DLT over independence at the combination starting dose
- 97.5th percentile is log(2), i.e. a 2-fold increase in odds of DLT over independence at the combination starting dose

Dabrafenib and PDR001 for all 3 models:

- η_{13k} is normally distributed and centered at 0, i.e. a 0% increase in odds of DLT over independence at the combination starting dose
- 97.5th percentile is log(2), i.e. a 2-fold increase in odds of DLT over independence at the combination starting dose

Trametinib and PDR001 for all 3 models:

- η_{23k} is normally distributed and centered at 0, i.e. a 0% increase in odds of DLT over independence at the combination starting dose
- 97.5th percentile is log(2), i.e. a 2-fold increase in odds of DLT over independence at the combination starting dose

Dabrafenib, trametinib and PDR001 for all 3 models:

- η_{123k} is normally distributed and centered at 0, i.e. a 0% increase in odds of DLT over independence at the combination starting dose
- 97.5th percentile is log(1.5), i.e. a 1.5-fold increase in odds of DLT over independence at the combination starting dose

14.2.3.3 Additional historical data for the combination of dabrafenib and trametinib

The dose-DLT data for the combination of dabrafenib and trametinib from the following clinical studies are considered as relevant information (Table 14-13) and will be incorporated into the prior model using discounted weighting.

- **BRF113220:** open-label, multi-center phase I/II dose escalation study to investigate the safety, pharmacokinetics, pharmacodynamics, and clinical activity of dabrafenib in combination with trametinib in subjects with BRAF mutant melanoma
- **MEK115306 (COMBI-d):** randomized, double-blind, phase III study comparing the combination of dabrafenib and trametinib to dabrafenib and placebo as first-line therapy in subjects with unresectable (Stage IIIc) or metastatic (Stage IV) BRAF V600E/K mutation-positive cutaneous melanoma
- **MEK116513 (COMBI-v):** randomized, open-label, phase III study comparing the combination of dabrafenib and trametinib to vemurafenib in subjects with unresectable (Stage IIIc) or metastatic (Stage IV) BRAF V600E/K mutation-positive cutaneous melanoma

The DLT observation window in the phase I dose escalation trial was 3 weeks.

As DLT's weren't formally assessed in the phase III's studies, a post-hoc analysis was conducted to estimate the number of patients who met DLT criteria in the first 8 weeks of combination treatment.

Data from these studies will be incorporated into the prior model using discounted weighting, where weight is determined as:

weight = $1 / (1 + (2*n*\tau^2)/(sd^2)$, where n refers to study sample size and τ and sd correspond to the assumed mean and standard deviation for between-trial heterogeneity. In this case, a substantial between-trial heterogeneity is assumed (τ= 0.50, sd = 2).

Table 14-13 Historical data for combination of dabrafenib and trametinib

Study	Dose level of dabrafenib and trametinib (mg/kg)	Number of evaluable patients	Number of DLTs	Weight to be used in prior model
BRF113220	75 mg BID + 1 mg QD	4	0	0.33
	150 mg BID + 1 mg QD	4	0	
	150 mg BID + 1.5 mg QD	4	0	
	150 mg BID + 2 mg QD	4	1	
MEK115306 (COMBI-d)	150 mg BID + 2 mg QD	209	23	0.037
MEK116513 (COMBI-v)	150 mg BID + 2 mg QD	350	51	0.022

14.2.3.4 Summary of prior distributions

The prior distributions of the model parameters and prior summaries for DLT rates for model 1 (dosing regimen 1) are summarized in Table 14-14 and Table 14-15.

Table 14-14 Prior distribution for model parameters: model 1 (dosing regimen 1)

Parameter	mean	Standard deviations	Correlation	weight
**Dabrafenib single agent MAP prior**				
$(\log(\alpha_{11}), \log(\beta_{11}))$ ~ BVN				
MAP component	(-3.049, 0.190)	(0.880, 0.835)	-0.284	N/A
**Trametinib single agent MAP prior**				
$(\log(\alpha_{21}), \log(\beta_{21}))$ ~ BVN				
MAP component	(-3.708, 0.052)	(1.090, 0.943)	-0.381	N/A
**PDR001 single agent model**				
$(\log(\alpha_{31}), \log(\beta_{31}))$ ~ BVN				
MAP component	(-3.983, 0.058)	(1.361, 1.011)	0.063	0.95
Robustification component	(-0.708, 0)	(2, 1)	0	0.05
**Interaction parameters ~ Normal**				
η_{121}	0.182	0.468	N/A	N/A
η_{131}	0	0.354	N/A	N/A
η_{231}	0	0.354	N/A	N/A
η_{1231}	0	0.207	N/A	N/A

Dosing regimen 1: 400 mg i.v. PDR001 Q4W, 150 mg BID dabrafenib, and 2 mg QD trametinib

Table 14-15 Summary of prior distribution of DLT rates (with dabrafenib fixed at 150 mg BID and trametinib fixed at 2 mg QD) for model 1 (dosing regimen 1)

PDR dose (regimen)	Prior probabilities that P(DLT) is in the interval:					Quantiles		
	[0, 0.16)	[0.16, 0.33)	[0.33,1]	Mean	SD	2.5%	50%	97.5%
400 mg (Q4W)	0.555	0.326	0.120	0.185	0.146	0.033	0.144	0.594

Table 14-15 shows that the prior model for dosing regimen 1 results in a probability of excessive toxicity (i.e. DLT rate of ≥ 33%) with the triple combination of 12% which satisfies the EWOC criteria (< 25%).

The prior distributions of the model parameters and prior summaries for DLT rates for model 2 (dosing regimen -1a) are summarized in Table 14-16 and Table 14-17.

Table 14-16 Prior distribution for model parameters: model 2 (dosing regimen -1a)

Parameter	mean	Standard deviations	Correlation	weight
**Dabrafenib single agent MAP prior**				
($\log(\alpha_{12})$, $\log(\beta_{12})$) ~ BVN				
MAP component	(-3.049, 0.190)	(0.880, 0.835)	-0.284	N/A
**Trametinib single agent MAP prior**				
($\log(\alpha_{22})$, $\log(\beta_{22})$) ~ BVN				
MAP component	(-3.708, 0.052)	(1.090, 0.943)	-0.381	N/A
**PDR001 single agent model**				
($\log(\alpha_{32})$, $\log(\beta_{32})$) ~ BVN				
MAP component	(-3.946, 0.051)	(1.500, 1.045)	0.061	0.95
Robustification component	(-0.708, 0)	(2, 1)	0	0.05
**Interaction parameters ~ Normal**				
η_{122}	0.182	0.468	N/A	N/A
η_{132}	0	0.354	N/A	N/A
η_{232}	0	0.354	N/A	N/A
η_{1232}	0	0.207	N/A	N/A

Dosing regimen -1a: 400 mg i.v. PDR001 Q8W, 150 mg BID dabrafenib, and 2 mg QD trametinib

Table 14-17 Summary of prior distribution of DLT rates (with dabrafenib fixed at 150 mg BID and trametinib fixed at 2 mg QD) for model 2 (dosing regimen -1a)

PDR dose (regimen)	Prior probabilities that P(DLT) is in the interval:					Quantiles		
	[0, 0.16)	[0.16, 0.33)	[0.33,1]	Mean	SD	2.5%	50%	97.5%
400 mg (Q8W*)	0.639	0.301	0.060	0.157	0.117	0.037	0.129	0.467

*Per PK simulations, it is reasonable to assume that 400 mg Q8W is approximately equivalent to 230 mg Q4W. Results are therefore shown for 230 mg Q4W.

Table 14-17 shows that the prior model for dosing regimen -1a results in a probability of excessive toxicity with the triple combination of 6% which satisfies the EWOC criteria.

The prior distributions of the model parameters and prior summaries for DLT rates for model 3 (dosing regimen -1b) are summarized in Table 14-18 and Table 14-19.

Table 14-18 Prior distribution for model parameters: model 3 (dosing regimen -1b)

Parameter	mean	Standard deviations	Correlation	weight
**Dabrafenib single agent MAP prior**				
($\log(\alpha_{13})$, $\log(\beta_{13})$) ~ BVN				
MAP component	(-3.049, 0.190)	(0.880, 0.835)	-0.284	N/A
**Trametinib single agent MAP prior**				
($\log(\alpha_{23})$, $\log(\beta_{23})$) ~ BVN				
MAP component	(-3.708, 0.052)	(1.090, 0.943)	-0.381	N/A
**PDR001 single agent model**				
($\log(\alpha_{33})$, $\log(\beta_{33})$) ~ BVN				
MAP component	(-3.946, 0.051)	(1.500, 1.045)	0.061	0.95
Robustification component	(-0.708, 0)	(2, 1)	0	0.05
**Interaction parameters ~ Normal**				
η_{123}	0	0.354	N/A	N/A
η_{133}	0	0.354	N/A	N/A
η_{233}	0	0.354	N/A	N/A
η_{1233}	0	0.207	N/A	N/A

Dosing regimen -1b: 4 weeks of 150 mg dabrafenib and 2 mg QD trametinib, followed by 400 mg i.v. PDR001 Q4W, 150 mg BID dabrafenib, and 2 mg QD trametinib

Table 14-19 Summary of prior distribution of DLT rates (with dabrafenib fixed at 150 mg BID and trametinib fixed at 2 mg QD) for model 3 (dosing regimen -1b)

PDR dose (regimen)	Prior probabilities that P(DLT) is in the interval:					Quantiles		
	[0, 0.16)	[0.16, 0.33)	[0.33,1]	Mean	SD	2.5%	50%	97.5%
400 mg (Q4W*)	0.608	0.291	0.102	0.173	0.145	0.030	0.132	0.601

*Q4W after a 4 week run-in with dabrafenib and trametinib.

Table 14-19 shows that the prior model for dosing regimen -1b results in a probability of excessive toxicity with the triple combination of 10% which satisfies the EWOC criteria.

14.2.3.5 Incorporating trial data into models 1, 2, and 3

When incorporating trial data from the first dosing regimen into model 1, a weight of 1 will be used. If the updated results for model 1 indicate that dosing regimen 1 is not tolerated, then new patients will be enrolled into dosing regimens -1a and -1b simultaneously. In this case:

- For model 2 (dosing regimen -1a): when incorporating trial data from dosing regimen -1a, a weight of 1 will be used. For data from regimen 1 and -1b, a discounted weighting approach will be used.
- For model 3 (dosing regimen -1b): when incorporating trial data from dosing regimen -1b, a weight of 1 will be used. For data from regimen 1 and -1a, a discounted weighting approach will be used.

Discounted weighting will be determined as:

- Weight = $1 / (1 + 2*n*\tau^2) / (sd^2)$, where n refers to the number of patients with data for a particular dosing regimen and τ and sd correspond to the assumed mean and standard deviation for between-trial heterogeneity.

- For model 2 (dosing regimen -1a): a substantial between-trial heterogeneity is assumed (τ = 0.50, sd = 2). Assuming data are evaluable for 6 patients per dosing regimen, weight is equal to 0.571.
- For model 3 (dosing regimen -1b): as less information is known about Q4W dosing of PDR001 after a 4 week run-in of dabrafenib and trametinib, large between-trial heterogeneity is assumed (τ = 1, sd = 2). Assuming data are evaluable for 6 patients per dosing regimen, weight is equal to 0.25.

14.2.4 Hypothetical scenarios

To illustrate the performance of the Bayesian model used to guide dosing decisions for PDR001 in combination with dabrafenib and trametinib, hypothetical scenarios following the provisional dose regimens specified in Section 6.2.2 are displayed in Table 14-20 and Table 14-21. In each case, the recommended next dosing regimen is determine using the model-based assessment of the risk of DLT in future patients and the dosing rules as described in Section 6.2.3. In practice, a dose below the recommended dosing regimen might be chosen based on additional safety, PK, or PD information.

Table 14-20 Hypothetical scenarios (with dabrafenib fixed at 150 mg BID and trametinib fixed at 2 mg QD) for all 3 dosing regimens, given 0-6 DLT's are observed in the first 6 patients receiving dosing regimen 16-6

				Next dose level (regimen)#		
**Scenario**	**PDR001 Regimen**	**Number of patients**	**Number of DLTs**	**Dose level: (regimen)#**	**Median P(DLT)**	**P(excessive toxicity)#**
1	Q4W<br>Q8W^<br>Q4W*	6	0	1 (Q4W)	0.097	0.010
2	Q4W<br>Q8W^<br>Q4W*	6	1	1 (Q4W)	0.145	0.050
3	Q4W<br>Q8W^<br>Q4W*	6	2	1 (Q4W)	0.208	0.166
4	Q4W<br>Q8W^<br>Q4W*	6	3	-1A (Q8W),<br>-1B (Q4W*)	0.189<br>0.177	0.122<br>0.166
5	Q4W<br>Q8W^<br>Q4W*	6	4	-1A (Q8W),<br>-1B (Q4W*)	0.230<br>0.206	0.246<br>0.240
6	Q4W<br>Q8W^<br>Q4W*	6	5	Stop		
7	Q4W<br>Q8W^<br>Q4W*	6	6	Stop		

				Next dose level (regimen)#		
Scenario	PDR001 Regimen	Number of patients	Number of DLTs	Dose level: (regimen)#	Median P(DLT)	P(excessive toxicity)#

^Per PK simulations, it is reasonable to assume that 400 mg Q8W is approximately equivalent to 230 mg Q4W. Results are therefore shown for 230 mg Q4W.
* Q4W after a 4 week run-in with dabrafenib and trametinib.
# Results for Q4W come from Model 1; Q8W come from Model 2; Q4W* come from Model 3.
Dosing regimen 1 (Q4W): weight for incorporating data from dosing regimen 1 = 1.
Dosing regimen -1A (Q8W): weight for incorporating data from dosing regimen 1 = 0.571.
Dosing regimen -1B (Q4W*): weight for incorporating data from dosing regimen 1 = 0.25.

Table 14-20 shows that:

- If 0, 1, or 2 DLT's are observed in the first 6 patients who receive dosing regimen 1 (Q4W of PDR001), the probability of excessive toxicity (i.e. DLT rate ≥ 33%) with dosing regimen 1 is < 25%, satisfying the EWOC criteria. In this case, it would be recommended to continue with Q4W dosing of PDR001.
- If 3 or 4 DLT's are observed in the first 6 patients, the probability of excessive toxicity with dosing regimen 1 does not satisfy the EWOC criteria. In this case, it would be recommended to test dosing regiments -1a (Q8W of PDR001) and -1b (Q4W of PDR001, after 4 week run-in of dabrafenib and trametinib).
- If 5 or 6 DLT's are observed in the first 6 patients, the probability of excessive toxicity for all 3 dosing regimens do not satisfy the EWOC criteria. In this case, it would be recommended to stop the study.

Table 14-21 Hypothetical scenarios (with dabrafenib fixed at 150 mg BID and trametinib fixed at 2 mg QD) for cohorts -1a and -1b, given 3 DLT's are observed in first 6 patients receiving dosing regimen 1

				Next dose level (regimen)#		
Scenario	PDR001 Regimen	Number of patients	Number of DLTs	Dose level: (regimen)#	Median P(DLT)	Next dose level: (regimen)#
1	Q8W^	6	0	-1A (Q8W)	0.123	0.004
	Q4W*	6	0	-1B (Q4W*)	0.109	0.013
2	Q8W^	6	0	-1A (Q8W)	0.141	0.009
	Q4W*	6	1	-1B (Q4W*)	0.156	0.059
3	Q8W^	6	1	-1A (Q8W)	0.152	0.015
	Q4W*	6	0	-1B (Q4W*)	0.118	0.019
4	Q8W^	6	1	-1A (Q8W)	0.171	0.032
	Q4W*	6	1	-1B (Q4W*)	0.167	0.071
5	Q8W^	6	0	-1A (Q8W)	0.157	0.020
	Q4W*	6	2	-1B (Q4W*)	0.216	0.175
6	Q8W^	6	2	-1A (Q8W)	0.184	0.050
	Q4W*	6	0	-1B (Q4W*)	0.126	0.024
7	Q8W^	6	1	-1A (Q8W)	0.189	0.051
	Q4W*	6	2	-1B (Q4W*)	0.226	0.198
8	Q8W^	6	2	-1A (Q8W)	0.205	0.088
	Q4W*	6	1	-1B (Q4W*)	0.176	0.087
9	Q8W^	6	2	-1A (Q8W)	0.228	0.139
	Q4W*	6	2	-1B (Q4W*)	0.239	0.236
10	Q8W^	6	0	-1A (Q8W)	0.175	0.039
	Q4W*	6	3	Stop		

				Next dose level (regimen)#		
**Scenario**	**PDR001 Regimen**	**Number of patients**	**Number of DLTs**	**Dose level: (regimen)#**	**Median P(DLT)**	**Next dose level: (regimen)#**
11	Q8W^	6	3	-1A (Q8W)	0.219	0.120
	Q4W*	6	0	-1B (Q4W*)	0.135	0.031
12	Q8W^	6	1	-1A (Q8W)	0.211	0.102
	Q4W*	6	3	Stop		
13	Q8W^	6	3	-1A (Q8W)	0.244	0.190
	Q4W*	6	1	-1B (Q4W*)	0.187	0.107
14	Q8W^	6	2	-1A (Q8W)	0.251	0.215
	Q4W*	6	3	Stop		
15	Q8W^	6	3	Stop		
	Q4W*	6	2			
16	Q8W^	6	3	Stop		
	Q4W*	6	3			

^Per PK simulations, it is reasonable to assume that 400 mg Q8W is approximately equivalent to 230 mg Q4W. Results are therefore shown for 230 mg Q4W.
*Q4W after a 4 week run-in with dabrafenib and trametinib.
#Results for Q8W come from Model 2; results for Q4W* come from Model 3.
Dosing regimen -1A (Q8W): weight for incorporating data from dosing regimen -1A = 1; weight for incorporating data from dosing regimen 1 and -1B data = 0.571.
Dosing regimen -1B (Q4W*): weight for incorporating data from regimen -1B = 1; weight for incorporating data from dosing regimen 1 and -1A data = 0.25.

Table 14-21 shows that in the case that 3 DLT’s are observed in the first 6 patients who receive dosing regimen 1:

- If 0, 1, or 2 DLT’s are observed in the first 6 patients who receive dosing regimen -1a (Q8W of PDR001) and 0, 1, or 2 DLT’s are observed in the first 6 patients who receive dosing regimen -1b (Q4W of PDR001, after 4 week run-in of dabrafenib and trametinib), the probability of excessive toxicity (i.e. DLT rate ≥ 33%) for both dosing regimens satisfy the EWOC criteria. In this case, it would be recommended to continue with either dosing regimen -1a or -1b. Final probability values for both regimens, along with additional safety and PK data, would be used to decide which regimen should be selected.
- If 0, 1, or 2 DLT’s are observed in the first 6 patients who receive dosing regimen -1a and 3 DLT’s are observed in the first 6 patients who receive dosing regimen -1b, only the probability of excessive toxicity for dosing regimen -1a satisfies the EWOC criteria. In this case, it would be recommended to continue with dosing regimen -1a.
- If 3 DLT’s are observed in the first 6 patients who receive dosing regimen -1a and 0 or 1 DLT is observed in the first 6 patients who receive dosing regimen -1b, the probability of excessive toxicity for both dosing regimens satisfy the EWOC criteria. In this case, it would be recommended to continue with either dosing regimen -1a or -1b. Final probability values for both regimens, along with additional safety and PK data, would be used to decide which regimen should be selected.
- If ≥ 3 DLT’s are observed in the first 6 patients who receive dosing regimen -1a and ≥ 2 DLT’s are observed in the first 6 patients who receive dosing regimen -1b, the probability of excessive toxicity for both dosing regimens do not satisfy the EWOC criteria. In this case, it would be recommended to stop the study.

Note that the actual number of evaluable patients per cohort is variable (see Section 6.2.3). Recommendations during the study will depend on the number of evaluable patients at each dosing regimen and the observed number of DLT's.

Overall, from the examples illustrated above, it can be seen that the BLRM leads to decisions that make clinical sense.

14.2.5 References

Neuenschwander B, Matano A, Tang Z, et al (2014). A Bayesian Industry Approach to Phase I Combination Trials in Oncology. In Statistical Methods in Drug Combination Studies. Zhao W and Yang H (eds), Chapman & Hall/CRC

14.3 Appendix 3: Safety follow up flow chart

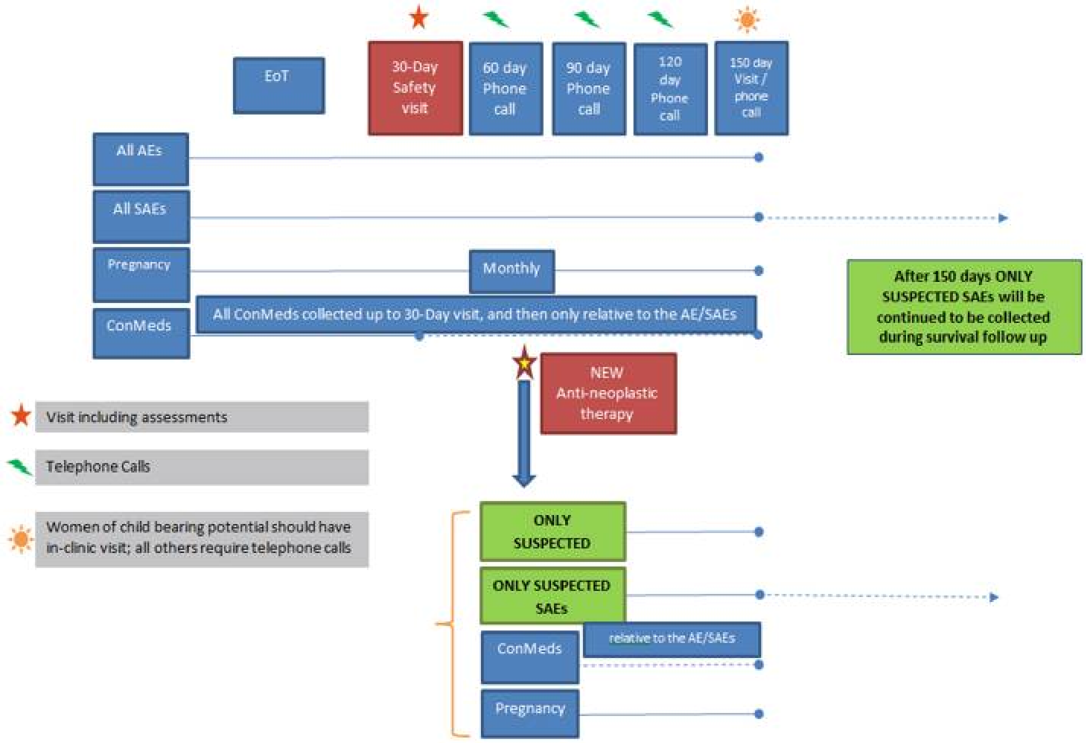

14.4 AJCC Melanoma Staging System (Edition 8)

Table 14-22 Definition of primary tumor (T)

T category	Thickness	Ulceration status
TX: not assessable	N/A	N/A
T0: No evidence of primary tumor (e.g, unknown)	N/A	N/A
Tis (melanoma *in situ*)	N/A	N/A
T1	≤1.0 mm	Unknown or unspecified
T1a	<0.8 mm	Without ulceration
T1b	<0.8 mm	With ulceration
	0.8 to 1 mm	With or without ulceration
T2	>1 to 2 mm	Unknown or unspecified
T2a	>1 to 2 mm	Without ulceration
T2b	>1 to 2 mm	With ulceration
T3	>2 to 4 mm	Unknown or unspecified
T3a	>2 to 4 mm	Without ulceration
T3b	>2 to 4 mm	With ulceration
T4	>4 mm	Unknown or unspecified
T4a	>4 mm	Without ulceration
T4b	>4 mm	With ulceration

Table 14-23 Definition of Regional Lymph Node (N)

N category	Number of tumor-involved regional lymph node(s)	Presence of in-transit, satellite, and/or microsatellite metastases
NX	Regional nodes not assessed (eg, SLN biopsy not performed, regional nodes previously removed for another reason).<br>Exception: Pathological N category is not required for T1 melanomas, use cN.	No
N0	No regional metastases detected	No
N1	One tumor-involved node or in-transit, satellite, and/or microsatellite metastases with no tumor-involved nodes	
N1a	One clinically occult (ie, detected by SLN biopsy)	No
N1b	One clinically detected	No
N1c	No regional lymph node disease	Yes
N2	Two or three tumor-involved nodes or in-transit, satellite, and/or microsatellite metastases with one tumor-involved node	
N2a	Two or three clinically occult (ie, detected by SLN biopsy)	No
N2b	Two or three, at least one of which was clinically detected	No
N2c	One clinically occult or clinically detected	Yes
N3	Four or more tumor-involved nodes or in-transit, satellite, and/or microsatellite metastases with two or more tumor-involved nodes, or any number of matted nodes without or with in-transit, satellite, and/or microsatellite metastases	
N3a	Four or more clinically occult (ie, detected by SLN biopsy)	No
N3b	Four or more, at least one of which was clinically detected, or presence of any number of matted nodes	No
N3c	Two or more clinically occult or clinically detected and/or presence of any number of matted nodes	Yes

Table 14-24 Definition of Distant Metastasis (M)

M Category	Anatomic site	LDH level
M0	No evidence of distant metastasis	Not applicable
M1	Evidence of distant metastasis	See below
M1a	Distant metastasis to skin, soft tissue including muscle, and/or non-regional lymph node	Not recorded or unspecified
M1a(0)		Not elevated
M1a(1)		Elevated
M1b	Distant metastasis to lung with or without M1a sites of disease	Not recorded or unspecified
M1b(0)		Not elevated
M1b(1)		Elevated
M1c	Distant metastasis to non-CNS visceral sites with or without M1a or M1b sites of disease	Not recorded or unspecified
M1c(0)		Not elevated
M1c(1)		Elevated
M1d	Distant metastasis to CNS with or without M1a, M1b, or M1c sites of disease	Not recorded or unspecified
M1d(0)		Normal
M1d(1)		Elevated

Table 14-25 Pathological (pTNM)

When T is...	And N is...	And M is...	Then the pathological stage group is...
Tis	N0	M0	0
T1a	N0	M0	IA
T1b	N0	M0	IA
T2a	N0	M0	IB
T2b	N0	M0	IIA
T3a	N0	M0	IIA
T3b	N0	M0	IIB
T4a	N0	M0	IIB
T4b	N0	M0	IIC
T0	N1b, N1c	M0	IIIB
T0	N2b, N2c, N3b, or N3c	M0	IIIC
T1a/b-T2a	N1a or N2a	M0	IIIA
T1a/b-T2a	N1b/c or N2b	M0	IIIB
T2b/T3a	N1a-N2b	M0	IIIB
T1a-T3a	N2c or N3a/b/c	M0	IIIC
T3b/T4a	Any N ≥N1	M0	IIIC
T4b	N1a-N2c	M0	IIIC
T4b	N3a/b/c	M0	IIID
Any T, Tis	Any N	M1	IV
