# Supplementary material for: Spartalizumab or placebo in combination with dabrafenib and trametinib in patients with BRAF V600-mutant melanoma: exploratory biomarker analyses from a randomized phase 3 trial (COMBI-i)
Source: J Immunother Cancer. 2022 Jun 21;10(6):e004226. doi: 10.1136/jitc-2021-004226 (PMC9214378; doi:10.1136/jitc-2021-004226)
Supplement: Supplementary data [file jitc-2021-004226supp001.pdf]

ONLINE SUPPLEMENTAL

METHODS

Tumor assessments

Treatment began on day 1 of cycle 1; cycles were defined as 28 days (≈ 4 weeks). Tumor assessments using Response Evaluation Criteria in Solid Tumors version 1.1 were conducted at baseline, at 12 weeks, then every 8 weeks for the first 18 months of treatment, and then every 3 months thereafter until disease progression, death, loss to follow-up, or withdrawal from study. An additional confirmatory tumor assessment was required no less than 4 weeks after response criteria were first met. Tumor response was also assessed by blinded independent central review based on the original imaging scans.

Tissue preparation

For immunohistochemistry (IHC), formalin-fixed paraffin-embedded (FFPE) tissue blocks or unstained slides were used. Blocks were sectioned in a nuclease-free manner into 4-µm-thick FFPE slides and baked at 60°C (± 2°C) for 30 minutes to 2 hours, depending on the antibody, at HistoGeneX (now CellCarta; Antwerp, Belgium). Tumor evaluation was performed by a certified pathologist.

For NanoString testing, sections of 4-µm (± 1 µm) thickness were cut from all tissue blocks received. A pathologist visually inspected archival FFPE and freshly cut slides to note the approximate percentage of tumor content in the region of interest (ROI) and the total tumor area (mm^2). Depending on the tumor content, four to eight slides were macrodissected for RNA and DNA isolation. If the ROI contained < 10% tumor content, further processing was canceled.

Tawbi HA, *et al. J Immunother Cancer* 2022; 10:e004226. doi: 10.1136/jitc-2021-004226

RNA and DNA were coextracted from all samples available using the AllPrep RNA/DNA Extraction from FFPE Tissue Kit (Qiagen; Hilden, Germany). Samples yielding RNA concentrations < 5 ng/μL were not processed further.

T-cell–inflamed signature score (TIS)

For each NanoString sample and control, the counts for the 18 target genes comprising the T-cell–inflamed signature were divided by the geometric mean of 10 internal housekeeping genes (HKGs) to generate HKG-normalized expression data. The HKG-normalized data for each sample were then scaled by HKG-normalized expression in the control sample to yield reference sample–corrected and HKG-normalized results. These were then log2 transformed, and a target-specific coefficient was applied prior to summation of the normalized expression values and generation of the TIS. The cutoff log2 value of HKG-normalized counts defining low vs high TIS was 6.29, derived from the distribution of values excluding the lowest 25%.[1,2]

Immunophenotyping by flow cytometric analysis

Immunophenotyping of peripheral blood mononuclear cells was performed on baseline and week 4 paired samples using fluorochrome-conjugated monoclonal antibodies for cell-surface expression of CD45 BUV395 (clone HI30), CD3 FITC (clone SK7), CD4 BUV737 (SK3), CD8 BVy5.521 (clone RPA-TA), TCRvd2 PerCP-Cy5.5 (clone B6), CD45RA BV711 (clone HI100), CCR7 PE (clone 150503), HLA-DR BV786 (clone G46-6), CD38 APC-eF780 (clone HIT2), PD-1 PE-Cy7 (clone EH12.1), LAG-3 PE-eF610 (clone 3DS223H), and TIM-3 BV650 (7D3) (BD Biosciences; San Jose, CA) and intracellular expression of Ki67 AF647 (clone B56). Cell viability was measured using Viability Dye BV510 (Thermo Fisher Scientific; Waltham, MA).

Tawbi HA, *et al. J Immunother Cancer* 2022; 10:e004226. doi: 10.1136/jitc-2021-004226

Pharmacodynamic biomarkers and phenotypic and functional characteristics of T cells were defined by coexpression of $CD38^+$/$HLA\text{-}DR^+$/$CD8^+$ (activated/cytotoxic $CD8^+$ T cells), $Ki67^+$/$CD8^+$ (proliferating $CD8^+$ T cells), and $PD\text{-}1^+$/$Ki67^+$/$CD8^+$ (activated/proliferating $CD8^+$ T cells). A minimum of 5000 white blood cells per sample were acquired using a BD LSRFortessa X-20 and analyzed by FlowJo software (v10.2) (BD Biosciences) at Navigate BioPharma, a Novartis subsidiary (Carlsbad, CA). For visualization purposes, the logistic transformation log (p/[1−p]) was applied to proportions positive for plotted analytes, transforming proportions into "logits." Since proportions of 0.0 or 1.0 would be undefined, all proportions were shrunk toward 0.5 using the transformation ([100 p+1/2]/[100+1]) prior to applying the logistic transformation.

Cytokine profiling

Cytokines profiled included interferon (IFN)-γ, IL (interleukin) 6, IL-8, TNF-α, IL-12p40, IL-15, IL-16, IL-17A, TNF-β, eotaxin, IP-10, MDC, MIP-1α, PLGF, VEGFC, VEGFR1, CRP, ICAM-1, SAA1, VCAM-1, and IL-18. Plasma samples were diluted two-fold with Diluent 2 as recommended by the assay kit manufacturer (Meso Scale Diagnostics [MSD]; Rockville, MD) using 50 μL of calibrators and diluted samples for each replicate. Three levels of controls were included in each run. Standards, controls, and samples were tested in duplicate. Assay signal, proportional to the amount of analyte present in the sample, was read on an MSD instrument. A four-parameter logistic curve fit was used to construct the standard curve off of which cytokine levels in the test samples were determined. Results from controls were checked before sample results were accepted. Those falling below the lower limit of detection were analyzed and plotted as limit/2, and those falling above the upper limit of detection were analyzed and plotted as 1.1 ×

limit. Limits were batch specific, and the data set contained multiple lower and upper limits for certain analytes.

Dualplex IHC assays and digital image analysis by HALO

Stained slides were scanned at 20× magnification using an Aperio AT2 Leica digital whole-slide scanner (Leica Biosystems; Wetzlar, Germany). Digital image analysis (IA) was then performed using the Multiplex IHC module HALO software platform version 2.3 (Indica Labs; Albuquerque, NM). A specific IA algorithm was developed internally at Novartis Precision Medicine to assess $CD8^+$ cells within melanoma lesions annotated by a pathologist working closely with imaging scientists; residential lymphoid tissue was excluded from IA. The algorithm together with a tissue classifier were used to quantitatively assess the percentage of $CD8^+$ cells within the melanoma tumor and stroma compartments. Lastly, infiltration analysis was performed within defined ROIs using the tumor boundary from the melanoma lesion classifier as the infiltration margin. Five bands (30 µm each) within and outside the tumor margin were analyzed for a total distance of 150 µm on each side. The $CD8^+$ cell density (cells/mm^2) was reported for each infiltration band.

CD8 phenotypes for infiltration analysis

Infiltration analysis was used to determine three CD8 phenotypes (T-cell–excluded/–infiltrated/immune desert) based on five 30-µm inner (150 to 0 µm) and two 30-µm outer (0 to 60 µm) bands of CD8 density around the tumor-stroma invasive margin. Samples were ranked by their average tumor CD8 density values, and the top third of tumors were defined as inflamed. Similarly, stroma and other samples with values ranked in the bottom third were defined as

desert. Samples that were below the tumor threshold for inflamed and above the stroma cutoff for desert were defined as excluded. A small number of cases had a high CD8 density in the tumor area but a low CD8 density in the stroma, which was usually caused by small stroma area, and were defined as inflamed tumors.

CD11b/CD14/CD80/CD86/HLA-DR/DAPI multiplex fluorescence IHC and IA by AQUA

FFPE tissue samples were dewaxed and rehydrated through a series of xylene-to-alcohol washes to distilled water. Heat-induced antigen retrieval was then performed using the NxGen Decloaking Chamber in Diva buffer (Biocare Medical; Pacheco, CA) and transferred to tromethamine-buffered saline. All subsequent staining steps were performed at room temperature. Endogenous peroxidase was blocked using Peroxidazed 1 (Biocare) followed by incubation with a protein-blocking solution (Background Sniper; Biocare) to reduce nonspecific antibody staining. Slides were stained following the procedure described below:

**Bake and Dewax**	**Antigen Retrieval**	**First Round: CD80**		
20 min	20 min	Primary, 60 min	Secondary, 30 min	Detection, 10 min
Dewax buffer at 95°C	ER1 at 95°C	Mouse anti-CD80 (1:100 in DVGD)	Envision mouse	Opal 570 (1:200)
**Stripping**		**Second Round: CD14**		
20 min		Primary, 60 min	Secondary, 30 min	Detection, 10 min
ER1 at 95°C		Rabbit anti-CD14 (1:500 in DVGD)	Envision rabbit	Opal 520 (1:200)
**Stripping**		**Third Round: CD11b**		
20 min		Primary, 60 min	Secondary, 30 min	Detection, 10 min
ER1 at 95°C		Rabbit anti-CD11b (1:500 in DVGD)	Envision rabbit	Opal 620 (1:200)
**Stripping**		**Fourth Round: CD86**		
20 min		Primary, 60 min	Secondary, 30 min	Detection, 10 min
ER1 at 95°C		Rabbit anti-CD86	Envision	Opal 480

Tawbi HA, *et al. J Immunother Cancer* 2022; 10:e004226. doi: 10.1136/jitc-2021-004226

	(1:100 in DVGD)	rabbit	(1:200)
**Stripping**	**Fifth Round: HLA-DR**		
20 min	Primary, 60 min	Secondary, 30 min	Detection, 10 min
ER1 at 95°C	Mouse anti–HLA-DR (1:500 in DVGD)	Envision mouse	Opal DIG (1:50)
**Stripping**	**Fifth Round (Cont): Opal 780**		
20 min	Primary, 70 min		
ER1 at 95°C	Anti-DIG Opal 780 (1:50)		
**Sixth Round: DAPI**			
Primary, 5 min			
DAPI			

CD, cluster of differentiation; cont, continued; DAPI, 4′,6-diamidino-2-phenylindole; DIG, digoxygenin; ER1, epitope retrieval solution 1; HLA-DR, human leukocyte antigen, DR subtype.

Fluorescence images were acquired on the Vectra 2 Intelligent Slide Analysis System (Akoya Biosciences; Menlo Park, CA) first at 4× magnification to identify tissue areas based on DAPI signal. These 4× magnification images were processed using an automated INFORM software enrichment algorithm (INFORM GmbH; Aachen, Germany) to identify and rank 20× high-power fields of view according to the highest coexpression of CD11b and HLA-DR. All raw images were reviewed by a pathologist for acceptability. Images lacking tumor or highly necrotic cells were rejected prior to assessment by Automated QUantitative Analysis (AQUA [v3.2.4]; HistoRx; New Haven, CT) via a fully automated process. AQUA has been extensively validated in clinical settings for objective quantitation of biomarkers in tissues,[3-8] and the assays applied in this report were validated at Navigate BioPharma, a Novartis subsidiary, using Novartis internal guidance and standard operating procedures for exploratory assay development. DAPI signal within each accepted image was used to identify cell nuclei and then dilated to the approximate size of an entire cell. Using an overlap approach, a binary mask for CD11b and HLA-DR was

created to identify the $CD11b^{+}$/HLA-DR^{-} population that represents myeloid-derived suppressor cells.

RNA library processing

The captured next-generation sequencing (NGS) library was pooled with other libraries, each having a unique adaptor index sequence, and applied to a sequencing flow cell for cluster amplification and massively parallel sequencing by synthesis using Illumina (San Diego, CA) v4 chemistry and paired-end 100-bp reads. Sequence data were aligned to the reference human genome (build hg19) using STAR.[9] Mapped reads were used to quantify transcripts with HTSeq[10] and the RefSeq GRCh38 v82 gene annotation. Data were normalized using trimmed mean of M-value normalization as implemented in the edgeR R/Bioconductor package.[11]

Targeted and circulating tumor DNA sequencing

Samples were submitted to Foundation Medicine, Inc (Cambridge, MA), for NGS of tumor and circulating tumor (ctDNA) with the FoundationOne CDx assay. After sample adequacy assessment, DNA extraction and quality check, and library construction and quality check, hybrid capture and genomic profiling to identify genetic alterations in the NGS data were performed as previously described.[12,13] Sequencing data were mapped to the human genome (build hg19). Variant calling, including base substitutions, insertions/deletions (indels), copy number alterations, and genomic rearrangements, was performed in targeted genomic regions. Libraries were sequenced using Illumina v4 chemistry and paired-end 100-bp reads (HiSeq; Illumina).

Tawbi HA, *et al. J Immunother Cancer* 2022; 10:e004226. doi: 10.1136/jitc-2021-004226

For ctDNA sequencing data, unique molecular identifiers (UMIs) were trimmed from the reads using UMI-Toolkit v1, and the reads were then aligned to the human reference genome (build hg38) using BWA-MEM.[14] The alignments were then locally realigned and base quality scores recalibrated (Genome Analysis Toolkit [GATK]).[15,16] Consensus reads were created using the UMI and alignment position to remove polymerase chain reaction-duplicate reads and sequencing artifacts (UMI-Toolkit). Single-nucleotide variants (SNVs) were identified with MuTect v1.1.7.[17] Indels were identified using Pindel v1.0.[18] Structural variants were identified using PureCN v1.8.1.[19] Chromosomal rearrangements were called using Socrates v1.[20]

ctDNA libraries were included in the downstream analysis if the coverage was ≥ 500× and GC/AT dropouts were < 20%. Potential sequencing artifacts and germline genetic variants were removed from downstream analyses. A position-specific error rate was calculated based on the sequencing of plasma from 24 healthy controls, and mutations were retained only if they had support significantly greater than the position-specific error rate. Additional potential artifacts were removed based on low allelic fraction (< 0.005 unless known or probable oncogenic), poorly supported alignments (> 50 MQ0 reads), low base quality (< 20), low coverage (< 100×) or in repetitive regions. Probable germline SNVs and indels were identified by their presence in the databases dbSNP 147, the Exome Sequencing Project (ESP; ESP6500SI-V2-SSA137.GRCh38-liftover) and the Exome Aggregation Consortium (ExAC, now part of gnomAD; release 0.3) at appreciable frequency (ESP minor allele frequency > 0.001 or ExAC count > 3 unless a known hotspot mutation). SNVs and indels were assigned a functional significance based on their presence in the Catalog of Somatic Mutations in Cancer (COSMIC v83) and functional effect, with mutations reported in COSMIC in ≥ 5 tumors considered as

“known” oncogenic, mutations with COSMIC count < 5 but predicted to lead to premature truncation of the protein considered as “likely” oncogenic, and all others considered to have “unknown” oncogenic status. Copy number variations were considered to be amplifications if the estimated copy number was ≥ 7 or homozygous deletions if the estimated copy number was ≤ 0.5. PureCN uses a combination of the B allele frequency of single-nucleotide polymorphisms in copy number variants and the allele frequency of somatic point mutations to determine the proportion of cell-free DNA derived from the tumor.[19,21]

***Table S1.* Baseline Characteristics of the Intention-to-Treat Population[22]**

	Sparta-DabTram (n = 267)	Placebo-DabTram (n = 265)
Age, median (IQR), years	56 (46-66)	55 (47-65)
< 65 years, n (%)	189 (71)	195 (74)
≥ 65 years, n (%)	78 (29)	70 (26)
ECOG PS, n (%)		
0	195 (73)	196 (74)
1	67 (25)	66 (25)
2	5 (2)	3 (1)
Disease stage, n (%)[a]		
IIIC	16 (6)	15 (6)
IV M1a	30 (11)	42 (16)
IV M1b	55 (21)	36 (14)
IV M1c	166 (62)	172 (65)
BRAF mutation status (local), n (%)[b]		
V600E	236 (88)	236 (89)
V600K	26 (10)	22 (8)
V600 other	5 (2)	7 (3)
BRAF mutation status (central), n (%)[c]		
V600E	200 (75)	202 (76)
V600K	25 (9)	28 (11)
Not evaluable	42 (16)	35 (13)
LDH levels, n (%)		
< 1 × ULN	162 (61)	161 (61)
≥ 1 to < 2 × ULN	70 (26)	68 (26)
≥ 2 × ULN	35 (13)	36 (14)
Sum of lesion diameters at baseline, median (IQR), mm	49 (29-88)	48 (28-81)
No. of organ sites with disease, n (%)		
< 3	145 (54)	143 (54)
≥ 3	121 (45)	122 (46)
Unknown	1 (< 1)	0
Prior adjuvant therapy, n (%)	6 (2)	4 (2)
TMB status, n (%)		
< 10 mut/Mb (low)	115 (43)	122 (46)
≥ 10 mut/Mb (high)	87 (33)	90 (34)
Not evaluable	65 (24)	53 (20)
PD-L1 status, n (%)		
< 1% (negative)	98 (37)	115 (43)
≥ 1% (positive)	138 (52)	126 (48)
Not evaluable	31 (12)	24 (9)
T-cell–inflamed signature expression, n (%)		
< 6.29 log2 CPM (low)	52 (19)	58 (22)

≥ 6.29 log2 CPM (high)	160 (60)	163 (62)
Not evaluable	55 (21)	44 (17)

CPM, counts per million; ECOG PS, Eastern Cooperative Oncology Group performance status; IQR, interquartile range; LDH, lactate dehydrogenase; mut/Mb, mutations per megabase; PD-L1, programmed death ligand 1; placebo-DabTram, placebo plus dabrafenib and trametinib; sparta-DabTram, spartalizumab plus dabrafenib and trametinib; TMB, tumor mutational burden; ULN, upper limit of normal.

[a]Per American Joint Committee on Cancer's *Cancer Staging Manual*, 7th edition.

[b]Patients (n = 17) without results from local *BRAF* mutation testing were enrolled based on central *BRAF* testing results. If V600E was present with another V600 mutation, including V600K, the patient is listed under "V600E." If V600K was present with another V600 mutation, excluding V600E, the patient is listed under "V600K." "V600 other" includes patients with V600 mutations other than V600E or V600K.

[c]If both V600E and V600K mutations were present, the patient is listed under V600K.

***Table S2*. Baseline Characteristics of Biomarker Cohorts Compared With Those of Respective Cohorts Without Biomarker Data**

**TMB and PD-L1 Data Present vs One or Both Absent**				
	**N[a]**	**Present (n = 375)**	**Absent (n = 157)**	***P* Value**
Treatment with sparta-DabTram, n (%)	532	179 (48)	88 (56)	.08
Female, n (%)	532	155 (41)	70 (45)	.5
Age, median (IQR), years	532	56 (46-66)	56 (44-66)	.7
LDH levels, n (%)	532			.2
< 1 × ULN		234 (62)	89 (57)	
≥ 1 to < 2 × ULN		89 (24)	49 (31)	
≥ 2 × ULN		52 (14)	19 (12)	
Sum of lesion diameters, median (IQR), mm	526	50 (28-90)	48 (28-79)	.6
≥ 3 metastatic sites, n (%)	531	177 (47)	66 (42)	.3
ECOG PS, n (%)	531			.8
0		278 (74)	116 (74)	
1		91 (24)	40 (25)	
2		5 (1)	1 (1)	
Disease stage (per AJCC 7), n (%)	532			.5
IIIC		24 (6)	7 (4)	
IV M1a		48 (13)	24 (15)	
IV M1b		64 (17)	27 (17)	
IV M1c with normal LDH levels		117 (31)	38 (24)	
IV M1c with elevated LDH levels		122 (33)	61 (39)	
**T-Cell–Inflamed Signature Data Present vs Absent**				
	**N[a]**	**Present (n = 433)**	**Absent (n = 99)**	***P* Value**
Treatment with sparta-DabTram, n (%)	532	212 (49)	55 (56)	.2
Female, n (%)	532	178 (41)	47 (47)	.2
Age, median (IQR), years	532	55 (46-66)	59 (46-66)	.3
LDH levels, n (%)	532			.04
< 1 × ULN		273 (63)	50 (51)	
≥ 1 to < 2 × ULN		103 (24)	35 (35)	
≥ 2 × ULN		57 (13)	14 (14)	
Sum of lesion diameters, median (IQR), mm	526	48 (27-83)	53 (33-93)	.1
≥ 3 metastatic sites, n (%)	531	202 (47)	41 (41)	.4
ECOG PS, n (%)	531			.7
0		324 (75)	70 (71)	
1		103 (24)	28 (28)	
2		5 (1)	1 (1)	
Disease stage (per AJCC 7), n (%)	532			.09
IIIC		28 (6)	3 (3)	
IV M1a		57 (13)	15 (15)	

IV M1b		75 (17)	16 (16)	
IV M1c with normal LDH levels		134 (31)	21 (21)	
IV M1c with elevated LDH levels		139 (32)	44 (44)	

Flow Cytometry (Baseline CD4⁺/CD8⁺ T-Cell Ratios) Present vs Absent

	**N[a]**	**Present (n = 409)**	**Absent (n = 123)**	***P* Value**
Treatment with sparta-DabTram, n (%)	532	200 (49)	67 (54)	.3
Female, n (%)	532	169 (41)	56 (46)	.4
Age, median (IQR), years	532	56 (46-65)	57 (46-66)	.4
LDH levels, n (%)	532			.2
< 1 × ULN		255 (62)	68 (55)	
≥ 1 to < 2 × ULN		105 (26)	33 (27)	
≥ 2 × ULN		49 (12)	22 (18)	
Sum of lesion diameters, median (IQR), mm	526	47 (27-79)	60 (34-104)	.02
≥ 3 metastatic sites, n (%)	532	178 (44)	65 (53)	.2
ECOG PS, n (%)	532			.02
0		315 (77)	79 (64)	
1		88 (22)	43 (35)	
2		5 (1)	1 (1)	
Not available		1 (< 1)	0	
Disease stage (per AJCC 7), n (%)	532			.009
IIIC		27 (7)	4 (3)	
IV M1a		60 (15)	12 (10)	
IV M1b		73 (18)	18 (15)	
IV M1c with normal LDH levels		118 (29)	37 (30)	
IV M1c with elevated LDH levels		131 (32)	52 (42)	

Central *BRAF* Mutation Status Present vs Absent

	**N[a]**	**Present (n = 455)**	**Absent (n = 77)**	***P* Value**
Treatment with sparta-DabTram, n (%)	532	225 (49)	42 (55)	.4
Female, n (%)	532	191 (42)	34 (44)	.7
Age, median (IQR), years	532	55 (46-65)	60 (48-68)	.08
LDH levels, n (%)	532			.6
< 1 × ULN		280 (62)	43 (56)	
≥ 1 to < 2 × ULN		115 (25)	23 (30)	
≥ 2 × ULN		60 (13)	11 (14)	
Sum of lesion diameters, median (IQR), mm	526	48 (27-80)	60 (33-101)	.03
≥3 metastatic sites, n (%)	532	204 (45)	39 (51)	.03
ECOG PS, n (%)	532			.8
0		336 (74)	58 (75)	
1		112 (25)	19 (25)	
2		6 (1)	0	
Not available		1 (< 1)	0	
Disease stage (per AJCC 7), n (%)	532			.2
IIIC		29 (6)	2 (3)	

Tawbi HA, *et al. J Immunother Cancer* 2022; 10:e004226. doi: 10.1136/jitc-2021-004226

IV M1a		59 (13)	13 (17)	
IV M1b		81 (18)	10 (13)	
IV M1c with normal LDH levels		136 (30)	19 (25)	
IV M1c with elevated LDH levels		150 (33)	33 (43)	
**Digital Immunohistochemistry (CD8) Data Present vs Absent**				
	**N[a]**	**Present (n = 409)**	**Absent (n = 116)**	***P* Value**
Treatment with sparta-DabTram, n (%)	532	207 (51)	56 (48)	.7
Female, n (%)	532	171 (42)	48 (41)	.9
Age, median (IQR), years	532	56 (47-65)	56 (46-66)	.7
LDH levels, n (%)	532			.9
< 1 × ULN		250 (61)	68 (59)	
≥ 1 to < 2 × ULN		106 (26)	31 (27)	
≥ 2 × ULN		53 (13)	17 (15)	
Sum of lesion diameters, median (IQR), mm	526	48 (27-79)	54 (32-95)	.09
≥ 3 metastatic sites, n (%)	532	186 (45)	54 (47)	.8
ECOG PS, n (%)	532			.8
0		306 (75)	83 (72)	
1		97 (24)	32 (28)	
2		5 (1)	1 (1)	
Not available		1 (< 1)	0	
Disease stage (per AJCC 7), n (%)	532			.3
IIIC		23 (6)	7 (6)	
IV M1a		59 (14)	12 (10)	
IV M1b		74 (18)	16 (14)	
IV M1c with normal LDH levels		114 (28)	39 (34)	
IV M1c with elevated LDH levels		139 (34)	42 (36)	

For each assay, baseline clinical covariates were compared between cohorts with and without biomarker data available in order to assess potential case selection bias; *P* values unadjusted for multiplicity based on Pearson χ^2, proportional odds likelihood ratio, and Wilcoxon rank sum tests were applied to evaluate null hypotheses of "no difference" for unordered categorical, ordered categorical, and continuous covariates, respectively.

AJCC 7, American Joint Committee on Cancer's *Cancer Staging Manual*, 7th edition; ECOG PS, Eastern Cooperative Oncology Group performance status; IQR, interquartile range; LDH, lactate dehydrogenase; PD-L1, programmed death ligand 1; sparta-DabTram, spartalizumab plus dabrafenib and trametinib; TMB, tumor mutational burden; ULN, upper limit of normal.

[a]N corresponds to the total number of patients from whom data on each baseline characteristic were collected out of a possible total of 532 patients in the intention-to-treat population.

Tawbi HA, *et al. J Immunother Cancer* 2022; 10:e004226. doi: 10.1136/jitc-2021-004226

***Table S3*. Top Gene Signatures Differentially Expressed in *BRAF* V600E vs V600K Subgroups**

	W_{STAT}	Fold Change	*P* Value	False Discovery Rate
BIOCARTA_SPRY_PATHWAY	9314	1.04060867	1.64388E-06	0.002544492
REACTOME_COMMON_PATHWAY_ OF_FIBRIN_CLOT_FORMATION	9276	1.098765602	2.20684E-06	0.002544492
REACTOME_FORMATION_OF_FIBRIN_ CLOT_CLOTTING_CASCADE	9132	1.072751962	6.53214E-06	0.004425804
REACTOME_MELANIN_BIOSYNTHESIS	3385	0.793820096	7.67702E-06	0.004425804
REACTOME_CHL1_INTERACTIONS	9003	1.082241351	1.65684E-05	0.006785144
BIOCARTA_GHRELIN_PATHWAY	8994	1.110051935	1.76543E-05	0.006785144
REACTOME_VASOPRESSIN_LIKE_ RECEPTORS	8921	1.178884055	2.93379E-05	0.009664736
Endothelial_3	8864	1.061692603	4.32414E-05	0.012464329
REACTOME_CARGO_RECOGNITION_ FOR_CLATHRIN_MEDIATED_ ENDOCYTOSIS	8843	1.022069373	4.97895E-05	0.012605904
Lineage_endothelial_AvivRegev	8829	1.08681084	5.46657E-05	0.012605904
KEGG_PROXIMAL_TUBULE_ BICARBONATE_RECLAMATION	8808	1.052530752	6.2836E-05	0.013172706
REACTOME_TRAFFICKING_OF_GLUR2 _CONTAINING_AMPA_RECEPTORS	8766	1.052477515	8.2767E-05	0.015769406
REACTOME_PTK6_REGULATES_CELL_ CYCLE	3740	0.939950195	8.88995E-05	0.015769406
REACTOME_RHO_GTPASE_CYCLE	8731	1.022596588	.000103801	0.016261322
REACTOME_TP53_REGULATES_ TRANSCRIPTION_OF_DEATH_ RECEPTORS_AND_LIGANDS	8724	1.069996786	.000108574	0.016261322
BIOCARTA_PPARA_PATHWAY	8718	1.027416588	.000112828	0.016261322
BIOCARTA_CBL_PATHWAY	8658	1.043975777	.000164948	0.020244712
REACTOME_G2_PHASE	3843	0.925036238	.000171254	0.020244712
REACTOME_INTRINSIC_PATHWAY_OF _FIBRIN_CLOT_FORMATION	8651	1.05850306	.000172327	0.020244712
REACTOME_COENZYME_A_ BIOSYNTHESIS	3847	0.973782362	.000175583	0.020244712
BIOCARTA_SPPA_PATHWAY	8611	1.040333768	.000220805	0.02424651
Lineage_Megakarocyte_AvivRegev	8595	1.070434669	.000243569	0.024793299
PID_PTP1B_PATHWAY	8573	1.032366492	.00027848	0.024793299
REACTOME_G_ALPHA_Q_ SIGNALLING_EVENTS	8573	1.035230308	.00027848	0.024793299
REACTOME_PHASE_3_RAPID_ REPOLARISATION	8571	1.085241564	.000281876	0.024793299
REACTOME_TRAIL_SIGNALING	8565	1.069790492	.000292298	0.024793299
BIOCARTA_PDGF_PATHWAY	8550	1.024763299	.000319956	0.024793299
BIOCARTA_NOS1_PATHWAY	8548	1.030742472	.000323823	0.024793299
PID_THROMBIN_PAR1_PATHWAY	8545	1.019872982	.000329706	0.024793299
REACTOME_AXON_GUIDANCE	8535	1.016715722	.000350048	0.024793299
BIOCARTA_EXTRINSIC_PATHWAY	8529	1.10243525	.00036281	0.024793299
BIOCARTA_LDL_PATHWAY	8522	1.088048533	.000378249	0.024793299
BIOCARTA_EDG1_PATHWAY	8521	1.029039939	.000380504	0.024793299

Tawbi HA, *et al. J Immunother Cancer* 2022; 10:e004226. doi: 10.1136/jitc-2021-004226

REACTOME_PKMTS_METHYLATE_HISTONE_LYSINES	8520	1.032124475	.000382772	0.024793299
REACTOME_SYNTHESIS_OF_UDP_N_ACETYL_GLUCOSAMINE	3975	0.967120684	.000382772	0.024793299
BIOCARTA_INTRINSIC_PATHWAY	8514	1.062691896	.000396644	0.024793299
REACTOME_BIOSYNTHESIS_OF_THE_N_GLYCAN_PRECURSOR_DOLICHOL_LIPID_LINKED_OLIGOSACCHARIDE_LLO_AND_TRANSFER_TO_A_NASCENT_PROTEIN	3985	0.978633425	.000406153	0.024793299
BIOCARTA_EGF_PATHWAY	8509	1.022124448	.000408563	0.024793299
BIOCARTA_MSP_PATHWAY	8503	1.110684132	.000423306	0.025029312
REACTOME_SIGNALING_BY_EGFR_IN_CANCER	8494	1.02677227	.000446355	0.025732344
REACTOME_EGFR_DOWNREGULATION	8484	1.029195342	.000473336	0.026622241
REACTOME_INTERLEUKIN_7_SIGNALING	8479	1.050811254	.00048739	0.026665436
KEGG_TGF_BETA_SIGNALING_PATHWAY	8473	1.028576502	.000504768	0.026665436
BIOCARTA_AMI_PATHWAY	8471	1.06361796	.000510688	0.026665436
REACTOME_INTERLEUKIN_27_SIGNALING	8467	1.04015791	.000522722	0.026665436
REACTOME_ACTIVATION_OF_HOX_GENES_DURING_DIFFERENTIATION	8464	1.049488586	.000531921	0.026665436
BIOCARTA_IL1R_PATHWAY	8456	1.033210804	.000557194	0.026768528
REACTOME_AMYLOID_FIBER_FORMATION	8456	1.04872505	.000557194	0.026768528
PID_EPHB_FWD_PATHWAY	8450	1.022773677	.000576878	0.027148577
BIOCARTA_ERYTH_PATHWAY	8446	1.10219787	.000590358	0.027227312
KEGG_CYSTEINE_AND_METHIONINE_METABOLISM	4058	0.977645958	.000621768	0.02792646
BIOCARTA_AMAN_PATHWAY	8433	1.031174028	.000636221	0.02792646
REACTOME_TRANSPORT_OF_NUCLEOTIDE_ SUGARS	4065	0.969715072	.000647265	0.02792646
REACTOME_MAPK_FAMILY_SIGNALING_ CASCADES	8427	1.01734774	.000658487	0.02792646
BIOCARTA_HER2_PATHWAY	8425	1.020910241	.000666069	0.02792646
REACTOME_RET_SIGNALING	8406	1.024150114	.000742254	0.030564979
REACTOME_GLUCAGON_LIKE_PEPTIDE_1_GLP1_REGULATES_INSULIN_SECRETION	8399	1.019548981	.000772307	0.031244575
BIOCARTA_ACETAMINOPHEN_PATHWAY	8393	1.167484069	.000798962	0.03176563
REACTOME_SYNTHESIS_OF_IP3_AND_IP4_IN_THE_CYTOSOL	8386	1.030800011	.000831137	0.032123758
REACTOME_INTEGRIN_SIGNALING	8385	1.030217397	.000835831	0.032123758
BIOCARTA_PLCD_PATHWAY	8375	1.086351613	.000884135	0.033154233
PID_EPHA2_FWD_PATHWAY	8371	1.016506429	.000904172	0.033154233
REACTOME_SULFUR_AMINO_ACID_METABOLISM	4135	0.972793647	.000961463	0.033154233
REACTOME_ERYTHROCYTES_TAKE_UP_OXYGEN_AND_RELEASE_CARBON_DIOXIDE	8360	1.169158795	.000961463	0.033154233

REACTOME_ION_HOMEOSTASIS	8360	1.026611946	.000961463	0.033154233
REACTOME_SYNTHESIS_OF_ SUBSTRATES_IN_N_GLYCAN_ BIOSYTHESIS	4137	0.976605573	.000972234	0.033154233
REACTOME_CHONDROITIN_SULFATE _BIOSYNTHESIS	8357	1.03711389	.000977662	0.033154233
NABA_ECM_AFFILIATED	8357	1.042901978	.000977662	0.033154233
REACTOME_ORGANIC_ANION_ TRANSPORT	8352.5	1.283890351	.000998618	0.033374112
BIOCARTA_NFAT_PATHWAY	8350	1.022941354	.001016446	0.033484625
REACTOME_SIGNALING_BY_EGFR	8341	1.016892971	.001068404	0.034700552
PID_S1P_S1P3_PATHWAY	8334	1.024304081	.001110503	0.035273599
BIOCARTA_EICOSANOID_PATHWAY	8333	1.098150309	.00111664	0.035273599
REACTOME_O_GLYCOSYLATION_OF_ TSR_DOMAIN_CONTAINING_ PROTEINS	8323	1.066358042	.001179767	0.036473469
REACTOME_PRE_NOTCH_ PROCESSING_IN_THE_ENDOPLASMIC_ RETICULUM	8322	1.026456681	.001186258	0.036473469
REACTOME_OTHER_INTERLEUKIN_ SIGNALING	8319	1.018089292	.001205928	0.036514443
Endothelial_4	8311	1.118760339	.001259866	0.036514443
BIOCARTA_VEGF_PATHWAY	8311	1.021817962	.001259866	0.036514443
PID_S1P_S1P1_PATHWAY	8310	1.033638727	.001266763	0.036514443
REACTOME_SIGNALING_BY_GPCR	8310	1.020135866	.001266763	0.036514443
BIOCARTA_AT1R_PATHWAY	8305	1.021060757	.001301772	0.037060326
BIOCARTA_GATA3_PATHWAY	8302	1.027400547	.001323204	0.037211076
PID_RAC1_REG_PATHWAY	8299	1.025786037	.001344961	0.037367228
BIOCARTA_NTHI_PATHWAY	8284	1.0255393	.001458781	0.039780267
BIOCARTA_PKC_PATHWAY	8281	1.041343129	.001482584	0.039780267
BIOCARTA_PAR1_PATHWAY	8279	1.026948091	.001498652	0.039780267
REACTOME_SIGNALING_BY_ NUCLEAR_RECEPTORS	8273	1.021345984	.00154782	0.039780267
REACTOME_NOTCH4_ INTRACELLULAR_DOMAIN_ REGULATES_TRANSCRIPTION	8270	1.022116459	.001572958	0.039780267
REACTOME_CHONDROITIN_SULFATE _DERMATAN_SULFATE_METABOLISM	8268	1.032612899	.001589925	0.039780267
KEGG_SYSTEMIC_LUPUS_ ERYTHEMATOSUS	8267	1.070143867	.001598471	0.039780267
REACTOME_RUNX1_REGULATES_ GENES_INVOLVED_IN_ MEGAKARYOCYTE_ DIFFERENTIATION_AND_PLATELET_ FUNCTION	8267	1.046313144	.001598471	0.039780267
REACTOME_PLATELET_ACTIVATION_ SIGNALING_AND_AGGREGATION	8263	1.024495232	.001633081	0.039780267
KEGG_VASCULAR_SMOOTH_MUSCLE _CONTRACTION	8254	1.029242109	.00171349	0.039780267
REACTOME_G_ALPHA_12_13_ SIGNALLING_EVENTS	8253	1.020677939	.001722645	0.039780267
KEGG_NEUROACTIVE_LIGAND_ RECEPTOR_INTERACTION	8251	1.046701909	.001741091	0.039780267

REACTOME_G_ALPHA_I_SIGNALLING _EVENTS	8250	1.026857887	.001750382	0.039780267
REACTOME_NOTCH3_ INTRACELLULAR_DOMAIN_ REGULATES_TRANSCRIPTION	8250	1.027200829	.001750382	0.039780267
PID_EPHRINB_REV_PATHWAY	8248	1.035588352	.001769101	0.039780267
REACTOME_BIOSYNTHESIS_OF_ SPECIALIZED_PRORESOLVING_ MEDIATORS_SPMS	8248	1.062796091	.001769101	0.039780267
REACTOME_RUNX1_REGULATES_ EXPRESSION_OF_COMPONENTS_OF_ TIGHT_ JUNCTIONS	8246	1.039812053	.001788004	0.039780267

***Table S4*. Top Gene Signatures Associated With Improved Progression-Free Survival in Both the Sparta-DabTram and Placebo-DabTram Arms**

	Placebo-DabTram						Sparta-DabTram					
	Test	*df*	*P* Value	HR	Avg Log CPM	FDR	Test	*df*	*P* Value	HR	Avg Log CPM	FDR
Tcell_inflamed_ 18gene_ Merck	6.9973386	1	.0081631	0.7754625	4.9900542	0.1546405	14.1693644	1	.0001671	0.6757385	5.0309093	0.0341224
PID_CD8_TCR_ DOWNSTREAM_ PATHWAY	7.3552825	1	.0066866	0.5463906	3.8858485	0.1546405	13.2259484	1	.0002761	0.4120274	3.9039717	0.0341224
CD8_Tcell_Dan	9.4704884	1	.0020880	0.7338803	2.2609517	0.1546405	13.0972479	1	.0002957	0.6760745	2.3211695	0.0341224
Cytotoxic	9.4704884	1	.0020880	0.7338803	2.2609517	0.1546405	13.0972479	1	.0002957	0.6760745	2.3211695	0.0341224
REACTOME_ INTERFERON_ GAMMA_ SIGNALING	7.9381803	1	.0048402	0.6423139	5.5383384	0.1546405	12.6117407	1	.0003833	0.5542831	5.5679724	0.0341224
IFNG_28gene_Merck	8.2601084	1	.0040526	0.8001677	4.1812939	0.1546405	12.5593452	1	.0003942	0.7419113	4.2654570	0.0341224
REACTOME_ IMMUNOREGULATORY_ INTERACTIONS_ BETWEEN_A_ LYMPHOID_AND_A_ NON_LYMPHOID_CELL	6.8359209	1	.0089343	0.6838449	3.8160393	0.1546405	12.0076453	1	.0005298	0.5792560	3.8458405	0.0341224
PID_IL27_PATHWAY	7.5612452	1	.0059637	0.5725228	3.8766134	0.1546405	11.7368010	1	.0006128	0.4802072	3.9110179	0.0341224
Lineage_CD8T_Jincheng	8.7485036	1	.0030986	0.8264464	3.0259587	0.1546405	11.6090260	1	.0006563	0.7922888	3.0979724	0.0341224
BIOCARTA_CTL_ PATHWAY	8.3184339	1	.0039245	0.7674916	4.8617674	0.1546405	11.2553225	1	.0007940	0.7091900	4.9330630	0.0341224
PID_IFNG_PATHWAY	6.5660278	1	.0103944	0.3694849	6.0091049	0.1591746	11.2105077	1	.0008134	0.2169436	6.0185704	0.0341224
M1_BMS	7.0954023	1	.0077282	0.8003826	3.5103698	0.1546405	11.1787650	1	.0008274	0.7326888	3.5466687	0.0341224
KEGG_NATURAL_ KILLER_CELL_ MEDIATED_ CYTOTOXICITY	9.3086966	1	.0022807	0.4146673	3.9088796	0.1546405	11.1136270	1	.0008570	0.3435417	3.9205598	0.0341224
NK_function_GlennDranoff	8.2042177	1	.0041793	0.6134499	2.7339870	0.1546405	11.0354024	1	.0008939	0.5337673	2.7782989	0.0341224
Lineage_CytotoxicT_ AvivRegev	8.3236614	1	.0039132	0.7754039	2.6683406	0.1546405	11.0122213	1	.0009051	0.7156438	2.7221069	0.0341224
Lineage_immune_Jincheng	6.6237039	1	.0100630	0.7571477	3.4767614	0.1572896	10.9199217	1	.0009514	0.6657694	3.4874781	0.0341224
BIOCARTA_IL5_ PATHWAY	7.3075461	1	.0068666	0.6108005	2.9344007	0.1546405	10.7573498	1	.0010387	0.5320821	2.9266967	0.0341224
PID_IL12_STAT4_ PATHWAY	7.9181067	1	.0048942	0.6604880	4.5798373	0.1546405	10.6397521	1	.0011068	0.5944958	4.5892578	0.0341224
BIOCARTA_IL17_ PATHWAY	7.4599694	1	.0063086	0.7126603	2.9741063	0.1546405	10.5474953	1	.0011635	0.6435256	3.0009775	0.0341224
BIOCARTA_ BLYMPHOCYTE_ PATHWAY	6.4900847	1	.0108478	0.8029181	5.3201350	0.1591746	10.4628898	1	.0012180	0.7392298	5.3502597	0.0341224

Tawbi HA, *et al. J Immunother Cancer* 2022; 10:e004226. doi: 10.1136/jitc-2021-004226

KEGG_INTESTINAL_ IMMUNE_NETWORK_ FOR_IGA_PRODUCTION	6.8800831	1	.0087162	0.6867604	3.6473478	0.1546405	10.4356119	1	.0012361	0.6113599	3.6675983	0.0341224
PID_CD8_TCR_ PATHWAY	6.9013449	1	.0086131	0.6660705	5.1996307	0.1546405	10.4348920	1	.0012366	0.5825722	5.2327610	0.0341224
IFNG_10gene_Merck	8.1614409	1	.0042790	0.8121355	4.2353116	0.1546405	10.3967359	1	.0012624	0.7755110	4.2702299	0.0341224
KEGG_CELL_ADHESION_ MOLECULES_CAMS	6.8138035	1	.0090456	0.5583009	4.3365758	0.1546405	10.3461942	1	.0012974	0.4589655	4.3453446	0.0341224
KEGG_T_CELL_ RECEPTOR_SIGNALING_ PATHWAY	7.3208897	1	.0068158	0.4990537	4.9937133	0.1546405	10.3354581	1	.0013050	0.4044483	5.0121899	0.0341224
PID_IL12_2PATHWAY	7.6178863	1	.0057792	0.6479446	4.4628423	0.1546405	10.3329663	1	.0013068	0.5760117	4.4952550	0.0341224
Lineage_Tfh_AvivRegev	8.0582735	1	.0045296	0.7582789	4.1300249	0.1546405	10.2782152	1	.0013461	0.7002125	4.1757771	0.0341224
KEGG_JAK_STAT_ SIGNALING_PATHWAY	6.6931377	1	.0096785	0.3411091	3.6598379	0.1546405	10.2488589	1	.0013677	0.2316475	3.6707514	0.0341224
REACTOME_EXTRINSIC_ PATHWAY_FOR_ APOPTOSIS	6.4474361	1	.0111113	0.5781852	5.0425196	0.1602060	10.2057748	1	.0014000	0.4497605	5.0598908	0.0341224
Tcell_1	8.4725655	1	.0036054	0.7920639	3.0403441	0.1546405	10.0954301	1	.0014864	0.7633956	3.1138507	0.0348505
KEGG_VIRAL_ MYOCARDITIS	7.1724055	1	.0074033	0.5469232	5.5149403	0.1546405	9.9964921	1	.0015684	0.4495254	5.5180751	0.0351333
BIOCARTA_ TCYTOTOXIC_ PATHWAY	8.4507721	1	.0036489	0.7927967	4.6127283	0.1546405	9.9731453	1	.0015884	0.7667565	4.6648197	0.0351333
REACTOME_PD1_ SIGNALING	7.4532553	1	.0063322	0.7924210	4.6080733	0.1546405	9.9225125	1	.0016327	0.7531237	4.6392582	0.0351333
Lineage_NK_AvivRegev	8.0596920	1	.0045261	0.6896883	3.1521564	0.1546405	9.9177223	1	.0016369	0.6366492	3.1417347	0.0351333
BIOCARTA_NO2IL12_ PATHWAY	8.5332723	1	.0034871	0.7287629	3.3026567	0.1546405	9.7995431	1	.0017456	0.6922833	3.3427410	0.0364091
BIOCARTA_CTLA4_ PATHWAY	7.4175654	1	.0064590	0.7491202	4.4492272	0.1546405	9.7688724	1	.0017749	0.7019784	4.4582352	0.0364091
BIOCARTA_IL2RB_ PATHWAY	7.5809032	1	.0058990	0.5010033	5.9372363	0.1546405	9.7049239	1	.0018377	0.4258979	5.9685317	0.0364091
BIOCARTA_TC_ APOPTOSIS_ PATHWAY	10.122388 2	1	.0014648	0.7673136	3.6705106	0.1546405	9.5962888	1	.0019497	0.7580023	3.6967218	0.0364538
BIOCARTA_IL12_ PATHWAY	8.8537883	1	.0029248	0.6517756	3.9198493	0.1546405	9.5625741	1	.0019858	0.6202427	3.9537424	0.0364538
PID_TCR_PATHWAY	6.7060424	1	.0096087	0.6509354	5.4719976	0.1546405	9.5044319	1	.0020498	0.5758866	5.4962640	0.0364538
REACTOME_ COSTIMULATION_BY_ THE_ CD28_FAMILY	6.7549166	1	.0093490	0.5983445	5.4372828	0.1546405	9.4845167	1	.0020721	0.5119856	5.4504252	0.0364538
REACTOME_TRANSLOCATION_ OF_ZAP_70_TO_ IMMUNOLOGICAL_ SYNAPSE	7.6143962	1	.0057904	0.8099262	4.7231812	0.1546405	9.4768749	1	.0020808	0.7815901	4.7608555	0.0364538
BIOCARTA_TCRA_ PATHWAY	7.8777203	1	.0050048	0.8028368	5.0099223	0.1546405	9.4564479	1	.0021041	0.7745542	5.0545349	0.0364538
Bcell_2	7.7757658	1	.0052952	0.8160592	2.7990354	0.1546405	9.4272769	1	.0021378	0.7949247	2.9756741	0.0365030
REACTOME_GENERATION_OF_ SECOND_MESSENGER_ MOLECULES	7.2662147	1	.0070264	0.7477471	5.1816082	0.1546405	9.3808282	1	.0021927	0.7059001	5.2113039	0.0365030

Tawbi HA, *et al. J Immunother Cancer* 2022; 10:e004226. doi: 10.1136/jitc-2021-004226

General_immune_2	6.7351896	1	.0094529	0.7957626	3.8543524	0.1546405	9.3200743	1	.0022666	0.7505324	3.9317884	0.0366216
BIOCARTA_TCR_PATHWAY	6.8934513	1	.0086512	0.5354491	5.8404331	0.1546405	9.2534551	1	.0023505	0.4446587	5.8592317	0.0366677
BIOCARTA_THELPER_ PATHWAY	8.3481480	1	.0038608	0.7868793	4.8433679	0.1546405	9.1050995	1	.0025490	0.7672801	4.8810306	0.0366677
BIOCARTA_ASBCELL_ PATHWAY	7.8258234	1	.0051505	0.7087311	3.6553289	0.1546405	9.0731863	1	.0025938	0.6576562	3.6340428	0.0366677

CPM, counts per million; *df*, degrees of freedom; FDR, false discovery rate; HR, hazard ratio; placebo-DabTram, placebo plus dabrafenib and trametinib; sparta-DabTram, spartalizumab plus dabrafenib and trametinib.

***Figure S1*. COMBI-i trial consort diagram.**[22]

BID, twice daily; Q4W, every 4 weeks; QD, once daily.

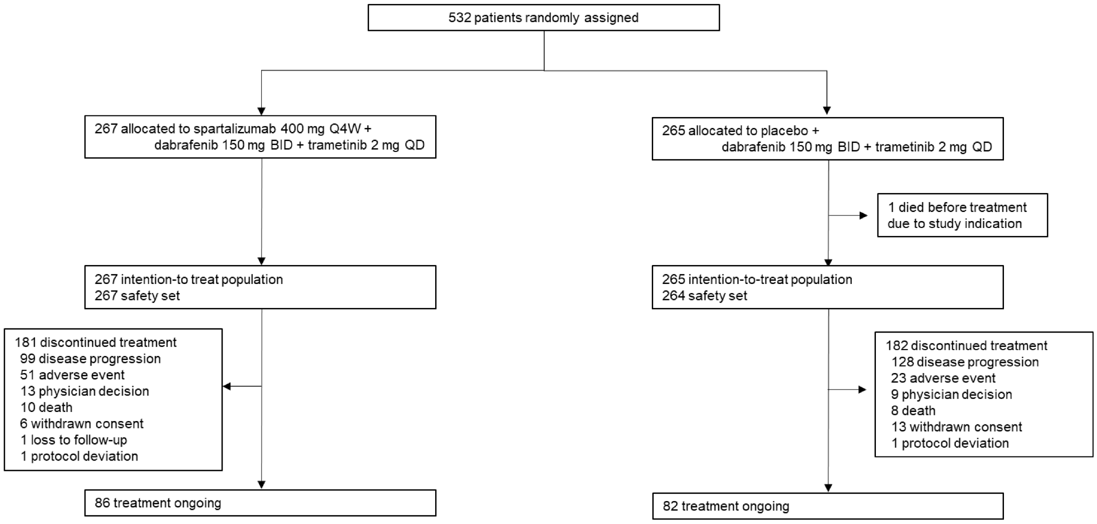

Figure S2. **Survival outcomes based on combined PD-L1 status and TMB.** Shown are Kaplan-Meier estimates of progression-free survival (A) and overall survival (B) based on PD-L1 status (< 1% [negative] or ≥ 1% [positive]) and TMB (< 10 mut/Mb [low] or ≥ 10 mut/Mb [high]). PD-L1 negative/TMB high (left top), N = 62; PD-L1 negative/TMB low (left bottom), N = 109; PD-L1 positive/TMB high (right top), N = 110; PD-L1 positive/TMB low (right bottom), N = 119. HR, hazard ratio; mut/Mb, mutations per megabase; NE, not estimable; PD-L1, programmed death ligand 1; placebo-DabTram, placebo plus dabrafenib and trametinib; sparta-DabTram, spartalizumab plus dabrafenib and trametinib; TMB, tumor mutational burden.

A

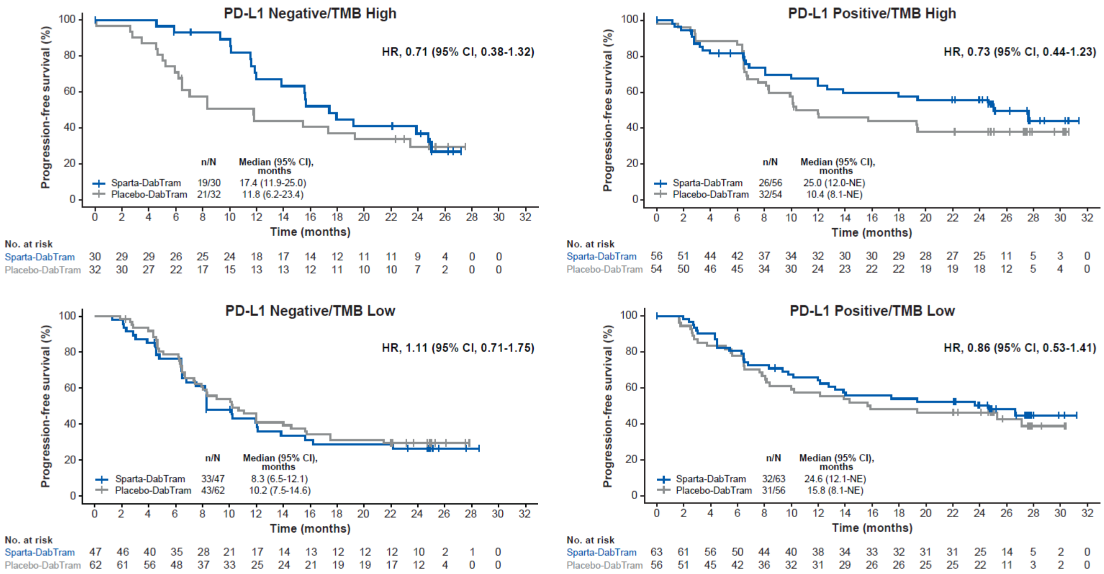

Tawbi HA, *et al. J Immunother Cancer* 2022; 10:e004226. doi: 10.1136/jitc-2021-004226

B

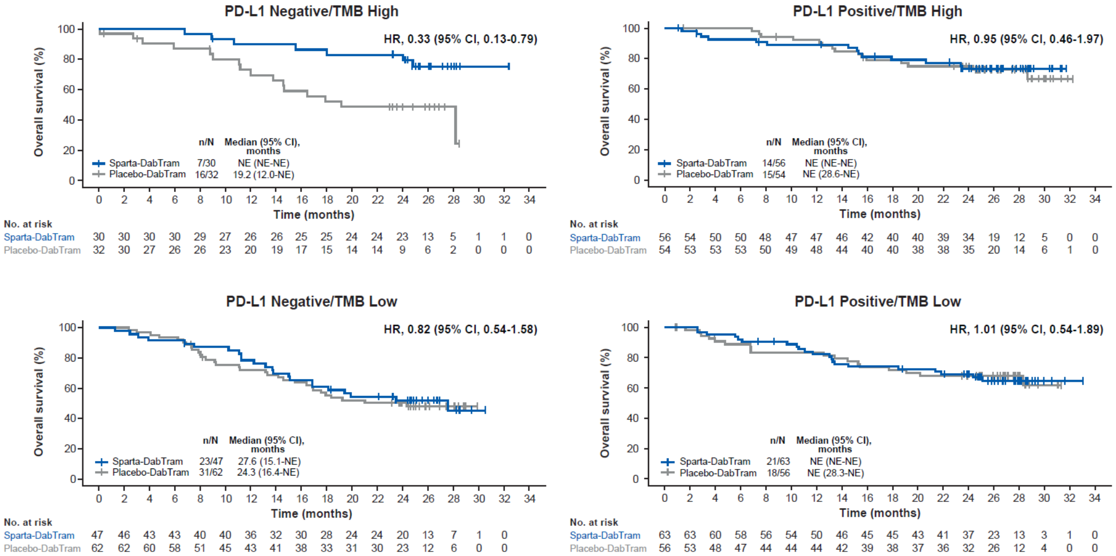
PD-L1 Negative/TMB High
HR, 0.33 (95% CI, 0.13-0.79)
n/N Median (95% CI), months
Sparta-DabTram 7/30 NE (NE-NE)
Placebo-DabTram 16/32 19.2 (12.0-NE)
Overall survival (%)
Time (months)
No. at risk
Sparta-DabTram 30 30 30 30 29 27 26 26 25 25 24 24 23 13 5 1 1 0
Placebo-DabTram 32 30 27 26 26 23 20 19 17 15 14 14 9 6 2 0 0 0
PD-L1 Positive/TMB High
HR, 0.95 (95% CI, 0.46-1.97)
n/N Median (95% CI), months
Sparta-DabTram 14/56 NE (NE-NE)
Placebo-DabTram 15/54 NE (28.6-NE)
Overall survival (%)
Time (months)
No. at risk
Sparta-DabTram 56 54 50 50 48 47 47 46 42 40 40 39 34 19 12 5 0 0
Placebo-DabTram 54 53 53 53 50 49 48 44 40 40 38 38 35 20 14 6 1 0
PD-L1 Negative/TMB Low
HR, 0.82 (95% CI, 0.54-1.58)
n/N Median (95% CI), months
Sparta-DabTram 23/47 27.6 (15.1-NE)
Placebo-DabTram 31/62 24.3 (16.4-NE)
Overall survival (%)
Time (months)
No. at risk
Sparta-DabTram 47 46 43 43 40 40 36 32 30 28 24 24 20 13 7 1 0
Placebo-DabTram 62 62 60 58 51 45 43 41 38 33 31 30 23 12 6 0 0
PD-L1 Positive/TMB Low
HR, 1.01 (95% CI, 0.54-1.89)
n/N Median (95% CI), months
Sparta-DabTram 21/63 NE (NE-NE)
Placebo-DabTram 18/56 NE (28.3-NE)
Overall survival (%)
Time (months)
No. at risk
Sparta-DabTram 63 63 60 58 56 54 50 46 45 45 43 41 37 23 13 3 1 0
Placebo-DabTram 56 53 48 47 44 44 44 42 39 38 37 36 32 26 12 4 0 0

Figure S3. **Analysis of the tumor microenvironment across combined PD-L1/TMB–defined subgroups.**

Comparison of T-cell–inflamed signature levels (N = 362) (A) and antigen-presenting cell counts based on expression of HLA-DR (N = 320) (B) and CD11b (N = 320) (C) within tumor samples from PD-L1/TMB–defined subgroups. Antigen-presenting cells were analyzed using multiplex fluorescence immunohistochemistry; shown are examples of high (D) and low (E) expressors. CD, cluster of differentiation; CPM, counts per million; DAPI, 4′,6-diamidino-2-phenylindole; GES, gene expression signature; HLA-DR, human leukocyte antigen, DR subtype; PD-L1, programmed death ligand 1; TMB, tumor mutational burden.

A

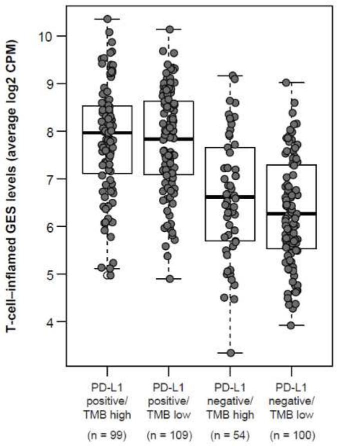

B

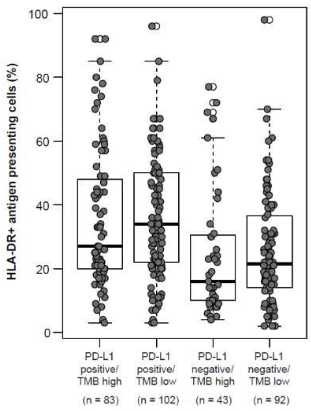

C

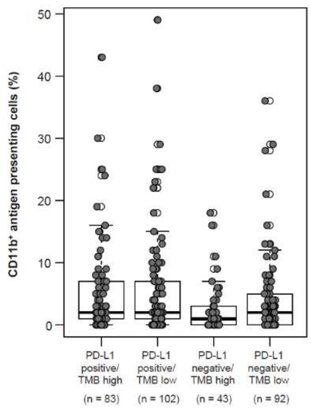

D

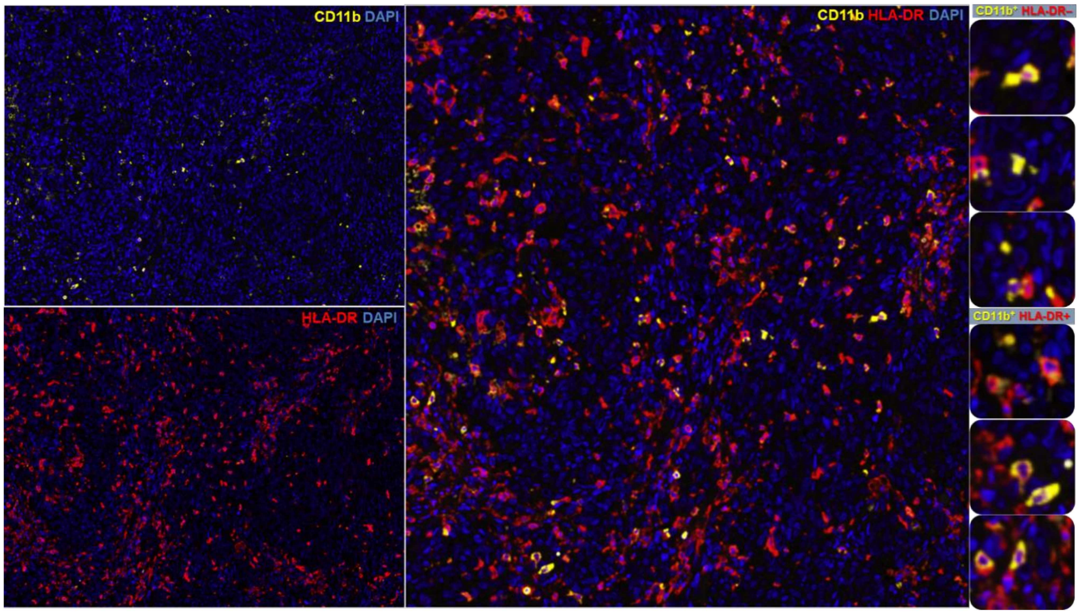
CD11b DAPI
HLA-DR DAPI
CD11b HLA-DR DAPI
CD11b+ HLA-DR−
CD11b+ HLA-DR+

E

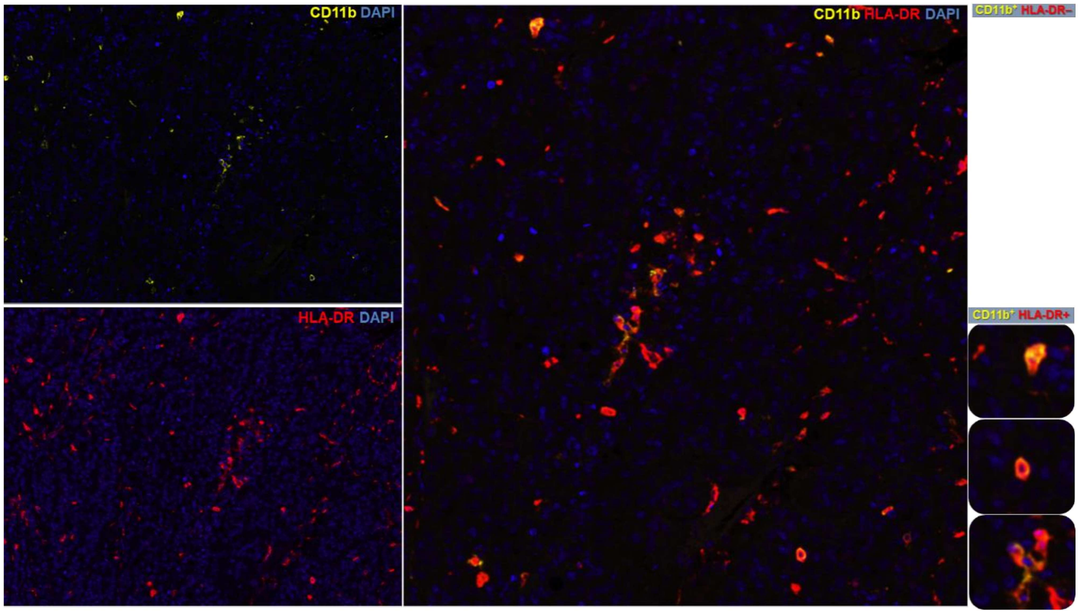
CD11b DAPI
HLA-DR DAPI
CD11b HLA-DR DAPI
CD11b+ HLA-DR−
CD11b+ HLA-DR+

Figure S4. **Analysis of tumor mutational burden (N = 384) (A) and age (N = 455) (B) based on *BRAF* V600K vs V600E mutation status.** mut/Mb, mutations per megabase.

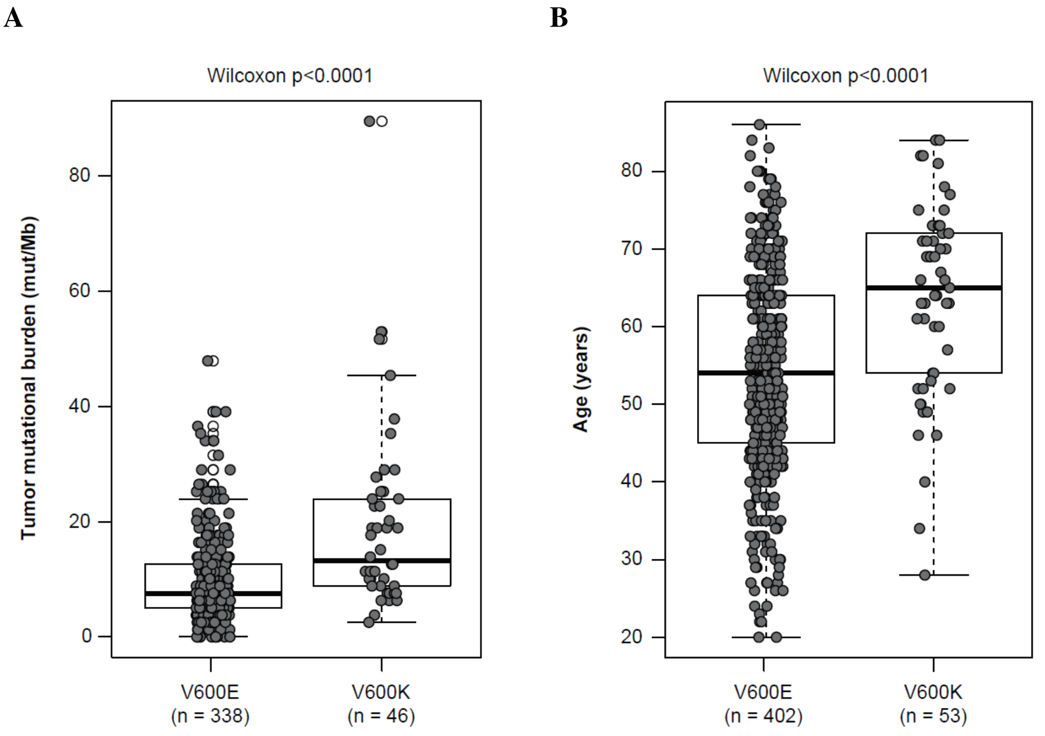

Figure S5. **Survival outcomes based on *BRAF* V600E/K mutation status.** Shown are Kaplan-Meier estimates of progression-free survival (A) and overall survival (B) in patients with confirmed *BRAF* V600E–mutant (N = 402) or V600K–mutant (N = 53) disease based on central assessment. HR, hazard ratio; NE, not estimable; placebo-DabTram, placebo plus dabrafenib and trametinib; sparta-DabTram, spartalizumab plus dabrafenib and trametinib.

A

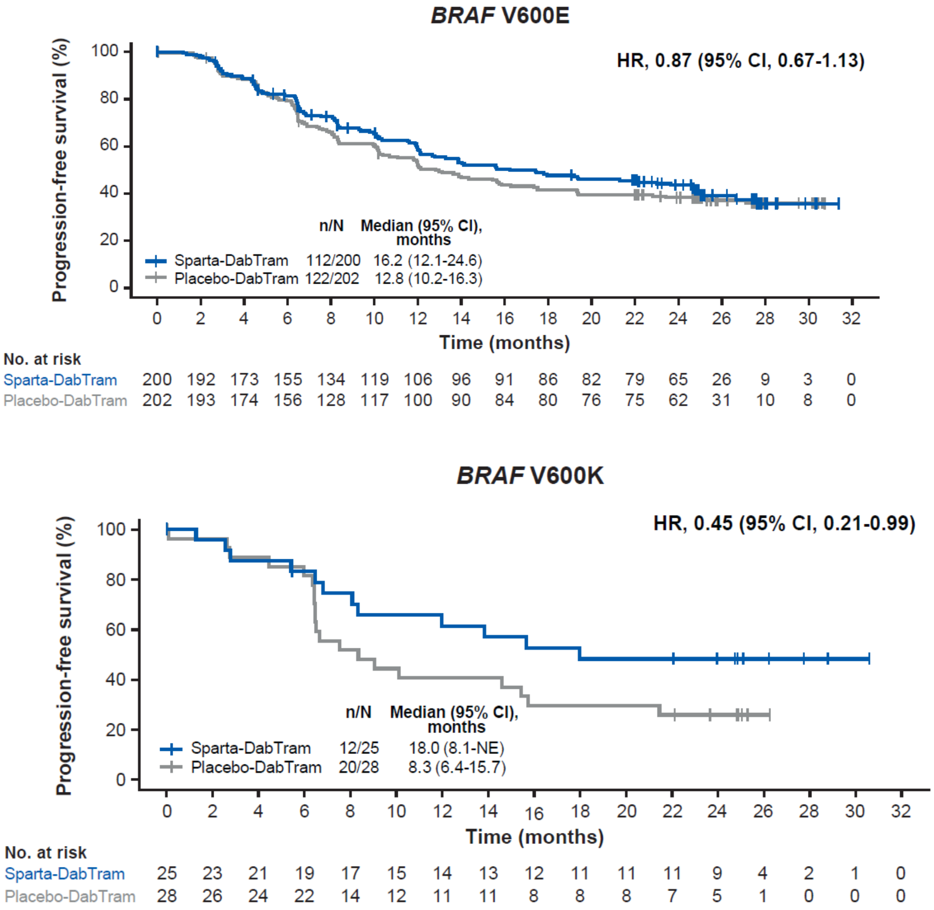

Tawbi HA, *et al. J Immunother Cancer* 2022; 10:e004226. doi: 10.1136/jitc-2021-004226

B

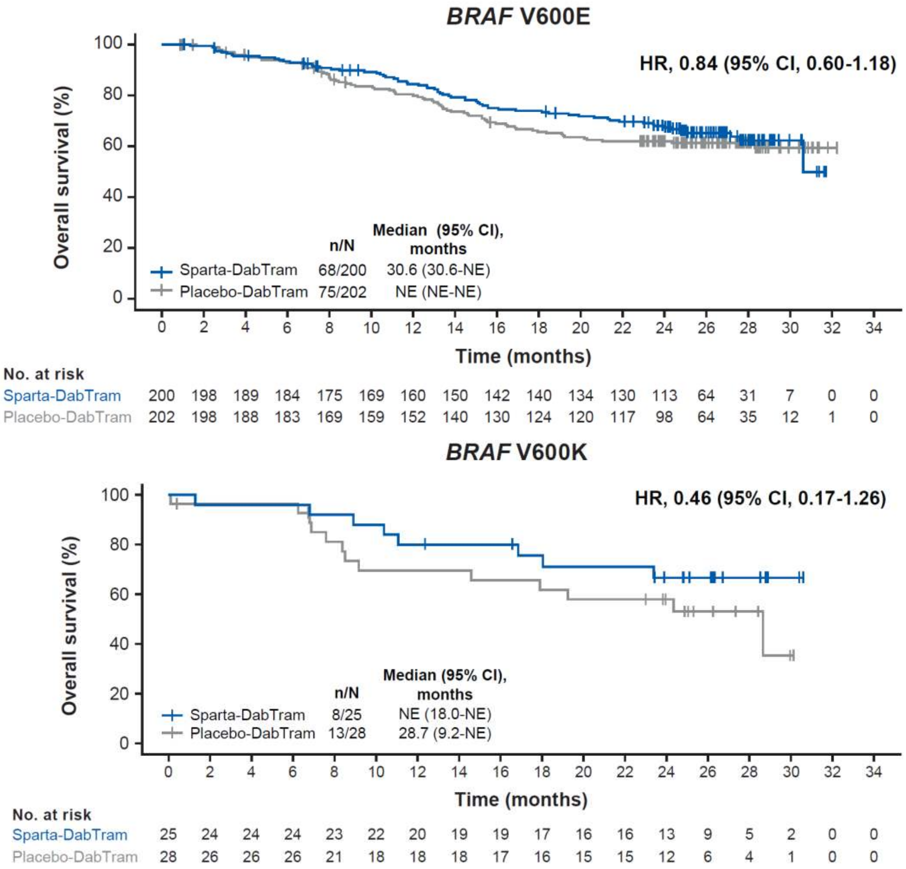
BRAF V600E
HR, 0.84 (95% CI, 0.60-1.18)
Overall survival (%)
n/N
Median (95% CI), months
Sparta-DabTram 68/200 30.6 (30.6-NE)
Placebo-DabTram 75/202 NE (NE-NE)
Time (months)
No. at risk
Sparta-DabTram 200 198 189 184 175 169 160 150 142 140 134 130 113 64 31 7 0 0
Placebo-DabTram 202 198 188 183 169 159 152 140 130 124 120 117 98 64 35 12 1 0
BRAF V600K
HR, 0.46 (95% CI, 0.17-1.26)
Overall survival (%)
n/N
Median (95% CI), months
Sparta-DabTram 8/25 NE (18.0-NE)
Placebo-DabTram 13/28 28.7 (9.2-NE)
Time (months)
No. at risk
Sparta-DabTram 25 24 24 24 23 22 20 19 19 17 16 16 13 9 5 2 0 0
Placebo-DabTram 28 26 26 26 21 18 18 18 17 16 15 15 12 6 4 1 0 0

Figure S6. **Downregulation of SPRY signaling pathways in patients with *BRAF* V600K–mutant disease.** Assessment of the SPRY signaling pathway gene expression signature in tumor samples (N = 369) included analysis of the following genes: *MAPK3, SPRY2, HRAS, MAP2K1, RAF1, SPRY4, SPRY3, EGF, MAPK1, SHC1, PTPRB, SPRY1, GRB2, EGFR, CBL, RASA1,* and *SOS1*. CPM, counts per million; GES, gene expression signature.

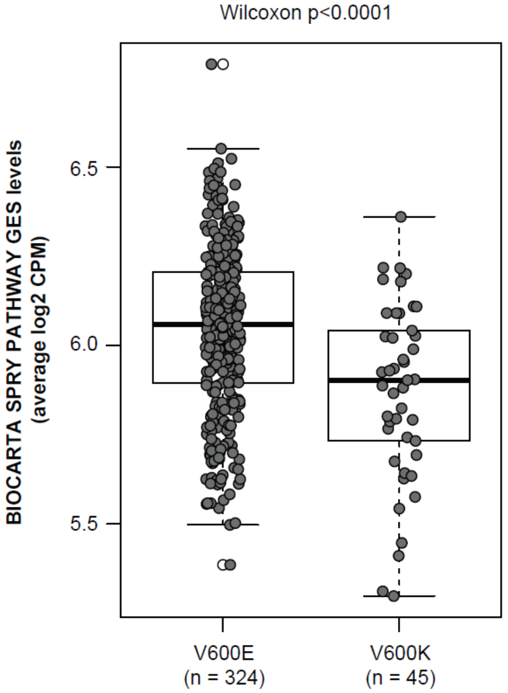

Figure S7. **Progression-free survival benefit based on gene expression signature levels.** Among the top 100 gene expression signatures in each arm, there was an overlap of 49 signatures (listed in online supplemental table S4) highly expressed in tumor samples from both sparta-DabTram– and placebo-DabTram–treated patients (A). An example of the prognostic and predictive value of these signatures, illustrated by Kaplan-Meier estimates of progression-free survival based on T-cell–inflamed signature expression (low expression [top], N = 110; high expression [bottom], N = 323), is shown in (B). GES, gene expression signature; HR, hazard ratio; NE, not estimable; placebo-DabTram, placebo plus dabrafenib and trametinib; sparta-DabTram, spartalizumab plus dabrafenib and trametinib.

A

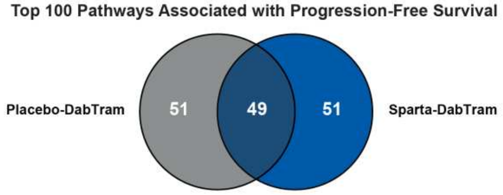

B

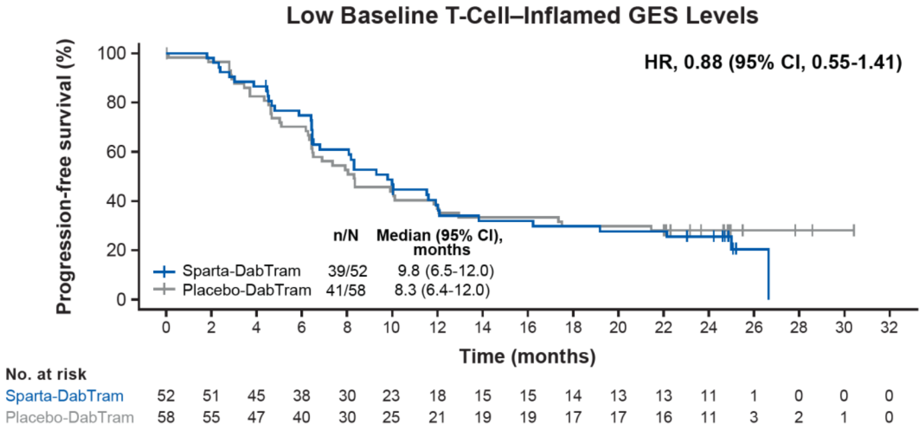

Tawbi HA, *et al. J Immunother Cancer* 2022; 10:e004226. doi: 10.1136/jitc-2021-004226

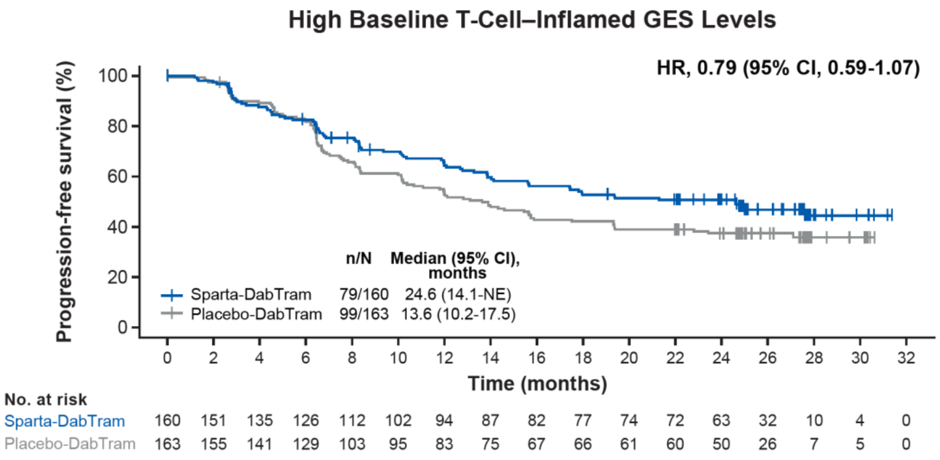
High Baseline T-Cell–Inflamed GES Levels
HR, 0.79 (95% CI, 0.59-1.07)
Progression-free survival (%)
100
80
60
40
20
0
n/N
Median (95% CI), months
Sparta-DabTram 79/160 24.6 (14.1-NE)
Placebo-DabTram 99/163 13.6 (10.2-17.5)
0 2 4 6 8 10 12 14 16 18 20 22 24 26 28 30 32
Time (months)
No. at risk
Sparta-DabTram 160 151 135 126 112 102 94 87 82 77 74 72 63 32 10 4 0
Placebo-DabTram 163 155 141 129 103 95 83 75 67 66 61 60 50 26 7 5 0

Figure S8. **Treatment benefit based on T-cell phenotype.** Shown are Kaplan-Meier estimates of progression-free survival (left) and overall survival (right) based on the inflamed (N = 139) (A), excluded (N = 163) (B), and immune desert (N = 115) (C) CD8^{+} tumor-infiltrating lymphocyte phenotypes in each treatment arm. CD, cluster of differentiation; HR, hazard ratio; placebo-DabTram, placebo plus dabrafenib and trametinib; sparta-DabTram, spartalizumab plus dabrafenib and trametinib.

A

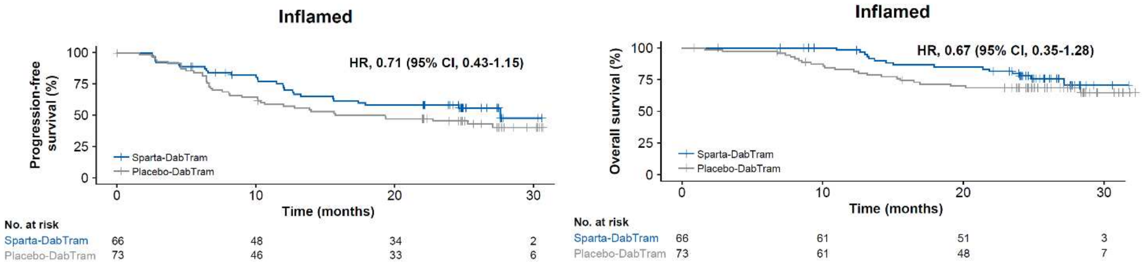

B

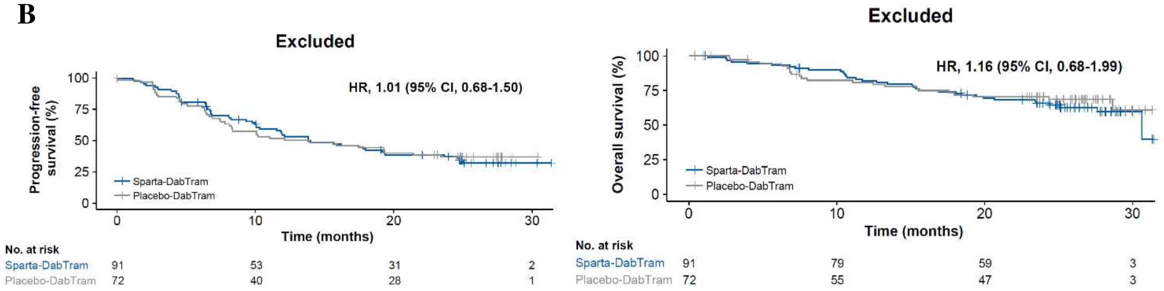

C

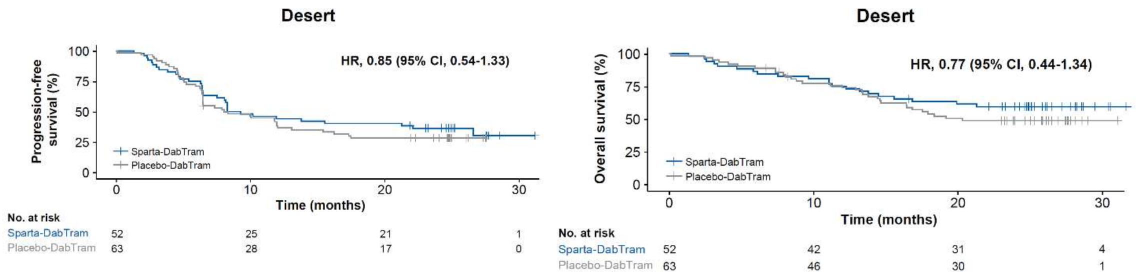

Figure S9. **Additional results from immunophenotyping of peripheral blood mononuclear cells at baseline and after 4 weeks of treatment.** Immunophenotyping of peripheral blood mononuclear cells using markers for T-cell activation and proliferation in plasma samples (N = 323) taken at baseline and after 4 weeks of treatment. Shown are the levels of proliferating $CD8^+$ T cells (A), activated $CD4^+$ T cells (B), and proliferating $PD\text{-}1^+$/ $CD4^+$ T cells (C). CD, cluster of differentiation; HLA-DR, human leukocyte antigen, DR subtype; PD-1, programmed death receptor 1; placebo-DabTram, placebo plus dabrafenib and trametinib; sparta-DabTram, spartalizumab plus dabrafenib and trametinib.

A

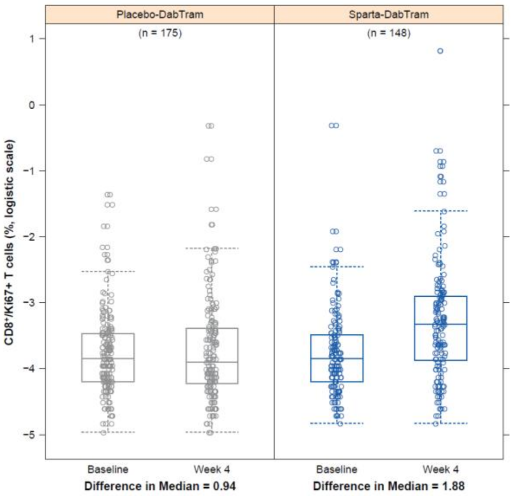

B

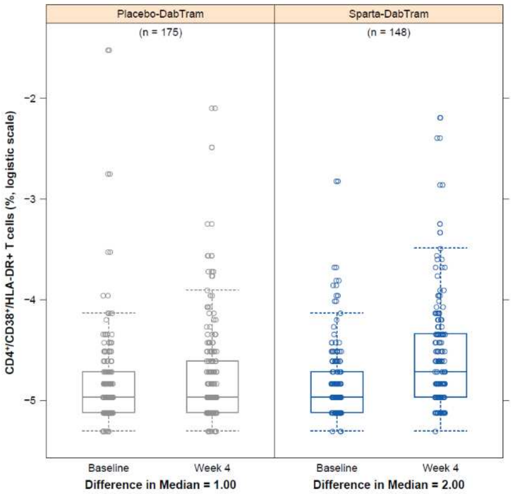

C

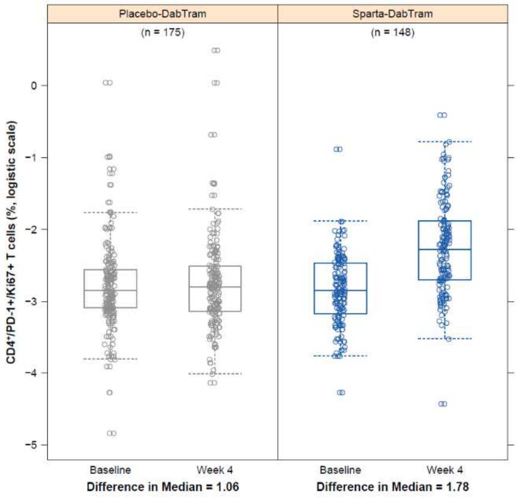
Placebo-DabTram
Sparta-DabTram
(n = 175)
(n = 148)
CD4+/PD-1+/Ki67+ T cells (%, logistic scale)
0
−1
−2
−3
−4
−5
Baseline
Week 4
Baseline
Week 4
Difference in Median = 1.06
Difference in Median = 1.78

***Figure S10*. Additional results from cytokine profiling at baseline and after 4 weeks of treatment.** Shown are the levels of CXCL10 (N = 474) (A), IL-15 (N = 473) (B), IL-18 (N = 467) (C), IL-17A (N = 473) (D), and IL-12p40 (N = 473) (E) in plasma samples taken at baseline and after 4 weeks of treatment. CXCL, C-X-C motif chemokine ligand; IL, interleukin; placebo-DabTram, placebo plus dabrafenib and trametinib; sparta-DabTram, spartalizumab plus dabrafenib and trametinib.

A

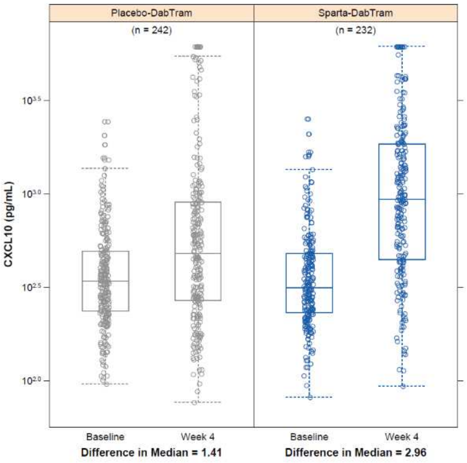

B

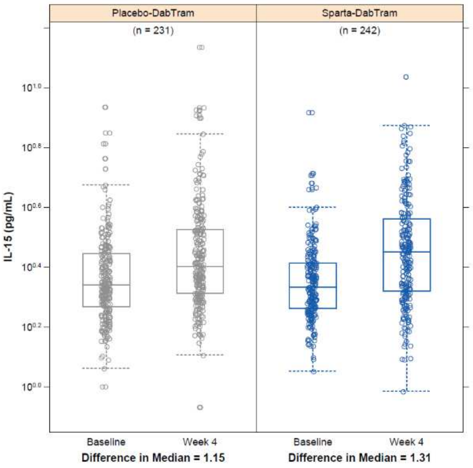

C

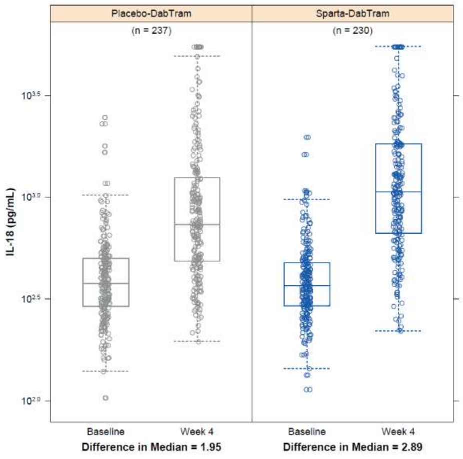
Placebo-DabTram
Sparta-DabTram
(n = 237)
(n = 230)
IL-18 (pg/mL)
Baseline
Week 4
Difference in Median = 1.95
Difference in Median = 2.89

D

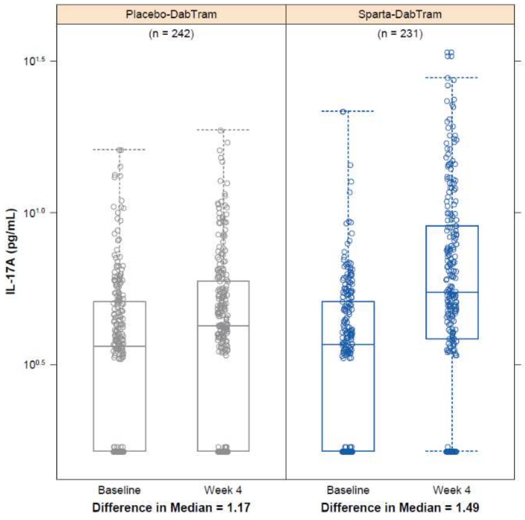
Placebo-DabTram
Sparta-DabTram
(n = 242)
(n = 231)
IL-17A (pg/mL)
Baseline
Week 4
Difference in Median = 1.17
Difference in Median = 1.49

E

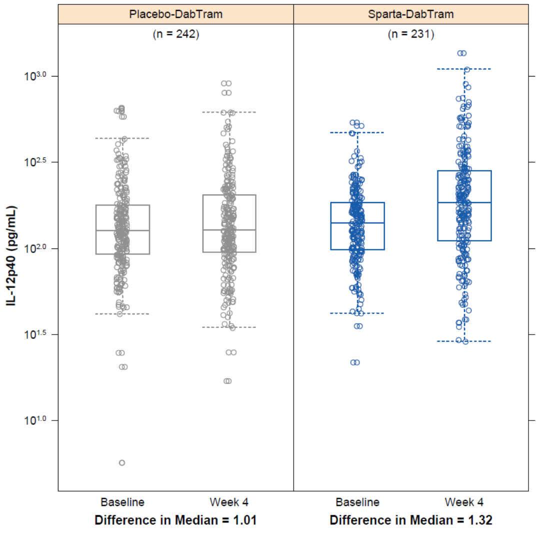
Placebo-DabTram
Sparta-DabTram
(n = 242)
(n = 231)
IL-12p40 (pg/mL)
Baseline
Week 4
Difference in Median = 1.01
Difference in Median = 1.32

Figure S11. **Progression-free survival based on baseline peripheral CD4⁺/CD8⁺ T-cell ratio and treatment arm.** Shown are Kaplan-Meier estimates of progression-free survival based on CD4⁺/CD8⁺ T-cell ratios at baseline in peripheral blood mononuclear cell samples from patients randomized to the placebo-DabTram arm (N = 208) (A) or the sparta-DabTram arm (N = 200) (B). HR, hazard ratio; placebo-DabTram, placebo plus dabrafenib and trametinib; sparta-DabTram, spartalizumab plus dabrafenib and trametinib.

A

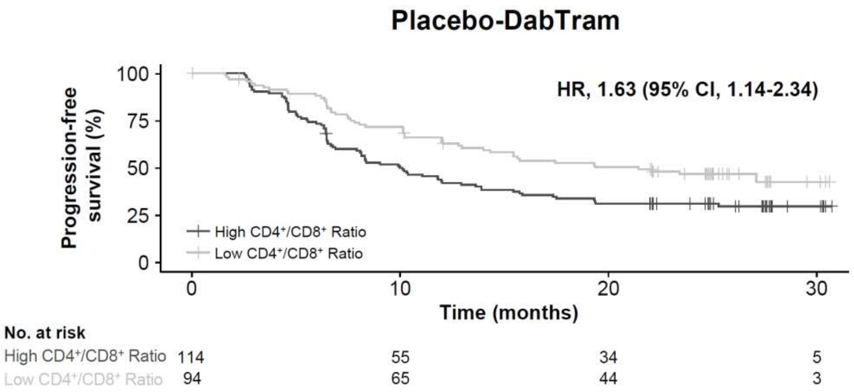

B

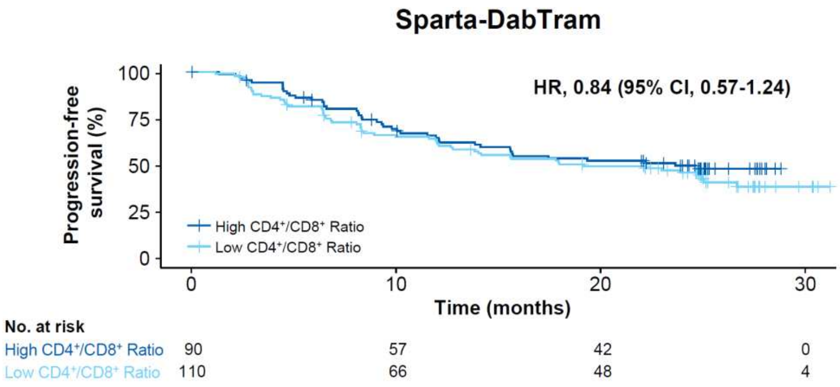

Tawbi HA, *et al. J Immunother Cancer* 2022; 10:e004226. doi: 10.1136/jitc-2021-004226

Figure S12. **Baseline ctDNA shedding correlates with disease burden.** Baseline ctDNA shedding based on levels of lactate dehydrogenase (N = 391) (A), sum of lesion diameters (N = 474) (B), and number of target lesions (N = 474) (C). ctDNA, circulating tumor DNA; LDH, lactate dehydrogenase.

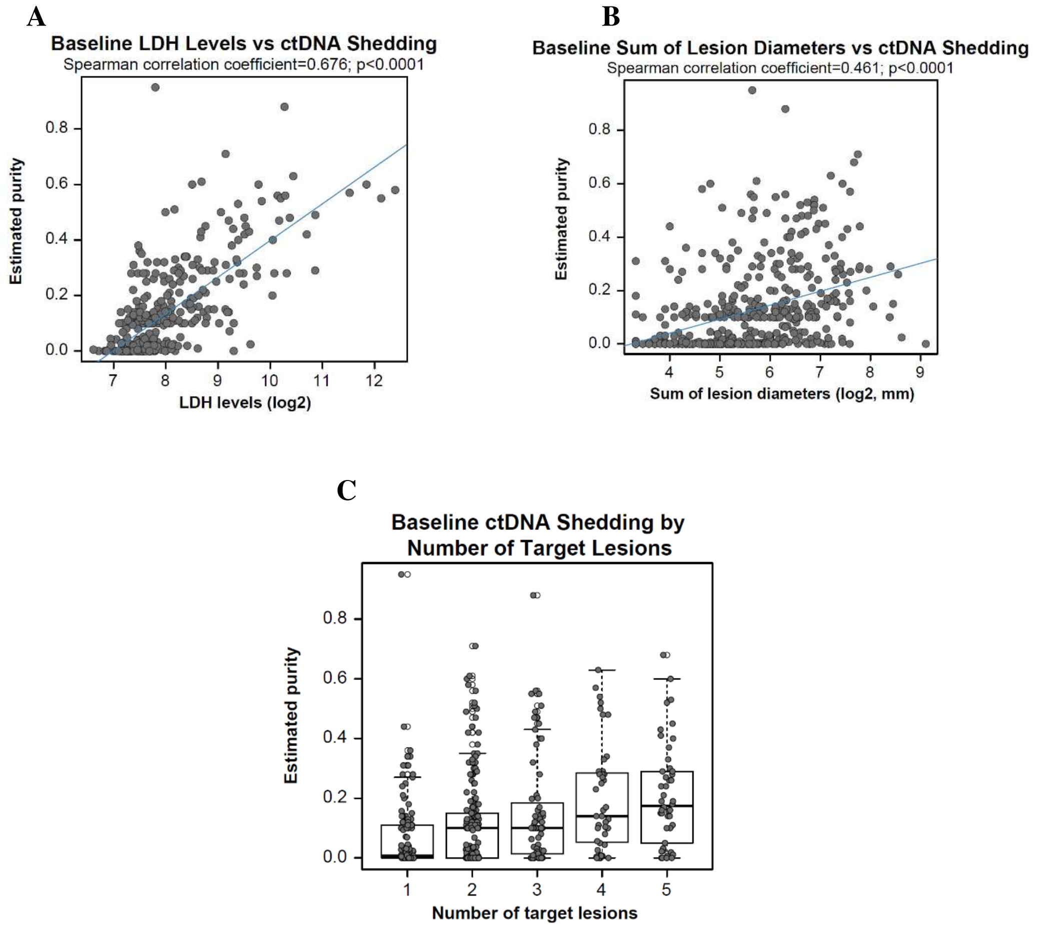

Tawbi HA, *et al. J Immunother Cancer* 2022; 10:e004226. doi: 10.1136/jitc-2021-004226

Figure S13. **Baseline ctDNA shedding based on best overall response and treatment arm.** Baseline ctDNA shedding based on best overall response of patients treated with placebo-DabTram (N = 246) (A) or sparta-DabTram (N = 234) (B). BOR, best overall response; cfDNA, cell-free DNA; CR, complete response; ctDNA, circulating tumor DNA; PD, progressive disease; placebo-DabTram, placebo plus dabrafenib and trametinib; PR, partial response; SD, stable disease; sparta-DabTram, spartalizumab plus dabrafenib and trametinib; UNK, unknown.

A

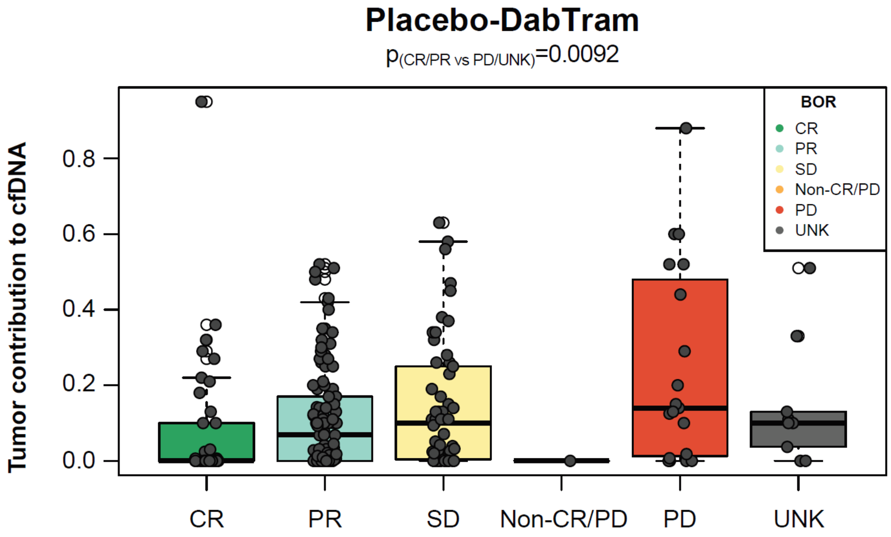

B

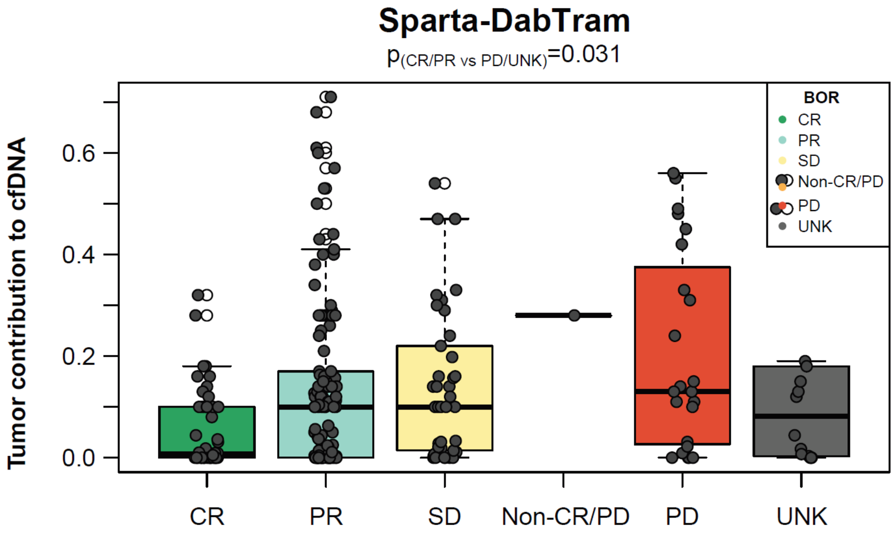

REFERENCES

1 Cristescu R, Mogg R, Ayers M, et al. Pan-tumor genomic biomarkers for PD-1 checkpoint blockade-based immunotherapy. *Science* 2018;362:eaar3593.

2 Ribas A, Robert C, Schachter J, et al. Tumor mutational burden (TMB), T cell-inflamed gene expression profile (GEP) and PD-L1 are independently associated with response to pembrolizumab (pembro) in patients with advanced melanoma in the KEYNOTE (KN)-006 study [abstract]. *Cancer Res* 2019;79(Suppl):4217.

3 Dolled-Filhart M, Gustavson M, Camp RL, et al. Automated analysis of tissue microarrays. *Methods Mol Biol* 2010;664:151–62.

4 Kluger HM, Zito CR, Barr ML, et al. Characterization of PD-L1 expression and associated T-cell infiltrates in metastatic melanoma samples from variable anatomic sites. *Clin Cancer Res* 2015;21:3052–60.

5 Schalper KA, Brown J, Carvajal-Hausdorf D, et al. Objective measurement and clinical significance of TILs in non-small cell lung cancer. *J Natl Cancer Inst* 2015;107:dju435.

6 Schalper KA, Carvajal-Hausdorf D, McLaughlin J, et al. Differential expression and significance of PD-L1, IDO-1, and B7-H4 in human lung cancer. *Clin Cancer Res* 2017;23:370–8.

7 Siska PJ, Johnpulle RAN, Zhou A, et al. Deep exploration of the immune infiltrate and outcome prediction in testicular cancer by quantitative multiplexed immunohistochemistry and gene expression profiling. *Oncoimmunology* 2017;6:e1305535.

8 Wimberly H, Brown JR, Schalper K, et al. PD-L1 expression correlates with tumor-infiltrating lymphocytes and response to neoadjuvant chemotherapy in breast cancer. *Cancer Immunol Res* 2015;3:326–32.

9 Dobin A, Davis CA, Schlesinger F, et al. STAR: ultrafast universal RNA-seq aligner. *Bioinformatics* 2013;29:15–21.

10 Anders S, Pyl PT, Huber W. HTSeq--a Python framework to work with high-throughput sequencing data. *Bioinformatics* 2015;31:166–9.

11 Robinson MD, McCarthy DJ, Smyth GK. edgeR: a Bioconductor package for differential expression analysis of digital gene expression data. *Bioinformatics* 2010;26:139–40.

12 FoundationOne CDx. Technical information. Foundation Medicine, Inc.; 2017. Available: https://info.foundationmedicine.com/hubfs/FMI%20Labels/FoundationOne_CDx_Label_Technical_Info.pdf [Accessed 8 September 2021].

13 Chalmers ZR, Connelly CF, Fabrizio D, et al. Analysis of 100,000 human cancer genomes reveals the landscape of tumor mutational burden. *Genome Med* 2017;9:34.

14 Li H, Durbin R. Fast and accurate short read alignment with Burrows-Wheeler transform. *Bioinformatics* 2009;25:1754–60.

15 McKenna A, Hanna M, Banks E, et al. The Genome Analysis Toolkit: a MapReduce framework for analyzing next-generation DNA sequencing data. *Genome Res* 2010;20:1297–303.

16 DePristo MA, Banks E, Poplin R, et al. A framework for variation discovery and genotyping using next-generation DNA sequencing data. *Nat Genet* 2011;43:491–8.

17 Cibulskis K, Lawrence MS, Carter SL, et al. Sensitive detection of somatic point mutations in impure and heterogeneous cancer samples. *Nat Biotechnol* 2013;31:213–9.

18 Ye K, Schulz MH, Long Q, et al. Pindel: a pattern growth approach to detect break points of large deletions and medium sized insertions from paired-end short reads. *Bioinformatics* 2009;25:2865–71.

19 Riester M, Singh AP, Brannon AR, et al. PureCN: copy number calling and SNV classification using targeted short read sequencing. *Source Code Biol Med* 2016;11:13.

20 Schroder J, Hsu A, Boyle SE, et al. Socrates: identification of genomic rearrangements in tumour genomes by re-aligning soft clipped reads. *Bioinformatics* 2014;30:1064–72.

21 Oh S, Geistlinger L, Ramos M, et al. Reliable analysis of clinical tumor-only whole-exome sequencing data. *JCO Clin Cancer Inform* 2020;4:321–35.

22 Dummer R, Long GV, Robert C, et al. Randomized phase III trial evaluating spartalizumab plus dabrafenib and trametinib for BRAF V600-mutant unresectable or metastatic melanoma. *J Clin Oncol* 2022:JCO2101601.
